# A Model *Roseobacter*, Ruegeria pomeroyi DSS-3, Employs a Diffusible Killing Mechanism To Eliminate Competitors

**DOI:** 10.1128/mSystems.00443-20

**Published:** 2020-08-11

**Authors:** Garrett C. Sharpe, Scott M. Gifford, Alecia N. Septer

**Affiliations:** a Environment, Ecology, and Energy Program, University of North Carolina at Chapel Hill, Chapel Hill, North Carolina, USA; b Department of Marine Sciences, University of North Carolina at Chapel Hill, Chapel Hill, North Carolina, USA; Northwestern University

**Keywords:** competition, marine bacteria, roseobacters, gamma-butyrolactone, roseobacter

## Abstract

The *Roseobacter* clade is a group of alphaproteobacteria that have diverse metabolic and regulatory capabilities. They are abundant in marine environments and have a substantial role in marine ecology and biogeochemistry. However, interactions between roseobacters and other bacterioplankton have not been extensively explored. In this study, we identify a killing mechanism in the model roseobacter Ruegeria pomeroyi DSS-3 by coculturing it with a group of phylogenetically diverse bacteria. The killing mechanism is diffusible and occurs when cells are grown both on surfaces and in suspension and is dependent on cell density. A screen of random transposon mutants revealed that the killing phenotype, as well as resistance to killing, require genes within an ∼8-kb putative gamma-butyrolactone synthesis gene cluster, which resembles similar pheromone-sensing systems in actinomycetes that regulate secondary metabolite production, including antimicrobials. Transcriptomics revealed the gene cluster is highly upregulated in wild-type DSS-3 compared to a nonkiller mutant when grown in liquid coculture with a roseobacter target. Our findings show that *R. pomeroyi* has the capability to eliminate closely and distantly related competitors, providing a mechanism to alter the community structure and function in its native habitats.

**IMPORTANCE** Bacteria carry out critical ecological and biogeochemical processes and form the foundations of ecosystems. Identifying the factors that influence microbial community composition and the functional capabilities encoded within them is key to predicting how microbes impact an ecosystem. Because microorganisms must compete for limited space and nutrients to promote their own propagation, they have evolved diverse mechanisms to outcompete or kill competitors. However, the genes and regulatory strategies that promote such competitive abilities are largely underexplored, particularly in free-living marine bacteria. Here, genetics and omics techniques are used to investigate how a model marine bacterium is capable of quickly eliminating natural competitors in coculture. We determined that a previously uncharacterized horizontally acquired gene cluster is required for this bacterium to kill diverse competitors. This work represents an important step toward understanding the mechanisms bacterial populations can use to become dominant members in marine microbial communities.

## Abstract (precis)

Bacteria carry out critical ecological and biogeochemical processes and form the foundations of ecosystems. Identifying the factors that influence microbial community composition and the functional capabilities encoded within them is key to predicting how microbes impact an ecosystem. Because microorganisms must compete for limited space and nutrients to promote their own propagation, they have evolved diverse mechanisms to outcompete or kill competitors. However, the genes and regulatory strategies that promote such competitive abilities are largely underexplored, particularly in free-living marine bacteria. Here, genetics and omics techniques are used to investigate how a model marine bacterium is capable of quickly eliminating natural competitors in coculture. We determined that a previously uncharacterized horizontally acquired gene cluster is required for this bacterium to kill diverse competitors. This work represents an important step toward understanding the mechanisms bacterial populations can use to become dominant members in marine microbial communities.

## INTRODUCTION

Roseobacters are abundant members of marine microbial communities, comprising up to 20% of coastal and open ocean communities (1). Their prevalence in the marine environment is largely attributed to their diverse functional capabilities, including a wide range of metabolic strategies (2). Given their prevalence and metabolic versatility, roseobacters can have a substantial role in marine biogeochemical cycles, including the breakdown and release of carbon, sulfur, and other elements (3–10). Although studies have investigated the genes and regulatory mechanisms governing roseobacters’ biogeochemically important functions, less is known about how roseobacters interact with other bacteria to influence community structure and function (2, 4, 11–14). Specifically, the factors and mechanisms that may allow certain roseobacter strains to dominate a community remain largely unknown.

Two strategies to increase fitness within a community include exploitative and interference competition. Although exploitative competition uses population-specific metabolic strategies to outgrow a competitor, interference competition employs molecular mechanisms to directly kill or inhibit competitors (15). Indeed, some roseobacter strains have the capacity to produce antimicrobials and behave antagonistically toward other bacteria (16, 17). *Leisingera* sp. strain JC1, isolated from the jelly coat of Hawaiian bobtail squid eggs, produces the antimicrobial indigoidine, which, combined with secondary metabolites produced by other jelly coat microbes, was hypothesized to protect eggs from fouling microorganisms (18, 19). Phaeobacter gallaeciensis produces the antimicrobial tropodithietic acid (TDA) when grown in coculture with the coccolithophore Emiliania huxleyi. TDA protects the algae from pathogens, and P. gallaeciensis receives nutrients in return. When the algal bloom begins to senesce, P. gallaeciensis produces antialgal compounds known as roseobacticides that cause the mutualistic relationship to transition to parasitism (20). Finally, extracts from 14 roseobacter strains were found to produce compounds that inhibit the gammaproteobacterium Vibrio anguillarum (17). As these examples demonstrate, antimicrobial and antialgal production by roseobacters can support beneficial relationships with their symbiotic partners and prevent competitor bacteria from gaining a foothold in preferred niches. Given that roseobacters are known to make up a large part of marine microbial assemblages and represent a mostly unexplored source of antimicrobials, further study of roseobacter interference competition mechanisms is important to understand how this diverse group shapes the communities they inhabit, which ultimately impacts the ecological services that these communities provide.

In this study, we used coculture assays to determine the potential for model roseobacter Ruegeria pomeroyi DSS-3 to kill competitors and then employed a random mutagenesis approach to identify the killing mechanism. We chose *R. pomeroyi* because it has a relatively large genome (4.2 Mb) encoding multiple metabolic pathways and accessory functions for its generalist lifestyle (21–23). It is best known as a model organism for studying marine carbon and sulfur cycling (24–26). Although a previous study found *R. pomeroyi* can produce inhibitory compounds (17), the mechanisms and targets for *R. pomeroyi* killing have not been investigated. In this study, we describe a diffusible, cell density-dependent killing mechanism that DSS-3 uses to outcompete a phylogenetically diverse range of marine bacteria. The genes required for killing competitors appear to be horizontally acquired and are a fitness cost to DSS-3, suggesting that these genes may be selected for in the environment.

## RESULTS

### *R. pomeroyi* uses interference competition to kill phylogenetically diverse bacteria.

To determine whether *R. pomeroyi* uses exploitative or interference mechanisms to outcompete other roseobacter strains, coculture assays were conducted in which DSS-3 was coincubated with six different roseobacter strains: Sagittula stellata E-37 (5), *Roseovarius* sp. strain TM1035 (27), *Sulfitobacter* sp. strain RAM1190, Phaeobacter caeruleus ANS2052, Phaeobacter daeponensis, and *Ruegeria* sp. strain RAM1602. DSS-3 was chromosomally tagged with a transposon expressing a kanamycin resistance gene (DSS-3 Kn), and competitor strains were differentially tagged with a stable plasmid to quantify each strain in a mixed culture by plating dilution series onto antibiotic plates selective for DSS-3 or the competitor strain. Competitor strains were incubated alone, or mixed with DSS-3 Kn at a 1:1 ratio, based on optical density, and spotted onto 1/2 yeast tryptone sea salts (YTSS) agar. The percent recovery of each strain was calculated by dividing the CFU after 24 h of coincubation by the initial CFU count at the start of the experiment. To determine whether a roseobacter strain was outcompeted by DSS-3, the percent recovery of each competitor strain with DSS-3 was compared to the recovery of the competitor strain when incubated alone. Using this approach, we were able to determine if a competitive strategy is being used and distinguish between exploitative (outcompeting for resources) and interference (growth inhibition or killing) mechanisms. We anticipated three possible outcomes: (i) recovery of competitor strain is the same when grown alone or with DSS-3, suggesting no competitive interaction; (ii) recovery of the competitor strain is less than the starting CFU (% recovery < 100), indicating competitor cells were killed (interference competition); or (iii) percent recovery of competitor strain is >100 in both incubations but is significantly lower when grown with DSS-3, compared to competitor strain alone (suggesting an exploitative mechanism).

**FIG 1:**
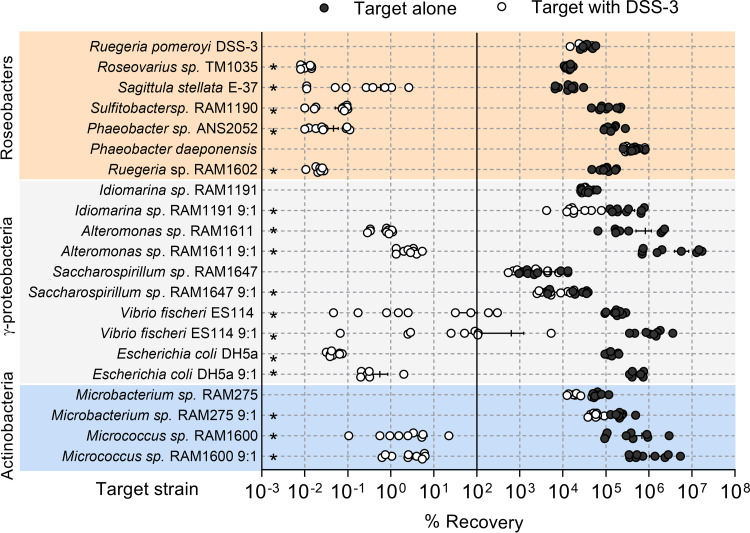
Effect of coincubation with Ruegeria pomeroyi DSS-3 on the growth of phylogenetically diverse bacteria. Percent recovery for each species after 24 h when incubated in monoculture with itself (black) or in coculture with DSS-3 (white) on 1/2 YTSS agar, where less than 100% recovery corresponds to a decrease from the initial CFU count and greater than 100% recovery corresponds to an increase from the initial CFU count. Asterisks denote *P < *0.05 using a Student *t* test comparing percent recovery of each species alone to percent recovery when coincubated with DSS-3. Error bars indicate standard error.

After coincubation with DSS-3 at an initial 1:1 ratio, the recovery of every competing roseobacter was significantly lower than its growth in monoculture, except for the control where DSS-3 was coincubated with itself, and for *P. daeponensis* (Student’s *t* test, *P < *0.05) (Fig. 1). However, when DSS-3 was mixed with *P. daeponensis* at a 9:1 ratio, this resulted in a similar reduction of *P. daeponensis* recoveries as observed with the other roseobacter strains (Fig. 1). Furthermore, the percent recovery of the competitor strains was less than 100%, indicating that fewer viable cells were recovered after 24 h than when the assay began. This substantial decrease in viable cell counts (in some cases resulting in no recovery of the competitor strain) indicates strains are being killed in the presence of DSS-3, which suggests DSS-3 is capable of interference competition.

To determine whether DSS-3 can inhibit more distantly related bacteria, several marine gammaproteobacteria and actinobacteria were selected as competitors: *Alteromonas* sp. strain RAM1611, *Saccharospirillum* sp. strain RAM1647, *Idiomarina* sp. strain RAM1191, Vibrio fischeri ES114, Escherichia coli DH5α, Microbacterium phyllosphaerae RAM275, and *Micrococcus* sp. strain RAM1600. These strains were initially mixed at a 1:1 ratio (DSS-3 to competitor) as done with the roseobacter coculture assays, but 9:1 ratios were also performed to determine if inhibition occurs at higher starting DSS-3 cell densities. In the 1:1 ratio experiments, the *Idiomarina*, *Saccharospirillum*, and *Microbacterium* strains did not show statistically significant decreases in recovery when coincubated with DSS-3 Kn, whereas the *Alteromonas*, *Vibrio*, *Escherichia*, and *Micrococcus* strains did (Student’s *t* test, *P < *0.05). In the 9:1 ratio experiments, all gammaproteobacterial and actinobacterial strains tested had significantly lower recoveries when cocultured with DSS-3 Kn than in monoculture (Fig. 1). Moreover, for coincubations where DSS-3 had an inhibitory effect, the percent recovery of the target strains was often well below 100%, indicating that the number of viable cells recovered at the end of the experiment was lower than the starting number. Taken together, these data suggest that DSS-3 uses an unknown mechanism to kill competitor strains during growth on surfaces.

### *R. pomeroyi* kills competitors in suspension.

**FIG 2:**
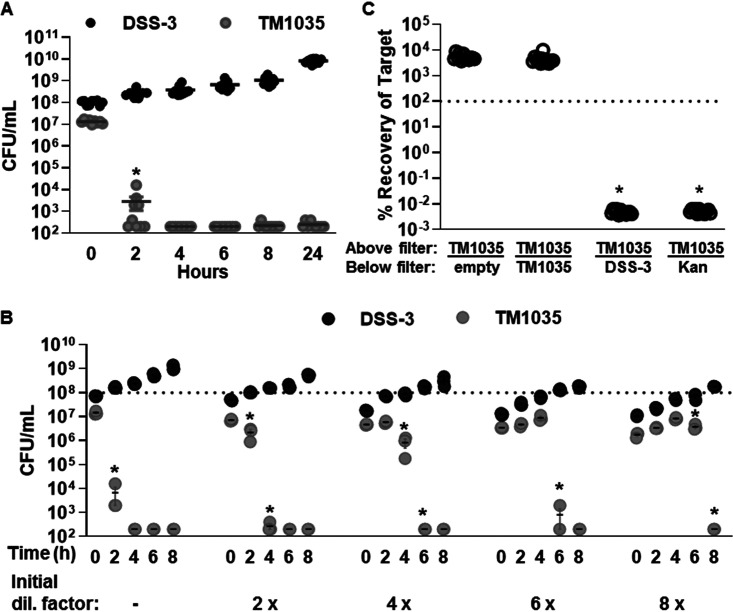
DSS-3 can kill in suspension using a diffusible, density-dependent mechanism. Cell density (CFU/ml) of DSS-3 Kn (black) and TM1035 (gray) in liquid coculture. (A) All 9 replicates for the undiluted liquid competition assay. (B) A representative experiment for the liquid dilution experiment. Asterisks denote time points where TM1035 CFU/ml are statistically lower than that of the previous time point (*P < *0.05, Student’s *t* test). (C) Percent recovery of tagged TM1035 at 24 h when grown on a filter above an empty control, a differentially tagged TM1035 strain, DSS-3, or 2 μg kanamycin antibiotic. Asterisks denote *P < *0.0001 using a Student *t* test comparing each experimental condition to the TM1035/empty control. Error bars indicate standard error, although some are too small to be seen.

To determine whether DSS-3 killing can also occur in liquid suspension, DSS-3 was coincubated for 24 h with the susceptible target strain *Roseovarius* sp. strain TM1035 in liquid medium at a 9:1 starting ratio (DSS-3 to TM1035), and CFU were quantified for each strain at various time points during the 24-h coincubation. Two hours after the start of the incubation, TM1035 CFU were reduced by 4 orders of magnitude when coincubated with DSS-3 in liquid medium, and by 4 h the TM1035 abundance was reduced below the limit of detection (200 CFU/ml) (Fig. 2A). These results, which show DSS-3 can rapidly eliminate a competitor strain to undetectable levels, provide further evidence that the competitive strategy used by DSS-3 is an interference mechanism, not competition for resources.

Given that some killing mechanisms are dependent on cell density (28–35), and we sometimes observed inhibition only when DSS-3 initially outnumbered its competitor (Fig. 1), we next examined whether the DSS-3 killing phenotype might be correlated with population density in culture. Liquid competition assays were conducted as described above, except the starting densities of both DSS-3 and TM1035 in the coculture were diluted by 2-, 4-, 6-, and 8-fold. If diluting the initial coculture cell density delays DSS-3 killing, then killing activity requires a particular cell density to promote the killing function. For each dilution tested, a statistically significant reduction of TM1035 target cells did not occur until DSS-3 reached densities of ∼10^8^ CFU ml^−1^ (Fig. 2B). This result suggests that, under the conditions used here, DSS-3 must achieve a cellular concentration threshold of >10^8^ cells ml^−1^ before killing is detected and that this interference competition mechanism may be controlled in a density-dependent manner.

### *R. pomeroyi* uses a diffusible killing mechanism.

Interference competition strategies include diffusible molecules, as is the case for most conventional antibiotics, or mechanisms that require direct cell-cell contact for transfer of a cytotoxic effector from killer to target cells (36–39). To determine whether DSS-3’s killing phenotype is contact dependent or diffusible, DSS-3 was cocultured with TM1035 on agar plates as described above, except the two strains were separated by an 0.22-μm nitrocellulose filter, which prevents physical contact between the two strains while allowing diffusible molecules to be exchanged. When tagged TM1035 was spotted on a filter with nothing below, or with untagged TM1035 below the filter, the percent recovery of tagged TM1035 was above 100% (Fig. 2C). However, if tagged TM1035 was spotted onto a filter above DSS-3 or kanamycin antibiotic, TM1035 CFU were reduced below the limit of detection (Fig. 2C). These results suggest that DSS-3 employs a diffusible killing mechanism that does not require direct contact with target cells.

### Random transposon mutagenesis yields nonkiller DSS-3 mutants.

**FIG 3:**
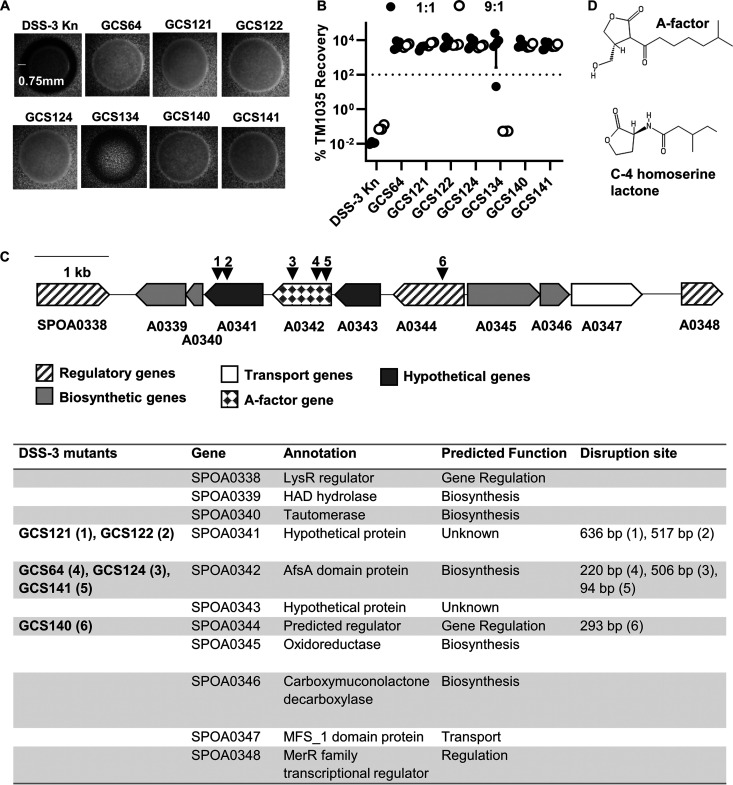
Characterization of DSS-3 mutants that have lost the killing phenotype. (A) Microscope images of DSS-3 Kn tagged wild type and seven identified nonkiller mutants when grown on fluorescent TM1035 agar overlay plates. Scale bar denotes length of inhibition zone if killing occurs. (B) Percent recovery of TM1035 after 24 h of coincubation with the DSS-3 Kn control or each potential nonkiller mutant at a 1:1 (black circles) and 9:1 (white circles) killer-to-target strain OD_600_ ratio. Asterisks denote significant reduction (*P < *0.05, Student’s *t* test) compared to the DSS-3 Kn control. Error bars indicate standard deviation. (C) Diagram of putative GBL synthesis gene cluster on the megaplasmid. Black triangles and numbers correspond to transposon insertion mutants listed in the table below. (D) Structures for quorum molecules A-factor GBL and C-4 homoserine lactone.

To identify the genes and possible mechanism(s) required for DSS-3 killing, we generated a random transposon library and screened it for DSS-3 mutants that can no longer kill a competitor strain. The screen was based on the observation that when wild-type DSS-3 is grown on a lawn of fluorescently tagged TM1035 target cells, a distinct zone of killing is observed around the DSS-3 colony (Fig. 3A). If the transposon disrupts a gene required for killing, then the DSS-3 mutant colony will not produce a zone of killing. We screened 10,000 DSS-3 mutants and isolated seven nonkiller mutants that either were unable to produce a zone of killing of TM1035 or displayed an intermediate zone of killing (Fig. 3A).

To confirm that these mutants could no longer kill, DSS-3 mutants were coincubated with TM1035 on agar surfaces as described above using both a 1:1 and 9:1 starting ratio of DSS-3 to TM1035 target cells. Of the seven mutants, six were unable to kill TM1035 after 24 h at either starting ratio (Fig. 3B). However, one mutant (GCS134), which exhibited only partial killing on agar overlay plates (Fig. 3A), was not as efficient at killing target at a 1:1 starting ratio (Fig. 3B, filled circles) but was able to kill when initially outnumbering the target at a 9:1 starting ratio (Fig. 3B, empty circles). These results suggest that six of the isolated mutants lost the ability to kill, and one mutant (GCS134) displayed reduced killing ability that could be restored by increasing its population size at the beginning of the coincubation experiment.

### The genes required for killing are located in a putative gamma-butyrolactone (GBL) biosynthesis gene cluster.

The locations of the transposon insertions were mapped using inverse PCR (iPCR). For mutant GCS134, whose killing ability was restored at a 9:1 starting ratio, the transposon insertion site mapped to a sulfate adenylyltransferase (ATP sulfurylase) gene (SPO0900), which is predicted to mediate the cellular assimilation of inorganic sulfur (40–42). We hypothesized that this mutant, which could not kill TM1035 cells at a 1:1 starting ratio (Fig. 3B), has a reduced growth rate and could not achieve the threshold density required for killing. Indeed, the growth rate of GCS134 was reduced compared to the tagged DSS-3 Kn strain (see Fig. S1A in the supplemental material). Moreover, when the CFU were calculated for both the mutant and TM1035 target strains in coculture, GCS134 was unable to reach the threshold of 10^8^ CFU ml^−1^ when a 1:1 starting ratio was used and did not eliminate TM1035 (Fig. S1B). However, when GSC134 was coincubated with TM1035 at a 9:1 starting ratio, the mutant could achieve the necessary cell density and eliminate TM1035 target after a 24-h coincubation (Fig. S1B). Because GCS134 retained its ability to kill when coincubation conditions permitted it to achieve a sufficiently high cell density, we did not consider this mutant in further analysis.

10.1128/mSystems.00443-20.2 FIG S1 Mutant GCS134’s reduced growth rate accounts for its impaired ability to kill target cells in coculture. (A) Growth curves for DSS-3 wild type (WT) (black circle), DSS-3 Kn (white circle), and GCS134 (gray circle) in liquid 1/2 YTSS, incubated at 29°C. A Student *t* test was used to determine whether the growth rates (slope of growth curve in exponential growth stage) were significantly different from DSS-3 WT (asterisk indicates *P* < 0.05). (B) SPO0900 mutant GCS134 can still kill target TM1035 when a higher starting ratio is used. CFU counts for GCS134 (black circle) and target TM1035 (gray circle) after 24 h of coincubation from the 1:1 and 9:1 mutant/target starting ratios. Error bars indicate standard deviation. Download FIG S1, TIF file, 1.8 MB. Copyright © 2020 Sharpe et al. 2020 Sharpe et al. This content is distributed under the terms of the Creative Commons Attribution 4.0 International license.

The six remaining mutants contained transposon insertions in a single gene cluster located on DSS-3’s megaplasmid (Fig. 3C). According to an antiSMASH analysis (43), this gene cluster encodes a potential gamma-butyrolactone (GBL) biosynthesis pathway, including a predicted A-factor synthesis (AfsA) domain protein that is essential for GBL production in other bacteria (44, 45), additional biosynthetic proteins, three predicted transcriptional regulators, a predicted transporter, and two hypothetical proteins (Fig. 3C). GBLs, such as A-factor, are quorum sensing molecules with a structure similar to that of the well-studied quorum sensing molecule C-4 homoserine lactone (Fig. 3D) and have been shown to regulate antibiotic production and cell differentiation in streptomycetes (46–50). Together, these data revealed two important findings: (i) they indicate our screen reached saturation because we obtained multiple, independent insertions in one gene cluster, with two genes having multiple, independent transposon insertions, and (ii) the predicted GBL biosynthesis cluster is required for DSS-3 to kill a competitor strain.

### DSS-3 GBL homologs are found primarily in distantly related taxa.

**FIG 4:**
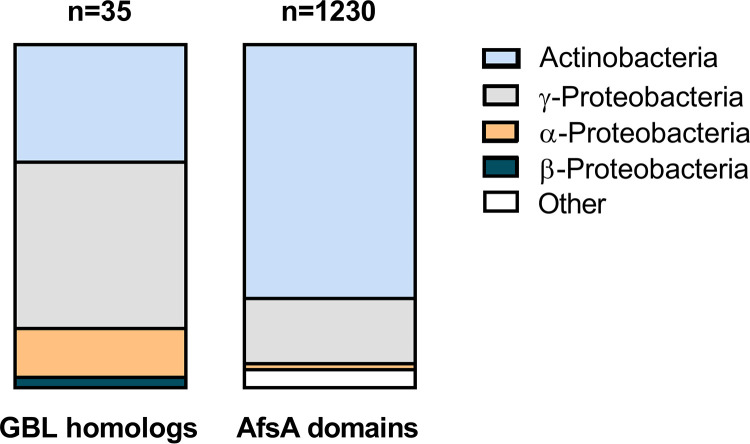
Presence of SPOA0341 to -0343 homologs and the AfsA domain (PF03756) in bacterial phyla. Homologs of genes SPOA0341, 0342 (AfsA), and 0343 with >30% identity were identified with BLASTX using nr database (left). AfsA domain (PF03756) proteins were also identified using AnnoTree (right).

Given that GBL synthesis has primarily been described in *Actinobacteria* (47, 48), we examined whether DSS-3’s GBL gene cluster may have arisen through horizontal transfer from distantly related bacteria. Homologs to the AfsA domain protein SPOA0342 and the flanking hypothetical proteins SPOA0341 and SPOA0343 were identified using BLASTx homology searches against NCBI’s nonredundant protein database in order to identify GBL presence in other bacterial clades (Table S1). Of the 35 organisms found to carry a homolog of SP0A0342, 29 carry homologs of all three genes and six carry homologs of SPOA0342 and one flanking gene. Only five of these organisms are alphaproteobacteria, two of which are roseobacters (*Ruegeria* sp. strain EL01 and Shimia marina), and the majority of organisms that carry homologs to at least two of these three GBL genes belonged to the gammaproteobacteria and actinobacteria (Fig. 4 and Table S1). We next searched more broadly for proteins containing the key A-factor domain (Pfam domain PF03756) using AnnoTree (51). Similar to the BLASTx search results, the AnnoTree results revealed that ∼93% of the total identified A-factor domain-containing proteins were found in gammaproteobacteria and actinobacteria, and less than 2% were found in roseobacters (Fig. 4). Together, these results suggest that DSS-3’s GBL gene cluster may have been acquired horizontally from either a *Gammaproteobacteria* or an *Actinobacteria* lineage.

10.1128/mSystems.00443-20.3 TABLE S1 Homologs of DSS-3 protein products encoded in SPOA0341, 0342, and 0343. Percent identity to DSS-3 protein sequence for homologs with a cutoff of >30% identity with Blastx using nr database. Download Table S1, DOCX file, 0.02 MB. Copyright © 2020 Sharpe et al. 2020 Sharpe et al. This content is distributed under the terms of the Creative Commons Attribution 4.0 International license.

### GBL genes are required to protect against self-killing.

**FIG 5:**
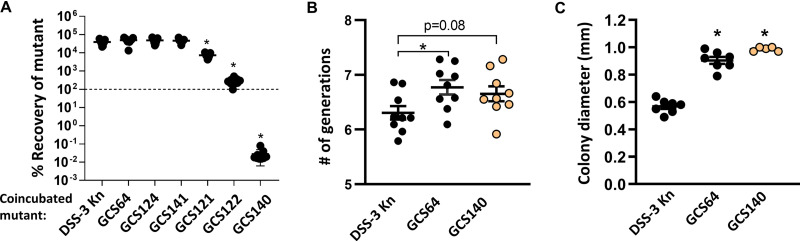
Killing ability, but not immunity function, is a fitness cost for DSS-3. (A) Percent recovery of DSS-3 Kn and each nonkiller mutant when coincubated on 1/2 YTSS agar plates with differentially tagged wild-type DSS-3 at a 1:1 ratio of DSS-3 wild type to DSS-3 mutant. Asterisks denote *P < *0.001 using a Student *t* test comparing mutant to wild type. (B) Number of generations for indicated strains spotted onto 1/2 YTSS agar and grown for 24 h. (C) Colony diameter for DSS-3 Kn and select mutants grown on 1/2 YTSS agar plates for 48 h. For panels B and C, error bars indicate standard error (*n* = 9), asterisks denote *P < *0.05 using a Student *t* test comparing mutant to DSS-3 wild type, and orange indicates a strain that is not immune to killing by parent (GCS140).

Bacteria will often produce immunity factors to prevent self-killing while employing interference competition mechanisms (52). Given that immunity genes are often carried near interference mechanisms on the genome and often coexpressed, our transposon insertions may have also disrupted DSS-3’s genetic factors for immunity. To determine whether the nonkiller mutants had become sensitive to killing, we coincubated the mutant DSS-3 strains with a differentially tagged parental DSS-3 strain using a 1:1 starting ratio and assayed percent recovery of each mutant after 24 h on surfaces. Although some mutants grew in the presence of the parent strain (>100% recovery), others were significantly inhibited or nearly eliminated. All three *afsA* (SPOA0342) mutants (GCS64, GCS124, and GCS141) showed no statistically significant reduction in percent recovery when coincubated with the parent strain, suggesting these strains retained immunity to killing (Fig. 5A). In contrast, both mutants with transposon insertions in the hypothetical protein SPOA0341 (GCS121 and GCS122) had reduced percent recoveries when coincubated with the wild type (Fig. 5A), suggesting they are sensitive to killing by the parent strain. Interestingly, we recovered ∼100-fold less of SPOA0341 mutant GCS122 than of SPOA0341 mutant GCS121, suggesting that although both mutants have a transposon insertion in the same gene, the insertion site and/or orientation of the transposon may also influence expression of immunity factors. Finally, the predicted regulator mutant (SPOA0344, GCS140) showed the highest sensitivity to killing, with the recovery of this strain reduced to below the limit of detection after 24 h coincubation with the parent, a result similar to what we observed when coincubating other sensitive roseobacter isolates with differentially tagged DSS-3. Taken together, these findings indicate that in addition to encoding the factors necessary for killing, the gene cluster also contains genes required for immunity.

### The GBL gene cluster is energetically costly.

In an analysis of the DSS-3 proteome, Christie-Oleza et al. found that five proteins encoded in the GBL gene cluster (SPOA0339 to -0343) comprise 1 to 6% of the entire DSS-3 proteome under various conditions (53). Therefore, we hypothesized that the nonkiller mutants would have a higher growth rate, due to either their reduced GBL protein synthesis or lack of production of the interference mechanism, which may also be energetically costly. To determine whether the mutants grew more quickly than the wild type on surfaces, the DSS-3 Kn, SPOA0342 *afsA* mutant GCS64, and the SPOA0344 regulator mutant GCS140 were grown alone on 1/2 YTSS agar. Two measurements were taken: (i) CFU were quantified at the beginning and after 24 h of growth on agar to calculate the number of generations for each strain type and (ii) the diameters of colonies for each strain were measured after 48 h. Both mutants had undergone more cell divisions than the DSS-3 Kn strain, although only mutant GCS64 was significantly different from wild type (Fig. 5B). Moreover, the colony diameter measurements for the two representative nonkiller mutants (one that is immune and one that is no longer immune) were approximately twice that of the wild-type strain (Fig. 5C). Taken together, these data suggest that under the conditions tested here, killing ability, but not immunity function, may be a fitness cost for DSS-3.

### *afsA* is required for transcription of the GBL gene cluster.

**FIG 6:**
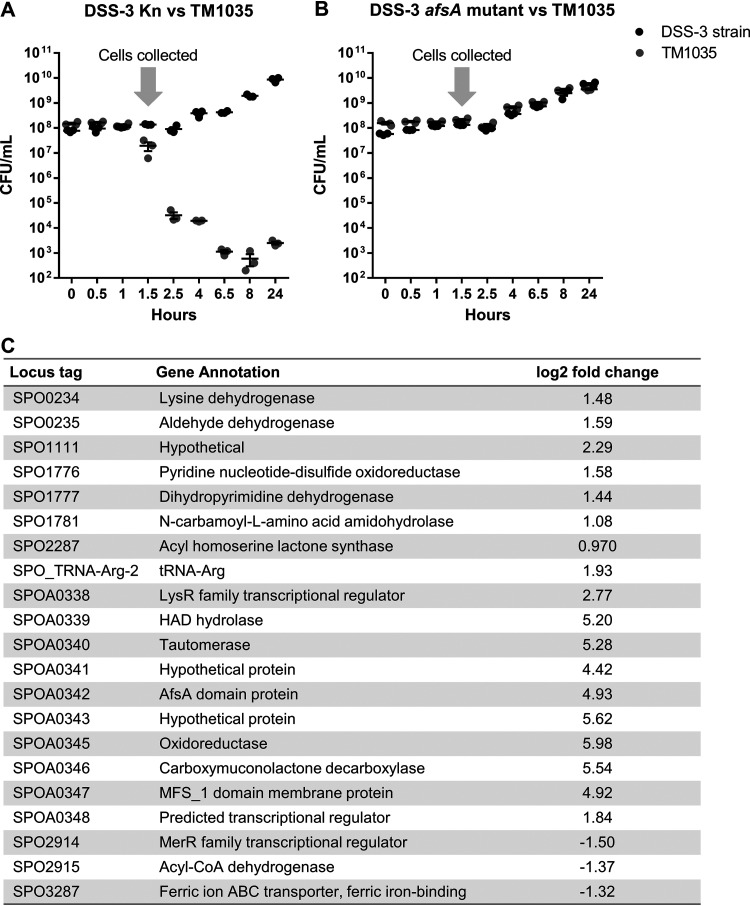
The *afsA* gene is required for transcription of GBL cluster genes in liquid coculture. (A and B) Collection of cells from coincubation experiments for transcriptome analysis of DSS-3 Kn and *afsA* mutant GCS64. DSS-3 Kn and DSS-3 mutant GCS64 were each coincubated with target strain TM1035 at a 1:1 ratio (OD_600_ of 0.2 each) and quantified by serial dilution on selective medium. Cells were collected on an 0.22-μm polyether sulfone filter at 1.5 h (arrow), and RNA was extracted from each filter and processed for transcriptome sequencing. (C) Statistically significant differentially regulated genes comparing the wild-type DSS-3 Kn and the SPOA0342 mutant GCS64 when coincubated with target *Roseobacter* strain TM1035. The log_2_ fold change denotes whether transcripts were enriched or depleted in the tagged wild-type strain DSS-3 Kn compared to the nonkiller mutant GCS64.

Because A-factor is known to regulate antimicrobial production in actinomycetes, we hypothesized that the *afsA* gene product may have a similar role in regulation of the DSS-3 killing function. To determine the AfsA-dependent regulon in DSS-3, we compared the transcriptomes of the *afsA* mutant GCS64 and DSS-3 Kn when coincubated with *Roseovarius* sp. TM1035 in liquid suspension (Table S2). The cocultured cells used for transcriptome sequencing were collected at 1.5 h when killing of TM1035 by wild-type DSS-3 begins to occur in a 1:1 liquid coculture (Fig. 6A and B). Of the 4,252 genes in the DSS-3 genome, only 21 genes were significantly differentially transcribed between DSS-3 Kn and the *afsA* mutant GCS64, 10 of which corresponded to the putative GBL cluster (Fig. 6C). Transcripts of these 10 genes were enriched by 4- to 60-fold in the wild-type strain compared to the *afsA* mutant. The only gene carried in the GBL gene cluster that was not differentially expressed was the predicted regulator encoded in SPOA0344. Taken together, these data show that (i) the *afsA* gene is required for expression of most of the genes in the GBL operon and (ii) this regulatory mechanism does not significantly impact expression of most other genes in the DSS-3 genome.

10.1128/mSystems.00443-20.4 TABLE S2 Read counts for coculture transcriptomes. Read counts for each transcriptome sample with each row corresponding to a DSS-3 gene and each column corresponding to a transcriptome sample. DvT 1 to 3 are triplicate cocultures of wild-type DSS-3 and TM1035. GCS64vT 1 to 3 are triplicate cocultures of the SPOA0342 (*afsA*) DSS-3 mutant with TM1035. Download Table S2, PDF file, 0.2 MB. Copyright © 2020 Sharpe et al. 2020 Sharpe et al. This content is distributed under the terms of the Creative Commons Attribution 4.0 International license.

### The GBL gene cluster is required for DSS-3 to outcompete phylogenetically diverse marine bacteria.

**FIG 7:**
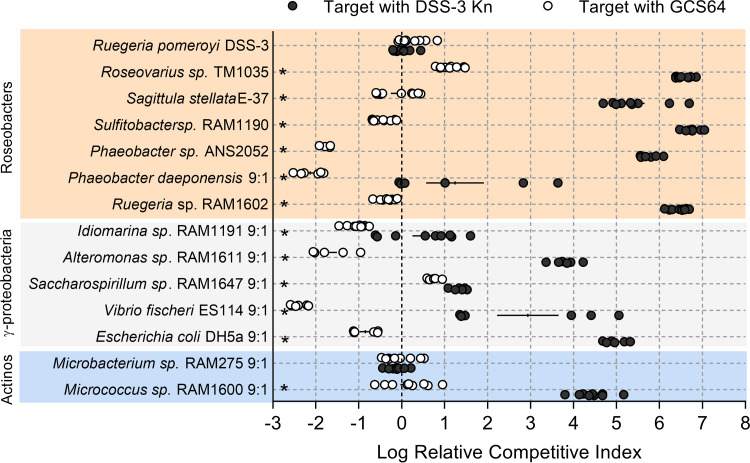
Putative GBL gene cluster is required to maintain DSS-3’s competitive advantage against phylogenetically diverse bacteria. Log relative competitive index (log RCI) for cocultures of chosen competitors with either DSS-3 Kn (black) or the *afsA* mutant GCS64 (white) after 24 h of coincubation on surfaces. Log RCI was calculated as follows: log RCI = log[(DSS-3 CFU_24HR_/competitor CFU_24HR_)/(DSS-3 CFU_0HR_/competitor CFU_0HR_)]. A positive log RCI value corresponds to DSS-3 having a competitive advantage, and a negative log RCI corresponds to the competitor strain having a competitive advantage. Asterisks denote *P < *0.05 using a Student *t* test comparing the log RCI values of competitions with DSS-3 Kn or with *afsA* mutant GCS64. Error bars indicate standard error.

To test the GBL gene cluster’s role in killing other bacterial types, DSS-3 Kn and the *afsA* mutant GCS64 were used in competition assays with the full taxonomic range of competitor strains described in Fig. 1. For the roseobacter competitions, a 1:1 starting ratio was used for all the roseobacter competitions except for *P. daeponensis*, in which case a 9:1 DSS-3-to-*P. daeponensis* ratio was used. For all *Gammaproteobacteria* and *Actinobacteria* competitions, 9:1 ratios (DSS-3 to competitor) were used. For these experiments, log relative competitive indexes (log RCIs) were calculated for each coincubation. A positive log RCI indicates that DSS-3 had a competitive advantage after coincubation, while a negative log RCI indicates that the competitor strain had an advantage. A log RCI of zero indicates that neither strain had a competitive advantage. After 24 h, all coincubations of competitor strains with DSS-3 Kn had significantly higher log RCI values than did coincubations with the nonkiller DSS-3 mutant GCS64 (Fig. 7), suggesting that expression of the GBL cluster is required for DSS-3 to outcompete these isolates in coculture. The one exception was *Microbacterium* sp. RAM275, which, when coincubated with either DSS-3 Kn or GCS64, had log RCI values near zero, suggesting the DSS-3 killing mechanism does not convey a competitive advantage against this actinobacterium under these conditions.

## DISCUSSION

Based on the data presented here, we propose the following model for how the GBL gene cluster may enable DSS-3 to eliminate competitors. DSS-3 grows together in the presence of competitor bacteria until a cell density threshold is reached, at which point the killing mechanism can eliminate phylogenetically diverse competing bacteria, significantly reducing their population sizes and enabling DSS-3 to dominate the niche space.

We found both the killing and immunity functions require genes carried in the GBL gene cluster. Given that the killing phenotype is density dependent and requires GBL biosynthesis genes, we hypothesize that a GBL-like molecule may be an antimicrobial and a high cell density is needed to achieve cytotoxic levels sufficient to kill competitor cells. Alternatively, GBL may act as a signaling molecule that combines with one or more regulators to activate production of an unknown, diffusible antimicrobial whose synthesis is regulated in a density- and GBL-dependent manner. Moreover, although some nonkiller mutants are sensitive to killing by the parent, our screen would not have allowed for identification of all immunity-related factors because disrupting immunity in a cell that still kills would be lethal. Future work should focus on identifying the molecules involved in killing and immunity and the role GBL may have in mediating interbacterial killing.

Because competition can impact microbial community structure and function, it is critical to identify the ecologically relevant habitats and conditions that support and restrict DSS-3’s killing activity. The density-dependent requirement of this killing mechanism limits the environments and microhabitats where such a competitive mechanism may be advantageous. In our study, DSS-3 needed to achieve a cell density of ∼10^8^ CFU/ml to kill a competitor. However, the cell density threshold required for killing in the marine environment may be different because environmental viscosity and cellular metabolism can influence fluid flow of the local environment, which may promote or prevent the accumulation of signaling and/or antimicrobial molecules (54, 55). For example, the phycosphere or organic particles are habitats with low diffusibility and high nutrients (56) and therefore may support growth of DSS-3 microcolonies and allow local concentrations of these molecules to activate killing. DSS-3 has already been found to establish a mutualistic relationship with the diatom Thalassiosira pseudonana in coculture (57): DSS-3 provides the limiting micronutrient B_12_ to *T. pseudonana*, and the diatom in turn produces the sulfur-carbon metabolite C-3 sulfonate 2,3-dihydroxypropane-1-sulfonate (DHSP) (57). This mutualistic exchange of resources between DSS-3 and *T. pseudonana* suggests that DSS-3 may indeed have the capacity to colonize certain phytoplankton. If DSS-3 does colonize the phycosphere, DSS-3 may be able to use the diffusible killing mechanism described here to kill competitors, significantly impacting the structure and function of the phytoplankton microbiome and ecophysiology.

We found several lines of evidence that suggest the GBL synthesis gene cluster in DSS-3 may have been a recent acquisition event. Specifically, we found that (i) the GBL synthesis gene cluster is located on a megaplasmid; (ii) homologs are absent in other *Ruegeria* species but present in disparate individuals within various families of alphaproteobacteria, gammaproteobacteria, and actinobacteria; and (iii) organisms encoding AfsA domain proteins were mostly actinobacteria and gammaproteobacteria. It is also notable that the putative GBL gene cluster identified here has previously been described only in actinobacteria as a mechanism to regulate the production of secondary metabolites, including antimicrobials (47). Therefore, the discovery of a putative GBL synthesis gene cluster outside *Actinobacteria* suggests an expanded role for GBL signaling that has not previously been considered.

In summary, this work demonstrates that *R. pomeroyi* DSS-3 encodes a broadly effective, diffusible killing mechanism that can impact the abundance of cooccurring marine bacteria, which can significantly alter the structure and function of marine microhabitats. Future work is needed to identify the molecular mechanism of this killing ability as well as the ecological niches where it is employed.

## MATERIALS AND METHODS

### Growth of bacterial strains.

Bacterial strains were grown on 1/2 YTSS plates supplemented with the appropriate antibiotic at 29°C, with the following two exceptions: V. fischeri ES114 pVSV208 was grown on Luria-Bertani with added salt (LBS) plates supplemented with the appropriate antibiotic at 24°C, and E. coli DH5α pGS001 was grown on LB plates supplemented with the appropriate antibiotic at 37°C. See Text S1 and Table S3 in the supplemental material for additional experimental details including isolation of competitor strains, construction of tagged variants, and antibiotic concentrations used for selection.

10.1128/mSystems.00443-20.1 TEXT S1 Additional experimental details including cultivation of wild-type bacterial isolates, construction of mutant strains, and transcriptome analysis. Download Text S1, DOCX file, 0.02 MB. Copyright © 2020 Sharpe et al. 2020 Sharpe et al. This content is distributed under the terms of the Creative Commons Attribution 4.0 International license.

10.1128/mSystems.00443-20.5 TABLE S3 Strains, plasmids, and oligonucleotides. Additional details on genotypes of wild-type and mutant strains and plasmids used in this study, as well as oligonucleotide sequences. Download Table S3, DOCX file, 0.02 MB. Copyright © 2020 Sharpe et al. 2020 Sharpe et al. This content is distributed under the terms of the Creative Commons Attribution 4.0 International license.

### Surface coincubation assay.

Individual colonies for each strain were placed into liquid medium, grown while shaking at 200 rpm for 24 h, and then subcultured and grown overnight under the same conditions. Overnight cultures were pelleted, and cells were resuspended in 1/2 YTSS medium and diluted to an optical density at 600 nm (OD_600_) of 1.0. The two competing strains were mixed at a 1:1 or 9:1 DSS-3/target OD_600_ ratio, and 5 μl of the mixture was spotted on 1/2 YTSS plates and incubated (29°C, 24 h). The starting population of each strain was quantified by plating serial dilutions onto 1/2 YTSS plates supplemented with antibiotics selective for each strain, and CFU were counted. After 24 h, each coincubation spot was resuspended in 1 ml 1/2 YTSS medium and quantified by plating serial dilutions.

Filter separation coincubations were set up by spotting 20 μl of an OD_600_ 1.0 culture of either differentially tagged DSS-3 or *Roseovarius* sp. TM1035, concentrated kanamycin antibiotic, or no addition (negative control) onto 1/2 YTSS plates and then placing an 0.2-μm nitrocellulose filter over the spot and spotting 5 μl of tagged TM1035 (OD_600_ = 1.0) on top of the filter. The filter separation coincubations were then incubated at 29°C for 24 h. The target strain was quantified at 0 and 24 h by plating serial dilutions onto selective medium as described above.

### Liquid suspension competitions.

Strains were cultured overnight in 15 ml 1/2 YTSS liquid medium containing the appropriate antibiotic at 29°C and shaken at 200 rpm. Cells were pelleted by centrifugation, the supernatant was removed, and the cell pellet was resuspended in 3 ml of fresh 1/2 YTSS. Each strain was then diluted to an OD_600_ of 0.2 in 10 ml 1/2 YTSS to achieve a 1:1 starting ratio. For the liquid competition assay testing the effect of cell starting densities, the 1:1 coincubation mixture was diluted with 1/2 YTSS medium to make 2-, 4-, 6-, 8-, and 10-fold-diluted starting cocultures. Cell densities (CFU/ml) of each strain were then quantified by plating serial dilutions onto selective medium at 0, 2, 4, 6, 8, and 24 h.

### Transposon mutagenesis.

The Tn*5*-Kn transposon was conjugated into *R. pomeroyi* DSS-3 via coincubation with E. coli RH03 carrying the pUT mini-Tn*5*-Kn transposon delivery plasmid. Cultures were grown overnight in 1/2 YTSS at 29°C for DSS-3, and LB at 37°C supplemented with kanamycin and diaminopimelic acid (DAP) for E. coli. The two cultures were mixed at a 4:1 (vol/vol) E. coli-to-DSS-3 ratio and pelleted by centrifugation. The supernatant was removed, and the pellet was washed with 1/2 YTSS medium and centrifuged again. All but 10 μl of the supernatant was removed, and this remaining supernatant was used to resuspend the cell pellet. The conjugation mixture was spotted on 1/2 YTSS DAP agar plates and incubated for 16 h at 29°C. After 16 h, the conjugation spot was resuspended in 1 ml 1/2 YTSS and centrifuged to pellet cells. The supernatant was removed, and the pellet was resuspended in 1/2 YTSS medium and plated onto 1/2 YTSS agar plates supplemented with kanamycin. The plates were incubated at 29°C until kanamycin-resistant DSS-3 mutant colonies were visible on the plate (2 to 3 days of incubation).

To screen for the nonkilling mutant phenotype, each DSS-3 mutant was patched onto two corresponding sets of plates: a 1/2 YTSS plate supplemented with kanamycin and an agar overlay plate containing mCherry-tagged TM1035 target strain. Patches of DSS-3 mutants that retained the ability to kill the target generated a zone of clearing around themselves on target overlay plates and were not examined further. Mutants that failed to create a zone of clearing were considered to be unable to kill, and those mutants were collected from the 1/2 YTSS kanamycin plate, cultured in 1/2 YTSS medium supplemented with kanamycin at 29°C while shaking at 200 rpm for 24 h, and stored at −80°C for further characterization. Approximately 10,000 DSS-3 transposon mutants were generated and screened.

### iPCR.

Mutants identified as losing the killing phenotype were cultured overnight in 1/2 YTSS medium, and their DNA was extracted using the ZR fungal/bacterial DNA miniprep kit (Zymo Research, Irvine, CA). The transposon insertion sites were then identified by inverse PCR (iPCR) (58). Briefly, 2 μg of mutant genomic DNA was digested overnight using the BssHII restriction enzyme (New England Biolabs, Ipswich, MA) in a 50-μl reaction mixture and then cleaned and concentrated to 20 μl using the ZR DNA Clean and Concentrate-5 kit (Zymo Research, Irvine, CA). The resulting linear genomic DNA fragments were circularized using T4 DNA ligase (New England Biolabs, Ipswich, MA). The DSS-3 DNA sequence flanking the transposon was amplified with PCR by pairing a reverse primer that anneals at the 5′ end of the transposon (5′-endseq, AS1193 or GS010) with a forward primer that anneals at the 3′ end of the transposon (3′-endseq, AS1196 or GS009). These primers anneal to the ends of the Tn*5*-Km transposon and amplify the circularized DNA fragment in between (see Text S1 for PCR cycles and conditions). Resulting PCR products were confirmed to be present using DNA electrophoresis, and the PCR products were cleaned and concentrated using the ZR DNA Clean and Concentrate-5 kit and Sanger sequenced by Eton Bio. The disrupted gene in each mutant was identified using NCBI BLAST. Mutation sites were confirmed by amplifying the transposon insertion site of uncut genomic DNA using a transposon-specific primer and a primer specific to the DSS-3 DNA near the mapped insertion site (see Text S1).

### Transcriptomes.

Liquid suspension competitions were set up as described above by mixing either DSS-3 Kn or DSS-3 SPOA0342 mutant (GCS64) with TM1035 pBBR1MCS-5 at a 1:1 ratio in triplicate, 10-ml cocultures (each strain at a starting OD_600_ of 0.2). The competition assay was subsampled regularly for 24 h to determine strain population densities via serial dilutions on selective medium plates. At 1.5 h after starting the experiment, cells were collected by filtering 5 ml of coculture through an 0.22-μm polyether sulfone filter, and the filters were flash frozen in liquid nitrogen and stored at −80°C. RNA was extracted from the filters using the MirVana RNA extraction kit (Thermo Fisher Scientific, Waltham, MA). Residual DNA was removed using the Turbo DNA-free kit (Invitrogen, Carlsbad, CA). cDNA libraries were prepared using the ScriptSEQ v2 kit and barcodes (Epicentre, Madison, WI) and sequenced with the HiSeq 4000 platform (paired end [PE] 50 × 50). Quality scores for each sequence were calculated using FastQC, and low-quality sequences were removed from the raw sequencing data using Trimmomatic (sliding window trimming with average quality score lower than 20 across 4 bases removed). Reads were mapped to the DSS-3 genome using Bowtie 2 and the count intervals tool in Galaxy. Genes with statistically different relative abundances were identified with DESeq using the DEApp deSEQ2 (59).

### Data availability.

Detailed parameters for the workflow are available in Text S1. The transcriptome data sets generated in this study can be found in the NCBI Short Read Archive (SRA) database under BioProject ID PRJNA645140 with BioSample accession numbers SAMN15493548 and SAMN15493549.
