# Supplementary figures and images for: A Model Roseobacter, Ruegeria pomeroyi DSS-3, Employs a Diffusible Killing Mechanism To Eliminate Competitors
Source: mSystems. 2020 Aug 11;5(4):e00443-20. doi: 10.1128/mSystems.00443-20 (PMC7426152; doi:10.1128/mSystems.00443-20)

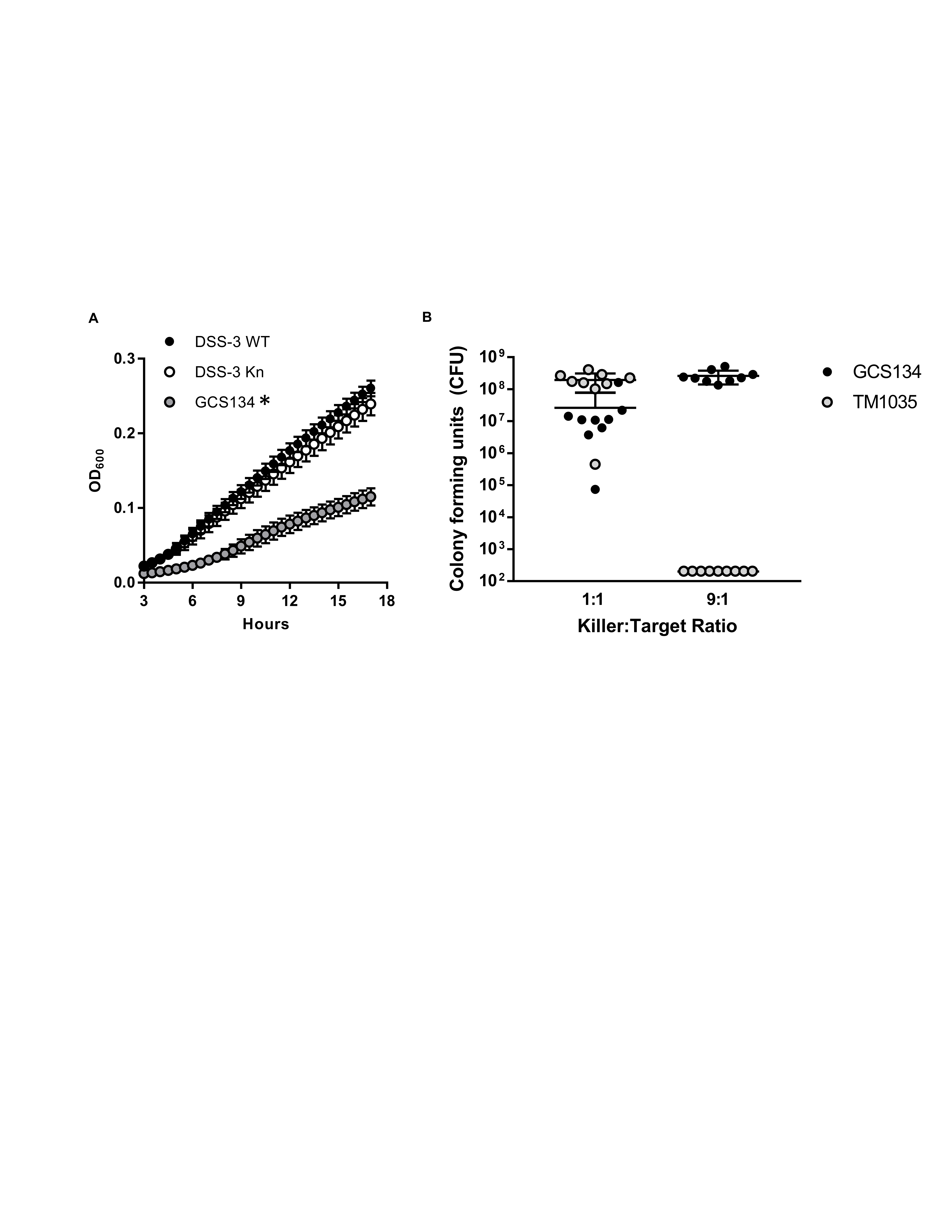

Supplement: FIG S1 [file mSystems.00443-20-sf001.tif]
