# Supplementary material for: A Model Roseobacter, Ruegeria pomeroyi DSS-3, Employs a Diffusible Killing Mechanism To Eliminate Competitors
Source: mSystems. 2020 Aug 11;5(4):e00443-20. doi: 10.1128/mSystems.00443-20 (PMC7426152; doi:10.1128/mSystems.00443-20)
Supplement: TABLE S1 [file mSystems.00443-20-st001.docx]

**Supplemental Table S1.** Homologs of DSS-3 protein products encoded in SPOA0341, 0342, and 0343

| **Species** | **Family** | **Percent Identity to DSS-3 protein^a^**  **SPOA0341 SPOA0342 SPOA0343** |
| --- | --- | --- |
| *Ruegeria sp. EL01* | Rhodobacterales | 76% 72% 70% |
| *Shimia marina* | Rhodobacterales | 69% 68% 74% |
| *Notoacmeibacter marinus* | Rhizobiales | 70% 62% 63% |
| *Halomonas sp. 54_146* | Oceanospirillales | 66% 56% 67% |
| *Rhodovulum imhoffii* | Rhodobacterales | 63% 54% 58% |
| *Azospirillum brasilense* | Rhodospirillales | 53%^b^ 47% 41% |
| *Serratia marcescens* | Enterobacterales | 56% 41% 47% |
| *Serratia plymuthica* | Enterobacterales | 40% 45% |
| *Xenorhabdus nematophila* | Enterobacterales | 55% 38% |
| *Protorhabdus asymbiotica* | Enterobacterales | 55% 38% 46% |
| *Chania multitudinisentens* | Enterobacterales | 55% 41% 48% |
| *Yersinia intermedia* | Enterobacterales | 53% 40% |
| *Erwinia persicina* | Enterobacterales | 57% 38% 46% |
| *Pseudomonas furukawaii* | Pseudomonadales | 51% 49% |
| *Vibrio jasicida* | Vibrionales | 56% 38% 46% |
| *Pantoea latae* | Enterobacterales | 54% 40% 46% |
| *Klebsiella michiganensis* | Enterobacterales | 55% 39% 46% |
| *Enterobacter bugandensis* | Enterobacterales | 55% 38% |
| *Buttiauxella warmboldiae* | Enterbacterales | 54% 37% 43% |
| *Gilliamella apicola* | Orbales | 52% 35% 40% |
| *Frischella perrara* | Orbales | 50% 37% 43% |
| *Moritella sp. PE36* | Alteromonadales | 31% 33% |
| *Glycomyces dulcitolivorans* | Glycomycetales | 59% 41% 55% |
| *Glycomyces arizonensis* | Glycomycetales | 56% 33% 45% |
| *Glycomyces sp. YIM 121974* | Glycomycetales | 57% 40% 62% |
| *Alcaligenes aquatilis* | Burkholderiales | 56% 36% 43% |
| *Streptomyces globosus* | Streptomycetales | 58% 37% 49% |
| *Streptomyces antibioticus* | Streptomycetales | 60% 33% 53% |
| *Actinokineospora inagensis* | Pseudonocardiales | 57% 31% 55% |
| *Actinokineospora terrae* | Pseudonocardiales | 57% 33% 52% |
| *Actinokineospora cianjurensis* | Pseudonocardiales | 57% 34% 51% |
| *Actinomyces ruminicola* | Streptomycetales | 51% 31% 49% |
| *Streptomyces catenulae* | Streptomycetales | 54% 31% 49% |
| *Streptomyces roseus* | Streptomycetales | 57% 37% 58% |
| *Sanguibacter sp. Leaf3* | Micrococcales | 51% 31% 53% |

^a^ Percent identity cutoff was >30% with Blastx using nr database.

^b^ SPOA0341 for *Azospirillum brasilense* is for *Azospirillum brasilense* Sp245
