# Supplementary material for: A Model Roseobacter, Ruegeria pomeroyi DSS-3, Employs a Diffusible Killing Mechanism To Eliminate Competitors
Source: mSystems. 2020 Aug 11;5(4):e00443-20. doi: 10.1128/mSystems.00443-20 (PMC7426152; doi:10.1128/mSystems.00443-20)
Supplement: TABLE S2 [file mSystems.00443-20-st002.pdf]

Gene	DvT 1	DvT 2	DvT 3	GCS64vT	GCS64vT	GCS64vT
CDS;ID=637287494;locus_tag=SPO0001;product=glucose-inhibited division protein A	33	42	23	36	39	37
CDS;ID=637287495;locus_tag=SPO0002;product=glucose-inhibited division protein B	9	7	9	11	16	16
CDS;ID=637287496;locus_tag=SPO0003;product=chromosome partitioning protein ParA	69	60	40	46	68	47
CDS;ID=637287497;locus_tag=SPO0004;product=chromosome partitioning protein parB	70	78	71	44	49	65
CDS;ID=637287498;locus_tag=SPO0005;product=hypothetical protein	15	15	16	15	10	8
CDS;ID=637287499;locus_tag=SPO0006;product=coproporphyrinogen III oxidase	10	37	13	19	18	20
CDS;ID=637287500;locus_tag=SPO0007;product=putative deoxyribonucleotide triphosphat	45	44	28	41	57	53
CDS;ID=637287501;locus_tag=SPO0008;product=ribonuclease PH	89	88	67	108	145	97
CDS;ID=637287502;locus_tag=SPO0009;product=heat-inducible transcription repressor	516	422	453	442	522	621
CDS;ID=637287503;locus_tag=SPO0010;product=co-chaperone GrpE	62	55	36	44	66	82
CDS;ID=637287504;locus_tag=SPO0011;product=DNA mismatch repair protein	41	55	36	68	58	53
CDS;ID=637287505;locus_tag=SPO0012;product=phosphate acetyltransferase	146	119	97	158	193	161
CDS;ID=637287506;locus_tag=SPO0013;product=ribokinase	18	10	17	15	16	22
CDS;ID=637287507;locus_tag=SPO0014;product=methionine-S-sulfoxide reductase	6	7	10	6	7	6
CDS;ID=637287508;locus_tag=SPO0015;product=hypothetical protein	24	28	24	29	18	25
CDS;ID=637287509;locus_tag=SPO0016;product=sterol carrier family protein	28	16	21	20	26	18
CDS;ID=637287510;locus_tag=SPO0017;product=hypothetical protein	5	3	16	9	11	10
CDS;ID=637287511;locus_tag=SPO0018;product=argininosuccinate synthase	77	47	63	75	85	64
CDS;ID=637287512;locus_tag=SPO0019;product=hypothetical protein	60	80	47	53	64	74
CDS;ID=637287513;locus_tag=SPO0020;product=threonine dehydratase	72	55	73	40	49	67
CDS;ID=637287514;locus_tag=SPO0021;product=Hpt domain protein	6	8	9	5	13	8
CDS;ID=637287515;locus_tag=SPO0022;product=response regulator	22	14	14	9	15	14
"CDS;ID=637287516;locus_tag=SPO0023;product=hydrolase, NUDIX family"	14	4	5	4	9	15
"CDS;ID=637287517;locus_tag=SPO0024;product=chaperonin, 33 kDa"	58	55	50	72	63	90
"CDS;ID=637287518;locus_tag=SPO0025;product=hydrolase, NUDIX family"	17	3	6	5	14	11
CDS;ID=637287519;locus_tag=SPO0026;product=polyA polymerase family protein	17	21	13	26	19	26
"CDS;ID=637287520;locus_tag=SPO0027;product=ABC transporter, transmembrane ATP-binc	98	69	58	51	70	93
"CDS;ID=637287521;locus_tag=SPO0028;product=ABC transporter, transmembrane ATP-binc	86	60	59	69	78	101
CDS;ID=637287522;locus_tag=SPO0029;product=23S rRNA (Uracil-5-)-methyltransferase r	39	21	36	45	30	35
CDS;ID=637287523;locus_tag=SPO0030;product=voltage-gated sodium channel	39	42	16	46	29	32
CDS;ID=637287524;locus_tag=SPO0031;product=ErfK/YbiS/YcfS/YnhG family protein	100	71	88	88	106	83
CDS;ID=637287525;locus_tag=SPO0032;product=hypothetical protein	28	21	24	15	24	21
"CDS;ID=637287526;locus_tag=SPO0033;product=transcriptional regulator, LysR family'	7	8	3	3	5	2
CDS;ID=637287527;locus_tag=SPO0034;product=hypothetical protein	33	26	13	31	20	12
CDS;ID=637287528;locus_tag=SPO0035;product=core-2/I-branching enzyme family protein	46	37	31	28	51	32
CDS;ID=637287529;locus_tag=SPO0036;product=hypothetical protein	66	47	36	45	39	52
CDS;ID=637287530;locus_tag=SPO0037;product=hypothetical protein	16	9	16	13	11	17
CDS;ID=637287531;locus_tag=SPO0038;product=3-deoxy-manno-octulosonate cytidylyltrar	17	13	18	19	30	13
"CDS;ID=637287532;locus_tag=SPO0039;product=3'(2'),5'-bisphosphate nucleotidase"	35	28	15	22	22	32
"CDS;ID=637287533;locus_tag=SPO0040;product=ABC transporter, permease protein"	36	34	18	15	40	20
CDS;ID=637287534;locus_tag=SPO0041;product=hypothetical protein	12	7	8	7	12	3
"CDS;ID=637287535;locus_tag=SPO0042;product=alkylated DNA repair protein, putative'	8	6	2	5	6	8

CDS;ID=637287536;locus_tag=SPO0043;product=chaperone protein DnaK	1036	952	857	987	1530	1107
CDS;ID=637287537;locus_tag=SPO0044;product=chaperone protein DnaJ	83	78	77	71	102	91
RNA;ID=641222523;locus_tag=SPO_R0063;product=TPP	341	245	155	207	81	298
CDS;ID=637287538;locus_tag=SPO0045;product=phosphomethylpyrimidine kinase	13	7	9	7	16	22
"CDS;ID=637287539;locus_tag=SPO0046;product=thiamine biosynthesis oxidoreductase Th	6	4	3	6	5	6
CDS;ID=637287540;locus_tag=SPO0046.1;product=thiamine biosynthesis protein ThiS	0	0	0	1	4	4
CDS;ID=637287541;locus_tag=SPO0047;product=thiazole synthase	5	6	5	4	9	9
CDS;ID=637287542;locus_tag=SPO0048;product=thiamine-phosphate pyrophosphorylase	11	13	8	4	15	9
CDS;ID=637287543;locus_tag=SPO0049;product=thiamine biosynthesis protein ThiF	4	11	3	2	3	2
"CDS;ID=637287544;locus_tag=SPO0050;product=ABC transporter, periplasmic substrate-	7	8	12	6	12	5
CDS;ID=637287545;locus_tag=SPO0051;product=TENA/THI-4 family protein	7	25	6	11	13	19
"CDS;ID=637287546;locus_tag=SPO0052;product=ABC transporter, ATP-binding protein"	2	1	5	3	4	3
"CDS;ID=637287547;locus_tag=SPO0053;product=ABC transporter, permease protein"	1	1	2	0	0	0
CDS;ID=637287548;locus_tag=SPO0054;product=DNA repair protein RadC	2	2	4	6	7	4
CDS;ID=637287549;locus_tag=SPO0055;product=OmpA domain protein	49	39	38	47	42	47
CDS;ID=637287550;locus_tag=SPO0056;product=hypothetical protein	17	14	13	14	11	11
CDS;ID=637287551;locus_tag=SPO0057;product=translocase	371	286	338	395	438	413
CDS;ID=637287552;locus_tag=SPO0058;product=PPIC-type PPIASE domain protein	138	102	115	103	155	131
CDS;ID=637287553;locus_tag=SPO0059;product=bifunctional ornithine acetyltransferase	127	143	97	123	120	141
CDS;ID=637287554;locus_tag=SPO0060;product=mutator mutT protein	31	60	33	33	37	26
CDS;ID=637287555;locus_tag=SPO0061;product=translation initiation factor IF-2	473	403	361	456	547	539
CDS;ID=637287556;locus_tag=SPO0062;product=hypothetical protein	329	252	212	250	359	330
CDS;ID=637287557;locus_tag=SPO0063;product=transcription elongation factor NusA	359	302	300	436	509	493
CDS;ID=637287558;locus_tag=SPO0064;product=hypothetical protein	209	167	196	230	254	259
"CDS;ID=637287559;locus_tag=SPO0065;product=peptide/opine/nickel uptake family ABC	46	34	33	33	21	32
CDS;ID=637287560;locus_tag=SPO0066;product=proline iminopeptidase	57	71	60	33	41	72
CDS;ID=637287561;locus_tag=SPO0067;product=3-demethylubiquinone-9 3-methyltransfera	16	12	28	22	27	24
"CDS;ID=637287562;locus_tag=SPO0068;product=transcriptional regulator, putative"	55	55	47	37	30	58
"CDS;ID=637287563;locus_tag=SPO0069;product=hydrolase, carbon-nitrogen family"	73	71	61	55	40	59
CDS;ID=637287564;locus_tag=SPO0070;product=glutaredoxin	17	16	16	13	27	12
"CDS;ID=637287565;locus_tag=SPO0071;product=competence protein F, putative"	0	1	0	0	0	0
CDS;ID=637287566;locus_tag=SPO0072;product=hypothetical protein	14	16	17	26	23	22
CDS;ID=637287567;locus_tag=SPO0073;product=ferrochelatase	50	31	46	67	43	58
CDS;ID=637287568;locus_tag=SPO0074;product=hypothetical protein	267	178	144	166	153	169
CDS;ID=637287569;locus_tag=SPO0075;product=ErfK/YbiS/YcfS/YnhG family protein/Tat d	42	30	46	45	38	54
"CDS;ID=637287570;locus_tag=SPO0076;product=lipoprotein, putative"	59	47	64	74	48	71
CDS;ID=637287571;locus_tag=SPO0077;product=PTS IIA-like nitrogen-regulatory protein	13	12	6	16	26	24
"CDS;ID=637287572;locus_tag=SPO0078;product=ribosomal subunit interface protein, pu	313	262	249	305	276	323
"CDS;ID=637287573;locus_tag=SPO0079;product=ABC transporter, ATP-binding protein"	33	27	22	27	31	36
CDS;ID=637287574;locus_tag=SPO0080;product=hypothetical protein	30	23	25	31	46	29
CDS;ID=637287575;locus_tag=SPO0081;product=hypothetical protein	63	56	53	63	66	45
CDS;ID=637287576;locus_tag=SPO0082;product=arabinose 5-phosphate isomerase	39	40	26	33	46	43
"CDS;ID=637287577;locus_tag=SPO0083;product=exonuclease, putative"	75	83	51	75	71	75

CDS;ID=637287578;locus_tag=SPO0084;product=betaine aldehyde dehydrogenase	23	20	16	16	20	20
CDS;ID=637287579;locus_tag=SPO0085;product=hypothetical protein	6	2	3	5	4	5
"CDS;ID=637287580;locus_tag=SPO0086;product=lipoprotein, putative"	16	12	11	35	23	20
CDS;ID=637287581;locus_tag=SPO0087;product=hypothetical protein	7	5	6	19	19	13
CDS;ID=637287582;locus_tag=SPO0088;product=hypothetical protein	12	5	6	8	7	11
CDS;ID=637287583;locus_tag=SPO0089;product=hypothetical protein	4	8	8	5	9	9
CDS;ID=637287584;locus_tag=SPO0090;product=hypothetical protein	13	10	2	16	9	12
CDS;ID=637287585;locus_tag=SPO0091;product=rhodanese-like domain protein	37	27	18	43	56	33
"CDS;ID=637287586;locus_tag=SPO0092;product=heat shock protein, Hsp70 family"	11	7	5	17	13	8
CDS;ID=637287587;locus_tag=SPO0093;product=pyrazinamidase/nicotinamidase	7	8	5	2	4	13
CDS;ID=637287588;locus_tag=SPO0094;product=sensory box sensor histidine kinase/resp	24	12	11	13	7	22
CDS;ID=637287589;locus_tag=SPO0095;product=nicotinate phosphoribosyltransferase	129	143	121	115	176	171
CDS;ID=637287590;locus_tag=SPO0096;product=hypothetical protein	5	6	5	6	11	5
CDS;ID=637287591;locus_tag=SPO0097;product=aldehyde dehydrogenase family protein	21	14	7	7	22	10
"CDS;ID=637287592;locus_tag=SPO0098;product=peptide/opine/nickel uptake family ABC	19	25	24	7	20	33
"CDS;ID=637287593;locus_tag=SPO0099;product=peptide/opine/nickel uptake family ABC	5	4	6	5	4	4
"CDS;ID=637287594;locus_tag=SPO0100;product=peptide/opine/nickel uptake family ABC	10	10	5	10	5	4
"CDS;ID=637287595;locus_tag=SPO0101;product=peptide/opine/nickel uptake family ABC	54	54	43	34	60	48
CDS;ID=637287596;locus_tag=SPO0102;product=3-methyl-2-oxobutanoate hydroxymethyltra	22	23	17	21	23	33
CDS;ID=637287597;locus_tag=SPO0103;product=pantoate--beta-alanine ligase	16	12	15	13	19	32
CDS;ID=637287598;locus_tag=SPO0104;product=glycerol kinase	94	65	54	45	53	89
CDS;ID=637287599;locus_tag=SPO0105;product=hypothetical protein	40	27	31	18	19	34
CDS;ID=637287600;locus_tag=SPO0106;product=hypothetical protein	21	29	19	18	25	23
CDS;ID=637287601;locus_tag=SPO0107;product=ATP-dependent DNA helicase RecQ	190	201	139	143	150	189
"CDS;ID=637287602;locus_tag=SPO0108;product=transposase, truncation"	13	21	17	15	21	24
CDS;ID=637287603;locus_tag=SPO0109;product=hypothetical protein	64	77	60	74	75	77
"CDS;ID=637287604;locus_tag=SPO0110;product=ABC transporter, ATP binding/permease p	186	145	144	172	227	227
CDS;ID=637287605;locus_tag=SPO0111;product=hypothetical protein	154	120	133	181	253	237
CDS;ID=637287606;locus_tag=SPO0112;product=poly(3-hydroxyalkanoate) polymerase fami	183	172	161	241	257	233
CDS;ID=637287607;locus_tag=SPO0113;product=enoyl-(acyl carrier protein) reductase	85	65	72	47	90	89
CDS;ID=637287608;locus_tag=SPO0114;product=hypothetical protein	42	40	36	32	28	37
CDS;ID=637287609;locus_tag=SPO0115;product=phosphate acetyltransferase	60	56	48	32	91	56
CDS;ID=637287610;locus_tag=SPO0116;product=acetate kinase	33	23	28	29	45	40
CDS;ID=640735048;locus_tag=SPO0117	7	1	1	1	2	6
CDS;ID=637287611;locus_tag=SPO0118;product=hypothetical protein	20	43	22	28	25	35
"CDS;ID=637287612;locus_tag=SPO0119;product=transcriptional regulator, AsnC family"	3	2	2	4	2	5
CDS;ID=637287613;locus_tag=SPO0120;product=hypothetical protein	8	3	2	2	3	2
CDS;ID=640735049;locus_tag=SPO0121	8	22	10	12	20	16
CDS;ID=637287614;locus_tag=SPO0122;product=YGGT family protein	22	20	25	24	36	42
CDS;ID=637287615;locus_tag=SPO0123;product=hypothetical protein	78	76	61	70	88	56
CDS;ID=637287616;locus_tag=SPO0124;product=penicillin-insensitive murein endopeptic	54	54	37	43	93	48
CDS;ID=637287617;locus_tag=SPO0125;product=hypothetical protein	18	9	11	7	11	13
CDS;ID=637287618;locus_tag=SPO0126;product=hypothetical protein	11	23	12	11	14	15

CDS;ID=637287619;locus_tag=SPO0127;product=hypothetical protein	32	25	32	41	35	28
CDS;ID=637287620;locus_tag=SPO0128;product=3-ketoacyl-(acyl-carrier-protein) reduct	26	46	18	21	17	27
"CDS;ID=637287621;locus_tag=SPO0129;product=peptidase, T4 family"	14	18	17	13	10	26
CDS;ID=637287622;locus_tag=SPO0130;product=cytochrome c family protein	146	123	115	103	162	121
CDS;ID=637287623;locus_tag=SPO0131;product=isopentyl-diphosphate delta-isomerase	19	16	10	16	7	5
CDS;ID=637287624;locus_tag=SPO0132;product=sensor histidine kinase/response regulat	5	10	5	11	13	10
CDS;ID=637287625;locus_tag=SPO0133;product=PAN domain protein	53	46	48	34	46	61
CDS;ID=637287626;locus_tag=SPO0134;product=hypothetical protein	7	5	5	7	10	9
CDS;ID=637287627;locus_tag=SPO0135;product=penicillin-binding protein 1C	11	6	1	4	3	4
CDS;ID=637287628;locus_tag=SPO0136;product=hypothetical protein	35	59	26	29	25	31
CDS;ID=637287629;locus_tag=SPO0137;product=hypothetical protein	43	40	29	16	27	32
CDS;ID=637287630;locus_tag=SPO0138;product=hypothetical protein	33	21	20	15	22	19
"CDS;ID=637287631;locus_tag=SPO0139;product=ATPase, MoxR family"	30	30	26	24	30	24
CDS;ID=637287632;locus_tag=SPO0140;product=hypothetical protein	18	20	23	20	24	33
"CDS;ID=637287633;locus_tag=SPO0141;product=DNA binding protein, putative"	25	16	9	16	32	14
CDS;ID=637287634;locus_tag=SPO0142;product=beta-ketothiolase	59	40	44	49	69	54
CDS;ID=637287635;locus_tag=SPO0143;product=iron-sulfur cluster-binding protein	10	6	4	4	4	5
CDS;ID=637287636;locus_tag=SPO0144;product=2-polyprenylphenol 6-hydroxylase	40	22	21	19	20	25
CDS;ID=637287637;locus_tag=SPO0145;product=ubiquinone/menaquinone biosynthesis meth	41	25	33	37	32	37
CDS;ID=637287638;locus_tag=SPO0146;product=formamidopyrimidine-DNA glycosylase	5	21	12	13	18	12
CDS;ID=637287639;locus_tag=SPO0147;product=enoyl-CoA hydratase	164	168	154	168	118	167
CDS;ID=637287640;locus_tag=SPO0148;product=ribosomal protein S20	94	86	80	117	95	107
CDS;ID=637287641;locus_tag=SPO0149;product=chromosomal replication initiation prote	233	201	196	252	256	250
CDS;ID=637287642;locus_tag=SPO0150;product=DNA polymerase III subunit beta	283	233	228	216	211	260
CDS;ID=637287643;locus_tag=SPO0151;product=recombination protein F	44	36	31	56	39	36
"CDS;ID=637287644;locus_tag=SPO0152;product=transporter, LysE family"	13	15	10	25	6	19
"CDS;ID=637287645;locus_tag=SPO0153;product=transcriptional regulator, TetR family'	6	2	10	9	7	5
"CDS;ID=637287646;locus_tag=SPO0154;product=NAD(P)H dehydrogenase, quinone family"	1	6	6	4	6	6
CDS;ID=637287647;locus_tag=SPO0155;product=DNA gyrase subunit B	263	193	171	183	248	253
CDS;ID=637287648;locus_tag=SPO0156;product=transcriptional regulator RtcR	19	14	17	10	15	18
CDS;ID=637287649;locus_tag=SPO0157;product=TROVE domain protein	320	309	271	284	177	289
tRNA;ID=640698507;locus_tag=SPO_tRNA-Gln-1	3	13	2	4	8	4
tRNA;ID=640698508;locus_tag=SPO_tRNA-Pro-2	6	14	8	7	10	3
CDS;ID=637287652;locus_tag=SPO0160;product=hypothetical protein	26	31	28	25	33	32
"CDS;ID=637287653;locus_tag=SPO0161;product=DNA-binding response regulator, LuxR fa	120	97	119	130	145	211
CDS;ID=637287654;locus_tag=SPO0162;product=hypothetical protein	160	166	142	117	75	99
CDS;ID=637287655;locus_tag=SPO0163;product=sensory box histidine kinase/response re	46	55	42	41	50	40
"CDS;ID=637287656;locus_tag=SPO0164;product=oxidoreductase, FMN-binding/pyridine nu	10	6	12	6	9	11
"CDS;ID=637287657;locus_tag=SPO0165;product=pyrroline-5-carboxylate reductase, puta	1	2	4	2	0	4
"CDS;ID=637287658;locus_tag=SPO0166;product=N-methylproline demethylase, putative"	29	25	24	25	29	15
CDS;ID=637287659;locus_tag=SPO0167;product=trimethylamine methyltransferase family	17	19	25	7	26	15
"CDS;ID=637287660;locus_tag=SPO0168;product=transcriptional regulator, TetR family'	25	12	13	15	9	14
"CDS;ID=637287661;locus_tag=SPO0169;product=transglycosylase, Slt family"	1	0	0	0	1	1

CDS;ID=637287662;locus_tag=SPO0170;product=flagellar biosynthesis protein FlhA	16	10	4	8	14	22
CDS;ID=637287663;locus_tag=SPO0171;product=flagellar biosynthetic protein FliR	11	9	8	9	12	7
CDS;ID=637287664;locus_tag=SPO0172;product=flagellar biosynthetic protein FlhB	5	1	4	6	3	0
CDS;ID=637287665;locus_tag=SPO0173;product=hypothetical protein	3	3	1	0	0	0
CDS;ID=637287666;locus_tag=SPO0174;product=hypothetical protein	1	1	3	0	2	3
CDS;ID=637287667;locus_tag=SPO0175;product=flagellar L-ring protein precursor	2	2	1	0	5	1
CDS;ID=637287668;locus_tag=SPO0176;product=flagellar basal-body P-ring formation pı	1	0	2	0	0	1
CDS;ID=637287669;locus_tag=SPO0177;product=flagellar basal-body rod protein FlgG	4	2	2	5	0	6
CDS;ID=637287670;locus_tag=SPO0178;product=flagellar basal-body rod protein FlgF	5	2	2	2	2	3
CDS;ID=637287671;locus_tag=SPO0179;product=flagellar biosynthetic protein FliQ	0	0	0	0	2	0
"CDS;ID=637287672;locus_tag=SPO0180;product=flagellar hook-basal body complex prote	0	0	0	0	2	0
CDS;ID=637287673;locus_tag=SPO0181;product=flagellar basal-body rod protein FlgC	0	3	0	0	1	1
CDS;ID=637287674;locus_tag=SPO0182;product=flagellar basal-body rod protein; FlgB	1	3	0	3	2	2
"CDS;ID=637287675;locus_tag=SPO0183;product=H+-transporting two-sector ATPase, flag	12	6	8	4	3	9
CDS;ID=637287676;locus_tag=SPO0184;product=hypothetical protein	59	51	36	49	33	48
CDS;ID=637287677;locus_tag=SPO0185;product=hypothetical protein	79	83	50	49	45	60
CDS;ID=637287678;locus_tag=SPO0186;product=bordetella uptake gene family protein	306	301	224	262	93	186
CDS;ID=637287679;locus_tag=SPO0187;product=DNA-binding response regulator	15	31	22	7	8	8
CDS;ID=637287680;locus_tag=SPO0188;product=sensor histidine kinase	27	34	24	33	17	31
CDS;ID=637287681;locus_tag=SPO0189;product=hypothetical protein	13	7	7	7	9	11
"CDS;ID=637287682;locus_tag=SPO0190;product=oxidoreductase, GMC family"	29	21	13	9	11	22
CDS;ID=637287683;locus_tag=SPO0191;product=chemotaxis protein MotB	20	20	12	17	23	26
"CDS;ID=637287684;locus_tag=SPO0192;product=flagellar hook protein FlgE, putative"	5	6	8	4	3	8
CDS;ID=637287685;locus_tag=SPO0193;product=flagellar hook-associated protein	5	12	21	11	9	15
CDS;ID=637287686;locus_tag=SPO0194;product=flagellar hook-associated protein FlgL f	33	18	17	24	22	14
CDS;ID=637287687;locus_tag=SPO0195;product=flagellar P-ring protein precursor	7	4	6	8	8	8
CDS;ID=637287688;locus_tag=SPO0196;product=flagellar biosynthetic protein FliP	2	3	3	3	1	3
CDS;ID=637287689;locus_tag=SPO0197;product=flagellar motor switch protein FliN	1	0	2	2	0	0
"CDS;ID=637287690;locus_tag=SPO0198;product=ABC transporter, ATP-binding protein, f	2	0	3	3	2	2
CDS;ID=637287691;locus_tag=SPO0199;product=flagellar M-ring protein FliF	1	4	2	2	5	3
CDS;ID=637287692;locus_tag=SPO0200;product=flagellar basal body-associated protein	0	1	0	2	2	1
CDS;ID=637287693;locus_tag=SPO0201;product=hypothetical protein	3	1	3	2	1	0
CDS;ID=637287694;locus_tag=SPO0202;product=hypothetical protein	0	1	1	3	0	1
CDS;ID=637287695;locus_tag=SPO0203;product=flagellar motor protein	4	2	2	5	2	3
CDS;ID=637287696;locus_tag=SPO0204;product=hypothetical protein	30	32	17	24	26	29
CDS;ID=637287697;locus_tag=SPO0205;product=MmgE/PrpD family protein	8	10	1	12	5	9
"CDS;ID=637287698;locus_tag=SPO0206;product=transcriptional regulator, LysR family'	3	7	10	9	7	14
CDS;ID=637287699;locus_tag=SPO0207;product=aminomethyl transferase family protein	15	17	4	9	14	11
tRNA;ID=640698509;locus_tag=SPO_tRNA-Pro-3	81	45	36	42	18	27
CDS;ID=637287701;locus_tag=SPO0209;product=Na/Pi-cotransporter family protein	18	9	7	9	8	7
CDS;ID=637287702;locus_tag=SPO0210;product=3-isopropylmalate dehydrogenase	36	42	33	59	69	59
"CDS;ID=637287703;locus_tag=SPO0211;product=transcriptional regulator, LysR family'	5	1	0	1	1	4
CDS;ID=637287704;locus_tag=SPO0212;product=hypothetical protein	38	23	31	45	40	49

CDS;ID=637287705;locus_tag=SPO0213;product=hypothetical protein	17	17	15	29	28	12
CDS;ID=637287706;locus_tag=SPO0214;product=hypothetical protein	29	25	25	25	43	59
CDS;ID=637287707;locus_tag=SPO0215;product=isopropylmalate isomerase small subunit	34	18	42	36	46	33
CDS;ID=637287708;locus_tag=SPO0216;product=isopropylmalate isomerase large subunit	163	199	173	194	252	210
CDS;ID=637287709;locus_tag=SPO0217;product=hypothetical protein	146	165	169	237	326	163
CDS;ID=637287710;locus_tag=SPO0218;product=mechanosensitive ion channel family prot	24	15	6	8	13	10
CDS;ID=637287711;locus_tag=SPO0219;product=iojap-related protein	151	108	90	180	151	156
CDS;ID=637287712;locus_tag=SPO0220;product=hypothetical protein	16	35	31	25	48	53
CDS;ID=637287713;locus_tag=SPO0221;product=hypothetical protein	12	16	9	13	26	12
CDS;ID=637287714;locus_tag=SPO0222;product=alanine dehydrogenase	31	18	33	54	62	53
"CDS;ID=637287715;locus_tag=SPO0223;product=transcriptional regulator, AsnC family'	1	4	6	5	5	2
"CDS;ID=637287716;locus_tag=SPO0224;product=polyphosphate kinase, putative"	56	24	52	45	87	49
"CDS;ID=637287717;locus_tag=SPO0225;product=transcriptional regulator, putative"	3	5	4	4	6	2
CDS;ID=637287718;locus_tag=SPO0226;product=cytochrome P450 family protein	26	22	13	25	25	16
"CDS;ID=637287719;locus_tag=SPO0227;product=PaxA, putative"	808	734	679	776	1001	938
CDS;ID=637287720;locus_tag=SPO0228;product=hypothetical protein	6	4	6	4	8	7
CDS;ID=637287721;locus_tag=SPO0229;product=30S ribosomal protein S21	5447	5052	5276	8313	3596	6593
CDS;ID=637287722;locus_tag=SPO0230;product=hypothetical protein	69	57	27	43	46	81
"CDS;ID=637287723;locus_tag=SPO0231;product=alcohol dehydrogenase, zinc-containing'	90	64	62	72	55	107
"CDS;ID=637287724;locus_tag=SPO0232;product=quinone oxidoreductase, putative"	47	48	32	31	45	44
"CDS;ID=637287725;locus_tag=SPO0233;product=transcriptional regulator, AsnC family'	24	23	10	25	9	30
CDS;ID=637287726;locus_tag=SPO0234;product=hypothetical protein	82	93	58	24	30	32
CDS;ID=637287727;locus_tag=SPO0235;product=aldehyde dehydrogenase family protein	142	183	104	41	40	66
"CDS;ID=637287728;locus_tag=SPO0236;product=glycerophosphoryl diester phosphodieste	9	9	6	2	8	12
"CDS;ID=637287729;locus_tag=SPO0237;product=SN-glycerol-3-phosphate ABC transporter	18	19	19	29	28	36
"CDS;ID=637287730;locus_tag=SPO0238;product=SN-glycerol-3-phosphate ABC transporter	15	11	31	29	50	40
"CDS;ID=637287731;locus_tag=SPO0239;product=SN-glycerol-3-phosphate ABC transporter	7	9	5	4	14	14
"CDS;ID=637287732;locus_tag=SPO0240;product=SN-glycerol-3-phophate ABC transporter,	30	29	54	45	57	69
"CDS;ID=637287733;locus_tag=SPO0241;product=transcriptional regulator, LysR family'	6	2	2	6	10	7
tRNA;ID=640698511;locus_tag=SPO_tRNA-Ile-1	189	114	86	116	100	132
tRNA;ID=640698512;locus_tag=SPO_tRNA-Ala-1	185	65	43	44	40	53
rRNA;ID=640698514;locus_tag=SPO_Sp5SD;product=5S	11146	7956	4173	4292	2432	4294
CDS;ID=637287739;locus_tag=SPO0247;product=1-deoxy-D-xylulose-5-phosphate synthase	35	38	27	35	43	36
CDS;ID=637287740;locus_tag=SPO0248;product=farnesyl diphosphate synthase	23	16	23	20	32	26
"CDS;ID=637287741;locus_tag=SPO0249;product=exodeoxyribonuclease VII, small subunit	5	4	3	10	11	2
CDS;ID=637287742;locus_tag=SPO0250;product=histone deacetylase family protein	5	15	9	4	13	12
CDS;ID=637287743;locus_tag=SPO0251;product=DNA-binding response regulator PetR	17	12	10	14	23	22
CDS;ID=637287744;locus_tag=SPO0252;product=transcriptional regulator PetP	19	27	9	11	12	23
CDS;ID=637287745;locus_tag=SPO0253;product=branched-chain amino acid aminotransfera	155	152	125	175	239	226
CDS;ID=637287746;locus_tag=SPO0254;product=organic hydroperoxide resistance protein	80	72	82	69	79	90
"CDS;ID=637287747;locus_tag=SPO0255;product=transcriptional regulator, MarR family'	13	18	15	9	8	6
"CDS;ID=637287748;locus_tag=SPO0256;product=copper-binding protein, plastocyanin/az	2	2	0	1	2	2
CDS;ID=637287749;locus_tag=SPO0257;product=hypothetical protein	2	3	0	0	1	6

"CDS;ID=637287750;locus_tag=SPO0258;product=transcriptional regulator, AraC family'	18	11	17	15	25	28
CDS;ID=637287751;locus_tag=SPO0259;product=hypothetical protein	7	8	4	12	5	9
"CDS;ID=637287752;locus_tag=SPO0260;product=alkaline phosphatase, putative"	4	7	1	4	4	13
CDS;ID=637287753;locus_tag=SPO0261;product=hypothetical protein	12	10	14	3	6	7
CDS;ID=637287754;locus_tag=SPO0262;product=cob(I)alamin adenosyltransferase	85	90	62	70	77	89
CDS;ID=637287755;locus_tag=SPO0263;product=membrane protein	8	14	4	4	7	8
CDS;ID=637287756;locus_tag=SPO0264;product=CaiB/BaiF family protein	22	25	17	10	18	20
CDS;ID=637287757;locus_tag=SPO0265;product=auxin efflux carrier family protein	10	4	6	2	12	1
CDS;ID=637287758;locus_tag=SPO0266;product=HD domain protein	15	3	3	8	9	10
CDS;ID=637287759;locus_tag=SPO0267;product=chorismate synthase	50	43	44	59	73	47
"CDS;ID=637287760;locus_tag=SPO0268;product=riboflavin synthase, beta subunit"	90	51	47	44	22	62
RNA;ID=641222524;locus_tag=SPO_R0064;product=FMN	79	46	35	31	11	49
CDS;ID=637287761;locus_tag=SPO0269;product=negative transcriptional regulator	19	12	13	19	13	18
CDS;ID=637287762;locus_tag=SPO0270;product=glutathione S-transferase family proteir	29	27	31	26	31	44
"CDS;ID=637287763;locus_tag=SPO0271;product=ubiquinol-cytochrome c reductase, iron-	164	142	148	198	221	144
"CDS;ID=637287764;locus_tag=SPO0272;product=ubiquinol--cytochrome c reductase, cyto	150	139	137	98	165	144
"CDS;ID=637287765;locus_tag=SPO0273;product=ubiquinol--cytochrome c reductase, cyto	155	160	139	151	140	136
CDS;ID=637287766;locus_tag=SPO0274;product=hypothetical protein	1	1	1	0	1	1
"CDS;ID=637287767;locus_tag=SPO0275;product=cytochrome b, putative"	50	49	29	28	46	35
"CDS;ID=637287768;locus_tag=SPO0276;product=transcriptional regulator, LuxR family'	1	7	5	1	6	7
CDS;ID=637287769;locus_tag=SPO0277;product=MiaB-like tRNA modifying enzyme	21	16	11	23	32	29
CDS;ID=637287770;locus_tag=SPO0278;product=diaminopimelate epimerase	52	29	39	50	45	62
tRNA;ID=640698515;locus_tag=SPO_tRNA-Lys-2	37	40	24	89	37	26
"CDS;ID=637287772;locus_tag=SPO0280;product=transposase, truncation"	4	5	4	1	3	2
CDS;ID=637287773;locus_tag=SPO0281;product=hypothetical protein	24	20	13	17	22	33
CDS;ID=637287774;locus_tag=SPO0282;product=hypothetical protein	4	6	3	2	8	5
CDS;ID=637287775;locus_tag=SPO0283;product=conserved hypothetical protein TIGR0002	95	45	34	47	60	48
"CDS;ID=637287776;locus_tag=SPO0284;product=dihydroorotase, multifunctional complex	59	76	64	54	80	76
CDS;ID=637287777;locus_tag=SPO0285;product=hypothetical protein	24	23	21	21	27	13
CDS;ID=637287778;locus_tag=SPO0286;product=hypothetical protein	21	14	24	18	12	27
CDS;ID=637287779;locus_tag=SPO0287;product=aspartate carbamoyltransferase catalytic	34	33	28	39	46	48
CDS;ID=637287780;locus_tag=SPO0288;product=uracil-DNA glycosylase	57	46	55	36	40	88
CDS;ID=637287781;locus_tag=SPO0289;product=Ser/Thr protein phosphatase family prote	44	30	19	20	28	29
CDS;ID=637287782;locus_tag=SPO0290;product=transmembrane amino acid efflux protein	26	24	15	21	24	35
CDS;ID=637287783;locus_tag=SPO0291;product=molybdenum cofactor biosynthesis proteir	44	31	17	19	35	23
"CDS;ID=637287784;locus_tag=SPO0292;product=efflux transporter, RND family, MFP sub	25	13	12	13	24	31
"CDS;ID=637287785;locus_tag=SPO0293;product=transporter, AcrB/AcrD/AcrF family"	28	33	28	33	26	23
"CDS;ID=637287786;locus_tag=SPO0294;product=hydrolase, NUDIX family"	11	25	12	15	7	21
CDS;ID=637287787;locus_tag=SPO0295;product=hypothetical protein	85	75	55	67	64	70
CDS;ID=637287788;locus_tag=SPO0296;product=hypothetical protein	39	32	31	18	34	37
CDS;ID=637287789;locus_tag=SPO0297;product=Sua5/YciO/YrdC family protein	17	25	26	14	28	25
CDS;ID=637287790;locus_tag=SPO0298;product=acyl-CoA dehydrogenase family protein	75	42	62	67	71	102
CDS;ID=637287791;locus_tag=SPO0299;product=metallo-beta-lactamase family protein	44	28	34	38	26	39

CDS;ID=637287792;locus_tag=SPO0300;product=hypothetical protein	12	26	19	11	16	16
CDS;ID=637287793;locus_tag=SPO0301;product=glycerophosphoryl diester phosphodiester	28	31	20	14	24	24
CDS;ID=637287794;locus_tag=SPO0302;product=hypothetical protein	10	8	8	2	4	6
"CDS;ID=637287795;locus_tag=SPO0303;product=cytochrome c oxidase, subunit IV domair	54	57	50	57	82	44
"CDS;ID=637287796;locus_tag=SPO0304;product=lipoprotein, putative"	26	33	23	41	31	40
CDS;ID=637287797;locus_tag=SPO0305;product=AzlC family protein	12	13	16	29	12	15
CDS;ID=637287798;locus_tag=SPO0306;product=formate dehydrogenase accessory protein	58	53	47	44	52	51
CDS;ID=637287799;locus_tag=SPO0307;product=molybdopterin-guanine dinucleotide biosy	8	7	7	11	6	8
CDS;ID=637287800;locus_tag=SPO0308;product=molybdopterin-guanine dinucleotide biosy	4	3	2	5	4	0
CDS;ID=637287801;locus_tag=SPO0309;product=hypothetical protein	19	11	16	12	10	15
CDS;ID=637287802;locus_tag=SPO0310;product=molybdopterin biosynthesis protein MoeA	18	19	14	5	13	20
CDS;ID=637287803;locus_tag=SPO0311;product=hypothetical protein	50	43	30	42	41	38
CDS;ID=637287804;locus_tag=SPO0312;product=transcription elongation factor GreA	75	61	59	59	73	81
CDS;ID=637287805;locus_tag=SPO0313;product=glyoxalase family protein	0	3	3	3	2	3
CDS;ID=637287806;locus_tag=SPO0314;product=redox-sensitive transcriptional activato	2	0	1	6	1	1
CDS;ID=637287807;locus_tag=SPO0315;product=hypothetical protein	18	14	16	9	8	6
CDS;ID=637287808;locus_tag=SPO0316;product=electrotransfer ubiquinone oxidoreductas	171	132	141	146	190	134
CDS;ID=637287809;locus_tag=SPO0317;product=TPR domain protein	157	120	120	103	102	163
CDS;ID=637287810;locus_tag=SPO0318;product=4-diphosphocytidyl-2C-methyl-D-erythrito	32	10	14	8	11	13
CDS;ID=637287811;locus_tag=SPO0319;product=decaprenyl diphosphate synthase	74	59	88	78	93	70
CDS;ID=637287812;locus_tag=SPO0320;product=hypothetical protein	174	129	127	152	139	132
CDS;ID=637287813;locus_tag=SPO0321;product=hypothetical protein	18	19	9	12	10	10
CDS;ID=637287814;locus_tag=SPO0322;product=hypothetical protein	806	863	710	736	404	656
CDS;ID=637287815;locus_tag=SPO0323;product=hypothetical protein	39	55	21	28	35	27
"CDS;ID=637287816;locus_tag=SPO0324;product=transcriptional regulator, LysR family'	5	8	6	2	7	3
CDS;ID=637287817;locus_tag=SPO0325;product=acetoacetyl-CoA reductase	30	13	11	21	12	14
CDS;ID=637287818;locus_tag=SPO0326;product=acetyl-CoA acetyltransferase	66	50	60	54	111	47
CDS;ID=637287819;locus_tag=SPO0327;product=EAL domain protein	0	0	0	0	0	2
CDS;ID=637287820;locus_tag=SPO0328;product=hypothetical protein	14	13	5	9	10	13
CDS;ID=637287821;locus_tag=SPO0329;product=hypothetical protein	17	19	11	5	14	14
CDS;ID=637287822;locus_tag=SPO0330;product=cytochrome c peroxidase	17	6	17	14	19	15
"CDS;ID=637287823;locus_tag=SPO0331;product=thiol:disulfide interchange protein, pu	19	13	14	15	23	26
CDS;ID=637287824;locus_tag=SPO0332;product=argininosuccinate lyase	83	75	80	79	102	103
"CDS;ID=637287825;locus_tag=SPO0333;product=lipoprotein, putative"	12	7	5	5	15	5
CDS;ID=637287826;locus_tag=SPO0334;product=diaminopimelate decarboxylase	99	74	60	107	96	130
CDS;ID=637287827;locus_tag=SPO0335;product=hypothetical protein	61	64	51	45	59	62
CDS;ID=637287828;locus_tag=SPO0336;product=hypothetical protein	109	74	70	101	94	90
CDS;ID=637287829;locus_tag=SPO0337;product=cell division ATP-binding protein FtsE	69	65	30	50	46	74
"CDS;ID=637287830;locus_tag=SPO0338;product=cell division permease protein FtsX, pu	28	20	23	26	21	35
CDS;ID=637287831;locus_tag=SPO0339;product=acyltransferase family protein	63	51	52	43	50	46
"CDS;ID=637287832;locus_tag=SPO0340;product=2-oxoglutarate dehydrogenase, E3 compor	65	72	66	86	94	80
CDS;ID=637287833;locus_tag=SPO0341;product=hypothetical protein	18	12	9	32	31	26
CDS;ID=637287834;locus_tag=SPO0342;product=hypothetical protein	11	10	9	25	20	21

CDS;ID=637287835;locus_tag=SPO0343;product=dihydrolipoamide acetyltransferase	32	39	27	27	45	32
"CDS;ID=637287836;locus_tag=SPO0344;product=2-oxoglutarate dehydrogenase, E1 compor	102	110	91	87	138	128
CDS;ID=637287837;locus_tag=SPO0345;product=hypothetical protein	9	3	3	4	10	10
"CDS;ID=637287838;locus_tag=SPO0346;product=succinyl-CoA synthase, alpha subunit"	99	87	82	98	180	128
CDS;ID=637287839;locus_tag=SPO0347;product=succinyl-CoA synthetase subunit beta	240	171	185	291	296	320
CDS;ID=637287840;locus_tag=SPO0348;product=hypothetical protein	33	16	23	13	25	17
CDS;ID=637287841;locus_tag=SPO0349;product=malate dehydrogenase	113	93	97	77	126	109
CDS;ID=637287842;locus_tag=SPO0350;product=hypothetical protein	14	13	6	12	3	19
CDS;ID=637287843;locus_tag=SPO0351;product=hypothetical protein	0	2	0	0	0	0
"CDS;ID=637287844;locus_tag=SPO0352;product=citrate lyase, beta subunit"	24	21	23	18	16	23
CDS;ID=637287845;locus_tag=SPO0353;product=NnrU family protein	17	19	17	14	23	17
CDS;ID=637287846;locus_tag=SPO0354;product=hypothetical protein	15	10	9	8	17	14
CDS;ID=637287847;locus_tag=SPO0355;product=hypothetical protein	34	39	33	40	32	27
CDS;ID=637287848;locus_tag=SPO0356;product=hypothetical protein	14	19	11	18	12	18
CDS;ID=637287849;locus_tag=SPO0357;product=von Willebrand factor type A domain prot	4	2	4	4	2	4
"CDS;ID=637287850;locus_tag=SPO0358;product=succinate dehydrogenase, cytochrome b55	51	26	35	50	59	70
"CDS;ID=637287851;locus_tag=SPO0359;product=succinate dehydrogenase, hydrophobic me	14	25	17	11	31	22
CDS;ID=637287852;locus_tag=SPO0360;product=succinate dehydrogenase	143	107	119	198	235	160
CDS;ID=637287853;locus_tag=SPO0361;product=succinate dehydrogenase catalytic subuni	146	110	125	127	191	89
CDS;ID=637287854;locus_tag=SPO0362;product=type I secretion target repeat protein	331	302	287	298	317	302
CDS;ID=637287855;locus_tag=SPO0363;product=hypothetical protein	297	190	199	215	174	201
"CDS;ID=637287856;locus_tag=SPO0364;product=decarboxylase, pyridoxal-dependent"	19	17	9	12	8	29
CDS;ID=637287857;locus_tag=SPO0365;product=hypothetical protein	37	33	25	18	27	24
CDS;ID=637287858;locus_tag=SPO0366;product=purine nucleoside phosphorylase	49	47	41	58	31	62
"CDS;ID=637287859;locus_tag=SPO0367;product=DNA-binding protein, H-NS family"	97	61	82	89	74	83
CDS;ID=637287860;locus_tag=SPO0368;product=methylmalonyl-CoA mutase	24	21	14	20	25	19
CDS;ID=637287861;locus_tag=SPO0369;product=acyltransferase family protein	16	20	10	12	16	12
CDS;ID=637287862;locus_tag=SPO0370;product=crotonyl-CoA reductase	97	89	77	73	75	81
CDS;ID=637287863;locus_tag=SPO0371;product=autoinducer-binding transcriptional regu	10	4	7	5	12	13
CDS;ID=637287864;locus_tag=SPO0372;product=autoinducer synthesis protein	1	0	2	0	0	4
"CDS;ID=637287865;locus_tag=SPO0373;product=helicase, ATP-dependent, putative"	40	42	23	34	51	54
CDS;ID=637287866;locus_tag=SPO0374;product=hypothetical protein	15	7	18	15	12	16
CDS;ID=637287867;locus_tag=SPO0375;product=membrane protein	22	12	10	19	27	24
"CDS;ID=637287868;locus_tag=SPO0376;product=sugar ABC transporter, permease protein	19	26	14	9	17	17
"CDS;ID=637287869;locus_tag=SPO0377;product=sugar ABC transporter, permease protein	18	11	9	6	12	13
"CDS;ID=637287870;locus_tag=SPO0378;product=sugar ABC transporter, ATP-binding prot	69	43	50	45	51	54
"CDS;ID=637287871;locus_tag=SPO0379;product=sugar ABC transporter, periplasmic suga	60	61	58	46	54	61
"CDS;ID=637287872;locus_tag=SPO0380;product=ribosomal-protein-alanine acetyltransfe	2	2	1	2	1	3
"CDS;ID=637287873;locus_tag=SPO0381;product=protease, putative"	10	3	2	5	2	2
CDS;ID=637287874;locus_tag=SPO0382;product=nifU domain protein	40	32	32	44	40	54
"CDS;ID=637287875;locus_tag=SPO0383;product=acyl carrier protein phosphodiesterase,	0	0	1	1	5	5
CDS;ID=637287876;locus_tag=SPO0384;product=hypothetical protein	5	7	4	8	11	7
"CDS;ID=637287877;locus_tag=SPO0385;product=2-hydroxychromene-2-carboxylate isomera	15	14	11	11	13	17

CDS;ID=637287878;locus_tag=SPO0386;product=universal stress family protein	48	62	47	45	36	53
CDS;ID=637287879;locus_tag=SPO0387;product=hypothetical protein	25	24	18	18	12	19
CDS;ID=637287880;locus_tag=SPO0388;product=branched-chain amino acid aminotransfera	92	81	75	67	87	84
"CDS;ID=637287881;locus_tag=SPO0389;product=transcriptional regulator, AsnC family'	2	2	0	2	5	1
CDS;ID=637287882;locus_tag=SPO0390;product=glutamate/leucine/phenylalanine/valine (	55	56	56	72	79	57
"CDS;ID=637287883;locus_tag=SPO0391;product=biphenyl-2,3-diol 1,2-dioxygenase III"	30	23	23	26	19	31
CDS;ID=637287884;locus_tag=SPO0392;product=tryptophanyl-tRNA synthetase	47	49	68	74	74	71
CDS;ID=637287885;locus_tag=SPO0393;product=rhomboid family protein	24	38	37	48	36	35
CDS;ID=637287886;locus_tag=SPO0394;product=hypothetical protein	6	1	1	3	3	2
CDS;ID=637287887;locus_tag=SPO0395;product=cyclic nucleotide-binding protein	5	9	5	2	2	6
CDS;ID=637287888;locus_tag=SPO0396;product=integral membrane protein MviN	16	14	14	8	13	14
CDS;ID=637287889;locus_tag=SPO0397;product=PII uridylyl-transferase	107	74	101	96	117	113
CDS;ID=637287890;locus_tag=SPO0398;product=hypothetical protein	37	32	28	47	40	61
CDS;ID=637287891;locus_tag=SPO0399;product=tetrapyrrole methylase family protein	19	34	34	35	24	22
CDS;ID=637287892;locus_tag=SPO0400;product=hypothetical protein	6	5	8	8	3	6
CDS;ID=637287893;locus_tag=SPO0401;product=glutathione synthetase	56	59	46	42	48	60
CDS;ID=637287894;locus_tag=SPO0402;product=competence protein ComM	21	12	8	7	12	14
CDS;ID=637287895;locus_tag=SPO0403;product=hypothetical protein	9	16	10	6	10	4
CDS;ID=637287896;locus_tag=SPO0404;product=phosphoglycerate mutase family protein	5	6	3	3	2	1
CDS;ID=637287897;locus_tag=SPO0405;product=cobinamide kinase/cobinamide phosphate (	19	11	15	8	11	15
CDS;ID=637287898;locus_tag=SPO0406;product=RNA polymerase sigma factor	21	12	17	16	18	21
CDS;ID=637287899;locus_tag=SPO0407;product=hypothetical protein	14	9	2	3	5	12
CDS;ID=637287900;locus_tag=SPO0408;product=phosphopantothenoylcysteine synthase/dec	71	55	49	46	90	51
CDS;ID=637287901;locus_tag=SPO0409;product=deoxyuridine 5'-triphosphate nucleotidoh	12	8	1	9	7	3
"CDS;ID=637287902;locus_tag=SPO0410;product=molybdopterin biosynthesis protein MoeE	32	33	21	27	32	22
CDS;ID=637287903;locus_tag=SPO0411;product=hypothetical protein	43	23	15	36	32	51
CDS;ID=637287904;locus_tag=SPO0412;product=hypothetical protein	51	34	37	66	63	72
CDS;ID=637287905;locus_tag=SPO0413;product=Cys/Met metabolism PLP-dependent enzyme	9	8	6	5	3	11
"CDS;ID=637287906;locus_tag=SPO0414;product=peptidyl-dipeptidase, putative"	78	76	48	55	67	79
CDS;ID=637287907;locus_tag=SPO0415;product=D-isomer specific 2-hydroxyacid dehydro(	11	13	10	9	17	10
CDS;ID=637287908;locus_tag=SPO0416;product=rod shape-determining protein MreD	22	20	20	13	14	23
CDS;ID=637287909;locus_tag=SPO0417;product=penicillin-binding protein 2	66	57	29	39	58	43
CDS;ID=637287910;locus_tag=SPO0418;product=hypothetical protein	32	27	23	12	15	15
CDS;ID=637287911;locus_tag=SPO0419;product=rod shape-determining protein MreC	26	20	16	27	24	34
CDS;ID=637287912;locus_tag=SPO0420;product=rod shape-determining protein MreB	108	115	112	123	123	157
CDS;ID=637287913;locus_tag=SPO0421;product=hypothetical protein	14	11	12	13	17	19
CDS;ID=637287914;locus_tag=SPO0422;product=2-isopropylmalate synthase	95	111	75	101	114	82
"CDS;ID=637287915;locus_tag=SPO0423;product=DNA-binding protein, putative"	0	0	2	0	1	5
CDS;ID=637287916;locus_tag=SPO0424;product=membrane protein	8	9	3	7	10	8
CDS;ID=637287917;locus_tag=SPO0425;product=MORN repeat protein	129	109	98	105	134	126
CDS;ID=637287918;locus_tag=SPO0426;product=NAD(+) synthase	111	102	91	118	98	131
CDS;ID=637287919;locus_tag=SPO0427;product=hypothetical protein	10	15	10	10	12	12
"CDS;ID=637287920;locus_tag=SPO0428;product=amino acid deaminase, putative"	46	50	53	81	78	79

CDS;ID=637287921;locus_tag=SPO0429;product=metallo-beta-lactamase family protein	4	5	6	4	6	3
CDS;ID=637287922;locus_tag=SPO0430;product=glutamyl-tRNA synthetase	67	73	73	76	96	92
CDS;ID=637287923;locus_tag=SPO0431;product=hypothetical protein	14	14	7	7	14	14
CDS;ID=637287924;locus_tag=SPO0432;product=rrf2 family protein	76	51	57	97	89	74
"CDS;ID=637287925;locus_tag=SPO0433;product=ABC transporter, permease protein"	31	24	16	23	29	29
"CDS;ID=637287926;locus_tag=SPO0434;product=ABC transporter, ATP-binding protein"	23	7	15	12	17	13
"CDS;ID=637287927;locus_tag=SPO0435;product=acyl-CoA thioesterase, putative"	20	6	4	6	9	14
CDS;ID=637287928;locus_tag=SPO0436;product=hypothetical protein	11	10	2	7	8	5
CDS;ID=637287929;locus_tag=SPO0437;product=cold shock family protein	25	17	13	21	12	19
CDS;ID=637287930;locus_tag=SPO0438;product=ErfK/YbiS/YcfS/YnhG family protein/Tat d	33	11	20	24	22	25
CDS;ID=637287931;locus_tag=SPO0439;product=hypothetical protein	3	7	4	13	3	2
CDS;ID=637287932;locus_tag=SPO0440;product=thioesterase family protein	31	23	14	26	16	32
CDS;ID=637287933;locus_tag=SPO0441;product=hypothetical protein	6	8	6	4	2	8
CDS;ID=637287934;locus_tag=SPO0442;product=thioredoxin	23	7	9	13	7	12
CDS;ID=637287935;locus_tag=SPO0443;product=hypothetical protein	12	10	6	11	9	8
CDS;ID=637287936;locus_tag=SPO0444;product=hypothetical protein	6	11	14	11	6	12
CDS;ID=637287937;locus_tag=SPO0445;product=hypothetical protein	15	10	10	12	19	11
"CDS;ID=637287938;locus_tag=SPO0446;product=ABC transporter, ATP-binding protein"	121	132	112	149	212	114
CDS;ID=637287939;locus_tag=SPO0447;product=hypothetical protein	3	5	4	6	9	7
CDS;ID=637287940;locus_tag=SPO0448;product=MOSC domain protein	4	2	1	2	4	4
CDS;ID=637287941;locus_tag=SPO0449;product=nicotinic acid mononucleotide adenyltrar	64	62	42	70	77	66
CDS;ID=637287942;locus_tag=SPO0450;product=hypothetical protein	13	4	7	8	12	3
CDS;ID=637287943;locus_tag=SPO0451;product=D-alanyl-D-alanine carboxypeptidase/D-al	42	60	28	33	51	50
CDS;ID=637287944;locus_tag=SPO0452;product=hypothetical protein	18	14	13	22	21	18
CDS;ID=637287945;locus_tag=SPO0453;product=hypothetical protein	0	0	5	0	3	1
"CDS;ID=637287946;locus_tag=SPO0454;product=transcriptional regulator, LysR family'	6	12	7	6	5	8
CDS;ID=637287947;locus_tag=SPO0455;product=lysyl-tRNA synthetase	76	61	58	113	86	124
CDS;ID=637287948;locus_tag=SPO0456;product=hypothetical protein	27	39	31	35	25	42
CDS;ID=637287949;locus_tag=SPO0457;product=hypothetical protein	9	16	3	11	12	17
CDS;ID=637287950;locus_tag=SPO0458;product=hypothetical protein	22	16	12	14	17	20
CDS;ID=637287951;locus_tag=SPO0459;product=hypothetical protein	23	18	12	13	16	21
CDS;ID=637287952;locus_tag=SPO0460;product=hypothetical protein	18	30	33	36	42	35
CDS;ID=637287953;locus_tag=SPO0461;product=ParA family protein	37	52	42	46	62	60
CDS;ID=637287954;locus_tag=SPO0462;product=antibiotic biosynthesis monooxygenase do	6	3	3	3	3	4
CDS;ID=637287955;locus_tag=SPO0463;product=universal stress family protein	104	40	56	106	147	118
CDS;ID=637287956;locus_tag=SPO0464;product=hypothetical protein	50	57	37	59	57	77
CDS;ID=637287957;locus_tag=SPO0465;product=pyridoxamine 5'-phosphate oxidase family	21	12	5	5	34	14
"CDS;ID=637287958;locus_tag=SPO0466;product=transporter, formate/nitrate family"	22	10	13	14	16	15
"CDS;ID=637287959;locus_tag=SPO0467;product=transcriptional regulator, GntR family'	1	1	3	9	3	1
CDS;ID=637287960;locus_tag=SPO0468;product=alkylphosphonate utilization protein Phr	2	6	3	6	5	12
CDS;ID=637287961;locus_tag=SPO0469;product=alkylphosphonate utilization protein Phr	0	0	1	3	3	5
CDS;ID=637287962;locus_tag=SPO0470;product=alkylphosphonate utilization protein Phr	0	4	3	5	7	11
CDS;ID=637287963;locus_tag=SPO0471;product=alkylphosphonate utilization protein Phr	1	3	8	6	7	4

CDS;ID=637287964;locus_tag=SPO0472;product=alkylphosphonate utilization protein Phr	9	6	0	3	8	7
CDS;ID=637287965;locus_tag=SPO0473;product=alkylphosphonate utilization protein Phr	0	2	0	1	1	0
CDS;ID=637287966;locus_tag=SPO0474;product=alkylphosphonate utilization protein Phr	1	0	2	1	0	1
CDS;ID=637287967;locus_tag=SPO0475;product=hypothetical protein	4	6	0	7	5	9
CDS;ID=637287968;locus_tag=SPO0476;product=alkylphosphonate utilization protein Phr	3	4	7	7	9	5
CDS;ID=637287969;locus_tag=SPO0477;product=hypothetical protein	7	10	10	14	8	8
CDS;ID=637287970;locus_tag=SPO0478;product=hypothetical protein	15	15	14	9	4	7
"CDS;ID=637287971;locus_tag=SPO0479;product=ISSpo8, transposase"	40	52	26	35	57	37
tRNA;ID=640698516;locus_tag=SPO_tRNA-Arg-2	111	65	36	14	6	36
"CDS;ID=637287973;locus_tag=SPO0481;product=acetyltransferase, GNAT family"	8	6	8	10	9	18
CDS;ID=637287974;locus_tag=SPO0482;product=NADH-ubiquinone oxidoreductase family pı	285	219	172	252	123	281
"CDS;ID=637287975;locus_tag=SPO0483;product=microcystin dependent protein, putative	69	62	40	28	42	28
CDS;ID=637287976;locus_tag=SPO0484;product=50S ribosomal protein L2	539	531	545	638	1005	536
CDS;ID=637287977;locus_tag=SPO0485;product=ribosomal protein S19	308	259	286	283	517	282
CDS;ID=637287978;locus_tag=SPO0486;product=50S ribosomal protein L22	333	369	333	360	605	358
CDS;ID=637287979;locus_tag=SPO0487;product=30S ribosomal protein S3	209	221	196	169	329	187
CDS;ID=637287980;locus_tag=SPO0488;product=50S ribosomal protein L16	142	115	104	106	144	100
"CDS;ID=637287981;locus_tag=SPO0489;product=ISSpo2, transposase"	3	7	6	0	4	7
CDS;ID=637287982;locus_tag=SPO0490;product=calcium-binding domain protein	4	9	5	9	9	11
CDS;ID=637287983;locus_tag=SPO0491;product=hypothetical protein	42	33	33	36	44	29
CDS;ID=637287984;locus_tag=SPO0492;product=cyclic nucleotide-binding domain proteir	10	13	8	16	15	13
CDS;ID=637287985;locus_tag=SPO0493;product=ribosomal protein L29	180	161	157	171	282	151
CDS;ID=637287986;locus_tag=SPO0494;product=ribosomal protein S17	1409	1753	1319	1105	952	877
CDS;ID=637287987;locus_tag=SPO0495;product=ribosomal protein L14	135	130	154	173	220	185
CDS;ID=637287988;locus_tag=SPO0496;product=ribosomal protein L24	212	199	174	184	258	192
CDS;ID=637287989;locus_tag=SPO0497;product=ribosomal protein L5	299	277	295	282	498	268
CDS;ID=637287990;locus_tag=SPO0498;product=ribosomal protein S14	135	125	123	107	232	141
CDS;ID=637287991;locus_tag=SPO0499;product=ribosomal protein S8	90	96	76	85	160	100
CDS;ID=637287992;locus_tag=SPO0500;product=50S ribosomal protein L6	439	382	386	378	553	338
CDS;ID=637287993;locus_tag=SPO0501;product=ribosomal protein L18	431	403	388	318	514	309
CDS;ID=637287994;locus_tag=SPO0502;product=30S ribosomal protein S5	153	206	212	220	247	165
CDS;ID=637287995;locus_tag=SPO0503;product=ribosomal protein L30	319	201	234	202	171	137
CDS;ID=637287996;locus_tag=SPO0504;product=hypothetical protein	48	63	38	39	46	27
CDS;ID=637287997;locus_tag=SPO0505;product=ribosomal protein L15	107	128	127	133	148	130
CDS;ID=637287998;locus_tag=SPO0506;product=preprotein translocase SecY	218	157	165	219	225	209
CDS;ID=637287999;locus_tag=SPO0507;product=adenylate kinase	136	114	104	134	114	153
CDS;ID=637288000;locus_tag=SPO0508;product=hypothetical protein	25	25	29	25	18	21
CDS;ID=637288001;locus_tag=SPO0509;product=30S ribosomal protein S13	224	200	252	229	327	218
CDS;ID=637288002;locus_tag=SPO0510;product=30S ribosomal protein S11	133	139	145	130	235	128
CDS;ID=637288003;locus_tag=SPO0511;product=DNA-directed RNA polymerase alpha subuni	359	308	358	330	521	351
CDS;ID=637288004;locus_tag=SPO0512;product=ribosomal protein L17	152	128	122	140	217	90
CDS;ID=637288005;locus_tag=SPO0513;product=hypothetical protein	3	2	3	3	2	2
"CDS;ID=637288006;locus_tag=SPO0514;product=periplasmic serine protease, DO/DeqQ fa	62	46	53	62	84	55

"CDS;ID=637288007;locus_tag=SPO0515;product=ATPase, AAA family"	18	11	8	20	13	18
CDS;ID=637288008;locus_tag=SPO0516;product=ribosomal large subunit pseudouridine sy	51	38	54	62	56	71
"CDS;ID=637288009;locus_tag=SPO0517;product=HAD-superfamily hydrolase, subfamily IA	21	28	23	19	35	35
CDS;ID=637288010;locus_tag=SPO0518;product=hypothetical protein	49	38	40	61	71	62
"CDS;ID=637288011;locus_tag=SPO0519;product=glutamate/glutamine/aspartate/asparagin	870	645	688	548	735	784
"CDS;ID=637288012;locus_tag=SPO0520;product=glutamate/glutamine/aspartate/asparagin	32	35	22	35	69	29
"CDS;ID=637288013;locus_tag=SPO0521;product=glutamate/glutamine/aspartate/asparagin	40	47	29	41	42	37
"CDS;ID=637288014;locus_tag=SPO0522;product=glutamate/glutamine/aspartate/asparagin	139	98	90	94	85	96
CDS;ID=637288015;locus_tag=SPO0523;product=phosphoglycerate mutase family protein	21	27	19	28	10	14
CDS;ID=637288016;locus_tag=SPO0524;product=hypothetical protein	5	5	4	1	4	8
"CDS;ID=637288017;locus_tag=SPO0525;product=sterol desaturase, homolog"	5	5	2	1	5	6
CDS;ID=637288018;locus_tag=SPO0526;product=acetylglutamate kinase	93	87	116	146	97	126
"CDS;ID=637288019;locus_tag=SPO0527;product=oxidoreductase, short chain dehydrogena	72	56	47	46	58	73
CDS;ID=637288020;locus_tag=SPO0528;product=hypothetical protein	25	20	9	23	10	18
CDS;ID=637288021;locus_tag=SPO0529;product=hypothetical protein	54	52	40	36	32	36
CDS;ID=637288022;locus_tag=SPO0530;product=GTP-binding protein	34	28	26	22	29	28
CDS;ID=637288023;locus_tag=SPO0531;product=MOSC domain protein	16	14	4	9	13	5
CDS;ID=637288024;locus_tag=SPO0532;product=putative inner membrane protein transloc	103	116	83	111	118	95
"CDS;ID=637288025;locus_tag=SPO0533;product=diguanylate cyclase, putative"	14	4	9	9	5	7
CDS;ID=637288026;locus_tag=SPO0534;product=hypothetical protein	8	8	7	23	13	10
CDS;ID=637288027;locus_tag=SPO0535;product=K+-dependent Na+/Ca+ exchanger related-p	11	10	16	14	21	16
CDS;ID=637288028;locus_tag=SPO0536;product=conserved hypothetical protein TIGR00278	6	4	1	7	9	3
CDS;ID=637288029;locus_tag=SPO0537;product=ribonuclease P protein component	15	7	6	11	16	7
CDS;ID=637288030;locus_tag=SPO0538;product=50S ribosomal protein L34	156	107	94	155	167	125
CDS;ID=637288031;locus_tag=SPO0539;product=hypothetical protein	2	9	6	8	6	4
"CDS;ID=637288032;locus_tag=SPO0540;product=mercuric reductase, putative"	32	19	19	18	31	21
CDS;ID=637288033;locus_tag=SPO0541;product=sensor histidine kinase	50	47	50	35	39	59
tRNA;ID=640698517;locus_tag=SPO_tRNA-Arg-3	478	265	177	203	39	220
CDS;ID=637288035;locus_tag=SPO0543;product=hypothetical protein	5	1	7	3	5	4
CDS;ID=637288036;locus_tag=SPO0544;product=aminomethyl transferase family protein	22	7	8	13	11	11
CDS;ID=637288037;locus_tag=SPO0545;product=excinuclease ABC subunit B	87	93	71	49	108	78
CDS;ID=637288038;locus_tag=SPO0546;product=hypothetical protein	17	7	22	20	34	26
CDS;ID=637288039;locus_tag=SPO0547;product=hypothetical protein	3	4	2	6	8	7
CDS;ID=637288040;locus_tag=SPO0548;product=hypothetical protein	4	7	15	4	4	10
CDS;ID=637288041;locus_tag=SPO0549;product=hypothetical protein	4	7	3	8	1	5
CDS;ID=637288042;locus_tag=SPO0550;product=hypothetical protein	10	8	8	9	6	10
CDS;ID=637288043;locus_tag=SPO0551;product=hypothetical protein	9	5	12	10	13	19
CDS;ID=637288044;locus_tag=SPO0552;product=phosphoglycerate mutase family protein	3	8	2	3	7	6
"CDS;ID=637288045;locus_tag=SPO0553;product=glycosyltransferase, group 1"	24	20	14	14	20	28
"CDS;ID=637288046;locus_tag=SPO0554;product=glycosyl transferase, group 1 family pr	29	25	21	14	15	22
CDS;ID=637288047;locus_tag=SPO0555;product=hypothetical protein	40	42	44	35	41	60
CDS;ID=637288048;locus_tag=SPO0556;product=UDP-glucose/GDP-mannose dehydrogenase fa	93	80	92	89	114	98
CDS;ID=637288049;locus_tag=SPO0557;product=mechanosensitive ion channel family prot	66	57	55	65	63	61

"CDS;ID=637288050;locus_tag=SPO0558;product=oligopeptide ABC transporter, permease	32	15	17	16	18	18
"CDS;ID=637288051;locus_tag=SPO0559;product=oligopeptide ABC transporter, permease	13	15	6	8	12	7
"CDS;ID=637288052;locus_tag=SPO0560;product=oligopeptide ABC transporter, periplasm	58	61	39	35	72	60
CDS;ID=640735050;locus_tag=SPO0561	67	61	51	35	56	60
CDS;ID=637288053;locus_tag=SPO0562;product=hypothetical protein	11	10	14	8	12	18
"CDS;ID=637288054;locus_tag=SPO0563;product=glutathione S-transferase, putative"	14	10	9	6	12	12
CDS;ID=637288055;locus_tag=SPO0564;product=aminomethyl transferase family protein	11	10	14	13	9	14
CDS;ID=637288056;locus_tag=SPO0565;product=trimethylamine methyltransferase family	1	3	2	8	4	5
"CDS;ID=637288057;locus_tag=SPO0566;product=transcriptional regulator, TetR family'	3	9	4	8	3	2
"CDS;ID=637288058;locus_tag=SPO0567;product=oxidoreductase, FAD-binding"	8	7	3	8	5	14
CDS;ID=637288059;locus_tag=SPO0568;product=indolepyruvate ferredoxin oxidoreductase	121	123	105	130	267	159
"CDS;ID=637288060;locus_tag=SPO0569;product=transcriptional regulator, AsnC family'	2	6	3	3	5	11
CDS;ID=637288061;locus_tag=SPO0570;product=hypothetical protein	10	7	11	2	11	17
CDS;ID=637288062;locus_tag=SPO0571;product=PKD domain protein	4	10	11	8	7	5
CDS;ID=637288063;locus_tag=SPO0572;product=adenylate/guanylate cyclase	9	15	7	9	15	16
CDS;ID=637288064;locus_tag=SPO0573;product=hypothetical protein	29	51	31	33	29	60
"CDS;ID=637288065;locus_tag=SPO0574;product=ABC transporter, transmembrane ATP-binc	109	94	56	69	87	95
CDS;ID=637288066;locus_tag=SPO0575;product=acyl-CoA dehydrogenase	6	6	0	7	11	6
"CDS;ID=637288067;locus_tag=SPO0576;product=transcriptional regulator, TetR family'	1	3	4	3	4	5
CDS;ID=637288068;locus_tag=SPO0577;product=hypothetical protein	16	9	10	12	15	19
CDS;ID=637288069;locus_tag=SPO0578;product=acetyl-CoA carboxylase carboxyltransfera	12	3	3	5	5	8
"CDS;ID=637288070;locus_tag=SPO0579;product=acetyl-CoA carboxylase, biotin carboxy	6	3	2	4	5	6
"CDS;ID=637288071;locus_tag=SPO0580;product=oxidoreductase, 2-nitropropane dioxyger	5	5	4	3	7	6
CDS;ID=637288072;locus_tag=SPO0581;product=hypothetical protein	4	4	1	2	7	1
CDS;ID=637288073;locus_tag=SPO0582;product=acyl-CoA dehydrogenase	77	54	34	61	50	59
"CDS;ID=637288074;locus_tag=SPO0583;product=transcriptional regulator, LysR family'	1	5	3	3	7	1
CDS;ID=637288075;locus_tag=SPO0584;product=aspartate aminotransferase	15	11	9	3	0	3
CDS;ID=637288076;locus_tag=SPO0585;product=dehydrogenase/transketolase family prote	33	26	26	12	17	12
CDS;ID=637288077;locus_tag=SPO0586;product=acyl-carrier domain protein	6	10	12	0	4	8
CDS;ID=637288078;locus_tag=SPO0587;product=hypothetical protein	9	14	14	11	16	17
"CDS;ID=637288079;locus_tag=SPO0588;product=transcriptional regulator, LysR family'	9	5	12	11	3	6
"CDS;ID=637288080;locus_tag=SPO0589;product=oxidoreductase, NAD-binding/iron-sulfur	4	2	5	3	7	12
"CDS;ID=637288081;locus_tag=SPO0590;product=transcriptional regulator, LacI family'	15	20	6	11	11	8
"CDS;ID=637288082;locus_tag=SPO0591;product=TRAP dicarboxylate transporter, DctP su	4	6	4	2	2	5
"CDS;ID=637288083;locus_tag=SPO0592;product=TRAP dicarboxylate transporter, DctQ su	0	0	0	3	2	3
"CDS;ID=637288084;locus_tag=SPO0593;product=TRAP dicarboxylate transporter, DctM su	3	3	1	2	3	6
CDS;ID=637288085;locus_tag=SPO0594;product=histidinol dehydrogenase	6	6	4	4	4	0
CDS;ID=637288086;locus_tag=SPO0595;product=gluconate 5-dehydrogenase	0	1	0	2	0	0
"CDS;ID=637288087;locus_tag=SPO0596;product=sorbitol dehydrogenase, putative"	0	1	0	3	2	0
CDS;ID=637288088;locus_tag=SPO0597;product=universal stress family protein	1	1	4	1	1	0
CDS;ID=637288089;locus_tag=SPO0598;product=FMN-dependent alpha-hydroxy acid dehydro	1	3	0	3	1	7
CDS;ID=637288090;locus_tag=SPO0599;product=agmatinase	6	11	5	10	7	8
CDS;ID=637288091;locus_tag=SPO0600;product=carboxynorspermidine decarboxylase	17	20	13	16	16	25

CDS;ID=637288092;locus_tag=SPO0601;product=saccharopine dehydrogenase	123	97	130	118	200	155
CDS;ID=637288093;locus_tag=SPO0602;product=arginine decarboxylase	69	77	68	93	154	105
CDS;ID=637288094;locus_tag=SPO0603;product=hypothetical protein	1	7	4	9	5	5
CDS;ID=637288095;locus_tag=SPO0604;product=hypothetical protein	5	10	9	11	0	9
"CDS;ID=637288096;locus_tag=SPO0605;product=transcriptional regulator, MarR family'	4	7	3	2	10	3
CDS;ID=637288097;locus_tag=SPO0606;product=hypothetical protein	7	1	6	11	6	6
"CDS;ID=637288098;locus_tag=SPO0607;product=proline racemase, putative"	6	4	0	1	4	3
"CDS;ID=637288099;locus_tag=SPO0608;product=sugar ABC transporter, periplasmic suga	551	533	458	315	363	457
"CDS;ID=637288100;locus_tag=SPO0609;product=sugar ABC transporter, ATP-binding prot	41	46	24	23	39	22
"CDS;ID=637288101;locus_tag=SPO0610;product=sugar ABC transporter, ATP-binding prot	44	43	22	34	46	51
"CDS;ID=637288102;locus_tag=SPO0611;product=sugar ABC transporter, permease protein	50	66	36	33	51	61
"CDS;ID=637288103;locus_tag=SPO0612;product=sugar ABC transporter, permease protein	25	27	26	15	24	19
CDS;ID=637288104;locus_tag=SPO0613;product=hypothetical protein	25	17	14	11	17	9
"CDS;ID=637288105;locus_tag=SPO0614;product=transcriptional regulator, putative"	156	176	136	109	157	119
CDS;ID=637288106;locus_tag=SPO0615;product=hypothetical protein	26	24	15	25	23	17
"CDS;ID=637288107;locus_tag=SPO0616;product=oxidoreductase, zinc-binding dehydrogen	7	7	2	5	5	2
tRNA;ID=640698518;locus_tag=SPO_tRNA-Gln-2	1582	773	545	407	77	477
"CDS;ID=637288109;locus_tag=SPO0618;product=transcriptional regulator, AraC family'	3	15	6	2	7	4
CDS;ID=637288110;locus_tag=SPO0619;product=sterol desaturase family protein	8	33	21	28	16	36
CDS;ID=637288111;locus_tag=SPO0620;product=hypothetical protein	1	1	2	2	3	2
CDS;ID=637288112;locus_tag=SPO0621;product=ankrin repeat protein	14	18	8	13	11	11
"CDS;ID=637288113;locus_tag=SPO0622;product=ISSpo1, transposase"	50	47	54	37	20	25
CDS;ID=637288114;locus_tag=SPO0623;product=cytochrome c oxidase domain protein	9	11	12	16	11	18
CDS;ID=637288115;locus_tag=SPO0624;product=hypothetical protein	26	24	22	19	28	25
CDS;ID=640735051;locus_tag=SPO0625	0	2	1	1	4	2
CDS;ID=640735052;locus_tag=SPO0626	1	2	0	1	0	2
CDS;ID=640735053;locus_tag=SPO0627	8	20	12	8	9	21
"CDS;ID=637288116;locus_tag=SPO0628;product=transposase, degenerate"	0	2	4	3	1	4
"CDS;ID=637288117;locus_tag=SPO0629;product=ISSpo3, transposase"	47	64	56	61	57	53
CDS;ID=640735054;locus_tag=SPO0630	11	14	11	11	10	6
CDS;ID=637288118;locus_tag=SPO0631;product=hypothetical protein	16	12	11	25	29	16
CDS;ID=637288119;locus_tag=SPO0632;product=2-hydroxyacid dehydrogenase	126	126	98	116	95	177
CDS;ID=637288120;locus_tag=SPO0633;product=gamma-glutamyltranspeptidase	72	56	74	54	57	53
"CDS;ID=637288121;locus_tag=SPO0634;product=oxidoreductase, FAD-binding"	13	11	11	13	13	7
CDS;ID=637288122;locus_tag=SPO0635;product=aminomethyl transferase family protein	418	413	413	451	505	496
CDS;ID=637288123;locus_tag=SPO0636;product=EF hand domain protein	1	2	5	3	1	2
CDS;ID=637288124;locus_tag=SPO0637;product=hypothetical protein	0	1	0	0	0	0
CDS;ID=637288125;locus_tag=SPO0638;product=hypothetical protein	7	12	12	3	10	4
"CDS;ID=637288126;locus_tag=SPO0639;product=transcriptional regulator, LysR family'	6	5	8	7	4	5
CDS;ID=637288127;locus_tag=SPO0640;product=4-carboxymuconolactone decarboxylase dom	19	13	4	16	8	11
CDS;ID=637288128;locus_tag=SPO0641;product=hypothetical protein	20	15	17	10	13	9
"CDS;ID=637288129;locus_tag=SPO0642;product=oxidoreductase, FAD-binding"	18	17	20	15	23	26
"CDS;ID=637288130;locus_tag=SPO0643;product=oxidoreductase, aldo/keto reductase far	31	37	28	23	41	32

CDS;ID=637288131;locus_tag=SPO0644;product=hypothetical protein	35	22	10	12	24	31
"CDS;ID=637288132;locus_tag=SPO0645;product=multidrug resistance efflux pump, putat	38	34	44	23	35	36
CDS;ID=637288133;locus_tag=SPO0646;product=hypothetical protein	5	4	7	8	10	6
CDS;ID=637288134;locus_tag=SPO0647;product=hypothetical protein	24	9	18	20	28	18
CDS;ID=637288135;locus_tag=SPO0648;product=bmp family protein	283	226	261	207	242	233
"CDS;ID=637288136;locus_tag=SPO0649;product=sugar ABC transporter, permease protein	192	186	179	164	258	111
"CDS;ID=637288137;locus_tag=SPO0650;product=sugar ABC transporter, permease protein	38	28	21	22	24	38
"CDS;ID=637288138;locus_tag=SPO0651;product=sugar ABC transporter, ATP-binding prot	56	45	69	39	53	48
CDS;ID=637288139;locus_tag=SPO0652;product=xanthine dehydrogenase accessory factor	54	48	46	30	56	42
"CDS;ID=637288140;locus_tag=SPO0653;product=xanthine dehydrogenase, B subunit"	112	98	90	86	99	80
"CDS;ID=637288141;locus_tag=SPO0654;product=xanthine dehydrogenase, A subunit"	51	33	42	25	40	45
CDS;ID=637288142;locus_tag=SPO0655;product=hypothetical protein	5	5	7	2	7	4
CDS;ID=637288143;locus_tag=SPO0656;product=DNA polymerase III subunit alpha	96	85	63	67	103	97
CDS;ID=637288144;locus_tag=SPO0657;product=CobW/P47K family protein	9	5	4	1	4	11
CDS;ID=637288145;locus_tag=SPO0658;product=amidohydrolase domain protein	8	3	7	4	8	10
"CDS;ID=637288146;locus_tag=SPO0659;product=transcriptional regulator, LysR family"	7	9	7	8	4	10
"CDS;ID=637288147;locus_tag=SPO0660;product=peptide ABC transporter, periplasmic pe	5	6	8	1	2	4
"CDS;ID=637288148;locus_tag=SPO0661;product=peptide ABC transporter, permease prote	1	0	5	2	3	8
"CDS;ID=637288149;locus_tag=SPO0662;product=peptide ABC transporter, permease prote	0	3	1	0	0	1
"CDS;ID=637288150;locus_tag=SPO0663;product=peptide ABC transporter, ATP-binding pr	0	0	0	0	1	4
"CDS;ID=637288151;locus_tag=SPO0664;product=peptide ABC transporter, ATP-binding pr	2	0	1	1	4	2
"CDS;ID=637288152;locus_tag=SPO0665;product=SlyX protein, putative"	24	24	7	27	18	28
CDS;ID=637288153;locus_tag=SPO0666;product=enoyl-CoA hydratase	2	4	2	4	4	3
CDS;ID=637288154;locus_tag=SPO0667;product=histidyl-tRNA synthetase	109	92	103	109	157	130
CDS;ID=637288155;locus_tag=SPO0668;product=ATP phosphoribosyltransferase regulatory	41	43	42	47	40	44
CDS;ID=637288156;locus_tag=SPO0669;product=ATP phosphoribosyltransferase	30	28	18	23	25	38
"CDS;ID=637288157;locus_tag=SPO0670;product=DNA polymerase III, alpha subunit, puta	28	20	9	10	18	16
CDS;ID=637288158;locus_tag=SPO0671;product=hypothetical protein	3	2	1	2	4	2
CDS;ID=637288159;locus_tag=SPO0672;product=hypothetical protein	6	5	1	4	4	9
CDS;ID=637288160;locus_tag=SPO0673;product=taurine--pyruvate aminotransferase	173	158	189	175	182	166
"CDS;ID=637288161;locus_tag=SPO0674;product=taurine ABC transporter, periplasmic ta	13	15	10	11	14	18
"CDS;ID=637288162;locus_tag=SPO0675;product=taurine ABC transporter, ATP-binding pr	3	3	3	1	2	6
"CDS;ID=637288163;locus_tag=SPO0676;product=taurine ABC transporter, permease prote	16	10	2	9	11	10
CDS;ID=637288164;locus_tag=SPO0677;product=acyl-CoA synthase	37	28	24	33	23	18
"CDS;ID=637288165;locus_tag=SPO0678;product=agmatinase, putative"	64	91	52	31	33	64
CDS;ID=637288166;locus_tag=SPO0679;product=maleylacetoacetate isomerase	10	6	6	10	20	19
CDS;ID=637288167;locus_tag=SPO0680;product=glyoxalase family protein	29	25	13	28	23	22
CDS;ID=637288168;locus_tag=SPO0681;product=hypothetical protein	12	11	10	15	19	20
CDS;ID=637288169;locus_tag=SPO0682;product=hypothetical protein	33	20	27	21	51	44
CDS;ID=637288170;locus_tag=SPO0683;product=metallo-beta-lactamase family protein	46	38	51	19	25	33
CDS;ID=637288171;locus_tag=SPO0684;product=glyoxalase family protein	2	3	11	2	7	8
CDS;ID=637288172;locus_tag=SPO0685;product=fumarylacetoacetase	6	12	16	4	11	11
"CDS;ID=637288173;locus_tag=SPO0686;product=homogentisate 1,2-dioxygenase"	10	13	14	4	13	11

"CDS;ID=637288174;locus_tag=SPO0687;product=transcriptional regulator, MarR family"	96	77	69	66	86	83
CDS;ID=637288175;locus_tag=SPO0688;product=adenylate/guanylate cyclase	39	40	31	29	42	51
CDS;ID=637288176;locus_tag=SPO0689;product=hypothetical protein	38	34	32	34	37	48
CDS;ID=637288177;locus_tag=SPO0690;product=hypothetical protein	4	1	2	6	3	12
CDS;ID=637288178;locus_tag=SPO0691;product=GTP-binding protein Era	75	66	74	125	114	93
CDS;ID=637288179;locus_tag=SPO0692;product=multicopper oxidase	7	3	8	4	8	11
CDS;ID=637288180;locus_tag=SPO0693;product=isobutyryl-CoA dehydrogenase	47	49	29	38	52	44
CDS;ID=637288181;locus_tag=SPO0694;product=hypothetical protein	6	7	4	5	4	1
CDS;ID=637288182;locus_tag=SPO0695;product=hypothetical protein	19	18	5	16	13	10
tRNA;ID=640698519;locus_tag=SPO_tRNA-Val-2	1467	946	650	846	293	351
"CDS;ID=637288184;locus_tag=SPO0697;product=molybdate ABC transporter, ATP-binding	4	1	1	2	9	6
CDS;ID=637288185;locus_tag=SPO0698;product=molybdate transport permease protein	6	3	4	5	9	8
"CDS;ID=637288186;locus_tag=SPO0699;product=molybdate ABC transporter, periplasmic	7	4	1	9	10	7
"CDS;ID=637288187;locus_tag=SPO0700;product=molybdenum-binding transcriptional regu	5	11	6	10	8	13
"CDS;ID=637288188;locus_tag=SPO0701;product=glyceraldehyde-3-phosphate dehydrogenas	62	71	36	78	81	76
"CDS;ID=637288189;locus_tag=SPO0702;product=oligopeptide ABC transporter, ATP-bindi	13	9	6	2	4	10
"CDS;ID=637288190;locus_tag=SPO0703;product=oligopeptide ABC transporter, ATP-bindi	18	11	7	8	17	8
"CDS;ID=637288191;locus_tag=SPO0704;product=oligopeptide ABC transporter, permease	7	4	7	2	8	5
"CDS;ID=637288192;locus_tag=SPO0705;product=oligopeptide ABC transporter, permease	14	9	7	18	13	4
"CDS;ID=637288193;locus_tag=SPO0706;product=oligopeptide ABC transporter, periplasm	92	65	70	53	66	61
"CDS;ID=637288194;locus_tag=SPO0707;product=transcriptional regulator, DeoR family"	46	53	37	30	65	27
CDS;ID=637288195;locus_tag=SPO0708;product=invasion protein IbeA	22	7	19	14	8	8
CDS;ID=637288196;locus_tag=SPO0709;product=phosphoenolpyruvate carboxykinase	94	69	80	100	112	110
CDS;ID=637288197;locus_tag=SPO0710;product=DNA-binding response regulator ChvI	53	80	65	78	96	66
CDS;ID=637288198;locus_tag=SPO0711;product=sensor histidine kinase ChvG	96	83	67	69	67	84
CDS;ID=637288199;locus_tag=SPO0712;product=Hpr serine kinase/phosphatase domain pro	17	12	9	11	8	9
CDS;ID=637288200;locus_tag=SPO0713;product=hypothetical protein	34	30	29	31	27	28
"CDS;ID=637288201;locus_tag=SPO0714;product=PTS system IIA component, Man family"	88	81	78	82	84	106
CDS;ID=637288202;locus_tag=SPO0715;product=phosphocarrier protein HPr	88	68	60	85	75	80
CDS;ID=637288203;locus_tag=SPO0716;product=hypothetical protein	52	28	30	47	49	26
CDS;ID=637288204;locus_tag=SPO0717;product=3-hydroxybutyryl-CoA dehydrogenase	111	86	61	79	58	104
CDS;ID=637288205;locus_tag=SPO0718;product=hypothetical protein	5	0	0	3	2	1
"CDS;ID=637288206;locus_tag=SPO0719;product=electron transfer flavoprotein, alpha s	167	126	126	165	161	173
"CDS;ID=637288207;locus_tag=SPO0720;product=electron transfer flavoprotein, beta su	176	185	148	169	245	214
CDS;ID=637288208;locus_tag=SPO0721;product=glyoxalase family protein	6	5	3	1	5	1
"CDS;ID=637288209;locus_tag=SPO0722;product=ATP:cob(I)alamin adenosyltransferase, p	17	24	18	24	20	20
CDS;ID=637288210;locus_tag=SPO0723;product=hypothetical protein	94	80	94	98	87	116
"CDS;ID=637288211;locus_tag=SPO0724;product=oxidoreductase, short-chain dehydrogena	12	7	8	10	12	5
CDS;ID=637288212;locus_tag=SPO0725;product=hypothetical protein	20	12	16	10	14	5
CDS;ID=637288213;locus_tag=SPO0726;product=DNA topoisomerase IV subunit A	168	170	138	161	215	165
CDS;ID=637288214;locus_tag=SPO0727;product=hypothetical protein	104	83	81	116	126	106
CDS;ID=637288215;locus_tag=SPO0728;product=translation elongation factor Tu	549	534	505	480	660	483
CDS;ID=637288216;locus_tag=SPO0729;product=hypothetical protein	34	36	23	29	19	37

tRNA;ID=640698520;locus_tag=SPO_tRNA-Trp-2	479	215	158	164	23	79
CDS;ID=637288218;locus_tag=SPO0731;product=glycerol-3-phosphate regulon repressor	65	36	71	70	81	69
CDS;ID=637288219;locus_tag=SPO0732;product=aerobic glycerol-3-phosphate dehydrogena	155	115	118	148	182	174
"CDS;ID=637288220;locus_tag=SPO0733;product=esterase, putative"	17	18	24	21	23	27
CDS;ID=637288221;locus_tag=SPO0734;product=PaaX domain protein	16	16	9	6	13	9
CDS;ID=637288222;locus_tag=SPO0735;product=phenylacetic acid degradation protein Pa	36	15	16	19	34	30
"CDS;ID=637288223;locus_tag=SPO0736;product=TRAP dicarboxylate transporter, DctM su	12	6	5	7	1	7
"CDS;ID=637288224;locus_tag=SPO0737;product=TRAP dicarboxylate transporter family p	0	3	0	0	1	0
"CDS;ID=637288225;locus_tag=SPO0738;product=TRAP dicarboxylate transporter, DctP su	9	9	2	9	12	8
CDS;ID=637288226;locus_tag=SPO0739;product=enoyl-CoA hydratase/isomerase/3-hydroxya	8	4	3	4	9	11
CDS;ID=637288227;locus_tag=SPO0740;product=enoyl-CoA hydratase	26	26	16	20	17	27
CDS;ID=637288228;locus_tag=SPO0741;product=phenylacetic acid degradation protein Pa	23	24	14	19	34	20
CDS;ID=637288229;locus_tag=SPO0742;product=phenylacetate-CoA ligase	33	39	35	40	37	37
"CDS;ID=637288230;locus_tag=SPO0743;product=transcriptional regulator, TetR family"	18	18	11	21	9	29
CDS;ID=637288231;locus_tag=SPO0744;product=hypothetical protein	7	9	9	11	10	5
CDS;ID=637288232;locus_tag=SPO0745;product=hypothetical protein	10	26	17	17	21	14
CDS;ID=637288233;locus_tag=SPO0746;product=hypothetical protein	16	16	13	14	26	7
CDS;ID=637288234;locus_tag=SPO0747;product=peroxidase family protein	2	2	2	2	4	4
CDS;ID=637288235;locus_tag=SPO0748;product=hypothetical protein	2	2	4	3	5	5
CDS;ID=637288236;locus_tag=SPO0749;product=hypothetical protein	1	1	0	0	0	2
CDS;ID=637288237;locus_tag=SPO0750;product=hypothetical protein	3	13	5	8	2	3
CDS;ID=637288238;locus_tag=SPO0751;product=adenylate/guanylate cyclase	28	17	11	26	18	15
"CDS;ID=637288239;locus_tag=SPO0752;product=phenylacetic acid degradation protein,	61	43	38	47	50	62
CDS;ID=637288240;locus_tag=SPO0753;product=phenylacetic acid degradation oxidoreduc	71	69	56	84	115	93
CDS;ID=637288241;locus_tag=SPO0754;product=phenylacetic acid degradation protein Pa	54	46	40	48	77	52
CDS;ID=637288242;locus_tag=SPO0755;product=phenylacetic acid degradation protein Pa	32	31	39	32	41	37
CDS;ID=637288243;locus_tag=SPO0756;product=phenylacetic acid degradation protein Pa	36	22	23	27	28	23
CDS;ID=637288244;locus_tag=SPO0757;product=phenylacetic acid degradation protein Pa	167	147	133	165	188	230
CDS;ID=637288245;locus_tag=SPO0758;product=acetyl-CoA acetyltransferase	104	107	95	94	160	128
CDS;ID=637288246;locus_tag=SPO0759;product=hypothetical protein	23	7	15	11	24	36
CDS;ID=637288247;locus_tag=SPO0760;product=4-carboxymuconolactone decarboxylase dor	1	0	0	1	1	2
CDS;ID=637288248;locus_tag=SPO0761;product=cyclase family protein	5	2	4	6	4	6
"CDS;ID=637288249;locus_tag=SPO0762;product=transcriptional regulator, GntR family"	17	40	22	24	33	25
"CDS;ID=637288250;locus_tag=SPO0763;product=glutamine amidotransferase, class I"	0	1	2	2	3	2
CDS;ID=637288251;locus_tag=SPO0764;product=cytochrome P450 family protein	7	6	5	9	10	7
CDS;ID=637288252;locus_tag=SPO0765;product=glutamine synthetase family protein	14	17	8	22	17	12
CDS;ID=637288253;locus_tag=SPO0766;product=iron-sulfur cluster-binding protein	3	5	0	2	1	1
"CDS;ID=637288254;locus_tag=SPO0767;product=methyltransferase, FkbM family"	59	39	44	38	48	51
"CDS;ID=637288255;locus_tag=SPO0768;product=transcriptional regulator, IclR family"	25	9	7	5	13	9
CDS;ID=637288256;locus_tag=SPO0769;product=hypothetical protein	2	4	6	4	4	9
CDS;ID=637288257;locus_tag=SPO0770;product=hypothetical protein	1	1	3	1	3	5
CDS;ID=637288258;locus_tag=SPO0771;product=membrane protein	4	4	3	3	10	5
CDS;ID=637288259;locus_tag=SPO0772;product=enoyl-CoA hydratase/isomerase/3-hydroxya	76	79	60	55	68	69

CDS;ID=637288260;locus_tag=SPO0773;product=acetyl-CoA acyltransferase/thiolase fami	45	43	32	48	57	35
CDS;ID=637288261;locus_tag=SPO0774;product=acyl-CoA dehydrogenase family protein	16	6	17	4	30	18
CDS;ID=637288262;locus_tag=SPO0775;product=acyl-CoA dehydrogenase family protein	6	10	6	4	19	8
CDS;ID=637288263;locus_tag=SPO0776;product=conserved hypothetical protein TIGR0036	5	4	3	3	1	4
CDS;ID=637288264;locus_tag=SPO0777;product=enoyl-CoA hydratase	9	9	3	1	1	8
"CDS;ID=637288265;locus_tag=SPO0778;product=long-chain-fatty-acid--CoA ligase, puta	4	8	3	1	5	2
CDS;ID=637288266;locus_tag=SPO0779;product=ribulose-phosphate 3-epimerase	32	21	21	33	36	31
"CDS;ID=637288267;locus_tag=SPO0780;product=phosphonate ABC transporter, ATP-bindi	1	0	0	0	0	2
"CDS;ID=637288268;locus_tag=SPO0781;product=phosphonate ABC transporter, periplasm	0	1	0	1	2	1
"CDS;ID=637288269;locus_tag=SPO0782;product=phosphonate ABC transporter, permease p	0	0	0	0	1	1
"CDS;ID=637288270;locus_tag=SPO0783;product=phosphonate ABC transporter, permease p	0	0	0	1	2	2
"CDS;ID=637288271;locus_tag=SPO0784;product=chloramphenicol acetyltransferase, puta	1	3	2	2	0	1
"CDS;ID=637288272;locus_tag=SPO0785;product=2-dehydro-3-deoxygalactonokinase, puta	13	19	12	12	11	21
CDS;ID=637288273;locus_tag=SPO0786;product=thioesterase family protein	25	24	27	35	34	40
CDS;ID=637288274;locus_tag=SPO0787;product=phosphoglycolate phosphatase	19	20	12	22	38	28
CDS;ID=637288275;locus_tag=SPO0788;product=metallo-beta-lactamase family protein	19	36	8	18	17	15
CDS;ID=637288276;locus_tag=SPO0789;product=hypothetical protein	5	1	0	4	3	8
CDS;ID=637288277;locus_tag=SPO0790;product=hypothetical protein	7	2	3	5	3	8
"CDS;ID=637288278;locus_tag=SPO0791;product=M23/M37 peptidase/aminotransferase, cla	64	51	45	59	51	67
CDS;ID=637288279;locus_tag=SPO0792;product=6-phosphogluconate dehydrogenase domain	54	65	43	41	70	69
CDS;ID=637288280;locus_tag=SPO0793;product=Cu(I)-responsive transcriptional regula	2	3	0	0	1	3
CDS;ID=637288281;locus_tag=SPO0794;product=copper-translocating P-type ATPase	8	4	7	7	16	9
"CDS;ID=637288282;locus_tag=SPO0795;product=oxidoreductase, short chain dehydrogena	4	0	0	0	0	3
CDS;ID=637288283;locus_tag=SPO0796;product=hypothetical protein	4	8	2	10	3	3
CDS;ID=637288284;locus_tag=SPO0797;product=bile acid transporter family protein	3	10	3	2	4	4
CDS;ID=637288285;locus_tag=SPO0798;product=monooxygenase domain protein	3	7	6	4	5	4
"CDS;ID=637288286;locus_tag=SPO0799;product=transcriptional regulator, TetR family"	8	3	3	1	1	1
"CDS;ID=637288287;locus_tag=SPO0800;product=choline sulfatase, putative"	5	2	2	0	10	2
CDS;ID=637288288;locus_tag=SPO0801;product=4-coumarate:CoA ligase	11	5	7	10	4	14
CDS;ID=637288289;locus_tag=SPO0802;product=30S ribosomal protein S1	479	490	423	442	684	399
CDS;ID=637288290;locus_tag=SPO0803;product=NUDIX domain protein	12	14	12	7	8	12
CDS;ID=637288291;locus_tag=SPO0804;product=integration host factor beta subunit	195	147	155	163	126	197
CDS;ID=637288292;locus_tag=SPO0805;product=hypothetical protein	24	25	18	25	40	42
CDS;ID=637288293;locus_tag=SPO0806;product=N-(5'-phosphoribosyl)anthranilate isome	38	54	38	48	44	45
CDS;ID=637288294;locus_tag=SPO0807;product=hypothetical protein	0	2	2	1	0	2
CDS;ID=637288295;locus_tag=SPO0808;product=tryptophan synthase subunit beta	216	180	187	203	252	231
CDS;ID=637288296;locus_tag=SPO0809;product=hypothetical protein	12	6	6	5	13	13
"CDS;ID=637288297;locus_tag=SPO0810;product=6-aminohexanoate-dimer hydrolase, puta	19	16	23	27	23	17
CDS;ID=637288298;locus_tag=SPO0811;product=peptidyl-tRNA hydrolase	45	40	33	37	42	36
CDS;ID=637288299;locus_tag=SPO0812;product=ribosomal protein L25	380	315	326	362	472	390
"CDS;ID=637288300;locus_tag=SPO0813;product=L-lactate dehydrogenase, putative"	31	26	25	14	17	12
CDS;ID=637288301;locus_tag=SPO0814;product=MaoC domain protein	10	8	4	5	10	7
CDS;ID=637288302;locus_tag=SPO0815;product=tryptophan synthase subunit alpha	87	88	80	146	109	126

CDS;ID=637288303;locus_tag=SPO0816;product=hypothetical protein	3	3	0	2	1	5
CDS;ID=637288304;locus_tag=SPO0817;product=GTP-binding protein YchF	57	61	61	104	98	103
CDS;ID=637288305;locus_tag=SPO0818;product=MAPEG family protein	3	6	3	14	8	9
CDS;ID=637288306;locus_tag=SPO0819;product=amidohydrolase domain protein	26	24	12	16	21	23
CDS;ID=637288307;locus_tag=SPO0820;product=CAIB/BAIF family protein	23	28	14	19	29	24
"CDS;ID=637288308;locus_tag=SPO0821;product=transcriptional regulator, MarR family'	18	11	7	8	13	7
"CDS;ID=637288309;locus_tag=SPO0822;product=branched-chain amino acid ABC transport	6	9	1	2	0	7
"CDS;ID=637288310;locus_tag=SPO0823;product=branched-chain amino acid ABC transport	3	1	5	2	4	5
"CDS;ID=637288311;locus_tag=SPO0824;product=branched-chain amino acid ABC transport	7	1	2	3	8	9
"CDS;ID=637288312;locus_tag=SPO0825;product=branched-chain amino acid ABC transport	29	25	17	14	22	24
CDS;ID=637288313;locus_tag=SPO0826;product=hypothetical protein	22	28	18	24	20	24
CDS;ID=637288314;locus_tag=SPO0827;product=hypothetical protein	30	34	18	28	24	29
CDS;ID=637288315;locus_tag=SPO0828;product=hypothetical protein	3	2	1	3	3	8
CDS;ID=637288316;locus_tag=SPO0829;product=hypothetical protein	11	5	5	5	5	4
"CDS;ID=637288317;locus_tag=SPO0830;product=xanthine dehydrogenase family protein,	9	15	12	10	25	10
"CDS;ID=637288318;locus_tag=SPO0831;product=xanthine dehydrogenase family protein,	5	16	10	13	6	8
"CDS;ID=637288319;locus_tag=SPO0832;product=transcriptional regulator, LysR family'	3	11	4	1	2	5
"CDS;ID=637288320;locus_tag=SPO0833;product=formate dehydrogenase, beta subunit"	121	74	75	97	123	114
"CDS;ID=637288321;locus_tag=SPO0834;product=formate dehydrogenase, alpha subunit"	54	54	45	60	67	58
"CDS;ID=637288322;locus_tag=SPO0835;product=cyclase, putative"	53	51	58	39	50	47
CDS;ID=637288323;locus_tag=SPO0836;product=hypothetical protein	32	27	23	22	29	27
CDS;ID=637288324;locus_tag=SPO0837;product=hypothetical protein	9	4	7	6	4	4
CDS;ID=637288325;locus_tag=SPO0838;product=bacterial sugar transferase	31	33	20	23	29	47
CDS;ID=637288326;locus_tag=SPO0839;product=exopolysaccharide biosynthesis domain pı	3	5	4	8	6	1
"CDS;ID=637288327;locus_tag=SPO0840;product=chain length determinant protein, putat	29	44	30	33	39	45
CDS;ID=637288328;locus_tag=SPO0841;product=polysaccharide biosynthesis protein	16	20	11	15	23	25
"CDS;ID=637288329;locus_tag=SPO0842;product=glycosyl transferase, group 1 family pı	45	26	28	37	31	49
"CDS;ID=637288330;locus_tag=SPO0843;product=glycosyl transferase, group 2 family pı	4	6	5	5	1	9
CDS;ID=637288331;locus_tag=SPO0844;product=hypothetical protein	40	31	20	16	55	35
CDS;ID=637288332;locus_tag=SPO0845;product=hypothetical protein	6	10	5	5	22	8
"CDS;ID=637288333;locus_tag=SPO0846;product=phosphopantetheinyl transferase PptA, ı	1	3	0	1	1	1
CDS;ID=637288334;locus_tag=SPO0847;product=non-ribosomal peptide synthetase	30	22	14	16	11	11
"CDS;ID=637288335;locus_tag=SPO0848;product=glycosyl transferase, group 2 family pı	15	9	5	7	6	14
CDS;ID=637288336;locus_tag=SPO0849;product=non-ribosomal peptide synthase	32	74	38	55	49	64
CDS;ID=637288337;locus_tag=SPO0850;product=hypothetical protein	9	13	10	13	15	10
"CDS;ID=637288338;locus_tag=SPO0851;product=glycosyl transferase, WecB/TagA/CpsF fa	19	9	5	9	6	12
"CDS;ID=637288339;locus_tag=SPO0852;product=oxidoreductase, Gfo/Idh/MocA family"	11	9	13	1	14	5
"CDS;ID=637288340;locus_tag=SPO0853;product=glycosyl transferase, group 2 family pı	2	1	5	2	6	0
"CDS;ID=637288341;locus_tag=SPO0854;product=glycosyl transferase, group 1 family pı	1	3	5	6	4	8
CDS;ID=637288342;locus_tag=SPO0855;product=xylulokinase	133	104	87	65	69	80
CDS;ID=637288343;locus_tag=SPO0856;product=xylose isomerase	23	22	26	19	40	28
CDS;ID=637288344;locus_tag=SPO0857;product=aldose 1-epimerase	14	12	8	5	8	5
"CDS;ID=637288345;locus_tag=SPO0858;product=methylamine utilization protein MauG, ı	15	12	4	4	12	16

CDS;ID=637288346;locus_tag=SPO0859;product=FG-GAP repeat domain protein	28	34	27	20	20	27
"CDS;ID=637288347;locus_tag=SPO0860;product=xylose repressor, putative"	31	32	32	23	19	32
"CDS;ID=637288348;locus_tag=SPO0861;product=xylose ABC transporter, periplasmic xy	192	174	129	137	142	184
"CDS;ID=637288349;locus_tag=SPO0862;product=xylose ABC transporter, permease prote	58	43	25	44	42	39
"CDS;ID=637288350;locus_tag=SPO0863;product=xylose ABC transporter, ATP-binding pro	63	72	53	43	80	74
"CDS;ID=637288351;locus_tag=SPO0864;product=glucokinase, putative"	28	22	21	16	23	24
"CDS;ID=637288352;locus_tag=SPO0865;product=oxidoreductase, Gfo/Idh/MocA family"	18	23	10	9	20	21
"CDS;ID=637288353;locus_tag=SPO0866;product=gamma-glutamyltranspeptidase, putative'	24	14	17	19	18	24
CDS;ID=637288354;locus_tag=SPO0867;product=hypothetical protein	68	50	47	67	60	65
"CDS;ID=637288355;locus_tag=SPO0868;product=outer membrane protein, OMP85 family"	42	35	28	27	21	58
CDS;ID=637288356;locus_tag=SPO0869;product=hypothetical protein	147	72	112	83	144	88
"CDS;ID=637288357;locus_tag=SPO0870;product=transcriptional regulator, LysR family'	18	10	8	20	15	18
CDS;ID=637288358;locus_tag=SPO0871;product=transthyretin family protein	115	64	90	91	147	41
CDS;ID=637288359;locus_tag=SPO0872;product=polysaccharide deacetylase family prote	102	44	63	65	112	83
CDS;ID=637288360;locus_tag=SPO0873;product=ureidoglycolate hydrolase	22	21	5	12	23	19
CDS;ID=637288361;locus_tag=SPO0874;product=xanthine/uracil permease family protein	24	32	20	12	17	26
"CDS;ID=637288362;locus_tag=SPO0875;product=glyceraldehyde-3-phosphate dehydrogena	18	23	7	26	27	15
CDS;ID=637288363;locus_tag=SPO0876;product=hypothetical protein	118	57	54	52	110	65
CDS;ID=637288364;locus_tag=SPO0877;product=hypothetical protein	27	21	20	26	35	30
CDS;ID=637288365;locus_tag=SPO0878;product=glycine cleavage system H protein	71	85	68	54	66	79
CDS;ID=637288366;locus_tag=SPO0879;product=acyl-CoA dehydrogenase family protein	105	83	61	51	85	80
CDS;ID=637288367;locus_tag=SPO0880;product=hypothetical protein	24	18	20	20	21	27
CDS;ID=637288368;locus_tag=SPO0881;product=hypothetical protein	87	68	64	85	50	96
CDS;ID=637288369;locus_tag=SPO0882;product=hypothetical protein	30	27	37	31	16	29
CDS;ID=637288370;locus_tag=SPO0883;product=hypothetical protein	87	109	75	61	59	99
CDS;ID=637288371;locus_tag=SPO0884;product=hypothetical protein	33	28	29	28	23	34
"CDS;ID=637288372;locus_tag=SPO0885;product=hydrolase, alpha/beta fold family"	23	29	18	13	16	24
"CDS;ID=637288373;locus_tag=SPO0886;product=chaperonin, 10 kDa"	170	130	145	174	323	187
CDS;ID=637288374;locus_tag=SPO0887;product=chaperonin GroEL	871	645	804	796	1317	788
"CDS;ID=637288375;locus_tag=SPO0888;product=acetyltransferase, GNAT family"	41	26	20	22	14	23
CDS;ID=637288376;locus_tag=SPO0889;product=hypothetical protein	5	11	3	7	14	13
"CDS;ID=637288377;locus_tag=SPO0890;product=lipase, putative"	22	20	12	14	16	20
"CDS;ID=637288378;locus_tag=SPO0891;product=alkylphosphonate utilization protein Ph	19	11	13	9	7	20
"CDS;ID=637288379;locus_tag=SPO0892;product=phosphoribosylaminoimidazole carboxylas	53	44	52	50	54	57
"CDS;ID=637288380;locus_tag=SPO0893;product=phosphoribosylaminoimidazole carboxylas	73	73	47	55	50	52
CDS;ID=637288381;locus_tag=SPO0894;product=hypothetical protein	14	16	12	18	11	11
"CDS;ID=637288382;locus_tag=SPO0895;product=heat shock protein, Hsp20 family"	69	50	53	66	80	109
CDS;ID=637288383;locus_tag=SPO0896;product=hypothetical protein	13	9	7	17	22	23
"CDS;ID=637288384;locus_tag=SPO0897;product=phenazine biosynthesis protein, PhzF fa	72	66	82	62	85	98
CDS;ID=637288385;locus_tag=SPO0898;product=hypothetical protein	27	23	23	33	25	24
CDS;ID=637288386;locus_tag=SPO0899;product=hypothetical protein	45	36	29	20	21	27
CDS;ID=637288387;locus_tag=SPO0900;product=binfunctional sulfate adenylyltransferas	153	158	114	166	159	203
CDS;ID=637288388;locus_tag=SPO0901;product=adenylate/guanylate cyclase	15	20	2	9	8	8

CDS;ID=637288389;locus_tag=SPO0902;product=hypothetical protein	1	8	2	11	8	8
CDS;ID=637288390;locus_tag=SPO0903;product=thioredoxin-disulfide reductase	71	54	81	72	93	64
"CDS;ID=637288391;locus_tag=SPO0904;product=leucine-responsive regulatory protein,	31	18	22	24	20	43
CDS;ID=637288392;locus_tag=SPO0905;product=hypothetical protein	11	7	3	8	5	6
CDS;ID=637288393;locus_tag=SPO0906;product=conserved hypothetical protein TIGR0072	22	17	8	12	6	18
CDS;ID=637288394;locus_tag=SPO0907;product=hypothetical protein	19	24	9	14	15	18
CDS;ID=637288395;locus_tag=SPO0908;product=prolipoprotein diacylglyceryl transfera	52	43	50	62	52	67
CDS;ID=637288396;locus_tag=SPO0909;product=hypothetical protein	21	20	8	12	14	17
CDS;ID=637288397;locus_tag=SPO0910;product=hypothetical protein	44	40	22	46	35	39
CDS;ID=637288398;locus_tag=SPO0911;product=pyrroline-5-carboxylate reductase	30	29	27	23	33	33
CDS;ID=637288399;locus_tag=SPO0912;product=chaperonin csaA	17	20	14	19	19	21
CDS;ID=637288400;locus_tag=SPO0913;product=D-isomer specific 2-hydroxyacid dehydro	25	21	31	19	19	14
CDS;ID=637288401;locus_tag=SPO0914;product=thymidine kinase	43	16	23	35	27	33
CDS;ID=637288402;locus_tag=SPO0915;product=hypothetical protein	78	82	52	86	56	77
CDS;ID=637288403;locus_tag=SPO0916;product=hypothetical protein	15	6	3	10	9	11
CDS;ID=637288404;locus_tag=SPO0917;product=glyoxalase family protein	12	14	12	9	9	10
CDS;ID=637288405;locus_tag=SPO0918;product=3-oxoacyl-(acyl carrier protein) syntha	20	17	18	24	43	10
"CDS;ID=637288406;locus_tag=SPO0919;product=transcriptional regulator, MarR family'	4	11	13	4	4	8
"CDS;ID=637288407;locus_tag=SPO0920;product=efflux transporter, RND family, MFP su	9	3	1	4	7	12
"CDS;ID=637288408;locus_tag=SPO0921;product=transporter, AcrB/AcrD/AcrF family"	15	9	15	12	26	24
CDS;ID=637288409;locus_tag=SPO0922;product=hypothetical protein	3	3	1	2	7	0
CDS;ID=637288410;locus_tag=SPO0923;product=carbamoyl-phosphate synthase large subu	157	161	146	191	203	168
CDS;ID=637288411;locus_tag=SPO0924;product=para-aminobenzoate synthase component I	26	33	30	45	33	53
CDS;ID=637288412;locus_tag=SPO0925;product=hypothetical protein	0	0	4	1	3	2
CDS;ID=637288413;locus_tag=SPO0926;product=aspartyl-tRNA synthetase	60	55	46	61	61	72
CDS;ID=637288414;locus_tag=SPO0927;product=hypothetical protein	4	3	6	1	2	5
CDS;ID=637288415;locus_tag=SPO0928;product=hypothetical protein	26	19	22	12	11	9
"CDS;ID=637288416;locus_tag=SPO0929;product=type II DNA modification methyltransfe	67	66	41	55	43	53
CDS;ID=637288417;locus_tag=SPO0930;product=hypothetical protein	0	0	0	0	0	0
CDS;ID=637288418;locus_tag=SPO0931;product=response regulator	1	0	0	0	0	2
CDS;ID=637288419;locus_tag=SPO0932;product=methylmalonyl-CoA epimerase	25	40	37	29	39	50
CDS;ID=637288420;locus_tag=SPO0933;product=hypothetical protein	10	17	12	15	12	9
CDS;ID=637288421;locus_tag=SPO0934;product=hypothetical protein	9	12	12	12	13	6
CDS;ID=637288422;locus_tag=SPO0935;product=nitroreductase family protein	32	19	23	42	27	31
CDS;ID=637288423;locus_tag=SPO0936;product=hypothetical protein	5	0	3	1	2	1
CDS;ID=637288424;locus_tag=SPO0937;product=hypothetical protein	10	3	7	12	4	10
CDS;ID=637288425;locus_tag=SPO0938;product=hypothetical protein	48	43	35	37	27	29
"CDS;ID=637288426;locus_tag=SPO0939;product=peptidase, M48 family"	54	44	34	34	39	43
CDS;ID=637288427;locus_tag=SPO0940;product=hypothetical protein	6	5	0	7	3	4
CDS;ID=637288428;locus_tag=SPO0941;product=hypothetical protein	27	10	26	35	32	21
CDS;ID=637288429;locus_tag=SPO0942;product=hypothetical protein	2	3	5	4	1	3
CDS;ID=637288430;locus_tag=SPO0943;product=hypothetical protein	8	7	10	11	6	14
"CDS;ID=637288431;locus_tag=SPO0944;product=RNA modification enzyme, MiaB-family"	63	37	39	54	61	71

CDS;ID=637288432;locus_tag=SPO0945;product=AsmA family protein	64	52	35	37	48	50
CDS;ID=637288433;locus_tag=SPO0946;product=phosphomannomutase/phosphoglucomutase	60	59	39	41	50	43
CDS;ID=637288434;locus_tag=SPO0947;product=2-dehydro-3-deoxyphosphooctonate aldolas	47	52	29	42	57	34
"CDS;ID=637288435;locus_tag=SPO0948;product=capsular polysaccharide export inner-me	47	49	43	30	43	38
CDS;ID=637288436;locus_tag=SPO0949;product=capsular polysaccharide export ATP-bindi	51	51	36	33	47	51
"CDS;ID=637288437;locus_tag=SPO0950;product=uracil-DNA glycosylase, putative"	10	5	10	5	10	12
CDS;ID=637288438;locus_tag=SPO0951;product=hypothetical protein	8	3	7	8	7	17
CDS;ID=637288439;locus_tag=SPO0952;product=hypothetical protein	8	12	6	7	10	12
CDS;ID=637288440;locus_tag=SPO0953;product=hypothetical protein	22	21	23	16	24	21
CDS;ID=637288441;locus_tag=SPO0954;product=hypothetical protein	15	17	17	19	12	17
CDS;ID=637288442;locus_tag=SPO0955;product=amidohydrolase domain protein	15	19	20	13	13	20
CDS;ID=637288443;locus_tag=SPO0956;product=hypothetical protein	18	22	15	24	18	17
"CDS;ID=637288444;locus_tag=SPO0957;product=ABC transporter, permease protein"	12	19	10	24	17	29
CDS;ID=637288445;locus_tag=SPO0958;product=hypothetical protein	9	3	7	5	6	9
CDS;ID=637288446;locus_tag=SPO0959;product=hypothetical protein	6	10	5	4	6	9
"CDS;ID=637288447;locus_tag=SPO0960;product=transcriptional regulator, MerR family'	9	7	10	6	9	7
CDS;ID=637288448;locus_tag=SPO0961;product=hypothetical protein	17	4	4	6	10	6
CDS;ID=637288449;locus_tag=SPO0962;product=acetylornithine aminotransferase	22	30	20	22	39	26
CDS;ID=637288450;locus_tag=SPO0963;product=ornithine carbamoyltransferase	12	27	11	18	24	22
CDS;ID=637288451;locus_tag=SPO0964;product=N-acetyl-gamma-glutamyl-phosphate reduct	10	16	11	11	17	9
"CDS;ID=637288452;locus_tag=SPO0965;product=acetyltransferase, GNAT family"	6	4	3	5	3	5
CDS;ID=637288453;locus_tag=SPO0966;product=2-dehydropantoate 2-reductase	7	7	14	9	10	17
CDS;ID=637288454;locus_tag=SPO0967;product=phosphate transporter family protein	34	30	35	39	49	53
"CDS;ID=637288455;locus_tag=SPO0968;product=hydrolase, NUDIX family"	34	27	29	26	20	34
CDS;ID=640735055;locus_tag=SPO0969	29	12	7	14	7	17
CDS;ID=637288456;locus_tag=SPO0970;product=aquaporin Z	2	3	1	5	7	2
"CDS;ID=637288457;locus_tag=SPO0971;product=oxidoreductase, GMC family"	44	49	25	26	23	45
CDS;ID=637288458;locus_tag=SPO0972;product=ATP-dependent helicase HrpB	24	25	23	18	37	25
CDS;ID=637288459;locus_tag=SPO0973;product=arginine/ornithine transport system ATPa	18	25	26	34	36	50
CDS;ID=637288460;locus_tag=SPO0974;product=ribosomal protein L28	90	85	63	110	101	86
CDS;ID=637288461;locus_tag=SPO0975;product=hypothetical protein	37	34	24	44	54	43
CDS;ID=637288462;locus_tag=SPO0976;product=hypothetical protein	203	202	165	204	152	158
CDS;ID=637288463;locus_tag=SPO0977;product=ornithine cyclodeaminase/mu-crystallin f	10	5	5	3	2	2
CDS;ID=637288464;locus_tag=SPO0978;product=NAD-dependent deacetylase	32	25	15	17	13	19
CDS;ID=637288465;locus_tag=SPO0979;product=low molecular weight phosphotyrosine pro	6	4	6	3	4	5
"CDS;ID=637288466;locus_tag=SPO0980;product=transcriptional regulator, LysR family'	14	12	7	11	6	12
CDS;ID=637288467;locus_tag=SPO0981;product=histidinol dehydrogenase	6	5	3	12	6	14
CDS;ID=637288468;locus_tag=SPO0982;product=hypothetical protein	33	18	16	10	15	14
CDS;ID=637288469;locus_tag=SPO0983;product=GTP-binding protein LepA	109	78	75	96	135	96
"CDS;ID=637288470;locus_tag=SPO0984;product=zinc ABC transporter, permease protein'	17	9	9	7	14	18
"CDS;ID=637288471;locus_tag=SPO0985;product=zinc ABC transporter, ATP-binding prote	13	2	9	10	13	25
CDS;ID=637288472;locus_tag=SPO0986;product=zinc uptake regulation protein	6	2	3	9	13	13
"CDS;ID=637288473;locus_tag=SPO0987;product=zinc ABC transporter, periplasmic zinc-	9	6	11	8	11	7

CDS;ID=637288474;locus_tag=SPO0988;product=YeeE/YedE family protein	22	19	12	28	11	16
CDS;ID=637288475;locus_tag=SPO0989;product=transcriptional regulator SoxR	19	20	20	26	18	35
CDS;ID=637288476;locus_tag=SPO0990;product=regulatory protein SoxS	29	29	18	23	21	14
CDS;ID=637288477;locus_tag=SPO0991;product=sulfur oxidation V protein	22	21	13	11	10	11
CDS;ID=637288478;locus_tag=SPO0992;product=thioredoxin SoxW	32	18	23	32	24	31
CDS;ID=637288479;locus_tag=SPO0993;product=monoheme cytochrome c SoxX	20	17	10	6	13	15
CDS;ID=637288480;locus_tag=SPO0994;product=sulfur oxidation protein SoxY	10	16	19	10	13	13
CDS;ID=637288481;locus_tag=SPO0995;product=sulfur oxidation Z protein	19	16	24	10	26	7
CDS;ID=637288482;locus_tag=SPO0996;product=diheme cytochrome c SoxA	12	11	9	10	11	4
CDS;ID=637288483;locus_tag=SPO0997;product=sulfur oxidation B protein	12	16	16	8	10	11
CDS;ID=637288484;locus_tag=SPO0998;product=sulfur oxidation molybdopterin C protein	10	5	8	5	8	14
CDS;ID=637288485;locus_tag=SPO0999;product=diheme cytochrome c SoxD	20	21	13	12	14	14
CDS;ID=637288486;locus_tag=SPO1000;product=diheme cytochrome c SoxE	74	97	61	81	40	71
CDS;ID=637288487;locus_tag=SPO1001;product=sulfur oxidation F protein	15	5	7	14	18	14
"CDS;ID=637288488;locus_tag=SPO1002;product=transcriptional regulator, AraC family"	11	6	9	8	5	9
"CDS;ID=637288489;locus_tag=SPO1003;product=ATP-dependent Clp protease, proteolytic	262	254	194	203	282	228
CDS;ID=637288490;locus_tag=SPO1004;product=ATP-dependent protease ATP-binding subun	464	402	369	351	468	413
CDS;ID=637288491;locus_tag=SPO1005;product=endoribonuclease L-PSP family protein	111	96	85	102	135	115
CDS;ID=637288492;locus_tag=SPO1006;product=NADH-ubiquinone oxidoreductase	159	132	118	153	130	150
CDS;ID=637288493;locus_tag=SPO1007;product=hypothetical protein	68	48	27	43	38	64
CDS;ID=637288494;locus_tag=SPO1008;product=hypothetical protein	25	39	12	22	30	42
CDS;ID=637288495;locus_tag=SPO1009;product=leucyl/phenylalanyl-tRNA--protein transf	6	10	4	13	11	12
CDS;ID=637288496;locus_tag=SPO1010;product=acetyl-CoA carboxylase	69	48	50	83	68	85
"CDS;ID=637288497;locus_tag=SPO1011;product=acetyl-CoA carboxylase, biotin carboxyl	76	72	57	41	60	78
"CDS;ID=637288498;locus_tag=SPO1012;product=transcriptional regulator, LuxR family"	9	9	13	8	13	17
CDS;ID=637288499;locus_tag=SPO1013;product=acetyl-CoA acetyltransferase	0	3	1	2	1	4
CDS;ID=637288500;locus_tag=SPO1014;product=AMP-binding enzyme	11	3	11	4	6	8
"CDS;ID=637288501;locus_tag=SPO1015;product=3-hydroxybutyrate dehydrogenase, putati	4	3	5	1	5	3
CDS;ID=637288502;locus_tag=SPO1016;product=hypothetical protein	7	2	10	5	5	2
"CDS;ID=637288503;locus_tag=SPO1017;product=branched-chain amino acid ABC transport	10	5	4	4	4	6
"CDS;ID=637288504;locus_tag=SPO1018;product=branched-chain amino acid ABC transport	2	3	2	0	4	1
"CDS;ID=637288505;locus_tag=SPO1019;product=branched-chain amino acid ABC transport	5	4	3	1	2	8
"CDS;ID=637288506;locus_tag=SPO1020;product=branched-chain amino acid ABC transport	9	3	4	5	4	0
"CDS;ID=637288507;locus_tag=SPO1021;product=branched-chain amino acid ABC transport	1	4	2	4	0	3
"CDS;ID=637288508;locus_tag=SPO1022;product=transcriptional regulator, putative"	33	23	31	40	49	15
CDS;ID=637288509;locus_tag=SPO1023;product=response regulator	16	14	14	3	3	12
CDS;ID=637288510;locus_tag=SPO1024;product=hypothetical protein	1	3	0	1	1	2
CDS;ID=637288511;locus_tag=SPO1025;product=sensor histidine kinase	18	19	13	15	16	23
CDS;ID=637288512;locus_tag=SPO1026;product=hypothetical protein	5	1	0	6	2	5
CDS;ID=637288513;locus_tag=SPO1027;product=conserved hypothetical protein TIGR0124	42	27	17	49	27	47
CDS;ID=637288514;locus_tag=SPO1028;product=YeeE/YedE family protein	8	3	0	5	3	3
CDS;ID=637288515;locus_tag=SPO1029;product=YeeE/YedE family protein	7	7	3	6	3	4
CDS;ID=637288516;locus_tag=SPO1030;product=metallo-beta-lactamase family protein	15	11	17	11	12	13

CDS;ID=637288517;locus_tag=SPO1031;product=hypothetical protein	96	36	70	87	97	78
CDS;ID=637288518;locus_tag=SPO1032;product=hypothetical protein	8	4	7	8	8	7
CDS;ID=637288519;locus_tag=SPO1033;product=hypothetical protein	70	47	41	85	83	58
CDS;ID=637288520;locus_tag=SPO1034;product=hypothetical protein	130	120	139	119	234	72
tRNA;ID=640698521;locus_tag=SPO_tRNA-Thr-2	1117	536	383	562	83	433
"CDS;ID=637288522;locus_tag=SPO1036;product=site-specific recombinase, phage integr	58	62	36	54	56	42
CDS;ID=637288523;locus_tag=SPO1037;product=hypothetical protein	228	176	159	152	173	197
CDS;ID=637288524;locus_tag=SPO1038;product=hypothetical protein	21	14	17	11	19	10
CDS;ID=637288525;locus_tag=SPO1039;product=hypothetical protein	4	3	5	3	3	7
CDS;ID=637288526;locus_tag=SPO1040;product=hypothetical protein	0	0	4	1	2	0
CDS;ID=637288527;locus_tag=SPO1041;product=hypothetical protein	3	2	4	8	1	1
CDS;ID=637288528;locus_tag=SPO1042;product=hypothetical protein	1	3	3	3	2	1
CDS;ID=637288529;locus_tag=SPO1043;product=hypothetical protein	0	0	0	0	0	0
CDS;ID=637288530;locus_tag=SPO1044;product=hypothetical protein	1	3	2	2	2	2
CDS;ID=637288531;locus_tag=SPO1045;product=hypothetical protein	76	84	59	64	80	93
CDS;ID=637288532;locus_tag=SPO1046;product=hypothetical protein	113	131	123	117	114	150
"CDS;ID=637288533;locus_tag=SPO1047;product=RNA-dependent DNA polymerase, putative"	167	171	172	174	155	195
CDS;ID=637288534;locus_tag=SPO1048;product=hypothetical protein	102	109	68	78	65	88
"CDS;ID=637288535;locus_tag=SPO1049;product=DNA methylase, C-5 cytosine-specific fa	172	177	161	149	119	156
"CDS;ID=637288536;locus_tag=SPO1050;product=site-specific recombinase, phage integr	5	12	10	12	5	15
CDS;ID=637288537;locus_tag=SPO1051;product=hypothetical protein	1	1	1	0	1	2
CDS;ID=637288538;locus_tag=SPO1052;product=hypothetical protein	1	1	0	1	0	0
CDS;ID=637288539;locus_tag=SPO1053;product=hypothetical protein	15	1	8	8	10	9
CDS;ID=637288540;locus_tag=SPO1054;product=hypothetical protein	5	6	2	0	4	0
CDS;ID=637288541;locus_tag=SPO1055;product=hypothetical protein	6	1	2	2	0	3
CDS;ID=637288542;locus_tag=SPO1056;product=hypothetical protein	1	1	1	0	0	1
"CDS;ID=637288543;locus_tag=SPO1057;product=transcriptional regulator, AraC family"	6	6	1	7	3	6
"CDS;ID=637288544;locus_tag=SPO1058;product=acyltransferase, HtrB family"	24	31	21	31	19	53
CDS;ID=637288545;locus_tag=SPO1059;product=serine/threonine protein kinase	70	85	53	57	73	84
CDS;ID=637288546;locus_tag=SPO1060;product=hypothetical protein	31	39	28	21	71	34
CDS;ID=637288547;locus_tag=SPO1061;product=LysM domain protein	39	37	43	35	49	50
CDS;ID=637288548;locus_tag=SPO1062;product=LysM domain protein	77	73	49	64	114	68
CDS;ID=637288549;locus_tag=SPO1063;product=hypothetical protein	8	8	6	10	15	9
"CDS;ID=637288550;locus_tag=SPO1064;product=serine/threonine protein phosphatase, p	15	8	9	8	13	10
CDS;ID=637288551;locus_tag=SPO1065;product=hypothetical protein	14	21	9	11	16	8
CDS;ID=637288552;locus_tag=SPO1066;product=hypothetical protein	51	37	20	44	37	37
CDS;ID=637288553;locus_tag=SPO1067;product=hypothetical protein	41	50	39	47	60	45
CDS;ID=637288554;locus_tag=SPO1068;product=hypothetical protein	33	26	20	28	25	35
CDS;ID=637288555;locus_tag=SPO1069;product=hypothetical protein	58	62	71	55	75	80
CDS;ID=637288556;locus_tag=SPO1070;product=hypothetical protein	21	10	20	7	13	27
CDS;ID=637288557;locus_tag=SPO1071;product=glutamine amidotransferase	6	13	12	11	18	17
CDS;ID=637288558;locus_tag=SPO1072;product=hypothetical protein	92	80	64	111	101	110
CDS;ID=637288559;locus_tag=SPO1073;product=bordetella uptake gene family protein	2	1	0	0	1	3

CDS;ID=637288560;locus_tag=SPO1074;product=hypothetical protein	0	1	1	0	0	0
CDS;ID=637288561;locus_tag=SPO1075;product=hypothetical protein	2	1	1	0	2	0
CDS;ID=637288562;locus_tag=SPO1076;product=trkA-C domain protein	26	21	25	20	18	23
CDS;ID=637288563;locus_tag=SPO1077;product=nitroreductase family protein	28	18	15	19	14	26
CDS;ID=637288564;locus_tag=SPO1078;product=chromosome replication initiation inhib:	0	2	0	2	0	1
"CDS;ID=637288565;locus_tag=SPO1079;product=L-lysine exporter, putative"	2	0	0	0	0	1
CDS;ID=637288566;locus_tag=SPO1080;product=hypothetical protein	31	20	15	13	20	21
CDS;ID=637288567;locus_tag=SPO1081;product=hypothetical protein	13	8	6	5	5	11
CDS;ID=637288568;locus_tag=SPO1082;product=HTH-type transcriptional regulator BetI	102	101	85	75	59	61
CDS;ID=637288569;locus_tag=SPO1083;product=choline sulfatase	46	49	31	14	32	28
CDS;ID=637288570;locus_tag=SPO1084;product=hypothetical protein	3	4	1	1	3	3
CDS;ID=637288571;locus_tag=SPO1085;product=hypothetical protein	21	17	6	17	18	19
"CDS;ID=637288572;locus_tag=SPO1086;product=transcriptional regulator, TetR family'	20	22	18	27	20	27
CDS;ID=637288573;locus_tag=SPO1087;product=hypothetical protein	14	13	2	5	9	6
CDS;ID=637288574;locus_tag=SPO1088;product=choline dehydrogenase	19	17	12	5	9	10
CDS;ID=637288575;locus_tag=SPO1089;product=hypothetical protein	660	452	614	832	1330	519
"CDS;ID=637288576;locus_tag=SPO1090;product=DNA-binding protein, putative"	57	38	35	62	97	72
CDS;ID=637288577;locus_tag=SPO1091;product=YCII-related domain protein	1	0	0	0	0	0
"CDS;ID=637288578;locus_tag=SPO1092;product=transcriptional regulator, LysR family'	3	6	3	1	5	5
"CDS;ID=637288579;locus_tag=SPO1093;product=drug resistance transporter, Bcr/CflA f	8	11	3	3	3	11
"CDS;ID=637288580;locus_tag=SPO1094;product=propionyl-CoA carboxylase, beta subunit	45	34	26	35	47	58
CDS;ID=637288581;locus_tag=SPO1095;product=hypothetical protein	8	1	5	3	3	6
"CDS;ID=637288582;locus_tag=SPO1096;product=lipoprotein, putative"	131	169	97	159	114	95
CDS;ID=637288583;locus_tag=SPO1097;product=hypothetical protein	4	0	3	2	3	0
CDS;ID=637288584;locus_tag=SPO1098;product=hypothetical protein	8	5	7	11	10	7
CDS;ID=637288585;locus_tag=SPO1099;product=hypothetical protein	9	3	2	5	3	3
"CDS;ID=637288586;locus_tag=SPO1100;product=lipoprotein, putative"	107	76	60	80	47	46
"CDS;ID=637288587;locus_tag=SPO1101;product=propionyl-CoA carboxylase, alpha subun:	58	50	30	36	55	59
"CDS;ID=637288588;locus_tag=SPO1102;product=lipoprotein, putative"	13	17	7	15	25	17
CDS;ID=637288589;locus_tag=SPO1103;product=hypothetical protein	66	65	51	77	88	87
CDS;ID=637288590;locus_tag=SPO1104;product=hypothetical protein	30	30	17	14	29	24
CDS;ID=637288591;locus_tag=SPO1105;product=methylmalonyl-CoA mutase	52	58	34	46	75	71
CDS;ID=637288592;locus_tag=SPO1106;product=hypothetical protein	11	13	7	7	3	8
"CDS;ID=637288593;locus_tag=SPO1107;product=phosphinothricin N-acetyltransferase, p	2	2	0	2	0	2
"CDS;ID=637288594;locus_tag=SPO1108;product=DnaJ-like protein DjlA, putative"	17	10	11	18	15	27
CDS;ID=637288595;locus_tag=SPO1109;product=endonuclease/exonuclease/phosphatase far	8	15	8	0	6	4
"CDS;ID=637288596;locus_tag=SPO1110;product=transcriptional regulator, LysR family'	11	11	9	14	14	14
CDS;ID=637288597;locus_tag=SPO1111;product=hypothetical protein	39	27	12	3	10	3
"CDS;ID=637288598;locus_tag=SPO1112;product=TRAP transporter, periplasmic protein"	18	29	7	2	7	6
"CDS;ID=637288599;locus_tag=SPO1113;product=TRAP transporter, transmembrane protein	12	9	0	3	0	1
"CDS;ID=637288600;locus_tag=SPO1114;product=TRAP dicarboxylate transporter, DctM su	13	10	2	3	3	4
CDS;ID=637288601;locus_tag=SPO1115;product=hypothetical protein	4	4	1	2	1	0
CDS;ID=637288602;locus_tag=SPO1116;product=hypothetical protein	152	109	100	123	139	158

"CDS;ID=637288603;locus_tag=SPO1117;product=phosphatase, Ppx/GppA family"	83	74	72	52	68	68
CDS;ID=637288604;locus_tag=SPO1118;product=hypothetical protein	26	16	17	21	10	15
CDS;ID=637288605;locus_tag=SPO1119;product=membrane protein	39	27	21	24	42	39
CDS;ID=637288606;locus_tag=SPO1120;product=hypothetical protein	3	3	4	2	3	1
CDS;ID=637288607;locus_tag=SPO1121;product=prolyl-tRNA synthetase	137	118	102	147	179	166
CDS;ID=637288608;locus_tag=SPO1122;product=hypothetical protein	17	17	11	16	17	28
CDS;ID=637288609;locus_tag=SPO1123;product=lipoprotein releasing system transmembra	39	39	30	23	31	42
CDS;ID=637288610;locus_tag=SPO1124;product=lipoprotein releasing system ATP-binding	76	87	58	54	62	84
CDS;ID=637288611;locus_tag=SPO1125;product=hypothetical protein	9	11	8	16	9	7
CDS;ID=637288612;locus_tag=SPO1126;product=cytochrome c family protein	28	10	31	18	16	18
CDS;ID=637288613;locus_tag=SPO1127;product=cytochrome c family protein	22	11	24	17	14	23
CDS;ID=637288614;locus_tag=SPO1128;product=hypothetical protein	4	5	3	2	6	1
CDS;ID=637288615;locus_tag=SPO1129;product=hypothetical protein	11	13	5	11	8	11
CDS;ID=637288616;locus_tag=SPO1130;product=thioesterase family protein	10	10	6	9	12	5
"CDS;ID=637288617;locus_tag=SPO1131;product=glycine betaine/proline ABC transporter	126	110	106	70	128	76
"CDS;ID=637288618;locus_tag=SPO1132;product=glycine betaine/proline ABC transporter	28	25	25	20	24	25
"CDS;ID=637288619;locus_tag=SPO1133;product=glycine betaine/proline ABC transporter	88	60	51	34	37	47
CDS;ID=637288620;locus_tag=SPO1134;product=NnrU family protein	1	2	2	6	3	4
CDS;ID=637288621;locus_tag=SPO1135;product=hypothetical protein	45	24	34	41	42	46
CDS;ID=637288622;locus_tag=SPO1136;product=hypothetical protein	7	3	4	9	4	4
CDS;ID=637288623;locus_tag=SPO1137;product=succinate-semialdehyde dehydrogenase	11	12	8	4	9	2
"CDS;ID=637288624;locus_tag=SPO1138;product=transcriptional regulator, AsnC family"	0	0	0	3	1	1
CDS;ID=637288625;locus_tag=SPO1139;product=succinylglutamate desuccinylase/aspartoa	0	2	1	0	3	2
"CDS;ID=637288626;locus_tag=SPO1140;product=peptidase, putative"	5	8	2	11	7	3
CDS;ID=637288627;locus_tag=SPO1141;product=ornithine cyclodeaminase	6	9	4	6	6	2
CDS;ID=637288628;locus_tag=SPO1142;product=threonine dehydratase	4	4	0	0	7	2
CDS;ID=637288629;locus_tag=SPO1143;product=Asp/Glu/Hydantoin racemase family protei	0	0	0	1	1	1
CDS;ID=637288630;locus_tag=SPO1144;product=universal stress protein family protein	1	0	1	0	0	0
"CDS;ID=637288631;locus_tag=SPO1145;product=TRAP dicarboxylate transporter, DctM su	5	5	2	4	1	2
"CDS;ID=637288632;locus_tag=SPO1146;product=TRAP dicarboxylate transporter, DctQ su	1	3	1	3	0	2
"CDS;ID=637288633;locus_tag=SPO1147;product=TRAP dicarboxylate transporter, DctP su	14	12	6	8	11	10
"CDS;ID=637288634;locus_tag=SPO1148;product=transcriptional regulator, GntR family"	19	22	21	13	10	8
tRNA;ID=640698522;locus_tag=SPO_tRNA-Pseudo-2	166	99	61	40	11	65
"CDS;ID=637288636;locus_tag=SPO1150;product=RNA methyltransferase, TrmH family, gro	15	20	20	19	24	19
CDS;ID=637288637;locus_tag=SPO1151;product=hypothetical protein	36	21	51	40	37	45
"CDS;ID=637288638;locus_tag=SPO1152;product=twin-arginine translocation pathway sig	5	16	12	7	11	5
CDS;ID=637288639;locus_tag=SPO1153;product=CoA-binding domain protein	22	9	18	10	18	20
"CDS;ID=637288640;locus_tag=SPO1154;product=lipoprotein, putative"	20	21	17	23	18	27
CDS;ID=637288641;locus_tag=SPO1155;product=phosphoribosyl-ATP pyrophosphatase	3	5	3	4	1	2
CDS;ID=637288642;locus_tag=SPO1156;product=imidazole glycerol phosphate synthase su	6	3	12	11	8	10
CDS;ID=637288643;locus_tag=SPO1157;product=hypothetical protein	7	5	0	5	5	5
CDS;ID=637288644;locus_tag=SPO1158;product=1-(5-phosphoribosyl)-5-[(5- phosphoribos	13	12	12	14	22	24
CDS;ID=637288645;locus_tag=SPO1159;product=hypothetical protein	47	35	39	38	28	36

CDS;ID=637288646;locus_tag=SPO1160;product=hypothetical protein	6	4	1	3	2	4
"CDS;ID=637288647;locus_tag=SPO1161;product=transcriptional regulator, AraC family'	16	11	9	12	24	29
CDS;ID=637288648;locus_tag=SPO1162;product=imidazole glycerol phosphate synthase su	26	21	15	17	22	39
CDS;ID=637288649;locus_tag=SPO1163;product=imidazoleglycerol-phosphate dehydratase	35	19	21	34	28	28
CDS;ID=637288650;locus_tag=SPO1164;product=hypothetical protein	36	40	38	34	38	36
CDS;ID=637288651;locus_tag=SPO1165;product=hypothetical protein	24	13	5	13	13	14
"CDS;ID=637288652;locus_tag=SPO1166;product=aminotransferase, class III family"	21	36	26	15	44	26
tRNA;ID=640698523;locus_tag=SPO_tRNA-Gly-2	848	394	222	204	27	257
CDS;ID=637288654;locus_tag=SPO1168;product=hypothetical protein	7	3	4	8	5	1
tRNA;ID=640698524;locus_tag=SPO_tRNA-Gly-3	1213	614	427	507	250	497
tRNA;ID=640698525;locus_tag=SPO_tRNA-Gly-4	1169	670	504	632	296	700
CDS;ID=637288657;locus_tag=SPO1171;product=pyruvate carboxylase	305	290	261	227	229	276
CDS;ID=637288658;locus_tag=SPO1172;product=FMN-dependent alpha-hydroxy acid dehydro	50	65	35	52	44	53
"CDS;ID=637288659;locus_tag=SPO1173;product=diguanylate cyclase, putative"	6	2	3	3	3	4
"CDS;ID=637288660;locus_tag=SPO1174;product=DNA helicase II, putative"	119	97	88	77	87	119
CDS;ID=637288661;locus_tag=SPO1175;product=hypothetical protein	12	10	7	11	13	5
CDS;ID=637288662;locus_tag=SPO1176;product=Ser/Thr protein phosphatase family prote	13	13	14	12	14	10
CDS;ID=637288663;locus_tag=SPO1177;product=hypothetical protein	221	143	108	106	93	96
"CDS;ID=637288664;locus_tag=SPO1178;product=MraZ, putative"	30	29	17	41	31	35
CDS;ID=637288665;locus_tag=SPO1179;product=S-adenosyl-methyltransferase MraW	40	33	37	27	32	36
CDS;ID=637288666;locus_tag=SPO1180;product=hypothetical protein	15	13	11	21	17	15
CDS;ID=637288667;locus_tag=SPO1181;product=penicillin-binding protein	95	74	82	67	71	100
"CDS;ID=637288668;locus_tag=SPO1182;product=UDP-N-acetylmuramoylalanyl-D-glutamate-	35	26	25	34	33	47
CDS;ID=637288669;locus_tag=SPO1183;product=UDP-N-acetylmuramoyl-tripeptide--D-alany	28	29	23	28	28	23
CDS;ID=637288670;locus_tag=SPO1184;product=phospho-N-acetylmuramoyl-pentapeptide- t	11	24	18	31	20	17
CDS;ID=637288671;locus_tag=SPO1185;product=hypothetical protein	37	44	31	34	51	37
CDS;ID=637288672;locus_tag=SPO1186;product=TPR domain protein	94	88	70	74	85	78
CDS;ID=637288673;locus_tag=SPO1187;product=UDP-N-acetylmuramoylalanine--D-glutamate	19	18	23	36	32	31
CDS;ID=637288674;locus_tag=SPO1188;product=conserved hypothetical protein TIGR00275	10	9	12	10	17	9
CDS;ID=637288675;locus_tag=SPO1189;product=hypothetical protein	2	2	1	3	0	2
CDS;ID=637288676;locus_tag=SPO1190;product=transcriptional regulator family protein	3	2	2	4	0	11
CDS;ID=637288677;locus_tag=SPO1191;product=glutathione S-transferase	3	5	4	1	3	6
CDS;ID=637288678;locus_tag=SPO1192;product=hypothetical protein	4	1	1	2	0	2
CDS;ID=637288679;locus_tag=SPO1193;product=4-oxalocrotonate tautomerase family prot	5	1	1	0	1	0
CDS;ID=637288680;locus_tag=SPO1194;product=cell division protein FtsW	103	89	97	84	90	106
CDS;ID=637288681;locus_tag=SPO1195;product=N-acetylglucosaminyl transferase	95	92	76	96	123	59
CDS;ID=637288682;locus_tag=SPO1196;product=UDP-N-acetylmuramate--L-alanine ligase	167	180	147	163	158	158
CDS;ID=637288683;locus_tag=SPO1197;product=amino acid permease	74	39	36	40	33	28
CDS;ID=637288684;locus_tag=SPO1198;product=hypothetical protein	5	5	4	3	3	6
CDS;ID=637288685;locus_tag=SPO1199;product=hypothetical protein	4	6	7	7	9	5
CDS;ID=637288686;locus_tag=SPO1200;product=UDP-N-acetylenolpyruvoylglucosamine redu	160	139	153	117	81	170
CDS;ID=637288687;locus_tag=SPO1201;product=D-alanine--D-alanine ligase	104	100	66	87	73	92
CDS;ID=637288688;locus_tag=SPO1202;product=cell division protein ftsQ	106	91	81	90	127	134

CDS;ID=637288689;locus_tag=SPO1203;product=cell division protein FtsA	78	89	93	81	102	98
CDS;ID=637288690;locus_tag=SPO1204;product=cell division protein FtsZ	393	364	304	291	462	297
CDS;ID=637288691;locus_tag=SPO1205;product=UDP-3-0-acyl N-acetylglucosamine deacety	100	74	83	62	60	67
"CDS;ID=637288692;locus_tag=SPO1206;product=competence lipoprotein ComL, putative"	99	93	97	120	112	123
CDS;ID=637288693;locus_tag=SPO1207;product=DNA repair protein RecN	92	78	85	71	59	84
CDS;ID=637288694;locus_tag=SPO1208;product=voltage-gated chloride channel family pı	23	24	31	38	19	37
CDS;ID=637288695;locus_tag=SPO1209;product=MlrC domain protein	50	29	28	27	26	29
"CDS;ID=637288696;locus_tag=SPO1210;product=oligopeptide ABC transporter, periplasn	36	43	28	27	29	42
"CDS;ID=637288697;locus_tag=SPO1211;product=oligopeptide ABC transporter, permease	11	4	6	5	9	10
"CDS;ID=637288698;locus_tag=SPO1212;product=oligopeptide ABC transporter, permease	9	16	14	8	5	4
"CDS;ID=637288699;locus_tag=SPO1213;product=oligopeptide ABC transporter, ATP-bindi	15	17	15	10	23	12
CDS;ID=637288700;locus_tag=SPO1214;product=hippurate hydrolase	7	8	8	9	6	11
CDS;ID=637288701;locus_tag=SPO1215;product=carboxypeptidase	17	4	4	12	5	9
CDS;ID=637288702;locus_tag=SPO1216;product=hypothetical protein	3	3	8	3	2	5
"CDS;ID=637288703;locus_tag=SPO1217;product=DNA-binding protein, putative"	6	4	10	6	1	14
"CDS;ID=637288704;locus_tag=SPO1218;product=site-specific recombinase, phage integı	11	19	19	20	16	13
"CDS;ID=637288705;locus_tag=SPO1219;product=site-specific recombinase, phage integı	27	26	23	29	24	23
CDS;ID=637288706;locus_tag=SPO1220;product=hypothetical protein	38	44	41	49	36	45
CDS;ID=637288707;locus_tag=SPO1221;product=hypothetical protein	157	123	132	162	161	151
"CDS;ID=637288708;locus_tag=SPO1222;product=site-specific recombinase, resolvase fa	13	9	1	11	3	19
CDS;ID=637288709;locus_tag=SPO1223;product=hypothetical protein	3	4	4	3	3	6
CDS;ID=637288710;locus_tag=SPO1224;product=hypothetical protein	1	3	0	2	5	2
CDS;ID=637288711;locus_tag=SPO1225;product=hypothetical protein	2	1	0	1	0	1
"CDS;ID=637288712;locus_tag=SPO1226;product=lipoprotein, putative"	41	33	37	21	21	26
CDS;ID=637288713;locus_tag=SPO1227;product=hypothetical protein	5	9	17	12	10	11
CDS;ID=637288714;locus_tag=SPO1228;product=hypothetical protein	8	5	10	10	2	5
CDS;ID=637288715;locus_tag=SPO1229;product=hypothetical protein	4	8	4	9	7	6
CDS;ID=637288716;locus_tag=SPO1230;product=hypothetical protein	19	28	17	7	21	18
CDS;ID=637288717;locus_tag=SPO1231;product=hypothetical protein	6	5	5	9	7	6
"CDS;ID=637288718;locus_tag=SPO1232;product=DNA-binding protein, H-NS family"	3	1	1	1	1	1
CDS;ID=637288719;locus_tag=SPO1233;product=hypothetical protein	94	89	81	94	86	126
CDS;ID=637288720;locus_tag=SPO1234;product=hypothetical protein	104	118	87	112	128	150
CDS;ID=637288721;locus_tag=SPO1235;product=hypothetical protein	237	234	210	193	257	257
CDS;ID=637288722;locus_tag=SPO1236;product=hypothetical protein	51	58	82	73	67	57
CDS;ID=637288723;locus_tag=SPO1237;product=hypothetical protein	43	39	39	34	51	37
tRNA;ID=640698526;locus_tag=SPO_tRNA-Cys-2	332	246	203	318	220	242
CDS;ID=637288725;locus_tag=SPO1239;product=protein-L-isoaspartate O-methyltransfera	112	112	103	108	121	150
"CDS;ID=637288726;locus_tag=SPO1240;product=type I secretion outer membrane proteir	126	138	125	109	115	104
CDS;ID=637288727;locus_tag=SPO1241;product=hypothetical protein	212	181	138	144	181	156
CDS;ID=637288728;locus_tag=SPO1242;product=cobyric acid synthase	33	24	14	32	23	34
CDS;ID=637288729;locus_tag=SPO1243;product=hypothetical protein	337	250	340	401	322	235
CDS;ID=637288730;locus_tag=SPO1244;product=elongation factor P	62	45	60	95	57	60
"CDS;ID=637288731;locus_tag=SPO1245;product=ABC transporter, permease protein"	12	6	12	4	5	5

CDS;ID=637288732;locus_tag=SPO1246;product=aminomethyl transferase family protein	10	15	12	10	6	7
"CDS;ID=637288733;locus_tag=SPO1247;product=glycosyl transferase, group 2 family pr	17	6	5	6	4	7
CDS;ID=640735056;locus_tag=SPO1248	39	45	28	25	26	42
"CDS;ID=637288734;locus_tag=SPO1249;product=paraquat-inducible protein, putative"	25	14	21	14	25	27
"CDS;ID=637288735;locus_tag=SPO1250;product=lipoprotein, putative"	6	9	3	5	2	2
CDS;ID=637288736;locus_tag=SPO1251;product=hypothetical protein	14	11	4	8	6	9
CDS;ID=637288737;locus_tag=SPO1252;product=hypothetical protein	2	8	2	4	6	9
CDS;ID=637288738;locus_tag=SPO1253;product=DNA topoisomerase IV subunit B	69	89	67	78	99	71
CDS;ID=637288739;locus_tag=SPO1254;product=hypothetical protein	14	11	21	16	7	9
CDS;ID=637288740;locus_tag=SPO1255;product=alkylhydroperoxidase AhpD family core dc	35	43	25	27	29	29
CDS;ID=637288741;locus_tag=SPO1256;product=polyphosphate kinase 2	101	64	109	119	254	139
CDS;ID=637288742;locus_tag=SPO1257;product=glutathione S-transferase family proteir	12	16	9	17	27	34
"CDS;ID=637288743;locus_tag=SPO1258;product=hydrolase, alpha/beta fold family"	22	9	4	11	20	13
CDS;ID=637288744;locus_tag=SPO1259;product=hypothetical protein	3	5	3	7	7	3
CDS;ID=637288745;locus_tag=SPO1260;product=MATE efflux family protein	31	26	21	39	51	36
"CDS;ID=637288746;locus_tag=SPO1261;product=transcriptional regulator, MarR family'	2	6	2	3	4	5
CDS;ID=637288747;locus_tag=SPO1262;product=hypothetical protein	4	10	5	12	13	7
CDS;ID=637288748;locus_tag=SPO1263;product=hypothetical protein	30	18	22	15	17	20
CDS;ID=637288749;locus_tag=SPO1264;product=aspartate aminotransferase	51	46	45	52	51	57
"CDS;ID=637288750;locus_tag=SPO1265;product=DNA-binding protein, putative"	81	71	70	80	94	114
CDS;ID=637288751;locus_tag=SPO1266;product=TPR repeat family protein	36	31	36	36	46	26
"CDS;ID=637288752;locus_tag=SPO1267;product=transcriptional regulator, MarR family'	15	18	10	17	11	14
"CDS;ID=637288753;locus_tag=SPO1268;product=lipoprotein, putative"	13	4	6	9	17	17
CDS;ID=637288754;locus_tag=SPO1269;product=hypothetical protein	13	3	4	2	5	3
"CDS;ID=637288755;locus_tag=SPO1270;product=lactoylglutathione lyase, putative"	21	33	17	14	25	11
CDS;ID=637288756;locus_tag=SPO1271;product=hypothetical protein	49	40	31	46	41	44
CDS;ID=637288757;locus_tag=SPO1272;product=hypothetical protein	152	140	173	202	209	153
CDS;ID=637288758;locus_tag=SPO1273;product=thymidylate synthase	87	75	63	45	72	66
CDS;ID=637288759;locus_tag=SPO1274;product=hypothetical protein	17	19	9	14	15	9
CDS;ID=637288760;locus_tag=SPO1275;product=cold shock family protein	59	72	48	37	41	38
CDS;ID=637288761;locus_tag=SPO1276;product=ArsC family protein	24	32	17	40	32	29
CDS;ID=637288762;locus_tag=SPO1277;product=hypothetical protein	16	17	7	14	9	18
CDS;ID=637288763;locus_tag=SPO1278;product=hypothetical protein	10	11	3	5	15	12
CDS;ID=637288764;locus_tag=SPO1279;product=hypothetical protein	10	8	7	7	15	10
CDS;ID=637288765;locus_tag=SPO1280;product=hypothetical protein	3	12	3	0	4	1
CDS;ID=637288766;locus_tag=SPO1281;product=hypothetical protein	10	4	9	10	12	16
CDS;ID=637288767;locus_tag=SPO1282;product=threonyl-tRNA synthetase	116	89	119	112	140	110
CDS;ID=637288768;locus_tag=SPO1283;product=hypothetical protein	2	0	2	0	5	3
"CDS;ID=637288769;locus_tag=SPO1284;product=transcriptional regulator, ArsR family'	11	6	5	10	6	12
CDS;ID=637288770;locus_tag=SPO1285;product=hypothetical protein	26	22	18	17	13	17
CDS;ID=637288771;locus_tag=SPO1286;product=hypothetical protein	6	11	4	5	8	10
CDS;ID=637288772;locus_tag=SPO1287;product=glyoxalase family protein	7	3	2	5	4	4
CDS;ID=637288773;locus_tag=SPO1288;product=hypothetical protein	5	2	3	2	0	1

"CDS;ID=637288774;locus_tag=SPO1289;product=hydrolase, alpha/beta fold family"	35	20	10	17	6	26
CDS;ID=637288775;locus_tag=SPO1290;product=hypothetical protein	25	28	26	27	10	27
"CDS;ID=637288776;locus_tag=SPO1291;product=polyhydroxyalkanoate depolymerase, intr	108	136	89	95	113	110
CDS;ID=637288777;locus_tag=SPO1292;product=poly(3-hydroxyalkanoate) polymerase	154	102	111	164	151	215
"CDS;ID=637288778;locus_tag=SPO1293;product=phasin, PhaP"	468	352	433	429	635	409
CDS;ID=637288779;locus_tag=SPO1294;product=polyhydroxyalkanoate synthesis represso	48	50	49	32	33	42
"CDS;ID=637288780;locus_tag=SPO1295;product=aminotransferase, DegT/DnrJ/EryC1/StrS	38	42	23	22	29	32
CDS;ID=640735057;locus_tag=SPO1296	0	0	1	1	0	0
CDS;ID=640735058;locus_tag=SPO1297	4	5	7	0	2	5
"CDS;ID=637288781;locus_tag=SPO1298;product=oxidoreductase, aldo/keto reductase far	10	18	11	13	6	22
"CDS;ID=637288782;locus_tag=SPO1299;product=transcriptional regulator, LysR family'	24	24	13	16	19	24
CDS;ID=637288783;locus_tag=SPO1300;product=glutamine synthetase family protein	47	28	25	24	30	19
"CDS;ID=637288784;locus_tag=SPO1301;product=glutamine amidotransferase, class I"	16	9	14	18	16	9
CDS;ID=637288785;locus_tag=SPO1302;product=glutamine synthetase family protein	32	17	22	25	15	9
"CDS;ID=637288786;locus_tag=SPO1303;product=fructosyl-amino acid oxidase, putative'	27	19	21	10	10	8
"CDS;ID=637288787;locus_tag=SPO1304;product=His/Glu/Gln/Arg/opine family ABC transp	21	22	18	17	21	12
"CDS;ID=637288788;locus_tag=SPO1305;product=ABC transporter, permease protein, HisM	10	18	11	7	24	4
"CDS;ID=637288789;locus_tag=SPO1306;product=His/Glu/Gln/Arg/opine family ABC transp	150	136	133	99	102	85
"CDS;ID=637288790;locus_tag=SPO1307;product=His/Glu/Gln/Arg/opine family ABC transp	92	70	70	64	98	63
CDS;ID=637288791;locus_tag=SPO1308;product=hypothetical protein	15	16	17	41	22	18
CDS;ID=637288792;locus_tag=SPO1309;product=hypothetical protein	7	6	7	14	19	6
CDS;ID=637288793;locus_tag=SPO1310;product=hypothetical protein	16	19	17	19	26	19
CDS;ID=637288794;locus_tag=SPO1311;product=renal dipeptidase family protein	34	25	33	27	36	34
CDS;ID=637288795;locus_tag=SPO1312;product=CTP synthetase	161	134	148	185	190	178
"CDS;ID=637288796;locus_tag=SPO1313;product=preprotein translocase, SecG subunit"	70	42	41	47	29	66
CDS;ID=637288797;locus_tag=SPO1314;product=nitrile hydratase subunit alpha	43	34	32	34	63	26
CDS;ID=637288798;locus_tag=SPO1315;product=nitrile hydratase beta subunit	30	20	33	17	19	21
CDS;ID=637288799;locus_tag=SPO1316;product=hypothetical protein	10	11	9	6	10	12
CDS;ID=637288800;locus_tag=SPO1317;product=hypothetical protein	4	0	0	2	3	1
CDS;ID=637288801;locus_tag=SPO1318;product=adenylosuccinate synthetase	121	85	72	120	94	98
CDS;ID=637288802;locus_tag=SPO1319;product=hypothetical protein	14	14	9	17	14	8
"CDS;ID=637288803;locus_tag=SPO1320;product=lipoprotein, putative"	43	41	53	44	44	51
CDS;ID=637288804;locus_tag=SPO1321;product=thiamine pyrophosphokinase	15	15	17	20	20	26
CDS;ID=637288805;locus_tag=SPO1322;product=hypothetical protein	2	2	1	8	14	3
CDS;ID=637288806;locus_tag=SPO1323;product=L-serine ammonia-lyase	19	15	16	12	12	17
CDS;ID=637288807;locus_tag=SPO1324;product=glutathione S-transferase family protein	2	7	1	3	2	4
"CDS;ID=637288808;locus_tag=SPO1325;product=lipoprotein, putative"	1	1	1	1	2	0
"CDS;ID=637288809;locus_tag=SPO1326;product=transcriptional regulator, MarR family'	5	2	6	7	9	2
CDS;ID=637288810;locus_tag=SPO1327;product=ribose 5-phosphate isomerase	71	61	52	75	66	94
CDS;ID=637288811;locus_tag=SPO1328;product=glutathione-disulfide reductase	135	132	147	123	168	120
CDS;ID=637288812;locus_tag=SPO1329;product=HflK protein	175	153	144	161	211	214
CDS;ID=637288813;locus_tag=SPO1330;product=HflC protein	69	52	47	38	57	59
CDS;ID=637288814;locus_tag=SPO1331;product=hypothetical protein	36	34	36	27	30	40

CDS;ID=637288815;locus_tag=SPO1332;product=hypothetical protein	8	1	2	6	3	2
"CDS;ID=637288816;locus_tag=SPO1333;product=periplasmic serine protease, DO/DeqQ fa	259	216	176	126	153	206
CDS;ID=637288817;locus_tag=SPO1334;product=hypothetical protein	11	13	7	4	13	10
"CDS;ID=637288818;locus_tag=SPO1335;product=transcriptional regulator, Crp/Fnr fam:	25	21	22	22	9	36
CDS;ID=637288819;locus_tag=SPO1336;product=intracellular septation protein A	34	29	28	19	40	37
CDS;ID=637288820;locus_tag=SPO1337;product=hypothetical protein	18	18	9	12	13	18
CDS;ID=637288821;locus_tag=SPO1339;product=signal recognition particle-docking prot	16	13	17	23	24	10
CDS;ID=637288822;locus_tag=SPO1340;product=hypothetical protein	3	1	1	0	2	1
"CDS;ID=637288823;locus_tag=SPO1341;product=alkane-1 monooxygenase, putative"	0	4	5	3	11	4
CDS;ID=637288824;locus_tag=SPO1342;product=hypothetical protein	79	77	85	72	44	84
CDS;ID=637288825;locus_tag=SPO1343;product=glutathione S-transferase family protein	4	1	3	6	3	8
"CDS;ID=637288826;locus_tag=SPO1344;product=exodeoxyribonuclease VII, large subunit	14	11	7	15	8	15
CDS;ID=637288827;locus_tag=SPO1345;product=phosphoribosylamine--glycine ligase	82	87	82	105	126	119
"CDS;ID=637288828;locus_tag=SPO1346;product=transcriptional regulator, LuxR family,	42	20	17	26	35	20
CDS;ID=637288829;locus_tag=SPO1347;product=hypothetical protein	8	5	4	10	7	8
CDS;ID=637288830;locus_tag=SPO1348;product=iron-sulfur cluster-binding protein	35	29	28	20	34	19
"CDS;ID=637288831;locus_tag=SPO1349;product=lipoprotein, putative"	30	18	18	24	25	31
CDS;ID=637288832;locus_tag=SPO1350;product=hypothetical protein	3	0	1	0	0	1
CDS;ID=637288833;locus_tag=SPO1351;product=O-succinylhomoserine sulfhydrylase	18	7	9	13	21	18
CDS;ID=637288834;locus_tag=SPO1352;product=hypothetical protein	59	57	35	28	40	40
CDS;ID=637288835;locus_tag=SPO1353;product=Trk system potassium uptake protein TrkH	56	52	46	40	39	65
CDS;ID=637288836;locus_tag=SPO1354;product=peptidoglycan binding domain protein	71	49	58	68	77	63
CDS;ID=637288837;locus_tag=SPO1355;product=glycyl-tRNA synthetase alpha subunit	115	111	76	126	157	117
"CDS;ID=637288838;locus_tag=SPO1356;product=methyltransferase, FkbM family"	81	70	93	85	101	102
CDS;ID=637288839;locus_tag=SPO1357;product=sodium/glutamate symporter	17	24	17	24	44	24
CDS;ID=637288840;locus_tag=SPO1358;product=glycyl-tRNA synthetase beta subunit	65	65	50	74	62	70
CDS;ID=637288841;locus_tag=SPO1359;product=pyruvate phosphate dikinase	73	61	81	62	61	92
CDS;ID=637288842;locus_tag=SPO1360;product=hypothetical protein	41	18	31	29	27	34
CDS;ID=637288843;locus_tag=SPO1361;product=multicopper oxidase domain protein	3	0	2	5	7	3
"CDS;ID=637288844;locus_tag=SPO1362;product=dihydroneopterin aldolase, putative"	34	37	33	31	36	53
CDS;ID=637288845;locus_tag=SPO1363;product=dihydropteroate synthase	13	15	11	13	15	16
CDS;ID=637288846;locus_tag=SPO1364;product=phosphoglucosamine mutase	197	170	145	145	219	213
CDS;ID=637288847;locus_tag=SPO1365;product=hypothetical protein	15	8	8	9	2	14
CDS;ID=637288848;locus_tag=SPO1366;product=ketol-acid reductoisomerase	44	48	40	48	59	61
"CDS;ID=637288849;locus_tag=SPO1367;product=transcriptional regulator, AsnC family'	0	2	2	4	3	1
"CDS;ID=637288850;locus_tag=SPO1368;product=transcriptional regulator, AsnC family'	5	4	2	1	5	8
"CDS;ID=637288851;locus_tag=SPO1369;product=transcriptional regulator, GntR family'	35	34	24	33	48	49
CDS;ID=637288852;locus_tag=SPO1370;product=aminotransferase family protein	26	16	26	20	18	25
CDS;ID=637288853;locus_tag=SPO1371;product=2-octaprenyl-6-methoxyphenyl hydroxylase	15	11	13	33	16	18
CDS;ID=637288854;locus_tag=SPO1372;product=hypothetical protein	9	7	1	11	10	8
CDS;ID=637288855;locus_tag=SPO1373;product=hypothetical protein	12	8	8	4	6	6
CDS;ID=637288856;locus_tag=SPO1374;product=pyrimidine 5'-nucleotidase	40	27	18	30	14	29
"CDS;ID=637288857;locus_tag=SPO1375;product=transcriptional regulator, GntR family'	35	40	17	23	32	33

"CDS;ID=637288858;locus_tag=SPO1376;product=glycosyl transferase, group 2 family pı	9	14	5	10	11	15
CDS;ID=637288859;locus_tag=SPO1377;product=carbamoyl-phosphate synthase small subuı	64	53	56	70	97	52
CDS;ID=637288860;locus_tag=SPO1378;product=GatB/YqeY domain protein	245	233	246	213	204	228
"CDS;ID=637288861;locus_tag=SPO1379;product=prephenate dehydrogenase, putative"	14	5	6	5	6	11
CDS;ID=637288862;locus_tag=SPO1380;product=hypothetical protein	16	16	22	22	21	26
CDS;ID=637288863;locus_tag=SPO1381;product=hypothetical protein	6	6	11	6	11	10
CDS;ID=637288864;locus_tag=SPO1382;product=hypothetical protein	62	52	49	84	54	72
"CDS;ID=637288865;locus_tag=SPO1383;product=cytochrome c oxidase, aa3-type, subunit	196	191	151	201	168	136
"CDS;ID=637288866;locus_tag=SPO1384;product=transcriptional regulator, MarR family"	21	15	16	9	10	13
CDS;ID=637288867;locus_tag=SPO1385;product=twin-arginine translocation pathway sigı	21	16	16	14	7	18
CDS;ID=637288868;locus_tag=SPO1386;product=HIT family protein	18	17	7	17	16	14
CDS;ID=637288869;locus_tag=SPO1387;product=cation efflux system protein	19	15	9	9	7	12
"CDS;ID=637288870;locus_tag=SPO1388;product=transcriptional regulator, LuxR family/	108	118	86	91	77	81
CDS;ID=637288871;locus_tag=SPO1389;product=lipoyltransferase	14	8	9	25	29	42
"CDS;ID=637288872;locus_tag=SPO1390;product=thioredoxin domain protein, DsbA family	7	4	5	9	4	4
tRNA;ID=640698527;locus_tag=SPO_tRNA-Leu-2	481	367	162	200	33	271
CDS;ID=637288874;locus_tag=SPO1392;product=4-carboxymuconolactone decarboxylase doı	5	5	2	2	7	5
CDS;ID=637288875;locus_tag=SPO1393;product=Rrf2 family protein	4	5	8	10	2	8
"CDS;ID=637288876;locus_tag=SPO1394;product=beta-lactamase, putative"	4	6	4	5	4	6
"CDS;ID=637288877;locus_tag=SPO1395;product=transcriptional regulator, AraC family"	0	3	3	1	2	2
"CDS;ID=637288878;locus_tag=SPO1396;product=transporter, putative"	11	9	9	6	10	4
CDS;ID=637288879;locus_tag=SPO1397;product=hydrophobe/amphiphile efflux-1 family pı	47	51	39	30	34	39
"CDS;ID=637288880;locus_tag=SPO1398;product=efflux transporter, RND family, MFP suk	35	47	33	29	29	19
"CDS;ID=637288881;locus_tag=SPO1399;product=transcriptional regulator, AraC family"	7	4	13	7	8	6
CDS;ID=637288882;locus_tag=SPO1400;product=hypothetical protein	8	0	3	1	3	2
CDS;ID=637288883;locus_tag=SPO1401;product=hypothetical protein	3	4	0	8	5	2
CDS;ID=637288884;locus_tag=SPO1402;product=hypothetical protein	47	26	42	44	24	32
CDS;ID=637288885;locus_tag=SPO1403;product=methionyl-tRNA synthetase	107	98	69	125	109	108
CDS;ID=637288886;locus_tag=SPO1404;product=hypothetical protein	13	13	11	23	11	10
CDS;ID=637288887;locus_tag=SPO1405;product=tyrosinase domain protein	12	5	1	6	4	6
CDS;ID=637288888;locus_tag=SPO1406;product=hypothetical protein	660	408	329	272	125	296
CDS;ID=637288889;locus_tag=SPO1407;product=hypothetical protein	17	26	15	18	17	19
CDS;ID=637288890;locus_tag=SPO1408;product=ribosomal large subunit pseudouridine sy	12	12	7	12	22	9
CDS;ID=637288891;locus_tag=SPO1409;product=RNA polymerase sigma factor	201	155	177	181	200	198
CDS;ID=637288892;locus_tag=SPO1410;product=renal dipeptidase family protein	7	10	6	3	7	10
"CDS;ID=637288893;locus_tag=SPO1411;product=C4-type zinc finger protein, DksA/TraR	8	7	9	5	5	13
CDS;ID=637288894;locus_tag=SPO1412;product=DNA modification methyltransferase domaı	7	0	2	3	3	2
CDS;ID=637288895;locus_tag=SPO1413;product=oligoendopeptidase F	203	199	184	207	208	231
"CDS;ID=637288896;locus_tag=SPO1414;product=hydrolase, alpha/beta fold family"	42	35	38	20	43	38
CDS;ID=637288897;locus_tag=SPO1415;product=sterol carrier family protein	96	75	58	69	63	74
CDS;ID=637288898;locus_tag=SPO1416;product=TPR domain protein	73	63	53	61	51	65
"CDS;ID=637288899;locus_tag=SPO1417;product=helicase, putative"	483	416	371	393	371	554
CDS;ID=637288900;locus_tag=SPO1418;product=S4 domain protein	42	22	21	29	24	42

CDS;ID=637288901;locus_tag=SPO1419;product=iron-sulfur cluster-binding protein	78	116	104	111	79	129
"CDS;ID=637288902;locus_tag=SPO1420;product=transcriptional regulator, CarD family'	97	83	79	90	97	58
CDS;ID=637288903;locus_tag=SPO1421;product=cation channel family protein	17	12	10	15	15	11
CDS;ID=637288904;locus_tag=SPO1422;product=cobalamin 5'-phosphate synthase	9	5	8	0	5	1
CDS;ID=637288905;locus_tag=SPO1423;product=nicotinate-nucleotide--dimethylbenzimida	26	28	39	27	37	41
CDS;ID=637288906;locus_tag=SPO1424;product=glutathione-regulated potassium-efflux s	49	31	38	37	41	41
"CDS;ID=637288907;locus_tag=SPO1425;product=transcriptional regulator, AsnC family'	147	93	68	85	55	111
CDS;ID=637288908;locus_tag=SPO1426;product=4-hydroxyphenylpyruvate dioxygenase	33	23	23	19	21	15
CDS;ID=637288909;locus_tag=SPO1427;product=hypothetical protein	36	22	24	12	27	18
CDS;ID=637288910;locus_tag=SPO1428;product=hypothetical protein	40	21	26	17	27	27
"CDS;ID=637288911;locus_tag=SPO1429;product=outer membrane protein, 28Kda"	113	84	57	87	96	104
CDS;ID=637288912;locus_tag=SPO1430;product=antibiotic efflux protein	42	29	23	30	34	36
CDS;ID=637288913;locus_tag=SPO1431;product=O-acetylhomoserine aminocarboxypropyltra	48	39	45	15	47	31
CDS;ID=637288914;locus_tag=SPO1432;product=rhodanese domain protein	56	41	32	29	52	41
"CDS;ID=637288915;locus_tag=SPO1433;product=oxidoreductase, aldo/keto reductase far	36	33	28	19	34	37
"CDS;ID=637288916;locus_tag=SPO1434;product=transcriptional regulator, AraC family'	14	6	11	8	34	6
CDS;ID=637288917;locus_tag=SPO1435;product=hypothetical protein	0	6	1	5	1	2
CDS;ID=637288918;locus_tag=SPO1436;product=3-hydroxybutyryl-CoA dehydrogenase	5	3	2	4	4	5
CDS;ID=637288919;locus_tag=SPO1437;product=short chain dehydrogenase	2	1	1	2	0	1
CDS;ID=637288920;locus_tag=SPO1438;product=hypothetical protein	41	26	30	24	26	18
"CDS;ID=637288921;locus_tag=SPO1439;product=DNA binding protein, putative"	10	10	13	9	13	8
CDS;ID=637288922;locus_tag=SPO1440;product=hypothetical protein	0	2	0	2	0	4
CDS;ID=637288923;locus_tag=SPO1441;product=fatty acid desaturase family protein	5	6	3	4	2	7
CDS;ID=637288924;locus_tag=SPO1442;product=hypothetical protein	43	42	37	25	44	49
CDS;ID=637288925;locus_tag=SPO1443;product=ATP-dependent RNA helicase RhlE	117	90	95	88	170	115
CDS;ID=637288926;locus_tag=SPO1444;product=hypothetical protein	25	15	27	13	23	18
"CDS;ID=637288927;locus_tag=SPO1445;product=oxidoreductase, short chain dehydrogena	4	0	3	1	5	4
"CDS;ID=637288928;locus_tag=SPO1446;product=cyclase, putative"	3	2	6	3	3	6
"CDS;ID=637288929;locus_tag=SPO1447;product=oxidoreductase, short chain dehydrogena	3	2	1	5	11	5
CDS;ID=637288930;locus_tag=SPO1448;product=hypothetical protein	15	18	18	11	11	8
CDS;ID=637288931;locus_tag=SPO1449;product=AMP-binding protein	9	7	4	10	4	8
"CDS;ID=637288932;locus_tag=SPO1450;product=aromatic 1,2-dioxygenase, beta subunit'	2	6	1	3	2	1
"CDS;ID=637288933;locus_tag=SPO1451;product=aromatic 1,2-dioxygenase, alpha subunit	32	30	18	21	17	23
CDS;ID=637288934;locus_tag=SPO1452;product=oxidoreductase NAD-binding domain/2Fe-2S	23	13	24	20	30	19
"CDS;ID=637288935;locus_tag=SPO1453;product=transcriptional regulator, MarR family'	11	5	3	5	16	6
"CDS;ID=637288936;locus_tag=SPO1454;product=TRAP dicarboxylate transporter, DctP su	32	33	24	17	36	34
"CDS;ID=637288937;locus_tag=SPO1455;product=TRAP dicarboxylate transporter, DctQ su	1	2	2	3	1	2
"CDS;ID=637288938;locus_tag=SPO1456;product=TRAP transporter, DctM subunit"	11	4	6	9	4	8
CDS;ID=637288939;locus_tag=SPO1457;product=thioesterase family protein	7	11	9	17	13	15
"CDS;ID=637288940;locus_tag=SPO1458;product=transcriptional regulator, MarR family'	19	15	18	16	8	18
"CDS;ID=637288941;locus_tag=SPO1459;product=indolepyruvate oxidoreductase, IorA sub	20	19	13	11	22	26
CDS;ID=637288942;locus_tag=SPO1460;product=indolepyruvate ferredoxin oxidoreductase	29	24	27	12	19	31
CDS;ID=637288943;locus_tag=SPO1461;product=hypothetical protein	25	15	22	17	16	15

CDS;ID=637288944;locus_tag=SPO1462;product=OmpA family protein	37	34	22	24	18	24
"CDS;ID=637288945;locus_tag=SPO1463;product=TRAP dicarboxylate transporter, DctM su	4	4	1	2	1	0
"CDS;ID=637288946;locus_tag=SPO1464;product=TRAP dicarboxylate transporter, DctQ su	4	3	1	0	6	2
"CDS;ID=637288947;locus_tag=SPO1465;product=TRAP dicarboxylate transporter, DctP su	12	9	11	10	8	8
"CDS;ID=637288948;locus_tag=SPO1466;product=opine dehydrogenase, putative"	12	6	3	5	8	11
CDS;ID=637288949;locus_tag=SPO1467;product=3-hydroxybutyryl-CoA dehydrogenase	1	1	2	1	0	3
CDS;ID=637288950;locus_tag=SPO1468;product=aminotransferase family protein	11	7	4	6	7	3
"CDS;ID=637288951;locus_tag=SPO1469;product=3-ketosteroid dehydrogenase, putative"	0	1	1	5	2	2
CDS;ID=637288952;locus_tag=SPO1470;product=isocitrate lyase family protein	0	1	1	7	2	2
CDS;ID=637288953;locus_tag=SPO1471;product=isochorismatase family protein	3	3	0	3	4	9
CDS;ID=637288954;locus_tag=SPO1472;product=hydantoin utilization protein A	6	8	4	6	5	4
"CDS;ID=637288955;locus_tag=SPO1473;product=hydantoin utilization protein B, putati	7	7	4	7	6	10
"CDS;ID=637288956;locus_tag=SPO1474;product=tautomerase, putative"	0	10	8	6	4	4
"CDS;ID=637288957;locus_tag=SPO1475;product=transcriptional regulator, GntR family"	15	9	10	6	5	11
CDS;ID=637288958;locus_tag=SPO1476;product=isopropylmalate isomerase large subunit	6	7	4	7	10	3
"CDS;ID=637288959;locus_tag=SPO1477;product=3-isopropylmalate dehydratase, small su	3	4	2	1	1	2
CDS;ID=637288960;locus_tag=SPO1478;product=RNA polymerase sigma-70 factor	36	35	18	31	17	27
"CDS;ID=637288961;locus_tag=SPO1479;product=transcriptional activator, putative"	8	9	6	8	6	5
"CDS;ID=637288962;locus_tag=SPO1480;product=oxidoreductase, short-chain dehydrogena	2	2	0	0	1	0
CDS;ID=637288963;locus_tag=SPO1481;product=hypothetical protein	7	3	8	6	0	10
CDS;ID=637288964;locus_tag=SPO1482;product=hypothetical protein	8	7	1	6	6	7
"CDS;ID=637288965;locus_tag=SPO1483;product=cyclopropane-fatty-acyl-phospholipid sy	6	6	0	6	1	1
CDS;ID=637288966;locus_tag=SPO1484;product=hypothetical protein	1	0	5	6	4	4
CDS;ID=637288967;locus_tag=SPO1485;product=sodium:galactoside symporter family prot	8	8	9	4	9	9
CDS;ID=637288968;locus_tag=SPO1486;product=hypothetical protein	1	1	0	2	1	6
CDS;ID=637288969;locus_tag=SPO1487;product=hypothetical protein	2	0	1	2	1	1
"CDS;ID=637288970;locus_tag=SPO1488;product=transcriptional regulator, LuxR family"	23	25	15	9	11	30
CDS;ID=637288971;locus_tag=SPO1489;product=indole acetimide hydrolase	28	28	13	11	26	21
"CDS;ID=637288972;locus_tag=SPO1490;product=regulatory protein, putative"	24	34	34	22	20	30
"CDS;ID=637288973;locus_tag=SPO1491;product=branched-chain amino acid ABC transport	9	4	1	4	4	7
"CDS;ID=637288974;locus_tag=SPO1492;product=branched-chain amino acid ABC transport	33	32	24	21	27	46
"CDS;ID=637288975;locus_tag=SPO1493;product=branched-chain amino acid ABC transport	17	8	7	10	12	14
"CDS;ID=637288976;locus_tag=SPO1494;product=2-hydroxychromene-2-carboxylate isomera	7	9	3	8	3	4
"CDS;ID=637288977;locus_tag=SPO1495;product=ABC transporter, ATP-binding protein"	21	26	17	19	34	39
"CDS;ID=637288978;locus_tag=SPO1496;product=ABC transporter, permease protein"	43	31	46	28	66	38
"CDS;ID=637288979;locus_tag=SPO1497;product=efflux transporter, RND family, MFP sub	25	16	8	10	14	17
"CDS;ID=637288980;locus_tag=SPO1498;product=fumarate hydratase, class I, putative"	78	80	47	50	94	85
"CDS;ID=637288981;locus_tag=SPO1499;product=hydrolase, alpha/beta fold family"	16	14	12	6	12	14
CDS;ID=637288982;locus_tag=SPO1500;product=pyrroloquinoline quinone biosynthesis pr	16	12	9	14	8	15
CDS;ID=637288983;locus_tag=SPO1501;product=coenzyme PQQ synthesis protein D	1	1	3	4	1	3
CDS;ID=637288984;locus_tag=SPO1502;product=pyrroloquinoline quinone biosynthesis pr	9	6	11	9	6	9
CDS;ID=637288985;locus_tag=SPO1503;product=pyrroloquinoline quinone biosynthesis pr	4	6	7	5	5	6
CDS;ID=637288986;locus_tag=SPO1504;product=coenzyme PQQ biosynthesis protein A	69	33	17	25	35	41

CDS;ID=637288987;locus_tag=SPO1505;product=sensor histidine kinase/response regulat	2	9	2	5	6	7
CDS;ID=637288988;locus_tag=SPO1506;product=GfdT protein	10	7	10	11	3	9
"CDS;ID=637288989;locus_tag=SPO1507;product=DNA-binding response regulator, LuxR fa	21	10	11	15	10	7
CDS;ID=637288990;locus_tag=SPO1508;product=quinoprotein ethanol dehydrogenase	45	25	24	24	38	64
CDS;ID=637288991;locus_tag=SPO1509;product=cytochrome c family protein	3	2	0	5	4	4
"CDS;ID=637288992;locus_tag=SPO1510;product=efflux ABC transporter, permease protei	13	4	6	3	7	4
"CDS;ID=637288993;locus_tag=SPO1511;product=efflux ABC transporter, ATP-binding pro	18	10	6	14	14	21
CDS;ID=637288994;locus_tag=SPO1512;product=hypothetical protein	6	1	3	5	5	3
CDS;ID=637288995;locus_tag=SPO1513;product=hypothetical protein	10	4	3	4	6	1
CDS;ID=637288996;locus_tag=SPO1514;product=hypothetical protein	3	4	3	2	3	1
"CDS;ID=637288997;locus_tag=SPO1515;product=cytochrome c550, putative"	4	1	1	3	5	7
"CDS;ID=637288998;locus_tag=SPO1516;product=amino acid ABC transporter, periplasmic	12	16	15	18	15	15
CDS;ID=637288999;locus_tag=SPO1517;product=carbon monoxide dehydrogenase operon G p	40	35	23	25	61	31
"CDS;ID=637289000;locus_tag=SPO1518;product=carbon-monoxide dehydrogenase, small su	71	54	33	42	59	66
"CDS;ID=637289001;locus_tag=SPO1519;product=carbon monoxide dehydrogenase, large su	75	56	42	74	44	85
"CDS;ID=637289002;locus_tag=SPO1520;product=carbon monoxide dehydrogenase, medium s	10	8	5	12	13	19
"CDS;ID=637289003;locus_tag=SPO1521;product=regulatory protein, putative"	13	9	12	7	7	7
CDS;ID=637289004;locus_tag=SPO1522;product=hypothetical protein	9	3	5	2	3	8
CDS;ID=637289005;locus_tag=SPO1523;product=metallo-beta-lactamase family protein	0	3	2	7	1	4
CDS;ID=637289006;locus_tag=SPO1524;product=hypothetical protein	22	8	10	14	2	5
"CDS;ID=637289007;locus_tag=SPO1525;product=transporter, AcrB/AcrD/AcrF family"	4	2	2	1	4	5
"CDS;ID=637289008;locus_tag=SPO1526;product=efflux transporter, RND family, MFP sub	3	0	3	1	4	5
CDS;ID=637289009;locus_tag=SPO1527;product=hypothetical protein	0	0	0	1	0	1
CDS;ID=637289010;locus_tag=SPO1528;product=hypothetical protein	2	2	1	1	0	4
CDS;ID=637289011;locus_tag=SPO1529;product=hypothetical protein	13	8	3	7	4	9
CDS;ID=637289012;locus_tag=SPO1530;product=integral membrane protein MviN	12	5	5	1	6	5
CDS;ID=637289013;locus_tag=SPO1531;product=hypothetical protein	5	6	2	3	1	7
CDS;ID=637289014;locus_tag=SPO1532;product=hypothetical protein	5	5	6	3	3	0
"CDS;ID=637289015;locus_tag=SPO1533;product=glycosyl transferase, group 1 family pr	9	4	4	2	2	12
CDS;ID=637289016;locus_tag=SPO1534;product=bacterial sugar transferase	15	6	8	12	13	25
CDS;ID=637289017;locus_tag=SPO1535;product=polysaccharide deacetylase domain protei	4	2	2	1	3	3
"CDS;ID=637289018;locus_tag=SPO1536;product=glycosyltransferase, group 1"	4	2	2	2	3	7
"CDS;ID=637289019;locus_tag=SPO1537;product=twin-arginine translocation pathway sig	0	0	1	0	1	0
CDS;ID=637289020;locus_tag=SPO1538;product=chain length determinant protein	8	13	13	13	9	18
"CDS;ID=637289021;locus_tag=SPO1539;product=O-antigen polymerase, putative"	2	2	1	0	2	1
"CDS;ID=637289022;locus_tag=SPO1540;product=glycosyl transferase, group 2"	3	2	3	3	1	1
"CDS;ID=637289023;locus_tag=SPO1541;product=transcriptional regulator, LysR family"	4	4	3	0	7	5
CDS;ID=637289024;locus_tag=SPO1542;product=renal dipeptidase family protein	11	15	15	5	8	8
"CDS;ID=637289025;locus_tag=SPO1543;product=peptide/opine/nickel uptake family ABC	27	22	20	23	17	25
"CDS;ID=637289026;locus_tag=SPO1544;product=peptide/opine/nickel uptake family ABC	4	8	5	1	1	3
"CDS;ID=637289027;locus_tag=SPO1545;product=peptide/opine/nickel uptake family ABC	5	8	1	6	4	4
"CDS;ID=637289028;locus_tag=SPO1546;product=peptide/opine/nickel uptake family ABC	19	18	15	17	7	8
"CDS;ID=637289029;locus_tag=SPO1547;product=peptide/opine/nickel uptake family ABC	0	0	0	2	1	1

"CDS;ID=637289030;locus_tag=SPO1548;product=glycine betaine/proline ABC transporter	5	3	2	6	1	6
"CDS;ID=637289031;locus_tag=SPO1549;product=glycine betaine/proline ABC transporter	1	3	0	4	3	5
"CDS;ID=637289032;locus_tag=SPO1550;product=glycine betaine/proline ABC transporter	9	6	4	7	8	3
CDS;ID=637289033;locus_tag=SPO1551;product=flavin-containing monooxygenase	16	18	34	17	30	13
"CDS;ID=637289034;locus_tag=SPO1552;product=ABC transporter, periplasmic substrate-	19	26	23	21	21	35
"CDS;ID=637289035;locus_tag=SPO1553;product=transcriptional regulator, GntR family"	26	36	21	21	33	57
CDS;ID=637289036;locus_tag=SPO1554;product=ammonium transporter family protein	15	22	15	6	24	23
"CDS;ID=637289037;locus_tag=SPO1555;product=formate dehydrogenase, beta subunit"	35	28	27	30	36	46
"CDS;ID=637289038;locus_tag=SPO1556;product=formate dehydrogenase, alpha subunit"	44	39	47	48	49	48
CDS;ID=637289039;locus_tag=SPO1557;product=formate--tetrahydrofolate ligase	138	112	95	111	151	129
CDS;ID=637289040;locus_tag=SPO1558;product=chorismate mutase family protein	30	15	16	22	36	27
CDS;ID=637289041;locus_tag=SPO1559;product=folD bifunctional protein	66	61	39	58	78	65
CDS;ID=637289042;locus_tag=SPO1560;product=folD bifunctional protein	9	11	7	7	10	10
CDS;ID=637289043;locus_tag=SPO1561;product=hypothetical protein	9	5	5	2	3	9
"CDS;ID=637289044;locus_tag=SPO1562;product=glycine cleavage system T protein, puta	12	5	11	12	16	7
CDS;ID=637289045;locus_tag=SPO1563;product=protozoan/cyanobacterial globin family p	6	8	15	21	3	14
CDS;ID=637289046;locus_tag=SPO1564;product=MOFRL domain protein	11	11	10	1	2	4
"CDS;ID=637289047;locus_tag=SPO1565;product=citrate lyase, putative"	38	22	38	10	24	14
"CDS;ID=637289048;locus_tag=SPO1566;product=DNA-binding response regulator, LuxR fa	10	8	5	10	9	16
"CDS;ID=637289049;locus_tag=SPO1567;product=aminotransferase, putative"	2	2	1	1	1	0
CDS;ID=637289050;locus_tag=SPO1568;product=succinyl-CoA synthetase subunit beta	5	12	2	8	6	0
CDS;ID=637289051;locus_tag=SPO1569;product=succinyl-CoA synthetase alpha subunit	1	0	0	1	1	0
CDS;ID=637289052;locus_tag=SPO1570;product=2-hydroxyacid dehydrogenase	0	0	1	0	0	0
CDS;ID=637289053;locus_tag=SPO1571;product=phosphoenolpyruvate carboxylase	5	7	8	6	4	5
CDS;ID=637289054;locus_tag=SPO1572;product=serine hydroxymethyltransferase	60	41	37	46	38	32
CDS;ID=637289055;locus_tag=SPO1573;product=glutamine synthetase III	6	7	4	6	7	8
CDS;ID=637289056;locus_tag=SPO1574;product=glutamate synthase family protein	9	10	9	17	17	16
CDS;ID=637289057;locus_tag=SPO1575;product=FwdC/FmdC family protein	6	3	0	5	9	3
"CDS;ID=637289058;locus_tag=SPO1576;product=glutamine amidotransferase, class II"	8	7	13	14	5	7
CDS;ID=637289059;locus_tag=SPO1577;product=hypothetical protein	6	10	6	6	6	7
CDS;ID=637289060;locus_tag=SPO1578;product=ammonium transporter family protein	5	7	0	6	9	12
CDS;ID=637289061;locus_tag=SPO1579;product=aminomethyl transferase family protein	7	6	7	6	10	16
CDS;ID=637289062;locus_tag=SPO1580;product=hypothetical protein	5	10	4	7	5	0
CDS;ID=637289063;locus_tag=SPO1581;product=oxidoreductase NAD-binding domain/2Fe-2S	3	2	0	4	2	4
CDS;ID=637289064;locus_tag=SPO1582;product=hypothetical protein	17	16	15	8	10	15
"CDS;ID=637289065;locus_tag=SPO1583;product=DNA-binding protein, putative"	58	43	40	47	55	50
"CDS;ID=637289066;locus_tag=SPO1584;product=transcriptional regulator, AraC family"	7	6	1	6	3	5
"CDS;ID=637289067;locus_tag=SPO1585;product=sarcosine oxidase, gamma subunit family	1	1	0	0	2	1
"CDS;ID=637289068;locus_tag=SPO1586;product=sarcosine oxidase, alpha subunit family	13	11	9	14	21	13
"CDS;ID=637289069;locus_tag=SPO1587;product=sarcosine oxidase, delta subunit family	0	0	0	0	0	1
"CDS;ID=637289070;locus_tag=SPO1588;product=sarcosine oxidase, beta subunit family"	1	1	3	4	5	6
CDS;ID=637289071;locus_tag=SPO1589;product=carboxymuconolactone decarboxylase famil	11	15	15	16	12	9
CDS;ID=637289072;locus_tag=SPO1590;product=Rieske 2Fe-2S domain protein	34	43	30	19	27	43

CDS;ID=637289073;locus_tag=SPO1591;product=SIS domain protein	1	5	1	4	4	8
CDS;ID=637289074;locus_tag=SPO1592;product=aminomethyl transferase family protein	11	6	7	4	2	7
"CDS;ID=637289075;locus_tag=SPO1593;product=alcohol dehydrogenase, zinc-containing"	1	0	3	6	3	1
"CDS;ID=637289076;locus_tag=SPO1594;product=mandelate racemase/muconate lactonizing	2	0	4	4	3	4
CDS;ID=637289077;locus_tag=SPO1595;product=mandelate racemase/muconate lactonizing	8	6	2	5	8	4
CDS;ID=637289078;locus_tag=SPO1596;product=hypothetical protein	10	4	5	4	7	2
"CDS;ID=637289079;locus_tag=SPO1597;product=glutamate-1-semialdehyde 2,1-aminomutas	8	8	15	2	8	6
CDS;ID=637289080;locus_tag=SPO1598;product=hypothetical protein	8	4	2	4	4	3
CDS;ID=637289081;locus_tag=SPO1599;product=MmgE/PrpD family protein	10	4	1	11	3	5
CDS;ID=637289082;locus_tag=SPO1600;product=fumarylacetoacetate hydrolase family pro	1	1	0	0	3	2
CDS;ID=637289083;locus_tag=SPO1601;product=2-dehydropantoate 2-reductase	3	0	3	1	0	6
CDS;ID=637289084;locus_tag=SPO1602;product=trimethylamine methyltransferase family	0	0	2	0	0	4
CDS;ID=637289085;locus_tag=SPO1603;product=malate/L-lactate dehydrogenase family pr	3	0	1	0	0	4
"CDS;ID=637289086;locus_tag=SPO1604;product=transcriptional regulator, LysR family"	19	23	20	25	15	35
CDS;ID=637289087;locus_tag=SPO1605;product=hypothetical protein	31	29	32	17	27	17
"CDS;ID=637289088;locus_tag=SPO1606;product=spermidine/putrescine ABC transporter,	76	65	64	54	74	72
"CDS;ID=637289089;locus_tag=SPO1607;product=spermidine/putrescine ABC transporter,	34	39	43	29	45	27
"CDS;ID=637289090;locus_tag=SPO1608;product=spermidine/putrescine ABC transporter,	11	18	14	4	23	17
"CDS;ID=637289091;locus_tag=SPO1609;product=spermidine/putrescine ABC transporter,	106	91	104	89	123	120
"CDS;ID=637289092;locus_tag=SPO1610;product=transcriptional regulator, LuxR family"	8	1	5	2	1	3
CDS;ID=637289093;locus_tag=SPO1611;product=hypothetical protein	5	5	3	5	4	6
CDS;ID=637289094;locus_tag=SPO1612;product=twin-arginine translocation pathway sigr	2	1	0	0	1	3
CDS;ID=637289095;locus_tag=SPO1613;product=hypothetical protein	3	2	2	0	0	2
CDS;ID=637289096;locus_tag=SPO1614;product=hypothetical protein	0	0	1	0	0	0
CDS;ID=637289097;locus_tag=SPO1615;product=cyclic nucleotide-binding protein	1	0	0	8	5	3
"CDS;ID=637289098;locus_tag=SPO1616;product=transcriptional regulator, putative"	3	4	4	9	4	6
CDS;ID=637289099;locus_tag=SPO1617;product=type I secretion target repeat protein	20	4	7	12	8	9
CDS;ID=637289100;locus_tag=SPO1618;product=hypothetical protein	1	1	0	0	0	0
CDS;ID=637289101;locus_tag=SPO1619;product=hypothetical protein	0	0	0	1	0	0
CDS;ID=637289102;locus_tag=SPO1620;product=glyoxalase family protein	0	0	0	3	1	5
CDS;ID=637289103;locus_tag=SPO1621;product=TfoX domain protein	23	22	13	6	22	14
CDS;ID=637289104;locus_tag=SPO1622;product=cytochrome P450 family protein	29	16	17	16	20	23
CDS;ID=637289105;locus_tag=SPO1623;product=sensor histidine kinase	15	8	11	10	18	19
CDS;ID=637289106;locus_tag=SPO1624;product=DNA-binding response regulator	9	11	13	16	19	27
"CDS;ID=637289107;locus_tag=SPO1625;product=periplasmic serine protease, DO/DeqQ fa	11	5	6	20	9	14
CDS;ID=637289108;locus_tag=SPO1626;product=type I secretion target repeat protein	59	62	55	78	103	107
CDS;ID=637289109;locus_tag=SPO1627;product=hypothetical protein	3	8	2	7	7	9
CDS;ID=637289110;locus_tag=SPO1628;product=aminomethyl transferase family protein	17	28	17	13	31	24
CDS;ID=637289111;locus_tag=SPO1629;product=hypothetical protein	10	6	3	7	6	8
CDS;ID=637289112;locus_tag=SPO1630;product=hypothetical protein	16	6	12	2	7	10
CDS;ID=637289113;locus_tag=SPO1631;product=CBS domain protein	32	26	18	13	43	20
CDS;ID=637289114;locus_tag=SPO1632;product=tyrosine recombinase XerD	25	16	9	16	23	16
CDS;ID=637289115;locus_tag=SPO1633;product=hypothetical protein	54	36	46	44	46	51

CDS;ID=637289116;locus_tag=SPO1634;product=shikimate kinase	196	153	189	184	273	214
CDS;ID=637289117;locus_tag=SPO1635;product=3-dehydroquinate synthase	55	53	45	50	55	70
CDS;ID=637289118;locus_tag=SPO1636;product=SREBP protease/CBS domain	12	8	9	17	16	15
CDS;ID=637289119;locus_tag=SPO1637;product=single-strand binding protein	177	134	145	120	141	144
CDS;ID=637289120;locus_tag=SPO1638;product=transglycosylase SLT domain protein	83	57	57	55	56	82
CDS;ID=637289121;locus_tag=SPO1639;product=fadB domain protein	33	29	34	15	20	48
"CDS;ID=637289122;locus_tag=SPO1640;product=transcriptional regulator, LysR family'	8	10	22	10	11	7
CDS;ID=637289123;locus_tag=SPO1641;product=beta-lactamase	4	6	7	3	4	5
CDS;ID=637289124;locus_tag=SPO1642;product=hypothetical protein	2	1	3	2	0	0
"CDS;ID=637289125;locus_tag=SPO1643;product=selenium-binding protein, putative"	26	17	32	16	14	26
"CDS;ID=637289126;locus_tag=SPO1644;product=oligopeptide/dipeptide ABC transporter,	2	2	3	1	2	2
"CDS;ID=637289127;locus_tag=SPO1645;product=oligopeptide/dipeptide ABC transporter,	3	0	2	1	3	1
"CDS;ID=637289128;locus_tag=SPO1646;product=oligopeptide/dipeptide ABC transporter,	3	0	0	2	0	0
"CDS;ID=637289129;locus_tag=SPO1647;product=oligopeptide/dipeptide ABC transporter,	5	4	1	1	11	1
CDS;ID=637289130;locus_tag=SPO1648;product=aminomethyl transferase family protein	6	3	5	1	3	1
CDS;ID=637289131;locus_tag=SPO1649;product=invasion protein IbeA	1	3	2	2	4	2
CDS;ID=637289132;locus_tag=SPO1650;product=hypothetical protein	0	0	0	0	0	0
CDS;ID=637289133;locus_tag=SPO1651;product=hypothetical protein	4	6	2	0	4	2
"CDS;ID=637289134;locus_tag=SPO1652;product=transposase, truncation"	1	4	3	5	5	5
CDS;ID=640735059;locus_tag=SPO1653	9	2	1	1	6	6
CDS;ID=640735060;locus_tag=SPO1654	5	3	0	0	0	2
CDS;ID=637289135;locus_tag=SPO1655;product=hypothetical protein	6	1	7	3	6	13
"CDS;ID=637289136;locus_tag=SPO1656;product=oligopeptide/dipeptide ABC transporter,	22	10	15	11	17	15
"CDS;ID=637289137;locus_tag=SPO1657;product=oligopeptide/dipeptide ABC transporter,	18	22	14	11	16	20
"CDS;ID=637289138;locus_tag=SPO1658;product=oligopeptide/dipeptide ABC transporter,	17	19	19	15	15	19
"CDS;ID=637289139;locus_tag=SPO1659;product=oligopeptide/dipeptide ABC transporter,	110	121	97	73	116	80
"CDS;ID=637289140;locus_tag=SPO1660;product=transcriptional regulator, AraC family'	12	15	11	10	14	14
CDS;ID=637289141;locus_tag=SPO1661;product=tRNA delta(2)-isopentenylpyrophosphate t	0	5	3	3	5	8
CDS;ID=637289142;locus_tag=SPO1662;product=uridylate kinase	145	117	97	100	112	164
CDS;ID=637289143;locus_tag=SPO1663;product=hypothetical protein	70	70	37	75	59	48
CDS;ID=637289144;locus_tag=SPO1664;product=ribosome recycling factor	235	222	201	216	293	253
CDS;ID=637289145;locus_tag=SPO1665;product=undecaprenyl diphosphate synthase	30	31	20	36	28	26
CDS;ID=637289146;locus_tag=SPO1666;product=phosphatidate cytidylyltransferase	55	44	37	42	46	46
CDS;ID=637289147;locus_tag=SPO1667;product=1-deoxy-D-xylulose 5-phosphate reductois	66	47	50	44	46	61
"CDS;ID=637289148;locus_tag=SPO1668;product=membrane-associated zinc metalloproteas	52	39	42	27	39	44
CDS;ID=637289149;locus_tag=SPO1669;product=hypothetical protein	57	58	29	51	44	32
"CDS;ID=637289150;locus_tag=SPO1670;product=outer membrane protein, OMP85 family"	553	516	506	644	413	622
CDS;ID=637289151;locus_tag=SPO1671;product=hypothetical protein	19	13	5	5	11	15
CDS;ID=637289152;locus_tag=SPO1672;product=beta-hydroxyacyl-(acyl-carrier-protein)	95	82	104	84	97	117
CDS;ID=637289153;locus_tag=SPO1673;product=UDP-N-acetylglucosamine acyltransferase	36	42	39	34	38	40
CDS;ID=637289154;locus_tag=SPO1674;product=hypothetical protein	28	20	18	15	23	18
CDS;ID=637289155;locus_tag=SPO1675;product=lipid-A-disaccharide synthase	33	31	31	27	16	42
"CDS;ID=637289156;locus_tag=SPO1676;product=acetyltransferase, GNAT family"	6	7	8	12	9	5

CDS;ID=637289157;locus_tag=SPO1677;product=tRNA (5-methylaminomethyl-2-thiouridylat	23	30	15	33	16	16
CDS;ID=637289158;locus_tag=SPO1678;product=hypothetical protein	1	0	1	1	1	1
CDS;ID=637289159;locus_tag=SPO1679;product=DNA-binding response regulator CtrA	46	34	33	27	31	25
"CDS;ID=637289160;locus_tag=SPO1680;product=DNA ligase, NAD-dependent"	79	80	52	62	63	90
CDS;ID=637289161;locus_tag=SPO1681;product=ATP-dependent DNA helicase RecG	38	45	23	34	47	27
CDS;ID=637289162;locus_tag=SPO1682;product=hypothetical protein	11	7	3	3	3	3
CDS;ID=637289163;locus_tag=SPO1683;product=hypothetical protein	21	7	14	16	23	25
CDS;ID=637289164;locus_tag=SPO1684;product=phosphoribosyl-AMP cyclohydrolase	18	12	7	23	23	13
"CDS;ID=637289165;locus_tag=SPO1685;product=tRNA synthetase, class I family protein	9	10	7	14	15	15
CDS;ID=637289166;locus_tag=SPO1686;product=glucose-inhibited division protein A	51	27	33	47	46	36
CDS;ID=637289167;locus_tag=SPO1687;product=enoyl-CoA hydratase	21	10	19	13	20	23
CDS;ID=637289168;locus_tag=SPO1688;product=thioesterase family protein	14	10	7	4	9	11
CDS;ID=637289169;locus_tag=SPO1689;product=hypothetical protein	10	9	14	16	11	12
"CDS;ID=637289170;locus_tag=SPO1690;product=transcriptional regulator, GntR family"	9	9	12	5	4	12
CDS;ID=637289171;locus_tag=SPO1691;product=50S ribosomal protein L13	359	302	355	362	602	318
CDS;ID=637289172;locus_tag=SPO1692;product=30S ribosomal protein S9	73	61	45	59	79	54
CDS;ID=637289173;locus_tag=SPO1693;product=hypothetical protein	294	336	257	211	271	305
"CDS;ID=637289174;locus_tag=SPO1694;product=carbohydrate kinase, pfkB family"	115	146	129	118	111	134
"CDS;ID=637289175;locus_tag=SPO1695;product=hydantoin utilization protein, putative	29	28	16	21	16	24
CDS;ID=637289176;locus_tag=SPO1696;product=hydantoinase/oxoprolinase family protein	20	13	13	12	29	14
"CDS;ID=637289177;locus_tag=SPO1697;product=aminotransferase, classes I and II"	5	6	3	6	6	2
CDS;ID=637289178;locus_tag=SPO1698;product=Asp/Glu/Hydantoin racemase family prote	7	10	5	2	3	2
CDS;ID=637289179;locus_tag=SPO1699;product=MmgE/PrpD family protein	11	11	10	6	11	4
CDS;ID=637289180;locus_tag=SPO1700;product=D-isomer specific 2-hydroxyacid dehydrog	17	11	10	18	23	24
CDS;ID=637289181;locus_tag=SPO1701;product=dihydrodipicolinate synthase family prot	5	8	14	9	8	8
CDS;ID=637289182;locus_tag=SPO1702;product=hypothetical protein	13	14	20	12	20	25
CDS;ID=637289183;locus_tag=SPO1703;product=CaiB/BaiF family protein	9	12	19	6	13	13
"CDS;ID=637289184;locus_tag=SPO1704;product=transcriptional regulator, AraC family"	22	13	20	7	8	16
"CDS;ID=637289185;locus_tag=SPO1705;product=phage head morphogenesis domain protein	14	22	16	10	27	19
CDS;ID=640735061;locus_tag=SPO1706	2	5	4	0	4	1
"CDS;ID=637289186;locus_tag=SPO1707;product=branched-chain amino acid ABC transport	2	4	1	1	4	6
"CDS;ID=637289187;locus_tag=SPOA0447;product=branched-chain amino acid ABC transpor	0	3	4	1	9	5
"CDS;ID=637289188;locus_tag=SPO1708;product=branched-chain amino acid ABC transport	9	9	8	17	17	22
"CDS;ID=637289189;locus_tag=SPO1709;product=branched-chain amino acid ABC transport	3	1	3	4	8	4
"CDS;ID=637289190;locus_tag=SPO1710;product=branched-chain amino acid ABC transport	1	1	0	4	2	1
CDS;ID=637289191;locus_tag=SPO1711;product=urease accessory protein UreD	0	0	0	0	2	1
"CDS;ID=637289192;locus_tag=SPO1712;product=urease, gamma subunit"	1	3	0	0	1	1
"CDS;ID=637289193;locus_tag=SPO1713;product=urease, beta subunit"	0	0	0	2	0	2
"CDS;ID=637289194;locus_tag=SPO1714;product=urease, alpha subunit"	6	9	5	5	7	8
CDS;ID=637289195;locus_tag=SPO1715;product=urease accessory protein ureE	2	1	1	1	0	1
CDS;ID=637289196;locus_tag=SPO1716;product=urease accessory protein UreF	2	4	3	3	6	0
CDS;ID=637289197;locus_tag=SPO1717;product=urease accessory protein UreG	0	0	0	2	2	0
CDS;ID=637289198;locus_tag=SPO1718;product=hypothetical protein	22	20	17	18	33	23

"CDS;ID=637289199;locus_tag=SPO3897;product=site-specific recombinase, resolvase fa	7	10	13	18	13	10
"CDS;ID=637289200;locus_tag=SPO1719;product=TRAP dicarboxylate transporter, DctM su	5	6	6	2	13	1
"CDS;ID=637289201;locus_tag=SPO1720;product=TRAP dicarboxylate transporter, DctQ su	1	2	1	2	8	5
"CDS;ID=637289202;locus_tag=SPO1721;product=TRAP dicarboxylate transporter, DctP su	12	9	3	11	6	8
"CDS;ID=637289203;locus_tag=SPO1722;product=transcriptional regulator, GntR family"	13	5	3	15	16	27
CDS;ID=637289204;locus_tag=SPO1723;product=mannonate dehydratase	4	5	2	3	5	6
CDS;ID=637289205;locus_tag=SPO1724;product=D-mannonate oxidoreductase	3	1	4	8	5	8
CDS;ID=637289206;locus_tag=SPO1725;product=hypothetical protein	41	52	37	33	33	76
"CDS;ID=637289207;locus_tag=SPO3896;product=transcriptional regulator, TetR family"	17	10	8	8	8	6
"CDS;ID=637289208;locus_tag=SPO1727;product=polyphosphate kinase 2, putative"	57	46	34	27	39	26
"CDS;ID=637289209;locus_tag=SPO1728;product=esterase, putative"	20	19	8	18	20	14
CDS;ID=637289210;locus_tag=SPO1729;product=homoserine O-succinyltransferase	63	66	72	70	82	82
tRNA;ID=640698528;locus_tag=SPO_tRNA-Glu-2	405	228	111	115	30	151
tRNA;ID=640698529;locus_tag=SPO_tRNA-Glu-3	414	234	134	133	46	174
CDS;ID=637289213;locus_tag=SPO1732;product=single-stranded-DNA-specific exonuclease	26	12	21	18	36	15
"CDS;ID=637289214;locus_tag=SPO1733;product=fructose-1,6-bisphosphatase, class II"	132	122	82	116	137	130
CDS;ID=637289215;locus_tag=SPO1734;product=homoserine dehydrogenase	56	47	42	55	70	90
CDS;ID=637289216;locus_tag=SPO1735;product=hypothetical protein	35	38	49	43	73	51
"CDS;ID=637289217;locus_tag=SPO1736;product=transcriptional regulator, TetR family"	95	106	72	88	88	86
CDS;ID=637289218;locus_tag=SPO1737;product=pirin domain protein	27	18	10	9	16	15
"CDS;ID=637289219;locus_tag=SPO1738;product=dehalogenase, putative"	10	8	7	3	6	2
CDS;ID=637289220;locus_tag=SPO1739;product=hypothetical protein	24	26	26	17	21	18
CDS;ID=637289221;locus_tag=SPO1740;product=hypothetical protein	11	7	8	15	8	12
CDS;ID=637289222;locus_tag=SPO1741;product=hypothetical protein	25	14	10	12	10	6
CDS;ID=637289223;locus_tag=SPO1742;product=hypothetical protein	80	62	34	68	63	60
CDS;ID=637289224;locus_tag=SPO1743;product=glutamate dehydrogenase	402	379	328	240	265	280
"CDS;ID=637289225;locus_tag=SPO1744;product=sarcosine oxidase, beta subunit family"	34	41	34	39	48	49
"CDS;ID=637289226;locus_tag=SPO1745;product=sarcosine oxidase, delta subunit family	3	2	2	0	4	2
"CDS;ID=637289227;locus_tag=SPO1746;product=sarcosine oxidase, alpha subunit family	36	39	29	34	40	48
"CDS;ID=637289228;locus_tag=SPO1747;product=sarcosine oxidase, gamma subunit family	1	1	0	3	4	2
CDS;ID=637289229;locus_tag=SPO1748;product=hypothetical protein	21	18	20	21	21	16
CDS;ID=637289230;locus_tag=SPO1749;product=DNA primase	132	121	95	114	123	147
CDS;ID=637289231;locus_tag=SPO1750;product=RNA polymerase sigma factor	232	221	182	185	272	183
CDS;ID=637289232;locus_tag=SPO1751;product=hypothetical protein	24	21	31	24	42	37
CDS;ID=637289233;locus_tag=SPO1752;product=conserved hypothetical protein TIGR0014	4	5	7	6	9	7
CDS;ID=637289234;locus_tag=SPO1753;product=hypothetical protein	43	46	48	42	35	30
CDS;ID=637289235;locus_tag=SPO1754;product=riboflavin biosynthesis protein RibD	17	4	4	6	7	7
CDS;ID=637289236;locus_tag=SPO1755;product=capsular polysaccharide export protein	14	5	7	11	5	7
CDS;ID=637289237;locus_tag=SPO1756;product=polysaccharide biosynthesis/export prote	51	43	35	69	66	66
CDS;ID=637289238;locus_tag=SPO1757;product=capsular polysaccharide export protein	14	14	13	14	29	17
CDS;ID=637289239;locus_tag=SPO1758;product=hypothetical protein	5	5	4	9	16	7
CDS;ID=637289240;locus_tag=SPO1759;product=riboflavin synthase subunit alpha	28	23	14	26	29	23
CDS;ID=637289241;locus_tag=SPO1760;product=hypothetical protein	6	3	3	3	8	3

"CDS;ID=637289242;locus_tag=SPO1761;product=3,4-dihydroxy-2-butanone 4-phosphate sy	55	60	46	41	82	55
"CDS;ID=637289243;locus_tag=SPO1762;product=riboflavin synthase, beta subunit"	30	36	23	40	39	41
CDS;ID=637289244;locus_tag=SPO1763;product=transcription antitermination factor Nus	23	18	18	24	17	24
"CDS;ID=637289245;locus_tag=SPO1764;product=transcriptional regulator, LuxR family"	21	11	12	11	11	19
CDS;ID=637289246;locus_tag=SPO1765;product=hypothetical protein	15	14	10	9	8	7
RNA;ID=643672546;locus_tag=SPO_RF0072;product=6S	325	307	191	165	148	207
CDS;ID=637289247;locus_tag=SPO1766;product=hypothetical protein	29	38	24	9	16	19
"CDS;ID=637289248;locus_tag=SPO1767;product=acetyltransferase, GNAT family"	5	10	6	6	2	4
tRNA;ID=640698530;locus_tag=SPO_tRNA-His-1	129	44	39	33	3	43
CDS;ID=637289250;locus_tag=SPO1769;product=hypothetical protein	59	69	59	70	59	70
CDS;ID=637289251;locus_tag=SPO1770;product=hypothetical protein	22	16	14	14	18	14
"CDS;ID=637289252;locus_tag=SPO1771;product=TRAP dicarboxylate transporter, DctM su	10	2	9	3	5	5
"CDS;ID=637289253;locus_tag=SPO1772;product=TRAP dicarboxylate transporter, DctQ su	13	7	2	0	4	3
"CDS;ID=637289254;locus_tag=SPO1773;product=TRAP dicarboxylate transporter, DctP su	17	16	7	9	14	17
"CDS;ID=637289255;locus_tag=SPO1774;product=3-hydroxyanthranilate 3,4-dioxygenase"	5	6	6	5	10	10
CDS;ID=637289256;locus_tag=SPO1775;product=hypothetical protein	7	8	2	12	7	2
CDS;ID=637289257;locus_tag=SPO1776;product=pyridine nucleotide-disulphide oxidoredu	434	452	406	174	148	120
CDS;ID=637289258;locus_tag=SPO1777;product=dihydropyrimidine dehydrogenase	372	302	261	98	136	121
CDS;ID=637289259;locus_tag=SPO1778;product=hypothetical protein	3	6	3	3	1	1
"CDS;ID=637289260;locus_tag=SPO1779;product=membrane protein, major facilitator tra	36	28	22	26	24	28
"CDS;ID=637289261;locus_tag=SPO1780;product=transcriptional regulator, TetR family"	9	19	7	9	4	8
CDS;ID=637289262;locus_tag=SPO1781;product=N-carbamoyl-L-amino acid amidohydrolase	142	163	107	51	71	79
CDS;ID=637289263;locus_tag=SPO1782;product=hypothetical protein	65	51	51	25	38	31
CDS;ID=637289264;locus_tag=SPO1783;product=dihydropyrimidinase	83	81	56	47	60	36
CDS;ID=637289265;locus_tag=SPO1784;product=hypothetical protein	30	32	31	25	26	21
"CDS;ID=637289266;locus_tag=SPO1785;product=ABC transporter, ATP-binding protein"	98	56	72	63	66	66
"CDS;ID=637289267;locus_tag=SPO1786;product=ABC transporter, permease protein"	42	50	30	20	31	24
"CDS;ID=637289268;locus_tag=SPO1787;product=ABC transporter, permease protein"	38	41	21	22	23	22
"CDS;ID=637289269;locus_tag=SPO1788;product=ABC transporter, periplasmic substrate-	103	85	74	60	58	49
"CDS;ID=637289270;locus_tag=SPO1789;product=sulfate/tungstate uptake family ABC tra	113	100	65	66	105	105
"CDS;ID=637289271;locus_tag=SPO1790;product=sulfate/tungstate uptake family ABC tra	73	62	60	72	88	75
"CDS;ID=637289272;locus_tag=SPO1791;product=sulfate/tungstate uptake family ABC tra	121	86	82	80	74	82
"CDS;ID=637289273;locus_tag=SPO1792;product=DNA-binding protein, excisionase family	12	19	8	6	8	20
CDS;ID=637289274;locus_tag=SPO1793;product=WD domain/cytochrome c family protein	13	9	2	5	0	5
"CDS;ID=637289275;locus_tag=SPO1794;product=formate dehydrogenase, gamma subunit"	13	5	3	1	1	3
"CDS;ID=637289276;locus_tag=SPO1795;product=formate dehydrogenase, iron-sulfur subu	6	10	2	6	5	2
"CDS;ID=637289277;locus_tag=SPO1796;product=formate dehydrogenase, alpha subunit, p	17	15	21	15	24	21
CDS;ID=637289278;locus_tag=SPO1797;product=twin-arginine translocation pathway sigr	8	3	3	3	3	2
"CDS;ID=637289279;locus_tag=SPO1798;product=chaperone, TorD family"	2	4	1	5	2	6
CDS;ID=637289280;locus_tag=SPO1799;product=hypothetical protein	5	4	0	2	2	0
CDS;ID=637289281;locus_tag=SPO1800;product=iron-sulfur cluster-binding protein	10	12	5	6	12	13
CDS;ID=637289282;locus_tag=SPO1801;product=hypothetical protein	21	26	30	35	26	43
CDS;ID=637289283;locus_tag=SPO1802;product=hypothetical protein	26	26	17	22	47	27

CDS;ID=637289284;locus_tag=SPO1803;product=Mrp/NBP35 family protein	25	36	41	17	32	34
CDS;ID=637289285;locus_tag=SPO1804;product=hypothetical protein	26	28	22	18	45	43
CDS;ID=637289286;locus_tag=SPO1805;product=hypothetical protein	14	18	17	10	18	21
CDS;ID=637289287;locus_tag=SPO1806;product=flavin reductase domain protein	2	3	2	0	3	1
"CDS;ID=637289288;locus_tag=SPO1807;product=exonuclease, DNA polymerase III, epsilo	7	12	4	12	14	12
CDS;ID=637289289;locus_tag=SPO1808;product=hypothetical protein	8	3	6	6	6	6
CDS;ID=637289290;locus_tag=SPO1809;product=nucleotidyltransferase/CBS/cyclic nucle	17	14	11	6	3	11
CDS;ID=637289291;locus_tag=SPO1810;product=sodium/solute symporter family protein	25	18	10	10	14	13
CDS;ID=637289292;locus_tag=SPO1811;product=hypothetical protein	2	1	1	1	1	1
CDS;ID=637289293;locus_tag=SPO1812;product=adenylate kinase	21	3	8	8	9	11
CDS;ID=637289294;locus_tag=SPO1813;product=acetyl-coenzyme A synthetase	52	37	33	42	49	38
"CDS;ID=637289295;locus_tag=SPO1814;product=TRAP dicarboxylate transporter, DctP su	95	43	50	66	59	71
"CDS;ID=637289296;locus_tag=SPO1815;product=TRAP dicarboxylate transporter, DctQ su	2	2	7	7	5	5
"CDS;ID=637289297;locus_tag=SPO1816;product=TRAP dicarboxylate transporter, DctM su	8	4	8	5	3	18
CDS;ID=637289298;locus_tag=SPO1817;product=hypothetical protein	4	9	2	5	1	4
CDS;ID=637289299;locus_tag=SPO1818;product=hypothetical protein	1	8	3	6	10	14
CDS;ID=637289300;locus_tag=SPO1819;product=phosphodiesterase	0	0	1	0	0	0
"CDS;ID=637289301;locus_tag=SPO1820;product=sugar ABC transporter, periplasmic suga	3	4	0	2	4	5
"CDS;ID=637289302;locus_tag=SPO1821;product=sugar ABC transporter, permease protein	1	1	1	0	2	1
"CDS;ID=637289303;locus_tag=SPO1822;product=sugar ABC transporter, permease protein	0	1	1	2	1	0
"CDS;ID=637289304;locus_tag=SPO1823;product=sugar ABC transporter, ATP-binding prot	3	4	0	0	2	0
"CDS;ID=637289305;locus_tag=SPO1824;product=transcriptional regulator, LysR family"	2	1	2	2	2	4
CDS;ID=637289306;locus_tag=SPO1825;product=glutamine synthetase family protein	95	65	87	90	96	104
"CDS;ID=637289307;locus_tag=SPO1826;product=transcriptional regulator, RpiR family"	132	134	141	164	157	146
CDS;ID=637289308;locus_tag=SPO1827;product=N-formylglutamate amidohydrolase	51	56	51	62	66	72
CDS;ID=637289309;locus_tag=SPO1828;product=isochorismatase family protein	76	69	68	31	49	58
"CDS;ID=637289310;locus_tag=SPO1829;product=branched-chain amino acid ABC transport	287	319	238	204	250	267
"CDS;ID=637289311;locus_tag=SPO1830;product=branched-chain amino acid ABC transport	139	145	97	89	143	114
"CDS;ID=637289312;locus_tag=SPO1831;product=branched-chain amino acid ABC transport	26	40	24	15	35	22
"CDS;ID=637289313;locus_tag=SPO1832;product=branched-chain amino acid ABC transport	34	46	21	33	28	32
"CDS;ID=637289314;locus_tag=SPO1833;product=branched-chain amino acid ABC transport	78	69	52	48	65	56
CDS;ID=637289315;locus_tag=SPO1834;product=acetamidase/formamidase family protein	69	41	36	36	44	45
"CDS;ID=637289316;locus_tag=SPO1835;product=sugar ABC transporter, ATP-binding prot	13	10	7	5	9	6
"CDS;ID=637289317;locus_tag=SPO1836;product=oxidoreductase, Gfo/Idh/MocA family"	4	2	8	4	4	2
"CDS;ID=637289318;locus_tag=SPO1837;product=sugar ABC transporter, permease protein	16	10	8	3	5	8
"CDS;ID=637289319;locus_tag=SPO1838;product=sugar ABC transporter, permease protein	3	12	4	5	5	5
"CDS;ID=637289320;locus_tag=SPO1839;product=sugar ABC transporter, periplasmic suga	28	21	23	14	23	19
CDS;ID=637289321;locus_tag=SPO1840;product=hypothetical protein	16	13	20	10	14	6
CDS;ID=637289322;locus_tag=SPO1841;product=BadF/BadG/BcrA/BcrD ATPase family protei	19	17	10	8	5	13
"CDS;ID=637289323;locus_tag=SPO1842;product=transcriptional regulator, GntR family"	1	2	1	2	1	7
CDS;ID=637289324;locus_tag=SPO1843;product=SIS domain protein	2	7	7	1	2	5
CDS;ID=637289325;locus_tag=SPO1844;product=N-acetylglucosamine-6-phosphate deacety	12	15	13	4	6	5
"CDS;ID=637289326;locus_tag=SPO1845;product=oxidoreductase, molybdopterin-binding"	4	4	1	4	0	6

"CDS;ID=637289327;locus_tag=SPO1846;product=branched-chain amino acid ABC transport	12	16	2	8	14	4
CDS;ID=637289328;locus_tag=SPO1847;product=AMP-binding enzyme	22	16	12	9	16	17
"CDS;ID=637289329;locus_tag=SPO1848;product=branched-chain amino acid ABC transport	16	13	7	8	14	22
"CDS;ID=637289330;locus_tag=SPO1849;product=branched-chain amino acid ABC transport	9	7	9	11	3	7
"CDS;ID=637289331;locus_tag=SPO1850;product=branched-chain amino acid ABC transport	2	5	3	2	2	2
"CDS;ID=637289332;locus_tag=SPO1851;product=branched-chain amino acid ABC transport	9	8	2	6	5	3
"CDS;ID=637289333;locus_tag=SPO1852;product=oxidoreductase, zinc-binding dehydroger	26	24	15	17	14	24
"CDS;ID=637289334;locus_tag=SPO1853;product=transcriptional regulator, TetR family"	11	8	2	4	16	4
CDS;ID=637289335;locus_tag=SPO1854;product=hypothetical protein	19	22	7	33	16	20
CDS;ID=637289336;locus_tag=SPO1855;product=hypothetical protein	214	224	166	155	251	171
"CDS;ID=637289337;locus_tag=SPO1856;product=ribonuclease BN, putative"	24	17	24	35	16	32
CDS;ID=637289338;locus_tag=SPO1857;product=hypothetical protein	7	3	6	3	3	4
CDS;ID=637289339;locus_tag=SPO1858;product=ribosomal RNA large subunit methyltrans	20	22	15	44	17	27
"CDS;ID=637289340;locus_tag=SPO1859;product=phosphatase, Ppx/GppA family"	89	79	77	50	32	73
CDS;ID=637289341;locus_tag=SPO1860;product=twin-arginine translocation pathway sigr	19	14	18	13	18	21
CDS;ID=637289342;locus_tag=SPO1861;product=hypothetical protein	67	33	54	52	68	74
"CDS;ID=637289343;locus_tag=SPO1862;product=metFprotein, homolog"	52	57	66	56	79	64
CDS;ID=637289344;locus_tag=SPO1863;product=hypothetical protein	48	46	53	44	59	52
CDS;ID=637289345;locus_tag=SPO1864;product=hypothetical protein	2	0	2	0	4	1
CDS;ID=637289346;locus_tag=SPO1865;product=transketolase	121	86	108	114	167	106
CDS;ID=637289347;locus_tag=SPO1866;product=hypothetical protein	198	153	150	165	171	157
CDS;ID=637289348;locus_tag=SPO1867;product=hypothetical protein	41	31	35	45	47	49
CDS;ID=637289349;locus_tag=SPO1868;product=glutaredoxin-related protein	45	41	35	50	31	38
CDS;ID=637289350;locus_tag=SPO1869;product=BolA family protein	109	88	93	121	101	76
CDS;ID=637289351;locus_tag=SPO1870;product=phosphoribosylformylglycinamidine syntha	86	93	81	113	120	108
CDS;ID=637289352;locus_tag=SPO1871;product=mechanosensitive ion channel family prot	49	36	46	21	36	26
"CDS;ID=637289353;locus_tag=SPO1872;product=transcriptional regulator, LysR family"	21	17	15	15	32	25
CDS;ID=637289354;locus_tag=SPO1873;product=indolepyruvate ferredoxin oxidoreductase	204	222	125	139	171	165
CDS;ID=637289355;locus_tag=SPO1874;product=acyltransferase domain protein	41	28	16	27	26	30
CDS;ID=637289356;locus_tag=SPO1875;product=glutamate racemase	62	54	42	49	62	67
CDS;ID=637289357;locus_tag=SPO1876;product=N-acetyl-gamma-glutamyl-phosphate reduct	58	52	60	74	65	64
CDS;ID=637289358;locus_tag=SPO1877;product=cytochrome c-type biogenesis protein Ccr	88	91	99	99	100	95
CDS;ID=637289359;locus_tag=SPO1878;product=hypothetical protein	1	0	0	0	3	1
CDS;ID=637289360;locus_tag=SPO1879;product=hypothetical protein	0	2	1	0	1	1
CDS;ID=637289361;locus_tag=SPO1880;product=cytochrome c-type biogenesis protein Ccr	33	33	35	24	48	41
CDS;ID=637289362;locus_tag=SPO1881;product=cytochrome c biogenesis family protein	1	9	7	4	5	8
CDS;ID=637289363;locus_tag=SPO1882;product=enoyl-CoA hydratase/isomerase family pro	37	28	37	37	36	46
CDS;ID=637289364;locus_tag=SPO1883;product=hypothetical protein	7	12	5	4	7	9
CDS;ID=637289365;locus_tag=SPO1884;product=methionine synthase I	19	17	19	13	16	29
CDS;ID=637289366;locus_tag=SPO1885;product=hypothetical protein	2	1	1	5	3	5
CDS;ID=637289367;locus_tag=SPO1886;product=hypothetical protein	161	121	129	105	156	170
CDS;ID=637289368;locus_tag=SPO1887;product=phosphoribosylaminoimidazole-succinocarb	137	123	125	149	184	125
"CDS;ID=637289369;locus_tag=SPO1888;product=phosphoribosylformylglycinamidine synth	42	52	45	55	40	49

"CDS;ID=637289370;locus_tag=SPO1889;product=alcohol dehydrogenase, zinc-containing'	46	41	42	54	55	54
CDS;ID=637289371;locus_tag=SPO1890;product=phosphoribosylformylglycinamidine syntha	92	107	108	85	91	68
"CDS;ID=637289372;locus_tag=SPO1891;product=C4-dicarboxylate transport sensor prote	206	149	173	145	159	189
CDS;ID=637289373;locus_tag=SPO1892;product=C4-dicarboxylate transport transcriptior	336	295	277	219	266	259
"CDS;ID=637289374;locus_tag=SPO1893;product=ribonuclease, Rne/Rng family"	649	552	468	418	748	554
CDS;ID=637289375;locus_tag=SPO1895;product=SirA family protein	9	28	15	17	29	16
CDS;ID=637289376;locus_tag=SPO1896;product=cytochrome c-type biogenesis protein Ccc	36	18	14	27	24	33
CDS;ID=637289377;locus_tag=SPO1897;product=hypothetical protein	17	7	6	9	8	13
CDS;ID=637289378;locus_tag=SPO1898;product=cytochrome P450 family protein	16	15	20	19	12	14
CDS;ID=637289379;locus_tag=SPO1899;product=fructose-bisphosphate aldolase	59	59	71	62	81	67
CDS;ID=637289380;locus_tag=SPO1900;product=DNA-3-methyladenine glycosylase I	4	2	7	2	3	5
CDS;ID=637289381;locus_tag=SPO1901;product=hypothetical protein	2	0	4	0	2	0
"CDS;ID=637289382;locus_tag=SPO1902;product=transcription regulator, TetR family"	8	8	11	14	9	13
CDS;ID=637289383;locus_tag=SPO1903;product=hypothetical protein	0	1	0	1	2	2
CDS;ID=637289384;locus_tag=SPO1904;product=hypothetical protein	2	2	7	1	2	2
"CDS;ID=637289385;locus_tag=SPO1905;product=fumarate hydratase, class II"	25	10	11	13	17	18
CDS;ID=637289386;locus_tag=SPO1906;product=hypothetical protein	51	27	22	23	21	33
CDS;ID=637289387;locus_tag=SPO1907;product=hypothetical protein	161	166	138	156	116	162
CDS;ID=637289388;locus_tag=SPO1908;product=chromate transporter	19	30	10	11	9	15
CDS;ID=637289389;locus_tag=SPO1909;product=hypothetical protein	13	9	11	4	5	9
RNA;ID=640698531;locus_tag=SPO_SptmRNA1	964	710	393	472	255	534
CDS;ID=637289390;locus_tag=SPO1911;product=hypothetical protein	2	0	0	2	0	0
"CDS;ID=637289391;locus_tag=SPO1912;product=transcriptional regulator, GntR family'	5	14	3	1	4	6
CDS;ID=637289392;locus_tag=SPO1913;product=aminomethyl transferase family protein	15	14	19	19	26	12
"CDS;ID=637289393;locus_tag=SPO1914;product=oxidoreductase, zinc-binding dehydroger	24	19	16	18	23	16
CDS;ID=637289394;locus_tag=SPO1915;product=glyoxalase family protein	3	1	1	0	1	1
"CDS;ID=637289395;locus_tag=SPO1916;product=aminotransferase, class V"	175	158	176	170	157	164
CDS;ID=637289396;locus_tag=SPO1917;product=deoxyribodipyrimidine photolyase	27	19	18	16	23	18
CDS;ID=637289397;locus_tag=SPO1918;product=cyclopropane-fatty-acyl-phospholipid syr	78	53	66	53	49	55
CDS;ID=637289398;locus_tag=SPO1919;product=tellurite resistance protein	32	20	18	7	15	26
CDS;ID=637289399;locus_tag=SPO1920;product=tellurite resistance protein	58	66	40	49	54	41
CDS;ID=637289400;locus_tag=SPO1921;product=pyridoxal-phosphate dependent enzyme	157	125	112	139	182	129
CDS;ID=637289401;locus_tag=SPO1922;product=mechanosensitive ion channel family prot	100	87	83	87	103	119
CDS;ID=637289402;locus_tag=SPO1923;product=hypothetical protein	36	26	26	35	32	24
tRNA;ID=640698532;locus_tag=SPO_tRNA-Ser-2	1113	590	285	318	64	265
CDS;ID=637289404;locus_tag=SPO1925;product=hypothetical protein	47	36	29	30	27	22
CDS;ID=637289405;locus_tag=SPO1926;product=hypothetical protein	104	121	98	108	107	104
"CDS;ID=637289406;locus_tag=SPO3898;product=DNA-binding protein, putative"	30	22	33	16	19	27
"CDS;ID=637289407;locus_tag=SPO1927;product=site-specfic recombinase, phage integra	97	79	77	112	84	109
CDS;ID=637289408;locus_tag=SPO1928;product=Tat (twin-arginine translocation) pathwa	242	155	149	128	84	148
"CDS;ID=637289409;locus_tag=SPO1929;product=peptidoglycan binding protein, putative	95	60	64	72	86	104
CDS;ID=637289410;locus_tag=SPO1930;product=UDP-3-O-3-hydroxymyristoyl glucosamine N	82	59	58	71	98	85
"CDS;ID=637289411;locus_tag=SPO1931;product=acyl carrier protein, putative"	39	39	26	34	30	28

CDS;ID=637289412;locus_tag=SPO1932;product=beta-ketoacyl synthase family protein	31	26	37	30	34	37
CDS;ID=637289413;locus_tag=SPO1933;product=invasion associated family protein	139	76	119	89	169	63
CDS;ID=637289414;locus_tag=SPO1934;product=hypothetical protein	23	32	12	18	14	13
"CDS;ID=637289415;locus_tag=SPO1935;product=branched-chain amino acid ABC transport	26	19	27	27	10	22
"CDS;ID=637289416;locus_tag=SPO1936;product=branched-chain amino acid ABC transport	11	16	12	11	10	18
"CDS;ID=637289417;locus_tag=SPO1937;product=branched-chain amino acid ABC transport	5	9	10	8	11	16
"CDS;ID=637289418;locus_tag=SPO1938;product=branched-chain amino acid ABC transport	49	38	30	48	42	42
"CDS;ID=637289419;locus_tag=SPO1939;product=branched-chain amino acid ABC transport	230	235	174	176	222	208
CDS;ID=637289420;locus_tag=SPO1940;product=hypothetical protein	5	6	2	3	5	4
"CDS;ID=637289421;locus_tag=SPO1941;product=transcriptional regulator, AraC family"	182	192	166	160	202	288
CDS;ID=637289422;locus_tag=SPO1942;product=3-deoxy-7-phosphoheptulonate synthase	214	205	168	221	160	218
CDS;ID=637289423;locus_tag=SPO1943;product=hypothetical protein	5	10	2	4	3	7
CDS;ID=637289424;locus_tag=SPO1944;product=yicC family protein	45	42	42	31	56	52
CDS;ID=637289425;locus_tag=SPO1945;product=guanylate kinase	23	17	25	22	33	18
CDS;ID=637289426;locus_tag=SPO1946;product=bacterial transferase family protein	16	7	11	9	6	11
"CDS;ID=637289427;locus_tag=SPO1947;product=phosphate regulon sensor histidine kina	11	11	8	4	9	6
"CDS;ID=637289428;locus_tag=SPO1948;product=phosphate ABC transporter, periplasmic	3	10	10	17	18	15
"CDS;ID=637289429;locus_tag=SPO1949;product=phosphate ABC transporter, permease pro	10	12	1	2	14	9
"CDS;ID=637289430;locus_tag=SPO1950;product=phosphate ABC transporter, permease pro	25	19	17	17	16	19
"CDS;ID=637289431;locus_tag=SPO1951;product=phosphate ABC transporter, ATP-binding	22	33	15	13	14	11
CDS;ID=637289432;locus_tag=SPO1952;product=phosphate transport system regulatory pr	13	10	8	5	21	19
CDS;ID=637289433;locus_tag=SPO1953;product=phosphate regulon transcriptional regula	19	15	3	13	8	7
"CDS;ID=637289434;locus_tag=SPO1954;product=transcriptional regulator, LysR family"	6	6	8	5	6	12
CDS;ID=637289435;locus_tag=SPO1955;product=glutaryl-CoA dehydrogenase	48	29	32	23	29	18
CDS;ID=637289436;locus_tag=SPO1956;product=sulfate permease	4	2	7	7	3	5
"CDS;ID=637289437;locus_tag=SPO1957;product=oxidoreductase, short chain dehydrogena	13	15	6	8	2	9
CDS;ID=637289438;locus_tag=SPO1958;product=acyl-CoA thioesterase II	18	10	11	17	9	22
CDS;ID=637289439;locus_tag=SPO1959;product=acetoacetyl-CoA synthase	43	37	19	36	23	45
CDS;ID=637289440;locus_tag=SPO1960;product=beta-lactamase	43	15	22	15	19	14
"CDS;ID=637289441;locus_tag=SPO1961;product=transcriptional regulator, IclR family"	20	2	1	7	6	4
CDS;ID=637289442;locus_tag=SPO1962;product=phosphotransferase family protein	9	7	3	6	10	6
CDS;ID=637289443;locus_tag=SPO1963;product=phosphoglycerate mutase family protein	3	2	2	4	11	2
CDS;ID=637289444;locus_tag=SPO1964;product=hypothetical protein	10	3	7	4	7	5
"CDS;ID=637289445;locus_tag=SPO1965;product=oxidoreductase, short chain dehydrogena	10	7	8	10	5	11
"CDS;ID=637289446;locus_tag=SPO1966;product=oxidoreductase, short chain dehydrogena	6	7	9	8	13	10
"CDS;ID=637289447;locus_tag=SPO1967;product=acyl-CoA dehydrogenase, putative"	9	18	16	9	9	8
CDS;ID=637289448;locus_tag=SPO1968;product=acyl-CoA dehydrogenase family protein	35	21	22	21	25	37
"CDS;ID=637289449;locus_tag=SPO1969;product=oxidoreductase, zinc-binding dehydroger	15	28	11	12	15	21
CDS;ID=637289450;locus_tag=SPO1970;product=phosphoglycerate mutase family protein	11	10	9	8	19	7
CDS;ID=637289451;locus_tag=SPO1971;product=enoyl-CoA hydratase	1	4	3	1	0	1
CDS;ID=637289452;locus_tag=SPO1972;product=nodulation protein N	1	0	1	0	0	2
CDS;ID=637289453;locus_tag=SPO1973;product=3-dehydroquinate dehydratase	43	41	29	43	27	48
"CDS;ID=637289454;locus_tag=SPO1974;product=autoinducer-binding transcriptional reg	36	23	21	23	16	15

CDS;ID=637289455;locus_tag=SPO1975;product=elongation factor Ts	149	117	126	108	158	120
CDS;ID=637289456;locus_tag=SPO1976;product=30S ribosomal protein S2	280	217	253	277	468	302
CDS;ID=637289457;locus_tag=SPO1977;product=hypothetical protein	9	8	15	12	21	17
CDS;ID=637289458;locus_tag=SPO1978;product=hypothetical protein	7	3	6	9	3	5
"CDS;ID=637289459;locus_tag=SPO1979;product=acyltransferase, putative"	12	5	11	12	9	14
CDS;ID=637289460;locus_tag=SPO1980;product=hypothetical protein	27	18	16	26	23	30
CDS;ID=637289461;locus_tag=SPO1981;product=hypothetical protein	5	5	4	10	2	3
CDS;ID=637289462;locus_tag=SPO1982;product=hypothetical protein	13	18	7	11	10	10
CDS;ID=637289463;locus_tag=SPO1983;product=hypothetical protein	10	7	3	6	3	4
CDS;ID=637289464;locus_tag=SPO1984;product=gamma-glutamyl phosphate reductase	32	34	26	15	35	25
CDS;ID=637289465;locus_tag=SPO1985;product=gamma-glutamyl kinase	26	22	34	21	29	41
"CDS;ID=637289466;locus_tag=SPO1986;product=GTP-binding protein, GTP1/OBG family"	100	102	95	127	177	137
"CDS;ID=637289467;locus_tag=SPO1987;product=acetyltransferase, GNAT family"	31	22	40	45	63	29
"CDS;ID=637289468;locus_tag=SPO1988;product=transporter, LysE family"	35	14	47	36	60	21
CDS;ID=637289469;locus_tag=SPO1989;product=50S ribosomal protein L27	266	219	199	232	356	205
CDS;ID=637289470;locus_tag=SPO1990;product=ribosomal protein L21	446	389	419	417	528	493
CDS;ID=637289471;locus_tag=SPO1991;product=hypothetical protein	28	29	24	18	22	38
CDS;ID=637289472;locus_tag=SPO1992;product=hypothetical protein	98	87	58	80	78	77
tRNA;ID=640698533;locus_tag=SPO_tRNA-Ser-3	657	456	513	721	486	1061
"CDS;ID=637289474;locus_tag=SPO1994;product=type I secretion membrane fusion prote:	26	29	25	16	28	22
"CDS;ID=637289475;locus_tag=SPO1995;product=ABC transporter, ATP-binding/permease p	54	33	28	45	38	46
CDS;ID=637289476;locus_tag=SPO1996;product=hypothetical protein	103	103	81	77	77	100
CDS;ID=637289477;locus_tag=SPO1997;product=hypothetical protein	5	8	10	4	13	12
CDS;ID=637289478;locus_tag=SPO1998;product=hypothetical protein	9	7	5	1	3	6
CDS;ID=637289479;locus_tag=SPO1999;product=hypothetical protein	1008	823	661	656	642	610
CDS;ID=637289480;locus_tag=SPO2000;product=endoribonuclease L-PSP family protein	4	4	3	4	5	7
"CDS;ID=637289481;locus_tag=SPO2001;product=oxidoreductase, FAD-binding"	6	3	10	3	5	6
CDS;ID=637289482;locus_tag=SPO2002;product=acetylpolyamine aminohydrolase	0	1	0	0	1	1
"CDS;ID=637289483;locus_tag=SPO2003;product=acetyltransferase, GNAT family"	1	4	0	0	1	0
CDS;ID=637289484;locus_tag=SPO2004;product=hypothetical protein	1	2	4	0	5	2
"CDS;ID=637289485;locus_tag=SPO2005;product=aminotransferase, class III"	5	0	2	0	3	2
"CDS;ID=637289486;locus_tag=SPO2006;product=spermidine/putrescine ABC transporter,	1	1	3	1	1	2
"CDS;ID=637289487;locus_tag=SPO2007;product=spermidine/putrescine ABC transporter,	12	8	9	3	10	9
"CDS;ID=637289488;locus_tag=SPO2008;product=spermidine/putrescine ABC transporter,	2	6	3	4	6	4
"CDS;ID=637289489;locus_tag=SPO2009;product=spermidine/putrescine ABC transporter,	6	5	2	3	3	3
"CDS;ID=637289490;locus_tag=SPO2010;product=transcriptional regulator, LysR family'	16	6	7	14	13	13
tRNA;ID=640698534;locus_tag=SPO_tRNA-Thr-3	794	386	198	169	55	247
tRNA;ID=640698535;locus_tag=SPO_tRNA-Arg-4	109	70	45	56	21	50
CDS;ID=637289493;locus_tag=SPO2013;product=hypothetical protein	9	4	4	4	5	7
CDS;ID=637289494;locus_tag=SPO2014;product=cysteine desulfurase SufS	31	40	22	32	41	27
CDS;ID=637289495;locus_tag=SPO2015;product=hypothetical protein	4	10	14	17	12	13
CDS;ID=637289496;locus_tag=SPO2016;product=hypothetical protein	5	6	17	14	12	16
CDS;ID=637289497;locus_tag=SPO2017;product=FeS assembly protein SufD	40	43	52	49	66	45

CDS;ID=637289498;locus_tag=SPO2018;product=FeS assembly ATPase SufC	68	62	45	45	66	73
CDS;ID=637289499;locus_tag=SPO2019;product=exoV domain protein	63	68	41	38	43	55
"CDS;ID=637289500;locus_tag=SPO2020;product=methyltransferase, FkbM family"	70	54	41	59	74	78
CDS;ID=637289501;locus_tag=SPO2021;product=hypothetical protein	24	12	12	15	17	28
CDS;ID=637289502;locus_tag=SPO2022;product=hypothetical protein	14	14	14	12	29	29
CDS;ID=637289503;locus_tag=SPO2023;product=FeS assembly protein SufB	228	178	222	254	278	253
CDS;ID=640735062;locus_tag=SPO2024	98	81	92	105	98	119
"CDS;ID=637289504;locus_tag=SPO2025;product=iron-sulfur cluster assembly transcript	8	6	6	10	7	9
CDS;ID=637289505;locus_tag=SPO2026;product=hypothetical protein	107	89	78	97	117	93
CDS;ID=637289506;locus_tag=SPO2027;product=hypothetical protein	23	14	18	11	16	22
CDS;ID=637289507;locus_tag=SPO2028;product=HD domain protein	17	30	12	16	24	20
CDS;ID=637289508;locus_tag=SPO2029;product=glutamine amidotransferase class-II doma	43	26	23	19	42	22
"CDS;ID=637289509;locus_tag=SPO2030;product=multidrug resistance efflux protein, SM	0	2	2	3	0	3
CDS;ID=637289510;locus_tag=SPO2031;product=GTP-binding protein TypA	112	88	90	145	147	126
CDS;ID=637289511;locus_tag=SPO2032;product=hypothetical protein	26	25	25	23	14	19
CDS;ID=637289512;locus_tag=SPO2033;product=alanyl-tRNA synthetase	300	255	258	256	340	260
CDS;ID=637289513;locus_tag=SPO2034;product=recombinase A	292	286	244	224	201	230
CDS;ID=637289514;locus_tag=SPO2035;product=hypothetical protein	11	20	12	12	4	11
CDS;ID=637289515;locus_tag=SPO2036;product=sensory box sensor histidine kianse/resp	33	22	18	27	18	10
CDS;ID=637289516;locus_tag=SPO2037;product=NOL1/NOP2/sun family protein	45	51	46	39	36	41
CDS;ID=637289517;locus_tag=SPO2038;product=metallo-beta-lactamase family protein	16	25	10	20	17	19
CDS;ID=637289518;locus_tag=SPO2039;product=inosine-5'-monophosphate dehydrogenase	118	130	110	187	142	115
CDS;ID=637289519;locus_tag=SPO2040;product=CaiB/BaiF family protein	33	26	36	35	67	47
CDS;ID=637289520;locus_tag=SPO2041;product=iron-sulfur cluster-binding protein	44	68	41	49	48	60
"CDS;ID=637289521;locus_tag=SPO2042;product=methyltransferase, UbiE/COQ5 family"	5	2	4	2	7	9
tRNA;ID=640698536;locus_tag=SPO_tRNA-Gly-5	535	277	169	162	42	125
"CDS;ID=637289523;locus_tag=SPO2044;product=lipoprotein, putative"	53	61	49	54	60	70
CDS;ID=637289524;locus_tag=SPO2045;product=medium-chain-fatty-acid--CoA ligase	42	35	25	29	23	36
CDS;ID=637289525;locus_tag=SPO2046;product=glucose-6-phosphate isomerase	50	72	53	73	66	64
CDS;ID=637289526;locus_tag=SPO2047;product=6-phosphogluconolactonase	22	22	21	22	17	17
CDS;ID=637289527;locus_tag=SPO2048;product=glucose-6-phosphate 1-dehydrogenase	119	145	84	111	108	108
CDS;ID=637289528;locus_tag=SPO2049;product=radical SAM domain protein	33	25	30	23	11	32
"CDS;ID=637289529;locus_tag=SPO2050;product=Usg, putative"	1	2	3	2	4	2
CDS;ID=637289530;locus_tag=SPO2051;product=DNA gyrase subunit A	159	159	167	168	178	191
CDS;ID=637289531;locus_tag=SPO2052;product=hypothetical protein	11	8	5	2	17	9
CDS;ID=637289532;locus_tag=SPO2053;product=thermostable carboxypeptidase	34	41	30	29	41	44
CDS;ID=637289533;locus_tag=SPO2054;product=cytochrome c oxidase assembly protein	78	81	45	60	58	88
"CDS;ID=637289534;locus_tag=SPO2055;product=RNA methyltransferase, TrmH family, grc	39	24	21	38	34	34
"CDS;ID=637289535;locus_tag=SPO2056;product=thiamine-phosphate pyrophosphorylase, p	11	9	6	12	10	12
CDS;ID=637289536;locus_tag=SPO2057;product=hypothetical protein	12	21	9	11	10	5
CDS;ID=637289537;locus_tag=SPO2058;product=hypothetical protein	6	4	6	3	5	2
CDS;ID=637289538;locus_tag=SPO2059;product=glutathione S-transferase family protein	14	7	10	14	10	16
"CDS;ID=637289539;locus_tag=SPO2060;product=kinase, pfkB family"	23	32	20	31	22	30

CDS;ID=637289540;locus_tag=SPO2061;product=indigoidine synthase A family protein	22	26	33	26	46	31
CDS;ID=637289541;locus_tag=SPO2062;product=hypothetical protein	32	16	17	15	27	25
"CDS;ID=637289542;locus_tag=SPO2063;product=acetyltransferase, GNAT family"	25	5	8	13	9	12
"CDS;ID=637289543;locus_tag=SPO2064;product=phospholipase, patatin-like family"	28	19	19	15	22	25
CDS;ID=637289544;locus_tag=SPO2065;product=D-beta-hydroxybutyrate dehydrogenase	69	59	45	42	53	53
"CDS;ID=637289545;locus_tag=SPO2066;product=ABC transporter, periplasmic substrate-	56	56	41	49	37	59
CDS;ID=637289546;locus_tag=SPO2067;product=hypothetical protein	14	7	10	5	6	13
"CDS;ID=637289547;locus_tag=SPO2068;product=DNA-binding protein, putative"	23	22	19	24	17	26
"CDS;ID=637289548;locus_tag=SPO2069;product=benzoate-coenzyme A ligase, putative"	23	18	13	22	28	22
CDS;ID=637289549;locus_tag=SPO2070;product=DSBA-like thioredoxin family protein	33	27	24	19	34	26
"CDS;ID=637289550;locus_tag=SPO2071;product=drug resistance transporter, Bcr/CflA s	33	27	9	31	32	19
CDS;ID=637289551;locus_tag=SPO2072;product=TPR domain protein	24	19	14	15	17	10
CDS;ID=637289552;locus_tag=SPO2073;product=hypothetical protein	8	4	5	2	2	3
CDS;ID=637289553;locus_tag=SPO2074;product=transcription-repair coupling factor	67	76	63	76	78	51
CDS;ID=637289554;locus_tag=SPO2075;product=hypothetical protein	71	48	49	64	56	76
CDS;ID=637289555;locus_tag=SPO2076;product=delta-aminolevulinic acid dehydratase	26	39	19	39	39	18
"CDS;ID=637289556;locus_tag=SPO2077;product=twin-arginine translocation pathway sig	78	71	54	73	80	45
CDS;ID=637289557;locus_tag=SPO2078;product=penicillin amidase family protein	40	38	35	42	44	49
"CDS;ID=637289558;locus_tag=SPO2079;product=Na(+)/H(+) antiporter, homolog"	18	14	16	11	6	11
CDS;ID=637289559;locus_tag=SPO2080;product=GTP-binding protein HflX	96	71	60	63	87	97
CDS;ID=637289560;locus_tag=SPO2081;product=RNA-binding protein Hfq	194	235	214	204	196	260
CDS;ID=637289561;locus_tag=SPO2082;product=Trk system potassium uptake protein TrkH	20	12	9	16	11	12
CDS;ID=637289562;locus_tag=SPO2083;product=Trk system potassium uptake protein TrkA	308	222	263	257	252	262
CDS;ID=637289563;locus_tag=SPO2084;product=hypothetical protein	6	9	10	12	9	12
CDS;ID=637289564;locus_tag=SPO2085;product=nitrogen assimilation regulatory protein	55	32	48	50	46	43
CDS;ID=637289565;locus_tag=SPO2086;product=nitrogen regulation protein ntrY	76	63	54	92	66	71
CDS;ID=637289566;locus_tag=SPO2087;product=nitrogen regulation protein NtrC	22	19	13	22	14	17
CDS;ID=637289567;locus_tag=SPO2088;product=nitrogen regulation protein NtrB	9	9	5	8	7	4
"CDS;ID=637289568;locus_tag=SPO2089;product=tRNA-dihydrouridine synthase, putative"	11	4	9	7	7	11
"CDS;ID=637289569;locus_tag=SPO2090;product=2-C-methyl-D-erythritol 4-phosphate cyt	22	20	15	19	21	25
"CDS;ID=637289570;locus_tag=SPO2091;product=phosphatidylglycerophosphatase, putativ	6	3	4	4	7	4
CDS;ID=637289571;locus_tag=SPO2092;product=competence/damage-inducible protein CinA	2	3	1	3	2	3
CDS;ID=637289572;locus_tag=SPO2093;product=ammonium transporter	22	7	12	20	15	15
CDS;ID=637289573;locus_tag=SPO2094;product=actC domain protein	9	13	4	6	6	5
CDS;ID=637289574;locus_tag=SPO2095;product=MmgE/PrpD family protein	60	61	44	66	71	49
CDS;ID=637289575;locus_tag=SPO2096;product=aromatic-rich family protein	8	3	7	20	13	12
CDS;ID=637289576;locus_tag=SPO2097;product=hypoxanthine phosphoribosyltransferase	28	22	21	35	21	23
"CDS;ID=637289577;locus_tag=SPO2098;product=transcriptional regulator, LysR family"	18	5	12	13	11	8
CDS;ID=637289578;locus_tag=SPO2099;product=cytochrome c-554	0	4	0	4	2	0
CDS;ID=637289579;locus_tag=SPO2100;product=diheme cytochrome c-type	19	3	13	8	7	7
CDS;ID=637289580;locus_tag=SPO2101;product=amidohydrolase family protein	18	12	14	14	8	9
CDS;ID=637289581;locus_tag=SPO2102;product=lipoyl synthase	83	90	71	53	75	75
"CDS;ID=637289582;locus_tag=SPO2103;product=antioxidant, AhpC/Tsa family"	86	74	85	85	108	82

CDS;ID=637289583;locus_tag=SPO2104;product=hypothetical protein	8	3	6	8	5	9
"CDS;ID=637289584;locus_tag=SPO2105;product=transcriptional regulator, LysR family"	14	14	7	7	13	10
CDS;ID=637289585;locus_tag=SPO2106;product=hypothetical protein	10	10	7	9	13	10
CDS;ID=637289586;locus_tag=SPO2107;product=hypothetical protein	4	10	5	4	9	3
CDS;ID=637289587;locus_tag=SPO2108;product=trimethylamine methyltransferase family	150	112	125	121	133	158
CDS;ID=637289588;locus_tag=SPO2109;product=GMP synthase	81	60	43	54	107	63
CDS;ID=637289589;locus_tag=SPO2110;product=hypothetical protein	8	8	6	2	5	7
"CDS;ID=637289590;locus_tag=SPO2111;product=outer membrane transporter, OMPP1/FadL/	3	5	0	4	6	2
CDS;ID=637289591;locus_tag=SPO2112;product=transmembrane drug/metabolite transporte	1	3	3	2	2	3
CDS;ID=637289592;locus_tag=SPO2113;product=hypothetical protein	30	25	29	20	18	41
tRNA;ID=640698537;locus_tag=SPO_tRNA-Asn-1	484	281	132	121	18	150
tRNA;ID=640698538;locus_tag=SPO_tRNA-Leu-3	660	277	139	130	14	133
CDS;ID=637289595;locus_tag=SPO2116;product=DedA family protein	33	32	37	38	27	33
"CDS;ID=637289596;locus_tag=SPO2117;product=disulfide bond formation protein, DsbB	31	39	23	28	26	28
"CDS;ID=637289597;locus_tag=SPO2118;product=transcriptional regulator, AsnC family"	23	23	8	23	16	22
CDS;ID=637289598;locus_tag=SPO2119;product=arginase	36	28	22	36	21	12
CDS;ID=637289599;locus_tag=SPO2120;product=amidinotransferase family protein	7	16	8	0	11	16
CDS;ID=637289600;locus_tag=SPO2121;product=ornithine cyclodeaminase	15	17	13	8	20	22
CDS;ID=637289601;locus_tag=SPO2122;product=alkylphosphonate utilization operon prot	17	22	13	10	10	18
CDS;ID=637289602;locus_tag=SPO2123;product=HNH endonuclease family protein	74	46	34	29	44	58
CDS;ID=637289603;locus_tag=SPO2124;product=RNA pseudouridylate synthase	16	15	14	18	20	15
"CDS;ID=637289604;locus_tag=SPO2125;product=acetyltransferase, GNAT family"	13	10	10	7	7	10
CDS;ID=637289605;locus_tag=SPO2126;product=phospholipase/carboxylesterase family pr	23	18	12	16	18	20
"CDS;ID=637289606;locus_tag=SPO2127;product=DNA-3-methyladenine glycosylase II, put	16	11	13	5	13	15
"CDS;ID=637289607;locus_tag=SPO2128;product=transporter, putative"	16	13	12	7	8	9
CDS;ID=637289608;locus_tag=SPO2129;product=hypothetical protein	9	12	12	9	11	8
CDS;ID=637289609;locus_tag=SPO2130;product=2-isopropylmalate synthase	22	39	18	39	41	29
CDS;ID=637289610;locus_tag=SPO2131;product=cysteinyl-tRNA synthetase	87	86	75	120	107	89
CDS;ID=637289611;locus_tag=SPO2132;product=aspartate aminotransferase	37	25	27	17	35	40
"CDS;ID=637289612;locus_tag=SPO2133;product=outer membrane protein, putative"	1222	1154	1080	864	934	970
CDS;ID=637289613;locus_tag=SPO2134;product=trimethylamine methyltransferase family	25	24	22	23	22	25
CDS;ID=637289614;locus_tag=SPO2135;product=hypothetical protein	1158	1087	1077	1045	678	939
CDS;ID=637289615;locus_tag=SPO2136;product=ribonucleotide-diphosphate reductase alp	208	185	218	173	202	183
"CDS;ID=637289616;locus_tag=SPO2137;product=lipoprotein, putative"	11	15	12	7	7	11
tRNA;ID=640698539;locus_tag=SPO_tRNA-Pro-4	110	76	34	29	9	57
CDS;ID=637289618;locus_tag=SPO2139;product=hypothetical protein	26	28	22	31	34	37
CDS;ID=637289619;locus_tag=SPO2140;product=cold shock DNA-binding domain protein	89	70	68	87	71	86
"CDS;ID=637289620;locus_tag=SPO2141;product=pyridoxamine 5''-phosphate oxidase, put	197	163	179	151	128	226
CDS;ID=637289621;locus_tag=SPO2142;product=enoyl-(acyl carrier protein) reductase	138	96	112	108	131	123
CDS;ID=637289622;locus_tag=SPO2143;product=xanthine phosphoribosyltransferase	26	19	20	18	23	18
CDS;ID=637289623;locus_tag=SPO2144;product=hypothetical protein	15	14	10	11	8	13
CDS;ID=637289624;locus_tag=SPO2145;product=hypothetical protein	142	110	118	124	152	162
CDS;ID=637289625;locus_tag=SPO2146;product=anthranilate synthase component I	67	82	53	64	81	79

CDS;ID=637289626;locus_tag=SPO2147;product=hypothetical protein	14	16	7	9	13	11
CDS;ID=637289627;locus_tag=SPO2148;product=hypothetical protein	30	15	17	25	19	30
CDS;ID=637289628;locus_tag=SPO2149;product=anthranilate synthase component II	32	25	18	18	32	24
CDS;ID=637289629;locus_tag=SPO2150;product=anthranilate phosphoribosyltransferase	40	33	34	26	38	27
CDS;ID=637289630;locus_tag=SPO2151;product=indole-3-glycerol phosphate synthase	44	62	55	38	78	53
CDS;ID=637289631;locus_tag=SPO2152;product=molybdenum cofactor biosynthesis protein	21	27	20	34	21	19
CDS;ID=637289632;locus_tag=SPO2153;product=molybdenum cofactor biosynthesis protein	54	42	27	29	41	44
CDS;ID=637289633;locus_tag=SPO2154;product=LexA repressor	67	58	44	36	28	31
CDS;ID=637289634;locus_tag=SPO2155;product=competence protein	5	3	5	3	5	5
CDS;ID=637289635;locus_tag=SPO2156;product=glutamyl-tRNA synthetase	210	205	193	291	215	261
CDS;ID=637289636;locus_tag=SPO2157;product=citrate synthase I	161	130	180	191	261	192
CDS;ID=637289637;locus_tag=SPO2158;product=hypothetical protein	19	9	15	9	21	13
CDS;ID=637289638;locus_tag=SPO2159;product=radical SAM domain protein	4	6	1	6	3	7
"CDS;ID=637289639;locus_tag=SPO2160;product=corrinoid methyltransferase protein, pu	114	140	88	118	101	112
CDS;ID=637289640;locus_tag=SPO2161;product=hypothetical protein	68	46	71	76	53	85
CDS;ID=637289641;locus_tag=SPO2162;product=hypothetical protein	13	8	11	12	8	9
CDS;ID=637289642;locus_tag=SPO2163;product=hypothetical protein	6	6	0	6	4	0
CDS;ID=637289643;locus_tag=SPO2164;product=hypothetical protein	3	2	2	1	3	2
CDS;ID=637289644;locus_tag=SPO2165;product=Fe-S metabolism associated family protei	52	56	59	80	124	75
"CDS;ID=637289645;locus_tag=SPO2166;product=lipoprotein, putative"	37	35	30	53	48	36
CDS;ID=637289646;locus_tag=SPO2167;product=ribonuclease D	128	86	89	95	79	109
CDS;ID=637289647;locus_tag=SPO2168;product=phosphoribosylglycinamide formyltransfer	6	6	7	17	19	15
CDS;ID=637289648;locus_tag=SPO2169;product=phosphoribosylaminoimidazole synthetase	23	26	31	37	40	50
CDS;ID=637289649;locus_tag=SPO2170;product=metallo-beta-lactamase family protein	10	9	6	1	0	0
"CDS;ID=637289650;locus_tag=SPO2171;product=transcriptional regulator, LysR family'	5	10	8	4	7	5
tRNA;ID=640698540;locus_tag=SPO_tRNA-Met-2	35	21	13	8	6	7
CDS;ID=637289652;locus_tag=SPO2173;product=sensor histidine kinase	28	38	12	24	18	31
CDS;ID=637289653;locus_tag=SPO2174;product=metallo-beta-lactamase family protein	4	6	2	9	4	6
tRNA;ID=640698541;locus_tag=SPO_tRNA-Asn-2	475	274	121	102	10	146
CDS;ID=637289655;locus_tag=SPO2176;product=hypothetical protein	20	25	9	14	12	12
CDS;ID=637289656;locus_tag=SPO2177;product=acetoin utilization protein AcuC	6	3	5	1	4	5
CDS;ID=637289657;locus_tag=SPO2178;product=cardiolipin synthetase	9	14	11	1	10	21
CDS;ID=637289658;locus_tag=SPO2179;product=hypothetical protein	25	21	25	23	22	23
CDS;ID=637289659;locus_tag=SPO2180;product=hypothetical protein	36	19	14	21	21	42
"CDS;ID=637289660;locus_tag=SPO2181;product=ABC transporter, ATP-binding protein"	10	12	4	7	2	4
"CDS;ID=637289661;locus_tag=SPO2182;product=permease, putative"	3	4	0	5	5	0
CDS;ID=637289662;locus_tag=SPO2183;product=hypothetical protein	1	1	1	1	1	1
CDS;ID=637289663;locus_tag=SPO2184;product=hypothetical protein	31	24	21	26	26	15
CDS;ID=637289664;locus_tag=SPO2185;product=universal stress protein family protein	3	6	2	6	7	9
"CDS;ID=637289665;locus_tag=SPO2186;product=TRAP transporter, 4TM/12TM fusion prote	59	60	55	64	69	76
"CDS;ID=637289666;locus_tag=SPO2187;product=TRAP transporter solute receptor, TAXI	191	187	143	176	201	199
"CDS;ID=637289667;locus_tag=SPO2188;product=acetyltransferase, GNAT family"	2	2	1	0	3	1
"CDS;ID=637289668;locus_tag=SPO2189;product=transcriptional regulator, AsnC family'	10	7	10	10	10	8

CDS;ID=637289669;locus_tag=SPO2190;product=histidine utilization repressor	11	13	8	11	6	11
CDS;ID=637289670;locus_tag=SPO2191;product=imidazolonepropionase	2	3	3	2	3	4
CDS;ID=637289671;locus_tag=SPO2192;product=histidine ammonia-lyase	3	6	4	1	4	7
CDS;ID=637289672;locus_tag=SPOA0448;product=N-formylglutamate amidohydrolase	2	6	6	1	2	3
CDS;ID=637289673;locus_tag=SPO2193;product=urocanate hydratase	23	12	13	6	21	13
CDS;ID=637289674;locus_tag=SPO2194;product=atrazine chlorohydrolase	4	3	2	0	1	9
CDS;ID=637289675;locus_tag=SPO2195;product=amidohydrolase family protein	2	4	0	1	0	0
CDS;ID=637289676;locus_tag=SPO2196;product=diaminopropionate ammonia-lyase	2	9	5	3	5	2
CDS;ID=637289677;locus_tag=SPO2197;product=hypothetical protein	29	30	17	15	20	39
"CDS;ID=637289678;locus_tag=SPO2198;product=glyceraldehyde-3-phosphate dehydrogenas	36	18	14	50	29	33
CDS;ID=637289679;locus_tag=SPO2199;product=hypothetical protein	7	10	10	4	5	5
CDS;ID=637289680;locus_tag=SPO2200;product=pantetheine-phosphate adenylyltransferas	12	9	19	9	12	8
CDS;ID=637289681;locus_tag=SPO2201;product=CBS domain protein	228	175	149	142	144	269
"CDS;ID=637289682;locus_tag=SPO2202;product=transcriptional regulator, LysR family"	37	23	20	21	22	19
CDS;ID=637289683;locus_tag=SPO2203;product=methylmalonate-semialdehyde dehydrogenas	204	157	144	82	112	94
CDS;ID=637289684;locus_tag=SPO2204;product=hypothetical protein	3	1	1	1	3	1
CDS;ID=637289685;locus_tag=SPO2205;product=ErfK/YbiS/YcfS/YnhG family protein	30	28	33	20	41	27
CDS;ID=637289686;locus_tag=SPO2206;product=hypothetical protein	10	8	5	7	6	5
CDS;ID=637289687;locus_tag=SPO2207;product=hypothetical protein	24	33	17	12	19	24
CDS;ID=637289688;locus_tag=SPO2208;product=hypothetical protein	12	20	18	18	19	13
CDS;ID=637289689;locus_tag=SPO2209;product=glutamate--cysteine ligase family prote:	27	21	12	21	16	18
CDS;ID=637289690;locus_tag=SPO2210;product=BNR/Asp-box repeat domain protein	120	142	118	124	113	103
CDS;ID=637289691;locus_tag=SPO2211;product=acyl-CoA dehydrogenase	427	440	335	302	237	219
CDS;ID=637289692;locus_tag=SPO2212;product=enoyl-CoA hydratase/isomerase family pro	363	283	242	208	188	230
CDS;ID=637289693;locus_tag=SPO2213;product=3-hydroxyisobutyrate dehydrogenase	86	57	51	45	39	36
CDS;ID=637289694;locus_tag=SPO2214;product=choline sulfatase	30	21	19	19	23	35
CDS;ID=637289695;locus_tag=SPO2215;product=glyoxalase family protein	2	2	2	0	3	1
"CDS;ID=637289696;locus_tag=SPO2216;product=DNA-binding protein, putative"	10	5	2	7	1	9
CDS;ID=637289697;locus_tag=SPO2217;product=hypothetical protein	6	12	8	12	4	12
CDS;ID=637289698;locus_tag=SPO2218;product=excinuclease ABC subunit A	211	236	174	167	185	208
CDS;ID=637289699;locus_tag=SPO2219;product=rhodanese-like domain protein	1	2	5	0	0	3
CDS;ID=637289700;locus_tag=SPO2220;product=hypothetical protein	21	11	9	12	19	22
CDS;ID=637289701;locus_tag=SPO2221;product=von Willebrand factor type A domain prot	10	15	12	19	19	8
CDS;ID=637289702;locus_tag=SPO2222;product=dihydrolipoamide dehydrogenase	103	116	115	112	167	140
CDS;ID=637289703;locus_tag=SPO2223;product=hypothetical protein	25	20	15	26	20	31
CDS;ID=637289704;locus_tag=SPO2224;product=hypothetical protein	76	89	52	45	36	71
CDS;ID=637289705;locus_tag=SPO2225;product=hypothetical protein	79	83	66	82	68	88
CDS;ID=637289706;locus_tag=SPO2226;product=S-adenosylmethionine:tRNA ribosyltransfe	12	13	19	21	19	18
CDS;ID=637289707;locus_tag=SPO2227;product=hypothetical protein	74	55	36	37	57	36
CDS;ID=637289708;locus_tag=SPO2228;product=AhpC/TSA family protein	54	45	26	26	53	52
CDS;ID=637289709;locus_tag=SPO2229;product=hypothetical protein	7	5	7	5	6	5
"CDS;ID=637289710;locus_tag=SPO2230;product=peptidase, M23/M37 family"	104	77	70	55	84	80
CDS;ID=637289711;locus_tag=SPO2231;product=hypothetical protein	32	49	30	19	36	16

CDS;ID=637289712;locus_tag=SPO2232;product=hypothetical protein	10	9	7	9	15	15
"CDS;ID=637289713;locus_tag=SPO2233;product=peptidyl-prolyl cis-trans isomerase, cy	27	20	22	26	28	21
"CDS;ID=637289714;locus_tag=SPO2234;product=peptidyl-prolyl cis-trans isomerase, cy	305	288	299	281	407	386
CDS;ID=637289715;locus_tag=SPO2235;product=phosphoglycerate kinase	48	38	28	40	36	36
CDS;ID=637289716;locus_tag=SPO2236;product=hypothetical protein	1	0	2	0	0	3
CDS;ID=637289717;locus_tag=SPO2237;product=hypothetical protein	8	12	2	3	10	5
"CDS;ID=637289718;locus_tag=SPO2238;product=transcriptional regulator, LysR family"	4	3	0	1	5	4
CDS;ID=637289719;locus_tag=SPO2239;product=hypothetical protein	53	40	33	36	31	43
"CDS;ID=637289720;locus_tag=SPO2240;product=pyruvate dehydrogenase complex, E1 comp	234	245	199	242	307	239
CDS;ID=637289721;locus_tag=SPO2241;product=dihydrolipoamide acetyltransferase	119	113	107	111	149	136
"CDS;ID=637289722;locus_tag=SPO2242;product=pyruvate dehydrogenase complex, E2 comp	64	45	56	54	53	50
CDS;ID=637289723;locus_tag=SPO2243;product=hypothetical protein	58	46	26	32	65	43
CDS;ID=637289724;locus_tag=SPO2244;product=hypothetical protein	54	35	38	23	35	38
CDS;ID=637289725;locus_tag=SPO2245;product=hypothetical protein	31	12	14	17	12	10
CDS;ID=637289726;locus_tag=SPO2246;product=cysteine synthase A	177	181	173	143	117	290
CDS;ID=637289727;locus_tag=SPO2247;product=serine O-acetyltransferase	110	112	94	69	102	90
"CDS;ID=637289728;locus_tag=SPO2248;product=dnaK suppressor protein, putative"	5	0	0	5	1	2
CDS;ID=637289729;locus_tag=SPO2249;product=hypothetical protein	0	2	0	1	1	0
CDS;ID=637289730;locus_tag=SPO2250;product=hypothetical protein	17	16	31	23	15	16
CDS;ID=637289731;locus_tag=SPO2251;product=hypothetical protein	0	4	0	0	0	0
CDS;ID=637289732;locus_tag=SPO2252;product=hypothetical protein	2	2	0	0	0	1
CDS;ID=637289733;locus_tag=SPO2253;product=hypothetical protein	2	1	0	3	1	2
CDS;ID=640735063;locus_tag=SPO2254	0	0	0	1	2	1
CDS;ID=637289734;locus_tag=SPO2255;product=hypothetical protein	0	1	0	0	0	1
CDS;ID=637289735;locus_tag=SPO2256;product=hypothetical protein	2	0	2	0	0	1
"CDS;ID=637289736;locus_tag=SPO2257;product=major tail protein, TP901-1 family"	6	1	2	0	0	2
CDS;ID=637289737;locus_tag=SPO2258;product=hypothetical protein	3	3	0	0	1	0
"CDS;ID=637289738;locus_tag=SPO2259;product=head-tail adaptor, putative"	5	2	0	1	0	0
CDS;ID=637289739;locus_tag=SPO2260;product=hypothetical protein	2	2	0	0	1	2
"CDS;ID=637289740;locus_tag=SPO2261;product=major capsid protein, HK97 family"	2	1	2	0	2	5
"CDS;ID=637289741;locus_tag=SPO2262;product=phage prohead protease, HK97 family"	1	0	2	3	0	1
CDS;ID=637289742;locus_tag=SPO2263;product=hypothetical protein	0	0	0	0	0	0
"CDS;ID=637289743;locus_tag=SPO2264;product=portal protein, HK97 family"	3	2	3	1	0	1
"CDS;ID=637289744;locus_tag=SPO2266;product=terminase, large subunit, putative"	3	3	1	2	5	0
CDS;ID=637289745;locus_tag=SPO2267;product=hypothetical protein	23	21	21	29	20	28
CDS;ID=637289746;locus_tag=SPO2268;product=hypothetical protein	11	13	11	9	13	16
CDS;ID=637289747;locus_tag=SPO2269;product=hypothetical protein	15	14	10	9	8	15
CDS;ID=637289748;locus_tag=SPO2270;product=conserved hypothetical protein TIGR0024	137	106	90	114	122	109
CDS;ID=637289749;locus_tag=SPO2271;product=3-oxoacyl-(acyl carrier protein) syntha	176	185	150	208	221	201
"CDS;ID=637289750;locus_tag=SPO2272;product=phosphoribosylformimino-5-aminoimidazo	27	22	17	27	23	29
"CDS;ID=637289751;locus_tag=SPO2273;product=acetyltransferase, GNAT family"	108	103	73	45	63	53
CDS;ID=637289752;locus_tag=SPO2274;product=acyl carrier protein	94	68	59	80	90	50
CDS;ID=637289753;locus_tag=SPO2275;product=3-oxoacyl-(acyl-carrier-protein) reduct	174	171	122	153	155	200

CDS;ID=637289754;locus_tag=SPO2276;product=acyl-carrier-protein S-malonyltransferas	41	47	29	32	42	35
"CDS;ID=637289755;locus_tag=SPO2277;product=cytochrome, putative"	6	8	8	9	10	9
CDS;ID=637289756;locus_tag=SPO2278;product=hypothetical protein	179	193	139	155	129	172
CDS;ID=637289757;locus_tag=SPO2279;product=hypothetical protein	40	23	24	18	25	12
CDS;ID=637289758;locus_tag=SPO2280;product=hypothetical protein	84	83	50	117	82	108
CDS;ID=637289759;locus_tag=SPO2281;product=ribosomal protein S6	437	332	442	500	819	422
CDS;ID=637289760;locus_tag=SPO2282;product=30S ribosomal protein S18	120	107	104	121	265	93
CDS;ID=637289761;locus_tag=SPO2283;product=50S ribosomal protein L9	93	76	68	74	85	79
CDS;ID=637289762;locus_tag=SPO2284;product=transglycosylase SLT domain protein	30	32	17	12	45	23
"CDS;ID=637289763;locus_tag=SPO2285;product=DNA-binding protein, putative"	241	232	215	129	112	211
CDS;ID=637289764;locus_tag=SPO2286;product=autoinducer-binding transcriptional regu	34	26	17	16	18	24
CDS;ID=637289765;locus_tag=SPO2287;product=autoinducer synthesis protein	688	607	434	271	303	327
CDS;ID=637289766;locus_tag=SPO2288;product=trigger factor	477	486	487	527	527	626
CDS;ID=637289767;locus_tag=SPO2289;product=hypothetical protein	95	54	65	47	63	57
CDS;ID=637289768;locus_tag=SPO2290;product=hypothetical protein	181	124	161	195	221	228
tRNA;ID=640698542;locus_tag=SPO_tRNA-Leu-4	110	92	36	39	7	33
CDS;ID=637289770;locus_tag=SPO2292;product=hypothetical protein	20	12	14	14	14	12
CDS;ID=637289771;locus_tag=SPO2293;product=YjeF family protein	72	47	43	51	60	61
CDS;ID=637289772;locus_tag=SPO2294;product=nitrogen regulatory protein P-II	119	89	95	129	127	115
"CDS;ID=637289773;locus_tag=SPO2295;product=glutamine synthetase, type I"	316	353	267	222	293	266
CDS;ID=637289774;locus_tag=SPO2296;product=hypothetical protein	13	12	10	10	8	11
CDS;ID=637289775;locus_tag=SPO2297;product=hypothetical protein	327	279	287	305	229	298
CDS;ID=637289776;locus_tag=SPO2298;product=hypothetical protein	9	12	9	17	12	13
"CDS;ID=637289777;locus_tag=SPO2299;product=metallopeptidase, family M24"	5	6	2	5	1	3
CDS;ID=637289778;locus_tag=SPO2300;product=hypothetical protein	1	2	1	0	2	1
CDS;ID=637289779;locus_tag=SPO2301;product=osmC-like family protein	8	1	1	3	5	1
CDS;ID=637289780;locus_tag=SPO2302;product=bioY family protein	176	168	134	138	181	199
CDS;ID=637289781;locus_tag=SPO2303;product=hypothetical protein	6	11	6	7	17	10
CDS;ID=637289782;locus_tag=SPO2304;product=hypothetical protein	16	23	29	26	53	17
CDS;ID=637289783;locus_tag=SPO2305;product=adenylosuccinate lyase	114	113	104	149	181	160
CDS;ID=637289784;locus_tag=SPO2306;product=hypothetical protein	9	4	7	7	11	17
CDS;ID=637289785;locus_tag=SPO2307;product=hypothetical protein	14	4	3	2	10	9
CDS;ID=637289786;locus_tag=SPO2308;product=flagellar motor switch protein FliG	7	5	1	4	0	3
CDS;ID=637289787;locus_tag=SPO2309;product=hypothetical protein	9	8	7	10	5	6
"CDS;ID=637289788;locus_tag=SPO2310;product=lipid A biosynthesis lauroyl acyltrans1	27	27	27	32	23	17
CDS;ID=637289789;locus_tag=SPO2311;product=hypothetical protein	32	20	26	20	29	18
CDS;ID=637289790;locus_tag=SPO2312;product=aconitate hydratase	193	167	150	224	279	233
CDS;ID=637289791;locus_tag=SPO2313;product=hypothetical protein	4	3	4	4	1	4
"CDS;ID=637289792;locus_tag=SPO2314;product=periplasmic protein thiol:disulfide ox:	38	21	35	25	32	50
CDS;ID=637289793;locus_tag=SPO2315;product=heme exporter protein CcmC	72	57	39	58	91	101
CDS;ID=637289794;locus_tag=SPO2316;product=heme exporter protein CcmB	17	9	21	19	14	18
CDS;ID=637289795;locus_tag=SPO2317;product=heme exporter protein CcmA	32	22	26	25	31	36
CDS;ID=637289796;locus_tag=SPO2318;product=hypothetical protein	3	2	0	5	0	3

CDS;ID=637289797;locus_tag=SPO2319;product=hypothetical protein	8	16	5	10	7	12
CDS;ID=637289798;locus_tag=SPO2320;product=protein-export membrane protein SecF	67	47	38	59	63	51
CDS;ID=637289799;locus_tag=SPO2321;product=protein-export membrane protein SecD	123	118	103	96	145	125
"CDS;ID=637289800;locus_tag=SPO2322;product=preprotein translocase, YajC subunit"	61	70	59	66	74	83
CDS;ID=637289801;locus_tag=SPO2323;product=mechanosensitive ion channel family prot	10	10	16	12	11	11
CDS;ID=637289802;locus_tag=SPO2324;product=hypothetical protein	0	4	8	4	6	5
CDS;ID=637289803;locus_tag=SPO2325;product=seryl-tRNA synthetase	50	31	33	34	44	55
CDS;ID=637289804;locus_tag=SPO2326;product=hypothetical protein	81	86	83	66	44	58
CDS;ID=637289805;locus_tag=SPO2327;product=fatty acid desaturase	67	44	49	47	31	65
CDS;ID=637289806;locus_tag=SPO2328;product=GTP-binding protein EngA	121	85	103	139	91	121
CDS;ID=637289807;locus_tag=SPO2329;product=PQQ enzyme repeat family protein	73	52	37	45	70	47
CDS;ID=637289808;locus_tag=SPO2330;product=hypothetical protein	58	35	27	37	43	45
"CDS;ID=637289809;locus_tag=SPO2331;product=efflux transporter, RND family, MFP sub	136	93	108	126	130	132
"CDS;ID=637289810;locus_tag=SPO2332;product=transporter, AcrB/AcrD/AcrF family"	282	311	285	261	356	323
CDS;ID=637289811;locus_tag=SPO2333;product=hypothetical protein	18	17	22	24	14	15
CDS;ID=637289812;locus_tag=SPO2334;product=CAAX amino terminal protease family prot	8	9	7	8	7	7
"CDS;ID=637289813;locus_tag=SPO2335;product=ABC transporter, ATP-binding/permease p	68	65	53	51	28	76
CDS;ID=637289814;locus_tag=SPO2336;product=lysM domain protein	33	13	16	13	20	19
CDS;ID=637289815;locus_tag=SPO2337;product=decarboxylase family protein	84	58	70	70	98	61
CDS;ID=637289816;locus_tag=SPO2338;product=RarD	16	16	9	13	13	14
CDS;ID=637289817;locus_tag=SPO2339;product=enoyl-CoA hydratase/isomerase family pro	21	25	17	18	24	33
"CDS;ID=637289818;locus_tag=SPO2340;product=superoxide dismutase, Fe"	248	231	225	250	297	241
"CDS;ID=637289819;locus_tag=SPO2341;product=methyltransferase, FkbM family"	6	15	3	5	3	4
CDS;ID=637289820;locus_tag=SPO2342;product=hypothetical protein	63	60	68	39	67	56
CDS;ID=637289821;locus_tag=SPO2343;product=hypothetical protein	41	72	50	33	51	30
"CDS;ID=637289822;locus_tag=SPO2344;product=sarcosine oxidase, gamma subunit family	18	12	12	9	18	17
"CDS;ID=637289823;locus_tag=SPO2345;product=sarcosine oxidase, alpha subunit family	211	206	165	198	207	213
"CDS;ID=637289824;locus_tag=SPO2346;product=sarcosine oxidase, delta subunit family	35	35	26	23	49	34
CDS;ID=637289825;locus_tag=SPO2347;product=hypothetical protein	7	6	6	8	4	8
"CDS;ID=637289826;locus_tag=SPO2348;product=sarcosine oxidase, beta subunit family'	122	118	116	101	138	106
CDS;ID=637289827;locus_tag=SPO2349;product=cytochrome c-type biogenesis protein Cyc	52	16	21	39	29	58
CDS;ID=637289828;locus_tag=SPO2350;product=conserved hypothetical protein TIGR0025(	23	24	18	18	15	29
CDS;ID=637289829;locus_tag=SPO2351;product=hypothetical protein	33	20	25	26	16	25
CDS;ID=637289830;locus_tag=SPO2352;product=type I secretion target repeat protein	17	16	9	17	19	14
CDS;ID=637289831;locus_tag=SPO2353;product=hypothetical protein	12	6	3	3	3	7
CDS;ID=637289832;locus_tag=SPO2354;product=membrane protein	16	11	16	19	9	12
"CDS;ID=637289833;locus_tag=SPO2355;product=transcriptional regulator, IclR family'	36	26	30	37	37	25
"CDS;ID=637289834;locus_tag=SPO2356;product=TRAP dicarboxylate transporter, DctM su	5	3	4	6	5	5
"CDS;ID=637289835;locus_tag=SPO2357;product=TRAP dicarboxylate transporter, DctQ su	2	5	0	1	0	6
"CDS;ID=637289836;locus_tag=SPO2358;product=TRAP dicarboxylate transporter, DctP su	5	2	2	9	7	8
"CDS;ID=637289837;locus_tag=SPO2359;product=oxidoreductase, GMC family"	4	1	2	4	4	1
CDS;ID=640735064;locus_tag=SPO2360	2	3	2	3	2	11
CDS;ID=637289838;locus_tag=SPO2361;product=sensor histidine kinase/response regulat	25	8	9	21	17	12

"CDS;ID=637289839;locus_tag=SPO2362;product=amino acid ABC transporter, periplasmic	19	14	12	16	25	15
CDS;ID=637289840;locus_tag=SPO2363;product=phosphoserine phosphatase	2	1	2	2	3	11
"CDS;ID=637289841;locus_tag=SPO2364;product=amino acid ABC transporter, periplasmic	2	0	0	1	2	2
"CDS;ID=637289842;locus_tag=SPO2365;product=amino acid ABC transporter, permease pr	0	3	3	5	0	6
"CDS;ID=637289843;locus_tag=SPO2366;product=amino acid ABC transporter, permease pr	0	3	0	3	2	1
"CDS;ID=637289844;locus_tag=SPO2367;product=amino acid ABC transporter, ATP-binding	3	5	2	3	3	7
CDS;ID=637289845;locus_tag=SPO2368;product=Na/Pi-cotransporter family protein	3	6	4	4	6	5
"CDS;ID=637289846;locus_tag=SPO2369;product=DNA-binding response regulator, LuxR fa	16	25	21	20	34	34
CDS;ID=637289847;locus_tag=SPO2370;product=sodium:alanine symporter family protein	211	156	137	157	147	85
CDS;ID=637289848;locus_tag=SPO2371;product=universal stress protein family protein	87	58	95	69	89	59
"CDS;ID=637289849;locus_tag=SPO2372;product=transcriptional regulator, AsnC family"	9	11	12	6	8	8
CDS;ID=637289850;locus_tag=SPO2373;product=hypothetical protein	7	8	0	8	6	5
CDS;ID=637289851;locus_tag=SPO2374;product=tRNA-dihydrouridine synthase A	36	32	31	34	41	50
CDS;ID=640735065;locus_tag=SPO2375	3	4	5	1	7	6
CDS;ID=637289852;locus_tag=SPO2376;product=CoA-binding domain protein	1	5	2	2	2	2
CDS;ID=637289853;locus_tag=SPO2377;product=ferredoxin	6	4	2	2	1	0
"CDS;ID=637289854;locus_tag=SPO2378;product=selenium-binding protein, putative"	17	20	20	11	14	11
CDS;ID=640735066;locus_tag=SPO2379	17	13	8	6	14	18
CDS;ID=637289855;locus_tag=SPO2380;product=acyl-CoA dehydrogenase	15	14	3	3	9	16
"CDS;ID=637289856;locus_tag=SPO2381;product=transcriptional regulator, LysR family"	24	23	21	25	14	34
CDS;ID=637289857;locus_tag=SPO2382;product=tricarboxylate transporter family prote	6	4	8	6	9	7
CDS;ID=637289858;locus_tag=SPO2383;product=hypothetical protein	5	7	3	2	3	3
CDS;ID=637289859;locus_tag=SPO2384;product=tricarboxylate transporter family prote	9	6	11	6	10	3
"CDS;ID=637289860;locus_tag=SPO2385;product=benzaldehyde lyase, putative"	18	12	10	6	4	13
CDS;ID=637289861;locus_tag=SPO2386;product=hypothetical protein	4	7	1	1	0	3
"CDS;ID=637289862;locus_tag=SPO2387;product=oxidoreductase, FAD-binding"	9	4	2	3	2	5
"CDS;ID=637289863;locus_tag=SPO2388;product=translocator protein, LysE family"	10	14	10	9	13	9
"CDS;ID=637289864;locus_tag=SPO2389;product=oxidoreductase, molybdopterin-binding"	26	32	32	25	22	34
"CDS;ID=637289865;locus_tag=SPO2390;product=site-specific recombinase, phage integr	40	47	39	44	28	60
CDS;ID=640735067;locus_tag=SPO2391	6	0	5	6	4	8
CDS;ID=637289866;locus_tag=SPO2392;product=hypothetical protein	4	9	2	3	5	9
"CDS;ID=637289867;locus_tag=SPO2393;product=carbon monoxide dehydrogenase G protein	5	1	6	0	9	1
CDS;ID=637289868;locus_tag=SPO2394;product=carbon monoxide dehydrogenase F protein	0	4	1	3	1	2
CDS;ID=637289869;locus_tag=SPO2395;product=carbon monoxide dehydrogenase E protein	1	3	1	1	1	1
CDS;ID=637289870;locus_tag=SPO2396;product=carbon monoxide dehydrogenase D protein	5	4	3	3	7	7
"CDS;ID=637289871;locus_tag=SPO2397;product=carbon monoxide dehydrogenase, large su	10	3	3	10	8	6
"CDS;ID=637289872;locus_tag=SPO2398;product=carbon monoxide dehydrogenase, small su	3	0	0	0	2	2
"CDS;ID=637289873;locus_tag=SPO2399;product=carbon monoxide dehydrogenase, medium s	4	2	6	2	3	6
CDS;ID=637289874;locus_tag=SPO2400;product=carbon monoxide dehydrogenase operon C p	17	13	6	3	15	12
CDS;ID=637289875;locus_tag=SPO2401;product=type I secretion target repeat protein	136	133	109	117	128	145
CDS;ID=640735068;locus_tag=SPO2402	3	4	2	0	3	5
CDS;ID=637289876;locus_tag=SPO2403;product=hypothetical protein	5	7	4	1	3	6
CDS;ID=640735069;locus_tag=SPO2404	6	6	5	6	8	8

"CDS;ID=637289877;locus_tag=SPO2405;product=site-specific recombinase, phage integı	25	38	40	54	34	63
"CDS;ID=637289878;locus_tag=SPO2406;product=ISSpo6, transposase orf A"	4	7	9	5	5	16
"CDS;ID=637289879;locus_tag=SPO2407;product=ISSpo6, transposase orfB"	5	8	2	2	1	3
CDS;ID=637289880;locus_tag=SPO2408;product=hypothetical protein	8	3	5	3	4	8
"CDS;ID=637289881;locus_tag=SPO2409;product=oxidoreductase, short chain dehydrogena	5	1	1	1	1	1
CDS;ID=637289882;locus_tag=SPO2410;product=dihydroxy-acid dehydratase	18	14	3	14	11	17
CDS;ID=637289883;locus_tag=SPO2411;product=hypothetical protein	4	6	7	2	10	4
"CDS;ID=637289884;locus_tag=SPO2412;product=aldolase, putative"	0	0	1	3	3	2
"CDS;ID=637289885;locus_tag=SPO2413;product=oxidoreductase, short chain dehydrogena	6	7	12	1	4	3
CDS;ID=637289886;locus_tag=SPO2414;product=mandelate racemase/muconate lactonizing	2	3	3	2	3	1
CDS;ID=637289887;locus_tag=SPO2415;product=phytanoyl-CoA dioxygenase family proteiı	2	6	2	4	4	2
CDS;ID=637289888;locus_tag=SPO2416;product=3-hydroxyisobutyrate dehydrogenase fami	5	4	4	3	2	5
CDS;ID=637289889;locus_tag=SPO2417;product=gluconate 5-dehydrogenase	17	9	10	8	21	9
"CDS;ID=637289890;locus_tag=SPO2418;product=transporter, LysE family"	1	0	2	0	3	4
CDS;ID=637289891;locus_tag=SPO2419;product=hypothetical protein	1	1	5	2	2	1
CDS;ID=637289892;locus_tag=SPO2420;product=2-dehydro-3-deoxygluconokinase	5	1	1	4	2	3
CDS;ID=637289893;locus_tag=SPO2421;product=hypothetical protein	2	4	1	3	7	3
CDS;ID=637289894;locus_tag=SPO2422;product=D-isomer specific 2-hydroxyacid dehydroç	2	3	1	5	2	3
CDS;ID=637289895;locus_tag=SPO2423;product=hypothetical protein	0	1	1	1	0	0
CDS;ID=637289896;locus_tag=SPO2424;product=L-idonate 5-dehydrogenase	3	1	0	1	1	2
CDS;ID=637289897;locus_tag=SPO2425;product=shikimate 5-dehydrogenase	0	0	0	0	0	1
CDS;ID=637289898;locus_tag=SPO2426;product=hypothetical protein	3	0	1	2	3	1
"CDS;ID=637289899;locus_tag=SPO2427;product=oxidoreductase, short chain dehydrogena	2	0	0	0	0	2
CDS;ID=637289900;locus_tag=SPO2428;product=6-phosphogluconate dehydrogenase domain	5	4	4	0	3	1
"CDS;ID=637289901;locus_tag=SPO2429;product=oxidoreductase, Gfo/Idh/MocA family"	4	2	3	1	4	5
"CDS;ID=637289902;locus_tag=SPO2430;product=hydrolase, UxaA family"	4	1	3	5	5	4
"CDS;ID=637289903;locus_tag=SPO2431;product=TRAP dicarboxylate transporter, DctM sı	3	4	6	2	0	3
"CDS;ID=637289904;locus_tag=SPO2432;product=TRAP dicarboxylate transporter, DctQ sı	3	0	1	1	0	1
"CDS;ID=637289905;locus_tag=SPO2433;product=TRAP dicarboxylate transporter, DctP sı	7	12	6	9	12	10
"CDS;ID=637289906;locus_tag=SPO2434;product=transcriptional regulator, GntR family'	17	15	20	12	35	39
CDS;ID=637289907;locus_tag=SPO2435;product=fumarylacetoacetate hydrolase family prc	16	9	10	15	26	20
CDS;ID=637289908;locus_tag=SPO2436;product=FAD dependent oxidoreductase/aminomethy	3	4	1	3	1	10
CDS;ID=637289909;locus_tag=SPO2437;product=Asp/Glu/Hydantoin racemase family protei	2	3	1	2	3	3
CDS;ID=637289910;locus_tag=SPO2438;product=phytanoyl-CoA dioxygenase family proteiı	2	1	1	1	0	5
"CDS;ID=637289911;locus_tag=SPO2439;product=transcriptional regulator, GntR family'	2	6	3	6	7	9
"CDS;ID=637289912;locus_tag=SPO2440;product=metallopeptidase, family M24"	1	4	1	1	5	5
"CDS;ID=637289913;locus_tag=SPO2441;product=glycine betaine/proline ABC transporteı	2	2	9	5	7	10
"CDS;ID=637289914;locus_tag=SPO2442;product=glycine betaine/proline ABC transporteı	1	3	1	2	1	1
"CDS;ID=637289915;locus_tag=SPO2443;product=glycine betaine/proline ABC transporteı	3	3	3	3	4	6
CDS;ID=637289916;locus_tag=SPO2444;product=nucleoside diphosphate kinase	170	139	178	190	164	165
"CDS;ID=637289917;locus_tag=SPO2445;product=drug resistance transporter, Bcr/CflA s	1	5	2	5	4	4
"CDS;ID=637289918;locus_tag=SPO2446;product=ABC transporter, ATP-binding protein"	64	39	35	41	68	65
"CDS;ID=637289919;locus_tag=SPO2447;product=acetyltransferase, GNAT family"	11	18	12	5	13	15

"CDS;ID=637289920;locus_tag=SPO2448;product=membrane protein, MarC family"	12	6	6	4	2	11
CDS;ID=637289921;locus_tag=SPO2449;product=hypothetical protein	24	22	14	15	13	14
CDS;ID=637289922;locus_tag=SPO2450;product=Asp/Glu/Hydantoin racemase family prote:	7	12	4	2	9	11
"CDS;ID=637289923;locus_tag=SPO2451;product=acyltransferase, putative"	5	7	4	8	3	5
CDS;ID=637289924;locus_tag=SPO2452;product=DNA polymerase III subunit chi	9	16	8	10	6	16
CDS;ID=637289925;locus_tag=SPO2453;product=leucyl aminopeptidase	82	81	52	60	81	76
"CDS;ID=637289926;locus_tag=SPO2454;product=permease, YjgP/YjgQ family"	47	17	21	14	25	16
"CDS;ID=637289927;locus_tag=SPO2455;product=organic solvent tolerance protein, puta	88	82	53	34	67	49
CDS;ID=637289928;locus_tag=SPO2456;product=PPIC-type PPIASE domain	66	58	42	52	53	55
CDS;ID=637289929;locus_tag=SPO2457;product=4-hydroxythreonine-4-phosphate dehydroge	16	18	16	5	9	21
CDS;ID=637289930;locus_tag=SPO2458;product=dimethyladenosine transferase	19	12	14	8	6	6
CDS;ID=637289931;locus_tag=SPO2459;product=hypothetical protein	200	147	133	167	180	197
"CDS;ID=637289932;locus_tag=SPO2460;product=modification methylase, HemK family"	4	12	9	17	6	13
CDS;ID=637289933;locus_tag=SPO2461;product=peptide chain release factor 1	58	44	32	55	59	78
CDS;ID=637289934;locus_tag=SPO2462;product=hypothetical protein	15	20	10	29	26	8
CDS;ID=637289935;locus_tag=SPO2463;product=hypothetical protein	3	3	2	6	2	3
CDS;ID=637289936;locus_tag=SPO2464;product=agmatinase	73	51	39	55	68	50
CDS;ID=637289937;locus_tag=SPO2465;product=hypothetical protein	0	1	1	0	1	4
CDS;ID=637289938;locus_tag=SPO2466;product=glyoxalase family protein	13	22	10	13	22	14
CDS;ID=637289939;locus_tag=SPO2467;product=agmatinase	26	18	11	27	21	17
CDS;ID=637289940;locus_tag=SPO2468;product=amidohydrolase family protein	57	59	37	39	61	52
CDS;ID=637289941;locus_tag=SPO2469;product=mazG family protein	26	44	29	29	52	43
CDS;ID=637289942;locus_tag=SPO2470;product=inosine-uridine preferring nucleoside hy	11	12	8	17	17	7
"CDS;ID=637289943;locus_tag=SPO2471;product=acetyltransferase, GNAT family"	7	9	6	12	13	14
CDS;ID=637289944;locus_tag=SPO2472;product=hypothetical protein	13	8	6	10	14	4
"CDS;ID=637289945;locus_tag=SPO2473;product=haloacid dehalogenase, type II, putativ	8	13	20	18	13	8
CDS;ID=637289946;locus_tag=SPO2474;product=enolase	195	191	163	227	271	256
CDS;ID=637289947;locus_tag=SPO2475;product=hypothetical protein	20	10	14	19	15	22
"CDS;ID=637289948;locus_tag=SPO2476;product=lipoprotein, putative"	2	0	1	1	2	3
"CDS;ID=637289949;locus_tag=SPO2477;product=transcriptional regulator, Fur family"	36	25	33	33	23	40
CDS;ID=640735070;locus_tag=SPO2478	110	86	58	83	125	74
CDS;ID=637289950;locus_tag=SPO2479;product=hypothetical protein	8	9	9	5	9	8
CDS;ID=637289951;locus_tag=SPO2480;product=hypothetical protein	21	29	12	29	24	25
CDS;ID=637289952;locus_tag=SPO2481;product=drug/metabolite exporter family protein	4	4	6	2	5	4
CDS;ID=637289953;locus_tag=SPO2482;product=endonuclease/exonuclease/phosphatase far	7	5	3	11	8	7
CDS;ID=637289954;locus_tag=SPO2483;product=hypothetical protein	31	17	22	17	34	39
CDS;ID=637289955;locus_tag=SPO2484;product=tyrosyl-tRNA synthetase	44	26	38	40	44	59
CDS;ID=637289956;locus_tag=SPO2485;product=hypothetical protein	1	3	0	1	2	2
CDS;ID=637289957;locus_tag=SPO2486;product=hypothetical protein	0	1	0	1	0	1
"CDS;ID=637289958;locus_tag=SPO2487;product=3-hydroxyacyl-CoA dehydrogenase, type I	38	33	38	45	34	49
"CDS;ID=637289959;locus_tag=SPO2488;product=ABC transporter, ATP-binding protein"	86	108	97	163	179	128
CDS;ID=637289960;locus_tag=SPO2489;product=hypothetical protein	39	17	17	14	15	25
"CDS;ID=637289961;locus_tag=SPO2490;product=lipoprotein, SmpA/OmlA family"	40	43	31	30	20	41

CDS;ID=637289962;locus_tag=SPO2491;product=hypothetical protein	58	45	29	45	40	28
CDS;ID=637289963;locus_tag=SPO2492;product=ribosomal protein L32	61	84	90	71	83	82
CDS;ID=637289964;locus_tag=SPO2493;product=fatty acid/phospholipid synthesis prote:	128	114	138	121	163	125
CDS;ID=637289965;locus_tag=SPO2494;product=3-oxoacyl-(acyl-carrier-protein) synthas	68	70	51	54	64	66
"CDS;ID=637289966;locus_tag=SPO2495;product=integration host factor, alpha subunit'	149	117	102	86	86	97
"CDS;ID=637289967;locus_tag=SPO2496;product=transcriptional regulator, MerR family'	45	32	26	39	40	36
tRNA;ID=640698543;locus_tag=SPO_tRNA-Pro-5	115	69	81	77	42	116
CDS;ID=637289969;locus_tag=SPO2498;product=2'-deoxycytidine 5'-triphosphate deamina	74	55	55	48	53	62
CDS;ID=637289970;locus_tag=SPO2499;product=hypothetical protein	38	26	30	40	20	30
CDS;ID=637289971;locus_tag=SPO2500;product=segregation and condensation protein B	41	45	20	41	34	36
CDS;ID=637289972;locus_tag=SPO2501;product=segregation and condensation protein A	45	40	33	35	34	37
"CDS;ID=637289973;locus_tag=SPO2502;product=beta-N-acetylhexosaminidase, putative"	31	36	41	38	31	42
CDS;ID=637289974;locus_tag=SPO2503;product=hypothetical protein	243	229	196	211	256	275
CDS;ID=637289975;locus_tag=SPO2504;product=arginyl-tRNA synthetase	165	164	164	191	176	171
"CDS;ID=637289976;locus_tag=SPO2505;product=deoxyguanosinetriphosphate triphosphohy	29	44	36	41	36	42
CDS;ID=637289977;locus_tag=SPO2506;product=Iron-sulfur cluster assembly family prot	40	28	36	37	45	34
CDS;ID=637289978;locus_tag=SPO2507;product=hypothetical protein	17	14	17	10	21	21
CDS;ID=637289979;locus_tag=SPO2508;product=hypothetical protein	13	8	12	5	13	19
CDS;ID=637289980;locus_tag=SPO2509;product=exodeoxyribonuclease III	34	28	24	29	47	22
CDS;ID=637289981;locus_tag=SPO2510;product=salicylate hydroxylase	9	15	9	12	17	11
"CDS;ID=637289982;locus_tag=SPO2511;product=dnaK suppressor protein, putative"	159	126	98	122	73	119
"CDS;ID=637289983;locus_tag=SPO2512;product=ATPase, AAA family"	57	49	66	77	78	91
CDS;ID=637289984;locus_tag=SPO2513;product=hypothetical protein	274	145	190	219	194	213
CDS;ID=637289985;locus_tag=SPO2514;product=hypothetical protein	27	30	25	29	19	24
CDS;ID=637289986;locus_tag=SPO2515;product=hypothetical protein	79	69	58	57	69	76
CDS;ID=637289987;locus_tag=SPO2516;product=hypothetical protein	24	22	17	28	12	13
"CDS;ID=637289988;locus_tag=SPO2517;product=peptidase, M48 family"	9	13	9	5	14	18
CDS;ID=637289989;locus_tag=SPO2518;product=bacterial luciferase family protein	9	7	2	8	4	6
CDS;ID=637289990;locus_tag=SPO2519;product=hypothetical protein	3	6	7	5	6	8
CDS;ID=637289991;locus_tag=SPO2520;product=hypothetical protein	18	8	11	19	15	24
"CDS;ID=637289992;locus_tag=SPO2521;product=lipoprotein, putative"	27	27	24	40	21	27
CDS;ID=637289993;locus_tag=SPO2522;product=hypothetical protein	3	3	2	8	5	3
CDS;ID=637289994;locus_tag=SPO2523;product=glutamate-ammonia ligase adenylyltransfe	47	36	31	46	43	43
CDS;ID=637289995;locus_tag=SPO2524;product=YbaK/prolyl-tRNA synthetase domain prote	12	11	6	6	13	10
CDS;ID=637289996;locus_tag=SPO2525;product=hypothetical protein	73	65	45	61	30	32
CDS;ID=637289997;locus_tag=SPO2526;product=hypothetical protein	2	5	1	3	0	1
CDS;ID=637289998;locus_tag=SPO2527;product=6-aminohexanoate-cyclic-dimer hydrolase	10	9	8	17	12	6
CDS;ID=637289999;locus_tag=SPO2528;product=acyl-CoA synthase	18	11	19	16	13	18
CDS;ID=637290000;locus_tag=SPO2529;product=hypothetical protein	15	5	7	7	8	4
"CDS;ID=637290001;locus_tag=SPO2530;product=branched-chain amino acid ABC transport	5	1	3	5	7	11
"CDS;ID=637290002;locus_tag=SPO2531;product=branched-chain amino acid ABC transport	5	4	7	2	3	10
"CDS;ID=637290003;locus_tag=SPO2532;product=branched-chain amino acid ABC transport	10	1	4	4	5	4
"CDS;ID=637290004;locus_tag=SPO2533;product=branched-chain amino acid ABC transport	3	5	2	1	3	5

"CDS;ID=637290005;locus_tag=SPO2534;product=branched-chain amino acid ABC transport	8	11	7	5	6	7
CDS;ID=637290006;locus_tag=SPO2535;product=histone deacetylase/AcuC/AphA family pro	9	3	0	2	0	0
"CDS;ID=637290007;locus_tag=SPO2536;product=transcriptional regulator, LuxR family"	8	10	6	5	4	2
CDS;ID=637290008;locus_tag=SPO2537;product=hypothetical protein	0	1	0	0	0	1
CDS;ID=637290009;locus_tag=SPO2538;product=acyl-CoA dehydrogenase famiy protein	6	5	4	4	2	6
CDS;ID=637290010;locus_tag=SPO2539;product=AMP-binding enzyme	6	5	12	6	10	3
"CDS;ID=637290011;locus_tag=SPO2540;product=carbamoyl-phosphate synthase, putative"	8	8	3	6	3	6
"CDS;ID=637290012;locus_tag=SPO2541;product=propionyl-CoA carboxylase, putative"	7	4	1	5	2	6
CDS;ID=637290013;locus_tag=SPO2542;product=biotin/lipoate binding domain protein	1	0	1	1	1	0
"CDS;ID=637290014;locus_tag=SPO2543;product=transcriptional regulator, GntR family"	32	39	29	38	29	42
CDS;ID=637290015;locus_tag=SPO2544;product=amidohydrolase family protein	5	9	8	5	3	4
"CDS;ID=637290016;locus_tag=SPO2545;product=TRAP dicarboxylate transporter, DctP su	1	3	4	2	2	1
"CDS;ID=637290017;locus_tag=SPO2546;product=TRAP dicarboxylate transporter, DctQ su	2	3	1	3	1	3
"CDS;ID=637290018;locus_tag=SPO2547;product=TRAP dicarboxylate transporter, DctM su	3	6	1	4	3	8
"CDS;ID=637290019;locus_tag=SPO2548;product=oxidoreductase, zinc-binding dehydroger	6	10	5	6	1	6
CDS;ID=637290020;locus_tag=SPO2549;product=phosphotransferase family protein	2	1	7	7	4	9
"CDS;ID=637290021;locus_tag=SPO2550;product=transcriptional regulator, GntR family"	24	23	14	34	20	35
"CDS;ID=637290022;locus_tag=SPO2551;product=peptide/opine/nickel uptake family ABC	46	50	38	39	40	54
"CDS;ID=637290023;locus_tag=SPO2552;product=peptide/opine/nickel uptake family ABC	10	16	14	8	6	32
"CDS;ID=637290024;locus_tag=SPO2553;product=peptide/opine/nickel uptake family ABC	5	3	3	6	0	3
"CDS;ID=637290025;locus_tag=SPO2554;product=peptide/opine/nickel uptake family ABC	8	11	11	13	3	13
CDS;ID=637290026;locus_tag=SPO2555;product=hypothetical protein	12	11	2	9	11	11
CDS;ID=637290027;locus_tag=SPO2556;product=N-carbamoyl-L-amino acid amidohydrolase	2	4	4	2	3	3
CDS;ID=640735071;locus_tag=SPO2558	49	40	40	50	22	29
CDS;ID=637290028;locus_tag=SPO2559;product=gluconolactonase	16	11	9	5	11	11
CDS;ID=637290029;locus_tag=SPO2560;product=2-hydroxy-3-oxopropionate reductase	7	7	9	4	9	10
CDS;ID=637290030;locus_tag=SPO2561;product=ygbK domain protein	13	11	10	10	23	7
CDS;ID=637290031;locus_tag=SPO2562;product=hypothetical protein	10	2	5	3	8	4
"CDS;ID=637290032;locus_tag=SPO2563;product=hydroxypyruvate isomerase, putative"	13	8	12	11	11	10
CDS;ID=637290033;locus_tag=SPO2564;product=hypothetical protein	1	0	0	0	0	1
CDS;ID=637290034;locus_tag=SPO2565;product=ribosome-associated GTPase	31	22	22	26	33	43
CDS;ID=637290035;locus_tag=SPO2566;product=glyoxalase family protein	8	6	2	12	8	7
CDS;ID=637290036;locus_tag=SPO2567;product=hypothetical protein	192	171	174	292	248	283
CDS;ID=637290037;locus_tag=SPO2568;product=RDD family protein	10	7	6	11	13	16
CDS;ID=637290038;locus_tag=SPO2569;product=arginyl-tRNA-protein transferase	79	58	50	64	51	46
CDS;ID=637290039;locus_tag=SPO2570;product=glyoxalase family protein	11	6	3	1	4	14
"CDS;ID=637290040;locus_tag=SPO2571;product=TRAP transporter, DctM subunit, putativ	77	53	45	55	45	55
CDS;ID=637290041;locus_tag=SPO2572;product=hypothetical protein	29	13	14	10	18	17
"CDS;ID=637290042;locus_tag=SPO2573;product=bacterial extracellular solute-binding	39	43	27	21	25	26
CDS;ID=637290043;locus_tag=SPO2574;product=sensor histidine kinase	58	51	53	50	50	67
"CDS;ID=637290044;locus_tag=SPO2575;product=DNA-binding response regulator, LuxR fa	11	12	9	5	12	8
"CDS;ID=637290045;locus_tag=SPO2576;product=transcriptional regulator, TetR family"	5	1	1	2	4	6
CDS;ID=637290046;locus_tag=SPO2577;product=hypothetical protein	7	6	4	19	12	10

CDS;ID=637290047;locus_tag=SPO2578;product=acetolactate synthase III large subunit	69	79	85	76	93	73
CDS;ID=637290048;locus_tag=SPO2579;product=acetolactate synthase small subunit	15	19	20	10	16	16
CDS;ID=637290049;locus_tag=SPO2580;product=hypothetical protein	152	98	77	71	79	96
"CDS;ID=637290050;locus_tag=SPO2581;product=DNA-binding protein, putative"	27	29	36	31	25	31
CDS;ID=637290051;locus_tag=SPO2582;product=hypothetical protein	8	15	7	6	10	12
CDS;ID=637290052;locus_tag=SPO2583;product=amidase	46	29	31	14	29	40
CDS;ID=637290053;locus_tag=SPO2584;product=glyoxalase family protein	4	5	5	4	8	7
CDS;ID=637290054;locus_tag=SPO2585;product=peptide chain release factor 2	174	173	148	191	263	232
CDS;ID=637290055;locus_tag=SPO2586;product=type I secretion target repeat protein	13	9	13	9	11	14
CDS;ID=637290056;locus_tag=SPO2587;product=penicillin-binding protein 1A	338	242	223	253	294	317
"CDS;ID=637290057;locus_tag=SPO2588;product=N-acetylmuramoyl-L-alanine amidase, far	14	21	17	13	25	24
"CDS;ID=637290058;locus_tag=SPO2589;product=aminotransferase, classes I and II"	66	58	60	71	52	58
"CDS;ID=637290059;locus_tag=SPO2590;product=peptidase, M48 family"	78	85	68	72	72	54
"CDS;ID=637290060;locus_tag=SPO2591;product=27 kDa outer membrane protein, putative	59	56	29	39	26	38
"CDS;ID=637290061;locus_tag=SPO2592;product=beta-lactamase, putative"	17	9	5	21	15	25
CDS;ID=637290062;locus_tag=SPO2593;product=hypothetical protein	2	4	1	1	2	1
CDS;ID=637290063;locus_tag=SPO2594;product=4-hydroxy-3-methylbut-2-en-1-yl diphosph	50	49	41	40	57	51
CDS;ID=637290064;locus_tag=SPO2595;product=hypothetical protein	216	148	136	128	129	183
CDS;ID=637290065;locus_tag=SPO2596;product=5-aminolevulinate synthase	118	75	104	171	166	201
CDS;ID=637290066;locus_tag=SPO2597;product=hypothetical protein	38	69	48	40	59	45
CDS;ID=637290067;locus_tag=SPO2598;product=hypothetical protein	34	47	16	34	22	33
tRNA;ID=640698544;locus_tag=SPO_tRNA-Asp-1	466	232	102	139	21	151
tRNA;ID=640698545;locus_tag=SPO_tRNA-Asp-2	677	338	180	203	30	225
CDS;ID=637290070;locus_tag=SPO2601;product=pirin domain protein	14	5	3	6	8	7
"CDS;ID=637290071;locus_tag=SPO2602;product=transcriptional regulator, RpiR family"	30	28	31	23	22	13
CDS;ID=637290072;locus_tag=SPO2603;product=N-formylglutamate amidohydrolase family	18	30	12	8	25	22
CDS;ID=637290073;locus_tag=SPO2604;product=hypothetical protein	8	4	3	5	14	6
"CDS;ID=637290074;locus_tag=SPO2605;product=TRAP dicarboxylate transporter, DctM su	62	36	48	33	41	13
"CDS;ID=637290075;locus_tag=SPO2606;product=bacterial extracellular solute-binding	110	88	79	50	65	78
"CDS;ID=637290076;locus_tag=SPO2607;product=gamma-glutamylisopropylamide synthetase	138	147	130	92	142	87
"CDS;ID=637290077;locus_tag=SPO2608;product=aldehyde dehydrogenase, putative"	57	65	61	66	79	43
"CDS;ID=637290078;locus_tag=SPO2609;product=alcohol dehydrogenase, iron-containing"	77	65	49	69	91	37
CDS;ID=637290079;locus_tag=SPO2610;product=hypothetical protein	103	98	62	92	84	85
tRNA;ID=640698546;locus_tag=SPO_tRNA-Val-3	1315	670	398	424	37	265
"CDS;ID=637290081;locus_tag=SPO2612;product=DNA-binding protein HU, putative"	30	22	30	33	24	36
CDS;ID=637290082;locus_tag=SPO2613;product=ATP-dependent protease La	609	470	474	449	857	558
CDS;ID=637290083;locus_tag=SPO2614;product=hypothetical protein	51	30	59	51	70	67
"CDS;ID=637290084;locus_tag=SPO2615;product=NADH-dependent flavin oxidoreductase, (	99	68	85	79	67	74
CDS;ID=637290085;locus_tag=SPO2616;product=queuine tRNA-ribosyltransferase	15	14	14	28	20	18
CDS;ID=637290086;locus_tag=SPO2617;product=SPFH domain/band 7 family protein	38	33	42	58	38	63
CDS;ID=637290087;locus_tag=SPO2618;product=hypothetical protein	8	4	11	12	8	16
CDS;ID=637290088;locus_tag=SPO2619;product=hypothetical protein	83	66	76	125	87	102
CDS;ID=637290089;locus_tag=SPO2620;product=iron-sulfur cluster assembly accessory p	46	24	36	26	28	34

CDS;ID=637290090;locus_tag=SPO2621;product=triosephosphate isomerase	27	8	18	21	19	17
"CDS;ID=637290091;locus_tag=SPO2622;product=acetyltransferase, GNAT family"	12	17	13	10	11	7
"CDS;ID=637290092;locus_tag=SPO2623;product=transporter, LysE family"	4	2	1	1	0	3
"CDS;ID=637290093;locus_tag=SPO2624;product=transcriptional regulator, AsnC family'	4	2	3	0	3	1
CDS;ID=637290094;locus_tag=SPO2625;product=hypothetical protein	7	20	16	10	8	10
"CDS;ID=637290095;locus_tag=SPO2626;product=TRAP transporter, DctM subunit"	106	89	99	77	74	68
"CDS;ID=637290096;locus_tag=SPO2627;product=TRAP transporter, DctQ subunit"	36	41	38	30	26	22
"CDS;ID=637290097;locus_tag=SPO2628;product=TRAP transporter solute receptor, DctP	121	150	109	74	87	58
CDS;ID=637290098;locus_tag=SPO2629;product=C4-dicarboxylate transport transcriptior	10	13	5	8	9	10
CDS;ID=637290099;locus_tag=SPO2630;product=C4-dicarboxylate transport sensor protei	21	23	12	27	22	24
"CDS;ID=637290100;locus_tag=SPO2631;product=transcriptional regulator, AsnC family'	5	5	2	2	4	4
CDS;ID=637290101;locus_tag=SPO2632;product=uroporphyrin-III C-methyltransferase	29	43	30	21	21	22
CDS;ID=637290102;locus_tag=SPO2633;product=hypothetical protein	13	17	7	5	9	3
"CDS;ID=637290103;locus_tag=SPO2634;product=sulfite reductase, putative"	51	57	43	47	47	57
CDS;ID=637290104;locus_tag=SPO2635;product=phosphoadenosine phosphosulfate reductas	14	13	11	22	26	20
CDS;ID=637290105;locus_tag=SPO2636;product=hypothetical protein	14	7	12	19	22	8
CDS;ID=637290106;locus_tag=SPO2637;product=ferredoxin--NADP reductase	93	74	96	139	177	135
CDS;ID=637290107;locus_tag=SPO2638;product=translation initiation factor IF-3	533	414	407	448	589	405
CDS;ID=637290108;locus_tag=SPO2639;product=molybdopterin biosynthesis protein	8	3	6	8	10	4
CDS;ID=637290109;locus_tag=SPO2640;product=XdhC/CoxI family protein	34	31	46	34	31	50
CDS;ID=637290110;locus_tag=SPO2641;product=hypothetical protein	1	0	2	1	0	0
CDS;ID=637290111;locus_tag=SPO2642;product=YeeE/YedE family protein	3	1	0	2	2	1
CDS;ID=637290112;locus_tag=SPO2643;product=hypothetical protein	5	1	0	3	0	2
CDS;ID=637290113;locus_tag=SPO2644;product=VWA domain containing CoxE-like family p	3	0	1	2	4	6
"CDS;ID=637290114;locus_tag=SPO2645;product=protease, S2 family"	14	13	6	15	15	11
CDS;ID=637290115;locus_tag=SPO2646;product=hypothetical protein	33	29	15	19	23	32
CDS;ID=637290116;locus_tag=SPO2647;product=hypothetical protein	42	54	45	43	43	51
CDS;ID=637290117;locus_tag=SPO2648;product=hypothetical protein	18	11	21	19	27	25
CDS;ID=637290118;locus_tag=SPO2649;product=hypothetical protein	58	51	51	30	40	75
"CDS;ID=637290119;locus_tag=SPO2650;product=methyltransferase, FkbM family"	18	17	16	14	16	7
"CDS;ID=637290120;locus_tag=SPO2651;product=glycosyl transferase, group 2 family pı	26	20	25	8	25	15
CDS;ID=637290121;locus_tag=SPO2652;product=hypothetical protein	21	13	18	14	11	12
CDS;ID=637290122;locus_tag=SPO2653;product=dihydroorotase	42	29	35	45	89	50
CDS;ID=637290123;locus_tag=SPO2654;product=orotate phosphoribosyltransferase	17	15	15	27	58	35
CDS;ID=637290124;locus_tag=SPO2655;product=replicative DNA helicase	134	112	101	147	155	151
"CDS;ID=637290125;locus_tag=SPO2656;product=transcriptional regulator, LysR family'	4	11	6	4	8	7
CDS;ID=637290126;locus_tag=SPO2657;product=D-cysteine desulfhydrase	0	2	0	1	3	1
"CDS;ID=637290127;locus_tag=SPO2658;product=glutamate/aspartate ABC transporter, pe	7	1	5	1	0	3
"CDS;ID=637290128;locus_tag=SPO2659;product=glutamate/aspartate ABC transporter, pe	4	2	1	1	8	1
"CDS;ID=637290129;locus_tag=SPO2660;product=glutamate/aspartate ABC transporter, pe	3	9	7	12	8	7
"CDS;ID=637290130;locus_tag=SPO2661;product=glutamate/aspartate ABC transporter, AT	2	6	0	2	1	2
"CDS;ID=637290131;locus_tag=SPO2662;product=aspartate racemase, putative"	5	9	2	5	8	11
CDS;ID=637290132;locus_tag=SPO2663;product=aspartate ammonia-lyase	6	1	3	2	0	6

"CDS;ID=637290133;locus_tag=SPO2664;product=polar amino acid uptake family ABC trar	1	7	4	5	3	3
"CDS;ID=637290134;locus_tag=SPO2665;product=polar amino acid uptake family ABC trar	1	3	1	2	1	1
"CDS;ID=637290135;locus_tag=SPO2666;product=polar amino acid uptake family ABC trar	3	2	1	0	1	4
"CDS;ID=637290136;locus_tag=SPO2667;product=polar amino acid uptake family ABC trar	1	0	0	1	2	0
"CDS;ID=637290137;locus_tag=SPO2668;product=transcriptional regulator, LysR family'	7	7	7	2	2	6
"CDS;ID=637290138;locus_tag=SPO2669;product=oxidoreductase, 2OG-Fe(II) oxygenase fa	12	7	13	6	8	6
CDS;ID=637290139;locus_tag=SPO2670;product=alanine racemase	37	37	31	62	48	60
CDS;ID=637290140;locus_tag=SPO2671;product=membrane protein	83	61	56	59	56	69
"CDS;ID=637290141;locus_tag=SPO2672;product=ABC transporter, ATP-binding protein"	64	56	42	51	44	62
"CDS;ID=637290142;locus_tag=SPO2673;product=paraquat-inducible protein A, putative'	15	21	11	12	33	10
CDS;ID=637290143;locus_tag=SPO2674;product=DNA repair protein RadA	74	67	68	45	59	102
CDS;ID=637290144;locus_tag=SPO2675;product=CvpA family protein	52	51	35	56	28	57
CDS;ID=637290145;locus_tag=SPO2676;product=hypothetical protein	2	2	1	1	0	0
CDS;ID=637290146;locus_tag=SPO2677;product=amidophosphoribosyltransferase	183	154	172	218	141	214
CDS;ID=637290147;locus_tag=SPO2678;product=hypothetical protein	47	38	49	69	48	51
"CDS;ID=637290148;locus_tag=SPO2679;product=oxidoreductase, short-chain dehydrogena	44	34	39	42	44	57
CDS;ID=637290149;locus_tag=SPO2680;product=hypothetical protein	17	14	7	7	5	16
CDS;ID=637290150;locus_tag=SPO2681;product=twin-arginine translocation protein Tat(	142	91	66	94	100	126
CDS;ID=637290151;locus_tag=SPO2682;product=twin-arginine translocation protein TatE	79	60	51	63	88	55
"CDS;ID=637290152;locus_tag=SPO2683;product=twin-arginine translocation protein, Ta	118	93	96	109	122	110
CDS;ID=637290153;locus_tag=SPO2684;product=hypothetical protein	0	0	0	2	0	0
CDS;ID=637290154;locus_tag=SPO2685;product=hypothetical protein	7	3	0	4	4	8
CDS;ID=637290155;locus_tag=SPO2686;product=LysM domain/M23/M37 peptidase	113	78	53	69	77	71
CDS;ID=637290156;locus_tag=SPO2687;product=protein-L-isoaspartate O-methyltransfera	68	60	70	53	69	69
CDS;ID=637290157;locus_tag=SPO2688;product=acid phosphatase SurE	78	55	61	92	53	92
"CDS;ID=637290158;locus_tag=SPO2689;product=iron ABC transporter, ATP-binding prote	72	54	44	71	69	105
CDS;ID=637290159;locus_tag=SPO2690;product=hypothetical protein	34	35	23	40	21	31
CDS;ID=637290160;locus_tag=SPO2691;product=hypothetical protein	34	49	15	23	31	31
"CDS;ID=637290161;locus_tag=SPO2692;product=oxidoreductase, short chain dehydrogena	15	5	7	6	10	12
CDS;ID=637290162;locus_tag=SPO2693;product=hypothetical protein	18	11	12	13	11	20
CDS;ID=637290163;locus_tag=SPO2694;product=cytochrome c'	7	2	9	1	5	5
CDS;ID=637290164;locus_tag=SPO2695;product=peptide chain release factor 3	68	69	64	72	88	96
"CDS;ID=637290165;locus_tag=SPO2696;product=beta-lactamase, putative"	3	6	0	1	5	8
"CDS;ID=637290166;locus_tag=SPO2697;product=acyl-CoA synthetase, putative"	1	3	0	5	3	3
CDS;ID=637290167;locus_tag=SPO2698;product=acyl-CoA dehydrogenase family protein	1	4	1	1	1	3
"CDS;ID=637290168;locus_tag=SPO2699;product=opine/polyamine ABC transporter, permea	0	1	3	2	2	0
"CDS;ID=637290169;locus_tag=SPO2700;product=opine/polyamine ABC transporter, permea	0	0	1	1	0	0
"CDS;ID=637290170;locus_tag=SPO2701;product=opine/polyamine ABC transporter, perip]	1	0	0	1	7	4
"CDS;ID=637290171;locus_tag=SPO2702;product=opine/polyamine ABC transporter, ATP-b:	4	2	1	2	1	3
CDS;ID=637290172;locus_tag=SPO2703;product=hypothetical protein	1	2	0	2	0	4
"CDS;ID=637290173;locus_tag=SPO2704;product=transcriptional regulator, AraC family'	12	9	15	9	8	24
CDS;ID=637290174;locus_tag=SPO2705;product=3-hydroxyacyl-CoA dehydrogenase family p	1	1	0	0	0	1
CDS;ID=637290175;locus_tag=SPO2706;product=carnitinyl-CoA dehydratase	0	0	3	1	2	6

CDS;ID=637290176;locus_tag=SPO2707;product=bacterial luciferase family protein	2	5	4	2	1	7
CDS;ID=637290177;locus_tag=SPO2708;product=aldehyde dehydrogenase family protein	2	3	2	3	2	3
"CDS;ID=637290178;locus_tag=SPO2709;product=endoribonuclease L-PSP, putative"	1	2	2	0	0	1
CDS;ID=637290179;locus_tag=SPO2710;product=3-oxoadipate enol-lactonase family prote	3	0	0	0	0	3
CDS;ID=637290180;locus_tag=SPO2711;product=CaiB/BaiF family protein	10	4	2	1	0	5
CDS;ID=637290181;locus_tag=SPO2712;product=hypothetical protein	12	0	6	3	1	5
CDS;ID=637290182;locus_tag=SPO2713;product=translocase	6	9	13	3	5	5
"CDS;ID=637290183;locus_tag=SPO2714;product=peptidase, M50 family"	25	34	17	21	22	18
CDS;ID=637290184;locus_tag=SPO2715;product=GAF domain protein	27	38	45	24	30	36
CDS;ID=637290185;locus_tag=SPO2716;product=type I secretion target repeat protein	161	161	126	122	151	150
"CDS;ID=637290186;locus_tag=SPO2717;product=SapC protein, putative"	16	19	13	12	9	17
CDS;ID=637290187;locus_tag=SPO2718;product=hypothetical protein	1	3	6	4	4	3
CDS;ID=637290188;locus_tag=SPO2719;product=hypothetical protein	13	6	7	1	5	13
CDS;ID=637290189;locus_tag=SPO2720;product=hypothetical protein	0	0	0	0	1	2
CDS;ID=637290190;locus_tag=SPO2721;product=hypothetical protein	18	16	8	8	12	9
CDS;ID=637290191;locus_tag=SPO2722;product=hypothetical protein	3	1	1	1	2	1
CDS;ID=637290192;locus_tag=SPO2723;product=hypothetical protein	169	184	158	139	207	194
CDS;ID=637290193;locus_tag=SPO2724;product=hypothetical protein	127	81	81	90	127	104
CDS;ID=637290194;locus_tag=SPO2725;product=hypothetical protein	0	0	0	0	0	0
CDS;ID=637290195;locus_tag=SPO2726;product=hypothetical protein	2	1	7	2	2	1
CDS;ID=637290196;locus_tag=SPO2729;product=hypothetical protein	0	0	0	0	0	0
CDS;ID=637290197;locus_tag=SPO2730;product=hypothetical protein	0	0	0	0	0	0
"CDS;ID=637290198;locus_tag=SPO2731;product=ISSpo7, transposase"	1	1	3	4	3	4
CDS;ID=640735072;locus_tag=SPO2732	0	4	3	0	0	1
"CDS;ID=637290199;locus_tag=SPO2733;product=type I restriction-modification system,	153	146	123	168	188	187
"CDS;ID=637290200;locus_tag=SPO2734;product=type I restriction-modification system,	79	81	74	84	74	87
"CDS;ID=637290201;locus_tag=SPO2735;product=type I restriction-modification system,	120	112	98	74	81	113
CDS;ID=637290202;locus_tag=SPO3899;product=hypothetical protein	51	41	30	32	28	48
CDS;ID=637290203;locus_tag=SPO2736;product=hypothetical protein	24	15	19	15	15	14
tRNA;ID=640698547;locus_tag=SPO_tRNA-Ser-4	118	62	34	51	13	35
"CDS;ID=637290205;locus_tag=SPO2738;product=lipoprotein, putative"	34	25	24	29	57	33
CDS;ID=637290206;locus_tag=SPO2739;product=D-alanyl-D-alanine carboxypeptidase	52	47	54	52	57	67
CDS;ID=637290207;locus_tag=SPO2740;product=thymidylate kinase	11	26	6	20	21	19
CDS;ID=637290208;locus_tag=SPO2741;product=DNA polymerase III subunit delta	13	12	14	10	12	24
"CDS;ID=637290209;locus_tag=SPO2742;product=hydrolase, TatD family"	26	10	15	12	17	17
CDS;ID=637290210;locus_tag=SPO2743;product=hypothetical protein	14	20	14	13	11	23
"CDS;ID=637290211;locus_tag=SPO2744;product=malonate transporter, putative"	22	9	2	17	14	10
CDS;ID=637290212;locus_tag=SPO2745;product=hypothetical protein	13	6	8	11	13	5
CDS;ID=637290213;locus_tag=SPO2746;product=hypothetical protein	131	134	109	94	150	153
"CDS;ID=637290214;locus_tag=SPO2747;product=diguanylate cyclase, putative"	4	1	5	2	4	5
CDS;ID=637290215;locus_tag=SPO2748;product=hypothetical protein	10	5	17	13	11	5
CDS;ID=637290216;locus_tag=SPO2749;product=hypothetical protein	26	15	14	25	19	32
CDS;ID=637290217;locus_tag=SPO2750;product=trimethylamine methyltransferase family	8	7	6	12	14	15

"CDS;ID=637290218;locus_tag=SPO2751;product=HAD-superfamily hydrolase, subfamily IA	14	13	13	6	7	8
CDS;ID=637290219;locus_tag=SPO2752;product=hypothetical protein	18	21	23	14	20	26
"CDS;ID=637290220;locus_tag=SPO2753;product=diguanylate cyclase, putative/response	5	1	1	6	1	5
CDS;ID=637290221;locus_tag=SPO2754;product=hypothetical protein	13	4	4	7	4	5
CDS;ID=637290222;locus_tag=SPO2755;product=hypothetical protein	11	6	3	10	10	9
CDS;ID=637290223;locus_tag=SPO2756;product=RNA polymerase sigma-70 factor	7	4	7	4	8	4
CDS;ID=637290224;locus_tag=SPO2757;product=EF hand domain protein	13	17	7	12	20	22
CDS;ID=637290225;locus_tag=SPO2758;product=hypothetical protein	103	63	50	62	60	96
"CDS;ID=637290226;locus_tag=SPO2759;product=hydrolase, NUDIX family"	37	31	27	33	39	31
CDS;ID=637290227;locus_tag=SPO2760;product=metallo-beta-lactamase family protein	77	65	51	70	81	66
"CDS;ID=637290228;locus_tag=SPO2761;product=transcriptional activator, Baf family"	36	29	34	30	30	39
CDS;ID=637290229;locus_tag=SPO2762;product=biotin--acetyl-CoA-carboxylase ligase	25	17	13	14	19	25
CDS;ID=637290230;locus_tag=SPO2763;product=NADH dehydrogenase subunit N	137	103	77	95	112	106
CDS;ID=637290231;locus_tag=SPO2764;product=NADH dehydrogenase subunit M	62	69	65	63	76	81
CDS;ID=637290232;locus_tag=SPO2765;product=NADH dehydrogenase subunit L	218	186	191	197	209	204
CDS;ID=637290233;locus_tag=SPO2766;product=NADH dehydrogenase kappa subunit	47	33	35	53	63	36
CDS;ID=637290234;locus_tag=SPO2767;product=NADH dehydrogenase subunit J	95	69	50	67	82	68
"CDS;ID=637290235;locus_tag=SPO2768;product=4-carboxymuconolactone decarboxylase, p	106	94	99	76	113	107
CDS;ID=637290236;locus_tag=SPO2769;product=hypothetical protein	122	109	112	109	175	118
CDS;ID=637290237;locus_tag=SPO2770;product=NADH dehydrogenase subunit I	60	66	59	57	88	50
CDS;ID=637290238;locus_tag=SPO2771;product=hypothetical protein	122	114	92	99	104	130
CDS;ID=637290239;locus_tag=SPO2772;product=NADH dehydrogenase subunit H	55	73	70	69	76	62
"CDS;ID=637290240;locus_tag=SPO2773;product=lipoprotein, putative"	48	36	28	32	17	43
CDS;ID=637290241;locus_tag=SPO2774;product=NADH dehydrogenase gamma subunit	145	121	103	103	113	139
CDS;ID=637290242;locus_tag=SPO2775;product=hypothetical protein	77	67	49	40	65	61
CDS;ID=637290243;locus_tag=SPO2776;product=hypothetical protein	46	31	25	41	25	42
"CDS;ID=637290244;locus_tag=SPO2777;product=NADH-quinone oxidoreductase, F subunit'	239	190	201	241	243	205
CDS;ID=637290245;locus_tag=SPO2778;product=hypothetical protein	100	92	78	102	106	95
CDS;ID=637290246;locus_tag=SPO2779;product=hypothetical protein	120	61	93	83	82	81
CDS;ID=637290247;locus_tag=SPO2780;product=ATP synthase subunit E	298	254	219	197	246	225
"CDS;ID=637290248;locus_tag=SPO2781;product=lipoprotein, putative"	172	140	123	127	123	167
CDS;ID=637290249;locus_tag=SPO2782;product=NADH dehydrogenase delta subunit	193	221	192	164	218	185
CDS;ID=637290250;locus_tag=SPO2783;product=hypothetical protein	13	7	15	35	18	7
CDS;ID=637290251;locus_tag=SPO2784;product=NADH dehydrogenase subunit C	52	72	45	60	86	65
CDS;ID=637290252;locus_tag=SPO2785;product=NADH dehydrogenase beta subunit	65	55	55	61	82	34
CDS;ID=637290253;locus_tag=SPO2786;product=NADH dehydrogenase alpha subunit	72	75	52	63	110	103
CDS;ID=637290254;locus_tag=SPO2787;product=enoyl-CoA hydratase	18	13	9	12	7	17
CDS;ID=637290255;locus_tag=SPO2788;product=hydroxymethylglutaryl-CoA lyase	29	21	19	22	30	29
"CDS;ID=637290256;locus_tag=SPO2789;product=methylcrotonyl-CoA carboxylase, alpha s	47	36	40	45	53	55
CDS;ID=637290257;locus_tag=SPO3900;product=thermonuclease family protein	8	10	16	15	13	4
"CDS;ID=637290258;locus_tag=SPO2790;product=methylcrotonyl-CoA carboxylase, beta su	34	40	31	26	42	25
CDS;ID=637290259;locus_tag=SPO2791;product=acetyl-coenzyme A synthetase	9	8	13	9	16	17
CDS;ID=637290260;locus_tag=SPO2792;product=outer membrane protein OmpW	77	74	81	143	183	116

CDS;ID=637290261;locus_tag=SPO2793;product=isovaleryl-CoA dehydrogenase	272	250	240	262	291	302
CDS;ID=637290262;locus_tag=SPO2794;product=hypothetical protein	0	0	1	0	2	1
"CDS;ID=637290263;locus_tag=SPO2795;product=aminotransferase, DegT/DnrJ/EryC1/StrS	16	10	9	22	22	23
CDS;ID=637290264;locus_tag=SPO2796;product=phosphoglycolate phosphatase	17	16	11	7	23	18
CDS;ID=637290265;locus_tag=SPO2797;product=UDP-N-acetylglucosamine pyrophosphorylas	37	34	29	40	50	40
CDS;ID=637290266;locus_tag=SPO2798;product=hypothetical protein	13	22	23	13	25	16
CDS;ID=637290267;locus_tag=SPO2799;product=molybdenum cofactor biosynthesis protein	77	74	73	84	90	82
CDS;ID=637290268;locus_tag=SPO2800;product=3-deoxy-D-manno-octulosonic acid transfe	25	8	20	11	21	14
CDS;ID=637290269;locus_tag=SPO2801;product=lipopolysaccharide core biosynthesis man	9	7	5	6	6	1
CDS;ID=637290270;locus_tag=SPO2802;product=bmp family protein	38	30	28	21	20	22
"CDS;ID=637290271;locus_tag=SPO2803;product=sugar ABC transporter, ATP-binding prot	14	14	12	8	11	8
"CDS;ID=637290272;locus_tag=SPO2804;product=sugar ABC transporter, permease protein	8	7	8	3	0	0
"CDS;ID=637290273;locus_tag=SPO2805;product=sugar ABC transporter, permease protein	9	5	6	1	6	3
CDS;ID=637290274;locus_tag=SPO2806;product=cytosine deaminase	6	4	10	1	1	5
CDS;ID=637290275;locus_tag=SPO2807;product=hypothetical protein	3	2	1	2	7	1
CDS;ID=637290276;locus_tag=SPO2808;product=amidohydrolase family protein	49	48	52	34	57	23
CDS;ID=637290277;locus_tag=SPO2809;product=amidohydrolase family protein	39	17	28	19	31	27
CDS;ID=637290278;locus_tag=SPO2810;product=amidohydrolase family protein	30	19	12	15	18	15
CDS;ID=637290279;locus_tag=SPO2811;product=amidohydrolase family protein	20	23	20	19	26	31
CDS;ID=637290280;locus_tag=SPO2812;product=acetylornithine deacetylase	40	36	36	36	40	39
"CDS;ID=637290281;locus_tag=SPO2813;product=peptide/nickel/opine uptake family ABC	150	143	123	131	166	154
"CDS;ID=637290282;locus_tag=SPO2814;product=peptide/opine/nickel uptake family ABC	272	243	216	199	289	205
"CDS;ID=637290283;locus_tag=SPO2815;product=peptide/nickel/opine uptake family ABC	21	27	14	19	25	19
"CDS;ID=637290284;locus_tag=SPO2816;product=peptide/nickel/opine uptake family ABC	14	11	8	10	14	13
CDS;ID=637290285;locus_tag=SPO2817;product=YeeE/YedE family protein	20	18	17	9	11	21
CDS;ID=637290286;locus_tag=SPO2818;product=hypothetical protein	45	22	43	49	41	51
"CDS;ID=637290287;locus_tag=SPO2819;product=NAD(P)+ transhydrogenase, beta subunit"	62	49	30	41	62	46
CDS;ID=637290288;locus_tag=SPO2820;product=NAD(P) transhydrogenase subunit alpha	103	76	86	77	114	132
CDS;ID=637290289;locus_tag=SPO2821;product=isoprenylcysteine carboxyl methyltransfe	66	46	41	66	41	60
"CDS;ID=637290290;locus_tag=SPO2822;product=soxH protein, homolog"	4	9	14	5	5	9
CDS;ID=637290291;locus_tag=SPO2823;product=D-beta-hydroxybutyrate dehydrogenase	35	32	23	23	13	28
CDS;ID=637290292;locus_tag=SPO2824;product=kynureninase	10	18	7	11	11	11
"CDS;ID=637290293;locus_tag=SPO2825;product=transcriptional regulator, TetR family"	46	58	63	61	40	54
"CDS;ID=637290294;locus_tag=SPO2826;product=isoquinoline 1-oxidoreductase, alpha su	9	14	10	5	8	4
"CDS;ID=637290295;locus_tag=SPO2827;product=isoquinoline 1-oxidoreductase, beta sub	19	16	9	16	19	17
CDS;ID=637290296;locus_tag=SPO2828;product=type I secretion target repeat protein	17	11	9	15	9	9
CDS;ID=637290297;locus_tag=SPO2829;product=dipeptidase domain protein	9	10	7	2	5	9
CDS;ID=637290298;locus_tag=SPO2830;product=hypothetical protein	9	12	8	6	7	8
"CDS;ID=637290299;locus_tag=SPO2831;product=oligopeptide ABC transporter, ATP-bindi	1	1	1	0	0	2
"CDS;ID=637290300;locus_tag=SPO2832;product=dipeptide ABC transporter, ATP-binding	1	2	0	0	1	1
"CDS;ID=637290301;locus_tag=SPO2833;product=dipeptide ABC transporter, permease pro	0	5	0	0	0	0
"CDS;ID=637290302;locus_tag=SPO2834;product=dipeptide ABC trasnporter, permease pro	2	0	0	4	0	0
"CDS;ID=637290303;locus_tag=SPO2835;product=dipeptide ABC transporter, periplasmic	9	2	4	2	7	4

"CDS;ID=637290304;locus_tag=SPO2836;product=DNA-binding protein, putative"	11	4	5	8	5	9
CDS;ID=637290305;locus_tag=SPO2837;product=hypothetical protein	84	86	90	56	68	81
CDS;ID=637290306;locus_tag=SPO2838;product=hypothetical protein	10	10	8	14	24	6
CDS;ID=637290307;locus_tag=SPO2839;product=malate synthase	103	60	57	102	126	119
CDS;ID=637290308;locus_tag=SPO2840;product=GGDEF domain protein	2	3	3	1	2	3
CDS;ID=637290309;locus_tag=SPO2841;product=aminopeptidase N	43	52	25	21	32	45
"CDS;ID=637290310;locus_tag=SPO2842;product=oxidoreductase, short chain dehydrogena	5	5	3	3	3	4
CDS;ID=637290311;locus_tag=SPO2843;product=hypothetical protein	3	4	3	2	4	1
CDS;ID=637290312;locus_tag=SPO2844;product=hypothetical protein	7	7	4	10	8	5
CDS;ID=637290313;locus_tag=SPO2845;product=hypothetical protein	11	8	5	12	11	15
CDS;ID=637290314;locus_tag=SPO2846;product=hypothetical protein	18	9	9	15	11	11
CDS;ID=637290315;locus_tag=SPO2847;product=aspartyl/glutamyl-tRNA amidotransferase	153	136	108	166	172	171
CDS;ID=637290316;locus_tag=SPO2848;product=hypothetical protein	21	16	18	19	26	24
"CDS;ID=637290317;locus_tag=SPO2849;product=BolA protein, truncation"	9	4	3	3	11	4
CDS;ID=637290318;locus_tag=SPO2850;product=DnaJ domain protein	50	29	42	46	60	38
CDS;ID=637290319;locus_tag=SPO2851;product=hypothetical protein	35	21	28	34	20	37
CDS;ID=637290320;locus_tag=SPO2852;product=czcN domain protein	2	4	3	3	1	11
"CDS;ID=637290321;locus_tag=SPO2853;product=cobalt chelatase, CobS subunit"	187	184	124	171	207	166
CDS;ID=637290322;locus_tag=SPO2854;product=thermonuclease	55	86	61	66	64	69
"CDS;ID=637290323;locus_tag=SPO2855;product=cobalt chelatase, pCobT subunit"	198	148	136	125	155	135
CDS;ID=637290324;locus_tag=SPO2856;product=hypothetical protein	16	8	15	11	8	13
"CDS;ID=637290325;locus_tag=SPO2857;product=metallopeptidase, family M24"	114	95	99	80	83	111
CDS;ID=637290326;locus_tag=SPO2858;product=hypothetical protein	37	34	42	39	33	34
CDS;ID=637290327;locus_tag=SPO2859;product=3-hydroxyisobutyrate dehydrogenase fami	2	11	13	14	13	9
"CDS;ID=637290328;locus_tag=SPO2860;product=transcriptional regulator, LysR family'	0	0	1	5	3	1
RNA;ID=641222525;locus_tag=SPO_R0065;product=Cobalamin	150	128	90	117	109	149
CDS;ID=637290329;locus_tag=SPO2861;product=hypothetical protein	29	27	40	26	56	29
CDS;ID=637290330;locus_tag=SPO2862;product=CobW	76	67	58	78	69	61
CDS;ID=637290331;locus_tag=SPO2863;product=cobaltochelatase	168	159	137	154	167	173
CDS;ID=637290332;locus_tag=SPO2864;product=CobG	108	84	80	69	83	92
CDS;ID=637290333;locus_tag=SPO2865;product=precorrin-8X methylmutase	51	39	35	37	45	45
CDS;ID=637290334;locus_tag=SPO2866;product=precorrin-2 C20-methyltransferase	61	51	34	43	33	48
CDS;ID=637290335;locus_tag=SPO2867;product=precorrin-3B C17-methyltransferase	90	57	46	52	41	67
CDS;ID=637290336;locus_tag=SPO2868;product=precorrin-6x reductase	26	27	22	16	25	16
"CDS;ID=637290337;locus_tag=SPO2869;product=precorrin-6Y C5,15-methyltransferase (c	49	43	25	37	34	42
CDS;ID=637290338;locus_tag=SPO2870;product=cobalamin biosynthesis domain protein	30	28	22	24	46	7
CDS;ID=637290339;locus_tag=SPO2871;product=precorrin-4 C11-methyltransferase	44	32	17	20	33	31
"CDS;ID=637290340;locus_tag=SPO2872;product=cobyrinic acid a,c-diamide synthase"	53	54	45	44	51	45
CDS;ID=637290341;locus_tag=SPO2873;product=uroporphyrin-III C-methyltransferase	12	10	3	4	16	3
CDS;ID=637290342;locus_tag=SPO2874;product=precorrin 6A synthase	42	27	25	15	28	31
RNA;ID=641222526;locus_tag=SPO_R0066;product=Cobalamin	189	135	100	120	75	146
CDS;ID=637290343;locus_tag=SPO2875;product=hypothetical protein	12	11	11	12	11	9
CDS;ID=637290344;locus_tag=SPO2876;product=hypothetical protein	39	28	39	46	30	28

CDS;ID=637290345;locus_tag=SPO2877;product=hypothetical protein	26	23	13	15	34	21
CDS;ID=637290346;locus_tag=SPO2878;product=hypothetical protein	22	9	11	13	32	15
"CDS;ID=637290347;locus_tag=SPO2879;product=transcriptional regulator, LysR family'	10	9	10	1	2	6
CDS;ID=637290348;locus_tag=SPO2880;product=hypothetical protein	3	3	3	1	6	0
"CDS;ID=637290349;locus_tag=SPO2881;product=xanthine dehydrogenase family protein,	8	1	5	3	1	1
CDS;ID=637290350;locus_tag=SPO2882;product=dihydrodipicolinate synthase family prot	2	1	1	1	1	1
CDS;ID=637290351;locus_tag=SPO2883;product=hypothetical protein	25	21	18	20	16	24
CDS;ID=637290352;locus_tag=SPO2884;product=hypothetical protein	3	7	3	2	2	5
"CDS;ID=637290353;locus_tag=SPO2885;product=acetolactate synthase, catabolic, putat	10	10	7	7	11	10
CDS;ID=637290354;locus_tag=SPO2886;product=CAIB/BAIF family protein	10	9	8	7	8	9
CDS;ID=637290355;locus_tag=SPO2887;product=hypothetical protein	136	115	101	109	150	109
CDS;ID=637290356;locus_tag=SPO2888;product=hypothetical protein	69	69	61	71	77	57
CDS;ID=637290357;locus_tag=SPO2889;product=periplasmic glucan biosynthesis protein	40	36	17	20	42	20
CDS;ID=637290358;locus_tag=SPO2890;product=glucosyltransferase MdoH	90	49	52	57	76	61
CDS;ID=637290359;locus_tag=SPO2891;product=hypothetical protein	7	4	3	7	4	3
CDS;ID=637290360;locus_tag=SPO2892;product=hypothetical protein	10	10	6	7	5	4
CDS;ID=637290361;locus_tag=SPO2893;product=pH adaption potassium efflux system prot	71	59	59	60	83	73
CDS;ID=637290362;locus_tag=SPO2894;product=hypothetical protein	9	8	6	12	9	3
CDS;ID=637290363;locus_tag=SPO2895;product=NADH dehydrogenase subunit N	16	18	5	21	9	20
CDS;ID=637290364;locus_tag=SPO2896;product=pH adaption potassium efflux system prot	9	5	1	8	8	8
CDS;ID=637290365;locus_tag=SPO2897;product=pH adaptation potassium efflux system pr	6	5	4	3	7	4
CDS;ID=637290366;locus_tag=SPO2898;product=pH adaption potassium efflux system prot	5	6	4	5	16	2
"CDS;ID=637290367;locus_tag=SPO2899;product=selenide,water dikinase, putative"	11	4	5	9	6	5
CDS;ID=637290368;locus_tag=SPO2900;product=hypothetical protein	12	8	5	11	13	15
CDS;ID=637290369;locus_tag=SPO2901;product=hypothetical protein	3	4	3	5	2	2
CDS;ID=637290370;locus_tag=SPO2902;product=glutathione S-transferase family protein	6	3	6	4	4	6
CDS;ID=637290371;locus_tag=SPO2903;product=TPR/sulfotransferase domain protein	59	41	42	52	52	63
"CDS;ID=637290372;locus_tag=SPO2904;product=Ser/Thr protein phosphatase/nucleotidas	25	36	35	24	31	46
CDS;ID=637290373;locus_tag=SPO2905;product=hypothetical protein	14	28	9	24	29	15
CDS;ID=637290374;locus_tag=SPO2906;product=hypothetical protein	8	17	11	18	23	15
CDS;ID=637290375;locus_tag=SPO2907;product=dihydroorotate dehydrogenase	43	42	16	42	43	44
CDS;ID=637290376;locus_tag=SPO2908;product=arsenate reductase	2	11	3	5	3	6
CDS;ID=637290377;locus_tag=SPO2909;product=DNA-damage-inducible protein F	15	21	9	15	21	22
CDS;ID=637290378;locus_tag=SPO2910;product=thioesterase family protein	8	7	17	8	12	8
CDS;ID=637290379;locus_tag=SPO2911;product=thioesterase family protein	16	19	4	14	16	24
"CDS;ID=637290380;locus_tag=SPO2912;product=transcriptional regulator, merR family'	143	111	131	154	118	136
CDS;ID=637290381;locus_tag=SPO2913;product=hypothetical protein	1	0	1	3	1	4
"CDS;ID=637290382;locus_tag=SPO2914;product=transcriptional regulator, merR family'	59	21	42	121	133	98
CDS;ID=637290383;locus_tag=SPO2915;product=acyl-CoA dehydrogenase family protein	37	40	45	74	153	104
CDS;ID=637290384;locus_tag=SPO2916;product=hypothetical protein	7	6	2	4	21	8
CDS;ID=637290385;locus_tag=SPO2917;product=glutathione S-transferase family protein	8	3	13	4	10	11
CDS;ID=637290386;locus_tag=SPO2918;product=acetyl-CoA acetyltransferase	6	12	11	5	17	16
CDS;ID=637290387;locus_tag=SPO2919;product=hypothetical protein	7	8	5	9	15	8

"CDS;ID=637290388;locus_tag=SPO2920;product=fatty oxidation complex, alpha subunit'	33	47	29	29	43	35
CDS;ID=637290389;locus_tag=SPO2921;product=hypothetical protein	7	8	1	6	4	4
CDS;ID=637290390;locus_tag=SPO2922;product=hypothetical protein	6	6	1	8	2	5
CDS;ID=637290391;locus_tag=SPO2923;product=hypothetical protein	5	2	4	13	6	8
CDS;ID=637290392;locus_tag=SPO2924;product=hypothetical protein	72	68	53	81	65	79
CDS;ID=637290393;locus_tag=SPO2925;product=hypothetical protein	69	45	50	33	37	58
CDS;ID=637290394;locus_tag=SPO2926;product=uracil phosphoribosyltransferase	22	25	23	26	23	19
CDS;ID=637290395;locus_tag=SPO2927;product=adenosine deaminase	47	48	36	35	39	46
CDS;ID=637290396;locus_tag=SPO2928;product=phosphopentomutase	29	30	24	27	28	26
CDS;ID=637290397;locus_tag=SPO2929;product=thymidine phosphorylase	38	28	21	27	27	36
CDS;ID=637290398;locus_tag=SPO2930;product=cytidine deaminase	8	12	11	14	11	18
CDS;ID=637290399;locus_tag=SPO2931;product=hypothetical protein	31	24	47	33	44	31
CDS;ID=637290400;locus_tag=SPO2932;product=phosphate acetyltransferase	64	53	51	70	92	90
CDS;ID=637290401;locus_tag=SPO2933;product=hypothetical protein	5	1	3	7	6	5
CDS;ID=637290402;locus_tag=SPO2934;product=propionate--CoA ligase	72	52	26	54	47	56
CDS;ID=637290403;locus_tag=SPO2936;product=hypothetical protein	7	4	5	4	4	3
CDS;ID=637290404;locus_tag=SPO2935;product=hypothetical protein	3	2	2	1	1	0
CDS;ID=637290405;locus_tag=SPO2937;product=SCO1/SenC family protein	2	5	1	3	0	4
CDS;ID=637290406;locus_tag=SPO2938;product=amidase	27	26	32	18	34	27
"CDS;ID=637290407;locus_tag=SPO2939;product=ATP-NAD kinase, putative"	39	35	31	21	11	16
CDS;ID=637290408;locus_tag=SPO2940;product=serine hydroxymethyltransferase	68	40	48	47	52	51
CDS;ID=637290409;locus_tag=SPO2941;product=hypothetical protein	8	8	1	11	13	8
CDS;ID=637290410;locus_tag=SPO2942;product=hypothetical protein	15	12	7	21	10	9
"CDS;ID=637290411;locus_tag=SPO2943;product=hydrolase, alpha/beta fold family"	9	8	16	21	20	21
CDS;ID=637290412;locus_tag=SPO2944;product=hypothetical protein	0	1	1	2	4	2
CDS;ID=637290413;locus_tag=SPO2945;product=rhodanese-like domain protein	7	8	9	5	6	6
CDS;ID=637290414;locus_tag=SPO2946;product=monooxygenase domain protein	14	1	6	3	3	4
CDS;ID=637290415;locus_tag=SPO2947;product=cupin family protein	7	0	3	2	3	2
"CDS;ID=637290416;locus_tag=SPO2948;product=transcriptional regulator, AraC family'	4	18	9	5	8	12
CDS;ID=637290417;locus_tag=SPO2949;product=endoribonuclease L-PSP family protein	36	24	17	20	21	34
CDS;ID=637290418;locus_tag=SPO2950;product=hypothetical protein	29	19	21	19	20	22
CDS;ID=637290419;locus_tag=SPO2951;product=hypothetical protein	36	33	22	25	30	31
CDS;ID=637290420;locus_tag=SPO2952;product=trkA domain protein	40	33	25	30	25	33
CDS;ID=637290421;locus_tag=SPO2953;product=Ser/Thr protein phosphatase family prote	46	55	49	59	54	93
CDS;ID=637290422;locus_tag=SPO2954;product=5-formyltetrahydrofolate cyclo-ligase fa	7	6	3	10	5	11
CDS;ID=637290423;locus_tag=SPO2955;product=magnesium transporter	22	35	20	40	38	43
CDS;ID=637290424;locus_tag=SPO2956;product=guanine deaminase	91	66	46	60	63	71
CDS;ID=637290425;locus_tag=SPO2957;product=hydroxydechloroatrazine ethylaminohydro	36	24	17	17	16	22
CDS;ID=637290426;locus_tag=SPO2958;product=inositol monophosphatase family protein	50	58	68	66	31	62
"CDS;ID=637290427;locus_tag=SPO2959;product=DNA-binding protein, putative"	184	161	151	125	127	180
"CDS;ID=637290428;locus_tag=SPO2960;product=oxidoreductase, zinc-binding dehydroger	37	50	27	33	37	42
"CDS;ID=637290429;locus_tag=SPO2961;product=transcriptional regulator, LysR family'	27	28	32	26	35	55
CDS;ID=637290430;locus_tag=SPO2962;product=hypothetical protein	3	1	1	3	1	2

CDS;ID=637290431;locus_tag=SPO2963;product=hypothetical protein	204	180	154	175	187	174
CDS;ID=637290432;locus_tag=SPO2964;product=hypothetical protein	42	49	32	30	24	28
CDS;ID=637290433;locus_tag=SPO2965;product=50S ribosomal protein L33	181	164	131	226	112	191
RNA;ID=640698548;locus_tag=SPO_SprnpB1	19971	20790	15735	17064	10703	21020
"CDS;ID=637290434;locus_tag=SPO2967;product=N-acetylmuramoyl-L-alanine amidase, put	22	27	18	14	22	16
CDS;ID=637290435;locus_tag=SPO2968;product=hypothetical protein	117	86	89	89	117	122
CDS;ID=637290436;locus_tag=SPO2969;product=glutamyl-tRNA amidotransferase subunit A	72	91	74	80	86	91
"CDS;ID=637290437;locus_tag=SPO2970;product=glutamyl-tRNA(Gln) amidotransferase, C	89	89	82	82	93	73
CDS;ID=637290438;locus_tag=SPO2971;product=hypothetical protein	3	10	7	13	11	12
CDS;ID=637290439;locus_tag=SPO2972;product=hypothetical protein	43	16	21	12	12	6
CDS;ID=640735073;locus_tag=SPO2973	2	3	2	3	0	2
CDS;ID=637290440;locus_tag=SPO2974;product=hypothetical protein	9	12	12	6	16	10
CDS;ID=637290441;locus_tag=SPO2975;product=hypothetical protein	3	2	1	3	0	1
CDS;ID=637290442;locus_tag=SPO2976;product=metallo-beta-lactamase family protein	29	21	30	12	35	29
CDS;ID=637290443;locus_tag=SPO2977;product=adenylate/guanylate cyclase	7	1	4	3	6	12
CDS;ID=637290444;locus_tag=SPO2978;product=adenylate/guanylate cyclase	9	8	4	5	6	8
CDS;ID=637290445;locus_tag=SPO2979;product=hypothetical protein	3	5	1	0	1	2
CDS;ID=637290446;locus_tag=SPO2980;product=porphyromonas-type peptidyl-arginine dei	3	4	6	12	9	6
CDS;ID=637290447;locus_tag=SPO2981;product=hypothetical protein	40	44	24	19	31	38
CDS;ID=637290448;locus_tag=SPO2982;product=hypothetical protein	10	3	7	8	5	6
CDS;ID=637290449;locus_tag=SPO2983;product=cytidine and deoxycytidylate deaminase f	19	15	13	13	13	14
CDS;ID=637290450;locus_tag=SPO2984;product=RNA pseudouridylate synthase family prot	81	55	55	50	58	66
CDS;ID=637290451;locus_tag=SPO2985;product=hypothetical protein	46	40	21	21	40	47
CDS;ID=637290452;locus_tag=SPO2986;product=tellurite resistance protein	51	61	46	30	66	46
"CDS;ID=637290453;locus_tag=SPO2987;product=lipoprotein, putative"	36	21	23	32	32	13
CDS;ID=637290454;locus_tag=SPO2988;product=hypothetical protein	48	29	32	37	49	35
CDS;ID=637290455;locus_tag=SPO2989;product=cytochrome b562	16	9	10	10	4	7
CDS;ID=637290456;locus_tag=SPO2990;product=hypothetical protein	34	27	21	27	21	31
CDS;ID=640735074;locus_tag=SPO3904	107	73	112	138	152	185
CDS;ID=637290457;locus_tag=SPO2991;product=nitroreductase family protein	9	11	2	4	8	8
CDS;ID=637290458;locus_tag=SPO2992;product=D-tyrosyl-tRNA deacylase	4	1	1	2	0	3
CDS;ID=637290459;locus_tag=SPO2993;product=MOFRL family protein	8	6	3	2	3	7
CDS;ID=637290460;locus_tag=SPO2994;product=X-Pro dipeptidyl-peptidase family prote:	44	57	40	29	49	43
"CDS;ID=637290461;locus_tag=SPO2995;product=peptide/nickel/opine uptake family ABC	159	166	98	105	125	91
"CDS;ID=637290462;locus_tag=SPO2996;product=peptide/nickel/opine uptake family ABC	39	24	18	17	30	20
"CDS;ID=637290463;locus_tag=SPO2997;product=peptide/nickel/opine uptake family ABC	24	16	8	10	7	8
"CDS;ID=637290464;locus_tag=SPO2998;product=peptide/nickel/opine uptake family ABC	108	110	90	68	73	70
CDS;ID=637290465;locus_tag=SPO2999;product=hypothetical protein	12	6	5	7	14	13
CDS;ID=637290466;locus_tag=SPO3000;product=hypothetical protein	12	21	15	22	20	21
CDS;ID=637290467;locus_tag=SPO3001;product=hypothetical protein	4	10	3	10	6	9
"CDS;ID=637290468;locus_tag=SPO3002;product=lipase, putative"	7	14	2	10	5	9
CDS;ID=637290469;locus_tag=SPO3003;product=acyl-CoA synthase	71	56	46	48	39	50
CDS;ID=637290470;locus_tag=SPO3004;product=LysM/phospholipid-binding domain protein	115	58	59	44	77	68

"CDS;ID=637290471;locus_tag=SPO3005;product=ABC transporter, ATP binding/permease p	52	46	51	63	78	76
tRNA;ID=640698549;locus_tag=SPO_tRNA-Gly-6	26	7	1	7	3	20
tRNA;ID=640698550;locus_tag=SPO_tRNA-Leu-5	10	12	3	10	4	11
CDS;ID=637290474;locus_tag=SPO3008;product=TPR domain protein	9	8	7	5	3	3
CDS;ID=637290475;locus_tag=SPO3009;product=proline dehydrogenase transcriptional ac	5	5	4	2	6	12
CDS;ID=637290476;locus_tag=SPO3010;product=bifunctional PutA protein	43	46	36	46	62	69
CDS;ID=637290477;locus_tag=SPO3011;product=rhomboid family protein	25	21	11	14	26	32
"CDS;ID=637290478;locus_tag=SPO3012;product=inositol-1-monophosphatase, putative"	115	123	116	193	241	150
"CDS;ID=637290479;locus_tag=SPO3013;product=NADP-dependent oxidoreductase, L4bD far	5	6	5	4	7	6
CDS;ID=637290480;locus_tag=SPO3014;product=hypothetical protein	5	9	5	10	11	6
CDS;ID=637290481;locus_tag=SPO3015;product=transcriptional regulator MetR	35	58	18	29	40	36
"CDS;ID=637290482;locus_tag=SPO3016;product=5,10-methylenetetrahydrofolate reductas	40	12	11	14	16	20
CDS;ID=637290483;locus_tag=SPO3017;product=thioesterase family protein	16	9	10	8	8	8
CDS;ID=637290484;locus_tag=SPO3018;product=hypothetical protein	14	23	13	15	9	13
"CDS;ID=637290485;locus_tag=SPO3019;product=xanthine dehydrogenase family protein,	82	79	54	52	67	58
"CDS;ID=637290486;locus_tag=SPO3020;product=transcriptional regulator, LysR family"	13	12	8	8	6	11
CDS;ID=637290487;locus_tag=SPO3021;product=phytanoyl-CoA dioxygenase family protein	2	0	0	1	1	1
CDS;ID=637290488;locus_tag=SPO3022;product=valyl-tRNA synthetase	130	80	80	105	120	101
CDS;ID=637290489;locus_tag=SPO3023;product=hypothetical protein	6	4	1	5	11	2
"CDS;ID=637290490;locus_tag=SPO3024;product=adenylate/guanylate cyclase/hydrolase,	14	6	2	9	7	13
CDS;ID=637290491;locus_tag=SPO3025;product=enoyl-CoA hydratase	31	27	13	35	36	39
CDS;ID=637290492;locus_tag=SPO3026;product=strictosidine synthase family protein	23	25	27	24	18	38
CDS;ID=637290493;locus_tag=SPO3027;product=histidinol-phosphate aminotransferase	39	41	39	32	33	56
CDS;ID=637290494;locus_tag=SPO3028;product=hypothetical protein	6	1	2	3	3	4
"CDS;ID=637290495;locus_tag=SPO3029;product=serine--glyoxylate transaminase, putati	37	42	33	44	47	49
"CDS;ID=637290496;locus_tag=SPO3030;product=efflux ABC transporter, transmembrane A	70	62	61	67	46	57
CDS;ID=637290497;locus_tag=SPO3031;product=2-dehydro-3-deoxyphosphogluconate aldola	24	24	20	36	28	36
CDS;ID=637290498;locus_tag=SPO3032;product=phosphogluconate dehydratase	64	38	35	59	49	70
CDS;ID=637290499;locus_tag=SPO3033;product=glucose-6-phosphate 1-dehydrogenase	109	140	95	125	135	169
CDS;ID=637290500;locus_tag=SPO3034;product=phosphoenolpyruvate-protein phosphotrans	92	96	97	66	72	105
"CDS;ID=637290501;locus_tag=SPO3035;product=aspartate kinase, monofunctional class"	118	84	78	104	75	111
CDS;ID=637290502;locus_tag=SPO3036;product=metallo-beta-lactamase family protein	278	174	222	220	297	288
"CDS;ID=637290503;locus_tag=SPO3037;product=DNA-binding response regulator, LysR fa	14	14	8	7	16	7
CDS;ID=637290504;locus_tag=SPO3038;product=sensor histidine kinase/response regulat	44	57	35	42	45	53
"CDS;ID=637290505;locus_tag=SPO3039;product=polar amino acid uptake family ABC tran	11	13	5	15	15	10
"CDS;ID=637290506;locus_tag=SPO3040;product=polar amino acid uptake family ABC tran	18	17	19	24	19	27
"CDS;ID=637290507;locus_tag=SPO3041;product=polar amino acid uptake family ABC tran	4	2	5	5	2	8
"CDS;ID=637290508;locus_tag=SPO3042;product=polar amino acid uptake family ABC tran	7	10	16	20	18	16
"CDS;ID=637290509;locus_tag=SPO3043;product=glutamine ABC transporter, ATP-binding	30	34	36	41	29	36
"CDS;ID=637290510;locus_tag=SPO3044;product=transcriptional regulator, LysR family"	6	5	7	9	6	9
"CDS;ID=637290511;locus_tag=SPO3045;product=glutamine amidotransferase, class I"	8	10	8	13	8	5
"CDS;ID=637290512;locus_tag=SPO3046;product=oligopeptide ABC transporter, ATP-bindi	18	7	7	9	7	12
"CDS;ID=637290513;locus_tag=SPO3047;product=dipeptide ABC transporter, permease pro	14	6	5	5	5	4

"CDS;ID=637290514;locus_tag=SPO3048;product=dipeptide ABC transporter, permease pro	9	4	7	5	8	6
"CDS;ID=637290515;locus_tag=SPO3049;product=dipeptide ABC transporter, periplasmic	11	5	7	5	3	12
CDS;ID=637290516;locus_tag=SPO3050;product=hypothetical protein	3	1	2	1	2	3
"CDS;ID=637290517;locus_tag=SPO3051;product=oxidoreductase, short-chain dehydrogena	17	14	8	7	16	15
CDS;ID=637290518;locus_tag=SPO3052;product=hypothetical protein	43	34	39	40	44	46
CDS;ID=637290519;locus_tag=SPO3053;product=NUDIX domain protein	7	7	3	7	3	5
CDS;ID=637290520;locus_tag=SPO3054;product=hypothetical protein	18	24	12	17	13	25
"CDS;ID=637290521;locus_tag=SPO3055;product=acetyltransferase, GNAT family"	48	32	21	26	16	32
CDS;ID=637290522;locus_tag=SPO3056;product=hypothetical protein	14	17	16	18	20	17
"CDS;ID=637290523;locus_tag=SPO3057;product=streptogramin acetyltransferase, putati	16	12	14	10	9	8
CDS;ID=637290524;locus_tag=SPO3058;product=sulfate transporter family protein	76	75	67	95	98	86
CDS;ID=637290525;locus_tag=SPO3059;product=hypothetical protein	4	5	1	5	6	5
CDS;ID=637290526;locus_tag=SPO3060;product=5'-methylthioadenosine phosphorylase	15	10	12	10	12	12
CDS;ID=640735075;locus_tag=SPO3061	7	11	0	11	5	5
CDS;ID=637290527;locus_tag=SPO3062;product=hypothetical protein	4	1	0	1	5	4
"CDS;ID=637290528;locus_tag=SPO3063;product=transmembrane efflux protein, homoserin	11	11	8	6	17	13
CDS;ID=637290529;locus_tag=SPO3064;product=hypothetical protein	20	16	13	8	17	15
CDS;ID=637290530;locus_tag=SPO3065;product=hypothetical protein	13	10	9	11	25	15
CDS;ID=637290531;locus_tag=SPO3066;product=adenine phosphoribosyltransferase	39	43	17	47	45	58
"CDS;ID=637290532;locus_tag=SPO3067;product=oxidoreductase, FAD-binding"	32	37	21	22	33	35
CDS;ID=637290533;locus_tag=SPO3068;product=hypothetical protein	4	6	2	3	3	3
"CDS;ID=637290534;locus_tag=SPO3069;product=ribosomal-protein-alanine acetyltransfe	22	30	14	19	21	19
"CDS;ID=637290535;locus_tag=SPO3070;product=peptidase, M16 family"	124	110	78	86	126	97
CDS;ID=637290536;locus_tag=SPO3071;product=threonine synthase	53	54	44	56	46	62
CDS;ID=637290537;locus_tag=SPO3072;product=SURF1 family protein	22	19	10	17	25	22
"CDS;ID=637290538;locus_tag=SPO3073;product=cytochrome c oxidase, subunit III"	44	36	33	37	52	51
CDS;ID=637290539;locus_tag=SPO3074;product=cytochrome C oxidase assembly protein	53	48	40	52	38	53
CDS;ID=637290540;locus_tag=SPO3075;product=protoheme IX farnesyltransferase	40	26	20	20	34	21
"CDS;ID=637290541;locus_tag=SPO3076;product=cytochrome c oxidase, subunit II"	53	68	56	51	55	64
CDS;ID=637290542;locus_tag=SPO3077;product=TldD/PmbA family protein	22	21	14	24	13	17
"CDS;ID=637290543;locus_tag=SPO3078;product=DNA processing protein DprA, putative"	47	20	29	27	33	42
CDS;ID=637290544;locus_tag=SPO3079;product=DNA topoisomerase I	340	243	285	273	319	294
"CDS;ID=637290545;locus_tag=SPO3080;product=transcriptional regulator, TetR family"	21	8	10	21	25	21
CDS;ID=637290546;locus_tag=SPO3081;product=glyoxalase family protein	41	9	35	37	51	50
"CDS;ID=637290547;locus_tag=SPO3082;product=3-oxoadipate CoA-succinyl transferase,	119	108	117	108	133	132
"CDS;ID=637290548;locus_tag=SPO3083;product=3-oxoadipate CoA-succinyl transferase,	110	82	103	75	107	96
"CDS;ID=637290549;locus_tag=SPO3084;product=transglycosylase, Slt family"	42	29	39	36	51	48
CDS;ID=637290550;locus_tag=SPO3085;product=hypothetical protein	165	174	137	168	123	151
CDS;ID=637290551;locus_tag=SPO3086;product=CpaB family protein	30	28	39	35	29	32
CDS;ID=637290552;locus_tag=SPO3087;product=bacterial type II/III secretion system p	121	129	113	127	147	122
CDS;ID=637290553;locus_tag=SPO3088;product=OmpA family protein	69	49	65	74	99	78
"CDS;ID=637290554;locus_tag=SPO3089;product=ATPase, putative"	33	31	36	46	36	30
"CDS;ID=637290555;locus_tag=SPO3090;product=type II/IV secretion system protein, Ta	15	22	14	14	22	25

"CDS;ID=637290556;locus_tag=SPO3091;product=type II/IV secretion system protein, Ta	29	19	20	31	53	39
"CDS;ID=637290557;locus_tag=SPO3092;product=type II/IV secretion system protein, Ta	18	10	9	12	14	8
CDS;ID=637290558;locus_tag=SPO3093;product=TPR domain protein	74	107	83	74	91	63
"CDS;ID=637290559;locus_tag=SPO3094;product=lipoprotein, putative"	80	88	54	66	64	89
CDS;ID=637290560;locus_tag=SPO3095;product=hypothetical protein	15	11	19	10	19	18
CDS;ID=637290561;locus_tag=SPO3096;product=hypothetical protein	22	30	25	24	37	20
CDS;ID=637290562;locus_tag=SPO3097;product=3-hydroxyisobutyrate dehydrogenase	10	10	6	7	11	7
"CDS;ID=637290563;locus_tag=SPO3098;product=ATP-dependent helicase, DEAD/DEAH box f	11	9	7	2	11	6
CDS;ID=637290564;locus_tag=SPO3099;product=hypothetical protein	21	19	11	20	9	16
CDS;ID=637290565;locus_tag=SPO3100;product=hypothetical protein	36	39	28	27	35	37
CDS;ID=637290566;locus_tag=SPO3101;product=folD bifunctional protein	59	45	47	62	87	67
CDS;ID=637290567;locus_tag=SPO3102;product=chorismate mutase family protein	28	22	12	29	35	31
CDS;ID=637290568;locus_tag=SPO3103;product=formate--tetrahydrofolate ligase	213	142	164	182	232	197
CDS;ID=637290569;locus_tag=SPO3104;product=MOSC domain protein	6	8	8	1	11	15
CDS;ID=637290570;locus_tag=SPO3105;product=ATP-dependent metalloprotease FtsH	312	295	234	245	243	272
CDS;ID=637290571;locus_tag=SPO3106;product=PP-loop family protein	33	27	12	15	17	23
CDS;ID=637290572;locus_tag=SPO3107;product=hypothetical protein	105	94	62	54	93	93
CDS;ID=637290573;locus_tag=SPO3108;product=peptidoglycan-associated lipoprotein	125	91	127	108	121	96
CDS;ID=637290574;locus_tag=SPO3109;product=translocation protein TolB precursor	93	70	77	70	76	63
"CDS;ID=637290575;locus_tag=SPO3110;product=tonB domain protein, putative"	59	46	41	36	49	57
"CDS;ID=637290576;locus_tag=SPO3111;product=biopolymer transport protein, ExbD/TolF	40	26	33	25	30	44
CDS;ID=637290577;locus_tag=SPO3112;product=proton transporter TolQ	70	54	54	55	74	83
CDS;ID=637290578;locus_tag=SPO3113;product=conserved hypothetical protein TIGR0005	6	4	10	13	7	11
CDS;ID=637290579;locus_tag=SPO3114;product=hypothetical protein	8	12	16	12	8	18
CDS;ID=637290580;locus_tag=SPO3115;product=Holliday junction DNA helicase RuvB	21	10	9	6	8	17
CDS;ID=637290581;locus_tag=SPO3116;product=Holliday junction DNA helicase motor pro	22	15	25	17	17	18
CDS;ID=637290582;locus_tag=SPO3117;product=Holliday junction resolvase	80	79	85	77	58	74
CDS;ID=637290583;locus_tag=SPO3118;product=hypothetical protein	100	57	105	125	177	61
CDS;ID=637290584;locus_tag=SPO3119;product=hypothetical protein	57	71	55	56	54	72
"CDS;ID=637290585;locus_tag=SPO3120;product=ribosomal protein L11 methyltransferase	30	36	29	36	44	43
CDS;ID=637290586;locus_tag=SPO3121;product=MATE efflux family protein	26	12	14	29	25	35
CDS;ID=637290587;locus_tag=SPO3122;product=hypothetical protein	18	22	14	15	12	20
CDS;ID=637290588;locus_tag=SPO3123;product=YeeE/YedE family protein	16	4	12	17	12	12
CDS;ID=637290589;locus_tag=SPO3124;product=hypothetical protein	1	3	1	3	2	0
"CDS;ID=637290590;locus_tag=SPO3125;product=RNA polymerase sigma-70 factor, ECF far	10	3	0	2	1	6
"CDS;ID=637290591;locus_tag=SPO3126;product=sigma factor regulator PrtR, putative"	2	3	0	3	1	5
CDS;ID=637290592;locus_tag=SPO3127;product=primosomal protein N'	47	51	28	34	58	53
CDS;ID=637290593;locus_tag=SPO3128;product=transaldolase	45	42	48	75	77	46
CDS;ID=637290594;locus_tag=SPO3129;product=hypothetical protein	35	40	22	39	37	31
CDS;ID=637290595;locus_tag=SPO3130;product=tyrosine recombinase	23	11	6	7	14	11
CDS;ID=637290596;locus_tag=SPO3131;product=phosphatidylcholine synthase	14	26	15	16	23	23
CDS;ID=637290597;locus_tag=SPO3132;product=hypothetical protein	14	14	21	24	34	25
CDS;ID=637290598;locus_tag=SPO3133;product=hypothetical protein	143	99	148	202	195	215

CDS;ID=637290599;locus_tag=SPO3134;product=methylated-DNA--protein-cysteine methylt	29	20	13	18	16	12
"CDS;ID=637290600;locus_tag=SPO3135;product=peptidase, T4 family"	48	69	56	61	58	68
CDS;ID=637290601;locus_tag=SPO3136;product=isoleucyl-tRNA synthetase	199	148	150	190	212	164
CDS;ID=637290602;locus_tag=SPO3137;product=hypothetical protein	22	28	28	27	48	23
CDS;ID=637290603;locus_tag=SPO3138;product=hypothetical protein	19	17	22	33	35	31
CDS;ID=637290604;locus_tag=SPO3139;product=hypothetical protein	12	8	10	10	14	6
CDS;ID=637290605;locus_tag=SPO3140;product=hypothetical protein	16	11	12	11	14	16
"CDS;ID=637290606;locus_tag=SPO3141;product=acetyltransferase, GNAT family"	12	16	7	15	8	11
CDS;ID=637290607;locus_tag=SPO3142;product=hypothetical protein	19	18	10	13	21	13
CDS;ID=637290608;locus_tag=SPO3143;product=tRNA pseudouridine synthase A	19	9	24	38	18	15
CDS;ID=637290609;locus_tag=SPO3144;product=hypothetical protein	10	10	9	5	4	7
CDS;ID=637290610;locus_tag=SPO3145;product=GumN family protein	18	15	14	12	20	4
CDS;ID=637290611;locus_tag=SPO3146;product=GumN family protein	18	5	21	15	18	15
CDS;ID=637290612;locus_tag=SPO3147;product=putative manganese-dependent inorganic p	61	57	63	60	80	52
CDS;ID=637290613;locus_tag=SPO3148;product=hypothetical protein	5	18	10	10	7	5
CDS;ID=637290614;locus_tag=SPO3149;product=hypothetical protein	0	0	0	0	0	1
"CDS;ID=637290615;locus_tag=SPO3150;product=transcriptional regulator, AraC family"	5	4	3	4	0	2
"CDS;ID=637290616;locus_tag=SPO3151;product=HAD-superfamily subfamily IIA hydrolase	38	40	33	34	45	45
CDS;ID=637290617;locus_tag=SPO3152;product=MaoC domain protein	18	10	4	12	14	18
"CDS;ID=637290618;locus_tag=SPO3153;product=monooxygenase, putative"	5	0	3	2	0	4
CDS;ID=637290619;locus_tag=SPO3154;product=riboflavin biosynthesis protein RibF	29	37	35	38	45	40
CDS;ID=637290620;locus_tag=SPO3155;product=hypothetical protein	27	31	34	16	56	31
"CDS;ID=637290621;locus_tag=SPO3156;product=L-threonine aldolase, low-specificity,	22	29	27	17	34	27
"CDS;ID=637290622;locus_tag=SPO3157;product=hydrolase, alpha/beta fold family"	29	29	21	19	20	27
CDS;ID=637290623;locus_tag=SPO3158;product=2-hydroxychromene-2-carboxylate isomeras	50	46	37	17	38	30
CDS;ID=637290624;locus_tag=SPO3159;product=ribose-phosphate pyrophosphokinase	79	58	49	65	58	62
CDS;ID=637290625;locus_tag=SPO3160;product=hypothetical protein	11	16	6	5	7	11
"CDS;ID=637290626;locus_tag=SPO3161;product=ATP synthase F1, epsilon subunit"	45	64	37	38	36	45
CDS;ID=637290627;locus_tag=SPO3162;product=ATP synthase subunit B	294	261	233	243	349	281
CDS;ID=637290628;locus_tag=SPO3163;product=ATP synthase subunit C	296	308	285	330	360	321
CDS;ID=637290629;locus_tag=SPO3164;product=ATP synthase subunit A	314	298	288	289	438	292
CDS;ID=637290630;locus_tag=SPO3165;product=ATP synthase delta chain	156	101	143	130	178	132
CDS;ID=637290631;locus_tag=SPO3166;product=hypothetical protein	219	196	230	189	216	197
CDS;ID=637290632;locus_tag=SPO3167;product=hypothetical protein	18	16	13	13	16	13
"CDS;ID=637290633;locus_tag=SPO3168;product=hydroxyacylglutathione hydrolase, putat	18	32	23	37	41	23
"CDS;ID=637290634;locus_tag=SPO3169;product=ATP-dependent Clp protease, ATP-binding	207	162	155	169	210	184
"CDS;ID=637290635;locus_tag=SPO3170;product=peptidase, M23/M37 family"	12	7	6	9	23	7
CDS;ID=637290636;locus_tag=SPO3171;product=flagellar motor protein	42	32	47	44	52	39
CDS;ID=637290637;locus_tag=SPO3172;product=hypothetical protein	26	18	20	27	40	43
"CDS;ID=637290638;locus_tag=SPO3173;product=cation transport protein ChaC, putative	20	15	24	11	14	36
CDS;ID=637290639;locus_tag=SPO3174;product=hypothetical protein	9	18	19	9	14	17
CDS;ID=637290640;locus_tag=SPO3175;product=hypothetical protein	22	19	15	23	23	22
CDS;ID=637290641;locus_tag=SPO3176;product=prephenate dehydrogenase	31	18	15	24	28	29

CDS;ID=637290642;locus_tag=SPO3177;product=histidinol-phosphate aminotransferase	18	22	4	14	30	22
CDS;ID=637290643;locus_tag=SPO3178;product=hypothetical protein	3	1	0	4	0	3
"CDS;ID=637290644;locus_tag=SPO3179;product=transcriptional regulator, AsnC family'	3	9	3	4	6	7
CDS;ID=637290645;locus_tag=SPO3180;product=ribose 5-phosphate isomerase B	8	8	8	6	14	16
CDS;ID=637290646;locus_tag=SPO3181;product=30S ribosomal protein S4	91	72	104	92	97	105
CDS;ID=637290647;locus_tag=SPO3182;product=hypothetical protein	71	48	49	64	62	96
"CDS;ID=637290648;locus_tag=SPO3183;product=oxidoreductase, molybdopterin-binding"	2	1	6	2	5	16
"CDS;ID=637290649;locus_tag=SPO3184;product=acetyltransferase, GNAT family"	5	2	5	3	1	2
"CDS;ID=637290650;locus_tag=SPO3185;product=transcriptional regulator, GntR family'	2	6	4	1	1	2
CDS;ID=637290651;locus_tag=SPO3186;product=glycine betaine transporter	19	37	17	24	22	27
CDS;ID=637290652;locus_tag=SPO3187;product=malate/L-lactate dehydrogenase family pı	5	0	1	1	1	1
CDS;ID=637290653;locus_tag=SPO3188;product=hypothetical protein	0	1	1	1	0	4
CDS;ID=637290654;locus_tag=SPO3189;product=hypothetical protein	3	2	1	0	4	4
CDS;ID=637290655;locus_tag=SPO3190;product=renal dipeptidase family protein	4	7	3	3	6	3
CDS;ID=637290656;locus_tag=SPO3191;product=aldehyde dehydrogenase family protein	7	10	9	3	1	6
"CDS;ID=637290657;locus_tag=SPO3192;product=transcriptional regulator, LysR family'	1	5	5	7	6	6
CDS;ID=637290658;locus_tag=SPO3193;product=hypothetical protein	99	88	114	120	111	128
"CDS;ID=637290659;locus_tag=SPO3194;product=ribonucleoside-diphosphate reductase, p	99	72	59	102	95	75
CDS;ID=637290660;locus_tag=SPO3195;product=histone deacetylase family protein	14	17	15	11	15	9
CDS;ID=637290661;locus_tag=SPO3196;product=DNA repair protein RecO	9	7	6	3	4	2
CDS;ID=637290662;locus_tag=SPO3197;product=hypothetical protein	15	7	7	8	10	5
CDS;ID=637290663;locus_tag=SPO3198;product=ribonuclease III	29	23	15	34	32	24
CDS;ID=637290664;locus_tag=SPO3199;product=signal peptidase I	86	72	84	78	85	130
CDS;ID=637290665;locus_tag=SPO3200;product=4'-phosphopantetheinyl transferase	11	17	15	10	7	7
CDS;ID=637290666;locus_tag=SPO3201;product=pyridoxal phosphate biosynthetic proteir	30	18	18	17	25	19
CDS;ID=637290667;locus_tag=SPO3202;product=hypothetical protein	15	12	9	12	14	20
"CDS;ID=637290668;locus_tag=SPO3203;product=guanosine-3',5'-bis(Diphosphate) 3'-pyı	174	193	128	130	199	187
CDS;ID=637290669;locus_tag=SPO3204;product=DNA-directed RNA polymerase omega subuni	791	661	819	1101	590	917
CDS;ID=637290670;locus_tag=SPO3205;product=2-amino-4-hydroxy-6- hydroxymethyldihydı	47	61	46	47	30	56
CDS;ID=637290671;locus_tag=SPO3206;product=hypothetical protein	150	157	115	150	120	169
CDS;ID=637290672;locus_tag=SPO3207;product=4-hydroxy-3-methylbut-2-enyl diphosphate	33	30	20	24	21	26
CDS;ID=637290673;locus_tag=SPO3208;product=glutathione S-transferase family proteir	7	4	7	7	4	4
"CDS;ID=637290674;locus_tag=SPO3209;product=transporter, LysE family"	8	3	5	4	7	10
CDS;ID=637290675;locus_tag=SPO3210;product=hypothetical protein	5	4	5	10	5	10
CDS;ID=637290676;locus_tag=SPO3211;product=hypothetical protein	18	11	10	21	15	24
CDS;ID=637290677;locus_tag=SPO3212;product=ribonuclease HI	8	7	2	5	11	7
CDS;ID=637290678;locus_tag=SPO3213;product=hypothetical protein	5	16	2	7	10	3
CDS;ID=637290679;locus_tag=SPO3214;product=hypothetical protein	10	10	8	12	7	15
CDS;ID=637290680;locus_tag=SPO3215;product=hypothetical protein	12	11	5	8	16	10
CDS;ID=637290681;locus_tag=SPO3216;product=methionyl-tRNA formyltransferase	54	49	30	40	39	46
CDS;ID=637290682;locus_tag=SPO3217;product=peptide deformylase	28	21	18	12	25	10
CDS;ID=637290683;locus_tag=SPO3218;product=peptide deformylase	19	10	11	8	19	20
CDS;ID=637290684;locus_tag=SPO3219;product=peptide deformylase	56	28	20	28	37	35

"CDS;ID=637290685;locus_tag=SPO3220;product=aminotransferase, classes I and II"	63	77	58	38	54	42
CDS;ID=637290686;locus_tag=SPO3221;product=hypothetical protein	16	13	12	8	7	12
CDS;ID=637290687;locus_tag=SPO3222;product=hypothetical protein	21	30	24	15	19	8
CDS;ID=637290688;locus_tag=SPO3223;product=response regulator	11	13	8	9	10	15
CDS;ID=637290689;locus_tag=SPO3224;product=cobalamin biosynthetic protein CobC	16	3	9	7	9	10
CDS;ID=637290690;locus_tag=SPO3225;product=cobalamin biosynthesis protein CobD	5	12	8	12	14	12
CDS;ID=637290691;locus_tag=SPO3226;product=hypothetical protein	28	25	24	23	23	32
CDS;ID=637290692;locus_tag=SPO3227;product=hypothetical protein	2	7	1	1	1	5
CDS;ID=637290693;locus_tag=SPO3228;product=SMC protein	153	86	104	78	81	96
"CDS;ID=637290694;locus_tag=SPO3229;product=lipoprotein, putative"	3	6	2	3	2	0
"CDS;ID=637290695;locus_tag=SPO3230;product=aminotransferase, classes I and II"	69	53	45	36	27	54
CDS;ID=637290696;locus_tag=SPO3231;product=pyruvate dehydrogenase complex represso	24	17	22	9	16	17
CDS;ID=637290697;locus_tag=SPO3232;product=hypothetical protein	68	66	67	35	47	44
"CDS;ID=637290698;locus_tag=SPO3233;product=ATP synthase F0, B subunit"	170	155	152	160	153	153
"CDS;ID=637290699;locus_tag=SPO3234;product=ATP synthase F0, B' subunit"	191	183	178	191	215	172
CDS;ID=637290700;locus_tag=SPO3235;product=ATP synthase subunit C	70	63	46	65	94	57
CDS;ID=637290701;locus_tag=SPO3236;product=ATP synthase subunit A	198	196	200	222	301	192
"CDS;ID=637290702;locus_tag=SPO3237;product=ATP synthase F0, subunit I"	411	308	301	419	355	573
"CDS;ID=637290703;locus_tag=SPO3238;product=transcriptional regulator, ArsR family'	2	0	0	2	3	2
CDS;ID=637290704;locus_tag=SPO3239;product=integral membrane protein	18	24	9	7	16	23
"CDS;ID=637290705;locus_tag=SPO3240;product=transcriptional regulator, LysR family'	53	61	42	31	37	50
CDS;ID=637290706;locus_tag=SPO3241;product=Asp/Glu/Hydantoin racemase family prote	7	8	7	8	3	6
CDS;ID=637290707;locus_tag=SPO3242;product=YbaK/prolyl-tRNA synthetase domain prote	18	29	15	28	17	27
CDS;ID=637290708;locus_tag=SPO3243;product=quinolinate synthetase	37	19	19	28	35	31
CDS;ID=637290709;locus_tag=SPO3244;product=L-aspartate oxidase	7	10	12	7	10	5
CDS;ID=637290710;locus_tag=SPO3245;product=nicotinate-nucleotide pyrophosphorylase	7	5	3	6	6	14
CDS;ID=637290711;locus_tag=SPO3246;product=signal recognition particle protein	98	43	90	157	96	117
"CDS;ID=637290712;locus_tag=SPO3247;product=acetyltransferase, GNAT family"	22	14	15	18	20	25
"CDS;ID=637290713;locus_tag=SPO3248;product=acetyltransferase, GNAT family"	4	6	5	9	11	8
"CDS;ID=637290714;locus_tag=SPO3249;product=acetyltransferase, GNAT family"	10	6	6	9	7	15
"CDS;ID=637290715;locus_tag=SPO3250;product=acetyltransferase, GNAT family"	21	20	17	18	16	20
CDS;ID=637290716;locus_tag=SPO3251;product=hypothetical protein	11	16	30	10	15	16
CDS;ID=637290717;locus_tag=SPO3252;product=chorismate mutase	68	51	86	58	93	55
CDS;ID=637290718;locus_tag=SPO3253;product=ribosomal protein S16	198	180	208	183	244	133
CDS;ID=637290719;locus_tag=SPO3254;product=cobalamin biosynthesis protein BluB	22	18	13	14	21	18
CDS;ID=637290720;locus_tag=SPO3255;product=16S rRNA processing protein RimM	9	10	5	12	6	5
CDS;ID=637290721;locus_tag=SPO3256;product=50S ribosomal protein L31	59	50	42	47	90	55
CDS;ID=637290722;locus_tag=SPO3257;product=50S ribosomal protein L19	178	169	168	185	237	143
"CDS;ID=637290723;locus_tag=SPO3258;product=glycosyl hydrolase, family 25"	12	7	5	5	4	12
CDS;ID=637290724;locus_tag=SPO3259;product=tRNA (guanine-N1)-methyltransferase	23	14	18	20	30	31
"CDS;ID=637290725;locus_tag=SPO3260;product=methyltransferase, homolog"	18	22	15	31	20	15
CDS;ID=637290726;locus_tag=SPO3261;product=glutathione S-transferase family protei	39	31	24	35	32	34
"CDS;ID=637290727;locus_tag=SPO3262;product=transcriptional regulator, MarR family'	21	14	6	15	13	9

CDS;ID=637290728;locus_tag=SPO3263;product=hypothetical protein	13	13	11	7	4	8
tRNA;ID=640698551;locus_tag=SPO_tRNA-Met-3	878	452	275	296	52	299
rRNA;ID=640698552;locus_tag=SPO_Sp5SE;product=5S	8403	6092	3245	3299	1812	3405
tRNA;ID=640698554;locus_tag=SPO_tRNA-Ala-2	2061	850	431	429	82	279
tRNA;ID=640698555;locus_tag=SPO_tRNA-Ile-2	1559	712	389	409	144	384
CDS;ID=637290735;locus_tag=SPO3270;product=hypothetical protein	7	5	3	1	3	4
CDS;ID=637290736;locus_tag=SPO3271;product=hypothetical protein	28	21	15	13	17	18
CDS;ID=637290737;locus_tag=SPO3272;product=glutamate synthase family protein	26	17	16	14	22	32
CDS;ID=637290738;locus_tag=SPO3273;product=hypothetical protein	5	9	9	10	14	6
CDS;ID=637290739;locus_tag=SPO3274;product=hypothetical protein	2	3	6	6	9	6
CDS;ID=637290740;locus_tag=SPO3275;product=hypothetical protein	6	11	4	5	7	5
"CDS;ID=637290741;locus_tag=SPO3276;product=ATP-dependent Clp protease, ATP-binding	134	94	150	177	199	179
CDS;ID=637290742;locus_tag=SPO3277;product=hypothetical protein	32	12	8	15	14	17
CDS;ID=637290743;locus_tag=SPO3278;product=orotidine 5'-phosphate decarboxylase	21	35	29	43	43	32
CDS;ID=637290744;locus_tag=SPO3279;product=NUDIX domain protein	65	63	65	49	57	50
CDS;ID=637290745;locus_tag=SPO3280;product=DNA polymerase IV	11	13	7	6	12	6
CDS;ID=637290746;locus_tag=SPO3281;product=DinB family protein	6	4	5	2	10	9
CDS;ID=637290747;locus_tag=SPO3282;product=N-formylglutamate amidohydrolase family	42	40	36	36	50	42
tRNA;ID=640698557;locus_tag=SPO_tRNA-Val-4	467	286	174	160	231	140
CDS;ID=637290749;locus_tag=SPO3284;product=ribosomal protein L36	62	59	31	72	32	111
CDS;ID=637290750;locus_tag=SPO3285;product=hypothetical protein	18	23	8	17	16	15
CDS;ID=637290751;locus_tag=SPO3286;product=arylsulfatase	5	7	4	7	3	8
"CDS;ID=637290752;locus_tag=SPO3287;product=ferric iron ABC transporter, periplasmi	46	44	54	124	134	116
"CDS;ID=637290753;locus_tag=SPO3288;product=ferric iron ABC transporter, permease p	28	18	26	46	37	35
"CDS;ID=637290754;locus_tag=SPO3289;product=phenylacetate-CoA ligase, putative"	13	7	6	6	7	11
"CDS;ID=637290755;locus_tag=SPO3290;product=branched-chain amino acid ABC transport	6	6	6	2	5	3
"CDS;ID=637290756;locus_tag=SPO3291;product=branched-chain amino acid ABC transport	11	3	10	18	7	14
"CDS;ID=637290757;locus_tag=SPO3292;product=branched-chain amino acid ABC transport	7	0	7	5	11	7
CDS;ID=637290758;locus_tag=SPO3293;product=hypothetical protein	6	7	4	5	6	1
"CDS;ID=637290759;locus_tag=SPO3294;product=branched-chain amino acid ABC transport	13	8	8	14	12	11
"CDS;ID=637290760;locus_tag=SPO3295;product=branched-chain amino acid ABC transport	21	15	16	5	16	14
CDS;ID=637290761;locus_tag=SPO3296;product=AMP-binding enzyme	58	40	37	30	41	30
CDS;ID=637290762;locus_tag=SPO3297;product=sensory box histidine kinase/response re	47	35	29	38	39	35
CDS;ID=637290763;locus_tag=SPO3298;product=DNA-binding response regulator	2	6	1	2	0	2
"CDS;ID=637290764;locus_tag=SPO3299;product=transcriptional regulator, TetR family"	2	4	2	5	0	2
CDS;ID=637290765;locus_tag=SPO3300;product=hypothetical protein	5	1	2	4	6	5
"CDS;ID=637290766;locus_tag=SPO3301;product=transcriptional regulator, LysR family"	23	15	17	19	23	28
CDS;ID=637290767;locus_tag=SPO3302;product=hypothetical protein	2	2	2	6	5	2
"CDS;ID=637290768;locus_tag=SPO3303;product=GDSL-like lipase/acylhydrolase, putativ	27	29	30	28	25	33
CDS;ID=637290769;locus_tag=SPO3304;product=N-carbamoyl-L-amino acid amidohydrolase	35	18	26	19	29	19
CDS;ID=637290770;locus_tag=SPO3305;product=phosphoglycerate mutase family protein	61	53	34	50	78	50
"CDS;ID=637290771;locus_tag=SPO3306;product=glutathione S-transferase, putative"	34	33	20	19	24	37
"CDS;ID=637290772;locus_tag=SPO3307;product=saccharopine dehydrogenase, putative"	37	47	29	40	46	43

CDS;ID=637290773;locus_tag=SPO3308;product=hypothetical protein	11	5	6	15	23	14
"CDS;ID=637290774;locus_tag=SPO3309;product=saccharopine dehydrogenase, putative"	49	34	34	36	50	37
CDS;ID=637290775;locus_tag=SPO3310;product=hypothetical protein	75	64	59	102	88	106
"CDS;ID=637290776;locus_tag=SPO3311;product=ADA regulatory protein, putative"	3	5	6	2	9	7
CDS;ID=637290777;locus_tag=SPO3312;product=hypothetical protein	3	1	1	0	2	2
"CDS;ID=637290778;locus_tag=SPO3313;product=DNA-binding protein, putative"	11	2	3	6	2	3
CDS;ID=637290779;locus_tag=SPO3314;product=dihydroxy-acid dehydratase	26	18	20	21	14	18
"CDS;ID=637290780;locus_tag=SPO3315;product=transcriptional regulator, LysR family'	8	8	5	2	5	5
CDS;ID=637290781;locus_tag=SPO3316;product=membrane protein	1	3	0	1	5	2
"CDS;ID=637290782;locus_tag=SPO3317;product=transcriptional regulator, putative"	13	6	8	6	6	8
CDS;ID=637290783;locus_tag=SPO3318;product=hypothetical protein	122	117	100	91	83	102
CDS;ID=637290784;locus_tag=SPO3319;product=epsK domain protein	125	86	63	96	88	79
CDS;ID=637290785;locus_tag=SPO3320;product=hypothetical protein	57	67	63	39	48	74
CDS;ID=637290786;locus_tag=SPO3321;product=hypothetical protein	30	21	22	23	24	25
CDS;ID=637290787;locus_tag=SPO3322;product=hypothetical protein	88	52	54	53	60	69
CDS;ID=637290788;locus_tag=SPO3323;product=hypothetical protein	41	43	51	48	66	47
CDS;ID=637290789;locus_tag=SPO3324;product=hypothetical protein	53	65	41	39	57	74
CDS;ID=637290790;locus_tag=SPO3325;product=hypothetical protein	292	301	275	271	326	314
CDS;ID=637290791;locus_tag=SPO3326;product=hypothetical protein	60	46	51	56	51	70
CDS;ID=637290792;locus_tag=SPO3327;product=creatinase	9	19	11	13	14	13
CDS;ID=637290793;locus_tag=SPO3328;product=succinate-semialdehyde dehydrogenase	37	28	33	19	19	27
CDS;ID=637290794;locus_tag=SPO3329;product=globin domain protein	4	7	6	6	6	7
CDS;ID=637290795;locus_tag=SPO3330;product=ribonuclease R	214	172	126	186	182	208
CDS;ID=637290796;locus_tag=SPO3331;product=hypothetical protein	2	0	3	9	1	8
CDS;ID=637290797;locus_tag=SPO3332;product=succinyl-diaminopimelate desuccinylase	17	28	22	23	31	22
CDS;ID=637290798;locus_tag=SPO3333;product=hypothetical protein	65	61	58	61	92	96
CDS;ID=637290799;locus_tag=SPO3334;product=hypothetical protein	9	13	9	13	15	24
"CDS;ID=637290800;locus_tag=SPO3335;product=glutamine ABC transporter, periplasmic	16	11	4	12	10	10
CDS;ID=637290801;locus_tag=SPO3336;product=hypothetical protein	18	13	15	11	12	5
"CDS;ID=637290802;locus_tag=SPO3337;product=2,3,4,5-tetrahydropyridine-2,6-dicarbo)	46	32	43	22	33	47
CDS;ID=637290803;locus_tag=SPO3338;product=biotin synthase	13	11	4	21	33	9
CDS;ID=637290804;locus_tag=SPO3339;product=bioY family protein	2	4	0	9	7	16
"CDS;ID=637290805;locus_tag=SPO3340;product=transcriptional regulator, GntR family'	4	8	8	5	4	4
CDS;ID=637290806;locus_tag=SPO3341;product=threonine dehydratase	30	22	27	12	20	19
CDS;ID=637290807;locus_tag=SPO3342;product=decarboxylase family protein	85	73	80	79	112	83
CDS;ID=637290808;locus_tag=SPO3343;product=hypothetical protein	23	21	26	20	25	19
CDS;ID=637290809;locus_tag=SPO3344;product=hypothetical protein	19	16	19	18	22	16
CDS;ID=637290810;locus_tag=SPO3345;product=hypothetical protein	6	12	2	7	8	2
"CDS;ID=637290811;locus_tag=SPO3346;product=radical SAM enzyme, Cfr family"	50	37	43	48	50	37
CDS;ID=637290812;locus_tag=SPO3347;product=hypothetical protein	20	18	18	11	22	14
CDS;ID=637290813;locus_tag=SPO3348;product=hypothetical protein	209	123	148	141	160	143
"CDS;ID=637290814;locus_tag=SPO3349;product=L-asparaginase, thermolabile family"	30	19	12	25	27	29
CDS;ID=637290815;locus_tag=SPO3350;product=hypothetical protein	1	0	1	3	2	1

CDS;ID=637290816;locus_tag=SPO3351;product=Aha1 domain protein	11	13	1	17	17	9
"CDS;ID=637290817;locus_tag=SPO3352;product=transcriptional regulator, ArsR family'	5	9	3	11	13	10
CDS;ID=637290818;locus_tag=SPO3353;product=phosphoserine phosphatase	18	10	13	18	12	20
RNA;ID=641222527;locus_tag=SPO_R0067;product=serC	15	11	17	41	22	31
CDS;ID=637290819;locus_tag=SPO3354;product=phosphoserine aminotransferase	67	58	51	109	116	80
CDS;ID=637290820;locus_tag=SPO3355;product=D-3-phosphoglycerate dehydrogenase	44	38	41	60	84	55
CDS;ID=637290821;locus_tag=SPO3356;product=hypothetical protein	1	1	1	1	2	0
"CDS;ID=637290822;locus_tag=SPO3357;product=transcriptional regulator, LysR family'	10	6	3	7	5	9
CDS;ID=637290823;locus_tag=SPO3358;product=serine/threonine protein phosphatase far	16	9	2	13	7	12
CDS;ID=637290824;locus_tag=SPO3359;product=L-threonine 3-dehydrogenase	20	22	19	15	27	9
CDS;ID=637290825;locus_tag=SPO3360;product=2-amino-3-ketobutyrate coenzyme A ligase	13	10	4	11	16	19
CDS;ID=637290826;locus_tag=SPO3361;product=RmuC domain protein	97	103	74	87	72	91
CDS;ID=637290827;locus_tag=SPO3362;product=DNA mismatch repair protein	94	88	85	89	55	97
"CDS;ID=637290828;locus_tag=SPO3363;product=zinc/manganese/iron ABC transporter, pe	17	8	9	9	16	31
"CDS;ID=637290829;locus_tag=SPO3364;product=zinc/manganese/iron ABC transporter, pe	46	26	21	35	46	38
"CDS;ID=637290830;locus_tag=SPO3365;product=zinc/manganese/iron ABC transporter, AT	14	11	10	34	13	38
"CDS;ID=637290831;locus_tag=SPO3366;product=zinc/manganese/iron ABC transporter, pe	56	31	46	47	51	86
CDS;ID=637290832;locus_tag=SPO3367;product=deoxyribose-phosphate aldolase	23	25	27	13	27	13
CDS;ID=637290833;locus_tag=SPO3368;product=aldehyde dehydrogenase family protein	89	66	64	34	51	64
CDS;ID=637290834;locus_tag=SPO3369;product=short chain dehydrogenase	11	3	3	8	9	14
"CDS;ID=637290835;locus_tag=SPO3370;product=peptidase, M16 family"	66	55	47	56	65	66
"CDS;ID=637290836;locus_tag=SPO3371;product=peptidase, M16 family"	134	88	89	107	110	117
CDS;ID=637290837;locus_tag=SPO3372;product=hypothetical protein	49	27	17	30	29	55
CDS;ID=637290838;locus_tag=SPO3373;product=lipoprotein signal peptidase	20	15	1	20	18	23
CDS;ID=637290839;locus_tag=SPO3374;product=bifunctional phosphoribosylaminoimidazo	72	60	52	57	67	70
CDS;ID=637290840;locus_tag=SPO3375;product=hypothetical protein	58	29	40	33	50	41
"CDS;ID=637290841;locus_tag=SPO3376;product=ribosomal RNA small subunit methyltrans	6	12	11	14	16	23
CDS;ID=637290842;locus_tag=SPO3377;product=hypothetical protein	8	9	11	11	10	8
CDS;ID=637290843;locus_tag=SPO3378;product=benzoate transporter	8	8	14	5	5	14
"CDS;ID=637290844;locus_tag=SPO3379;product=transcriptional regulator, LysR family'	1	4	2	3	2	6
"CDS;ID=637290845;locus_tag=SPO3380;product=gamma-butyrobetaine hydroxylase, putat:	0	2	3	0	0	2
CDS;ID=637290846;locus_tag=SPO3381;product=hypothetical protein	1	5	2	1	7	4
CDS;ID=637290847;locus_tag=SPO3382;product=aldehyde dehydrogenase family protein	134	123	91	116	129	112
CDS;ID=637290848;locus_tag=SPO3383;product=thiol-specific antioxidant protein	71	89	90	89	82	89
"CDS;ID=637290849;locus_tag=SPO3384;product=methyltransferase, FkbM family"	8	6	4	5	7	4
"CDS;ID=637290850;locus_tag=SPO3385;product=glycosyl transferase, family 25"	10	13	16	13	17	27
CDS;ID=637290851;locus_tag=SPO3386;product=hypothetical protein	2	6	6	6	2	6
CDS;ID=637290852;locus_tag=SPO3387;product=hypothetical protein	33	33	24	22	15	28
"CDS;ID=637290853;locus_tag=SPO3388;product=transcriptional regulator, TetR family'	2	5	3	3	2	3
CDS;ID=637290854;locus_tag=SPO3389;product=polyribonucleotide nucleotidyltransferas	464	425	364	432	535	399
CDS;ID=637290855;locus_tag=SPO3390;product=hypothetical protein	21	15	20	22	24	16
"CDS;ID=637290856;locus_tag=SPO3391;product=alpha-1,2-fucosyltransferase, putative'	26	24	27	27	19	19
CDS;ID=637290857;locus_tag=SPO3392;product=ribosomal large subunit pseudouridine sy	31	23	9	28	30	33

CDS;ID=637290858;locus_tag=SPO3393;product=hypothetical protein	4	1	2	6	7	2
"CDS;ID=637290859;locus_tag=SPO3394;product=GDSL-like lipase/acylhydrolase, putativ	4	2	0	2	1	2
CDS;ID=637290860;locus_tag=SPO3395;product=RarD	27	17	17	15	21	15
CDS;ID=637290861;locus_tag=SPO3396;product=FAD dependent oxidoreductase/aminomethy	17	24	9	15	8	13
"CDS;ID=637290862;locus_tag=SPO3397;product=transcriptional regulator, AraC family"	2	2	0	2	5	5
CDS;ID=637290863;locus_tag=SPO3398;product=homocysteine S-methyltransferase family	2	7	2	6	2	3
"CDS;ID=637290864;locus_tag=SPO3399;product=alcohol dehydrogenase, zinc-containing"	1	7	5	2	8	4
CDS;ID=637290865;locus_tag=SPO3400;product=aminomethyl transferase family protein	6	6	8	12	9	11
CDS;ID=637290866;locus_tag=SPO3401;product=hypothetical protein	1	0	1	1	0	0
"CDS;ID=637290867;locus_tag=SPO3402;product=transporter, LysE family"	1	1	0	0	0	1
"CDS;ID=637290868;locus_tag=SPO3403;product=DNA-binding protein, putative"	0	0	2	1	1	1
CDS;ID=637290869;locus_tag=SPO3404;product=hypothetical protein	25	12	10	20	18	18
CDS;ID=637290870;locus_tag=SPO3405;product=hypothetical protein	20	3	6	13	13	11
"CDS;ID=637290871;locus_tag=SPO3406;product=transcriptional regulator, TetR family"	4	0	3	3	4	1
CDS;ID=637290872;locus_tag=SPO3407;product=hypothetical protein	13	9	3	2	5	5
CDS;ID=637290873;locus_tag=SPO3408;product=acetyl-CoA C-acetyltransferase	25	26	6	29	33	35
CDS;ID=637290874;locus_tag=SPO3409;product=anti-anti-sigma factor	0	2	0	1	1	0
"CDS;ID=637290875;locus_tag=SPO3410;product=anti-sigma B factor, putative"	0	0	0	1	0	0
"CDS;ID=637290876;locus_tag=SPO3411;product=gamma-glutamyltranspeptidase, putative"	24	14	8	19	12	15
CDS;ID=637290877;locus_tag=SPO3412;product=hypothetical protein	29	49	41	55	49	41
CDS;ID=637290878;locus_tag=SPO3413;product=auxin efflux carrier family protein	13	12	8	10	6	7
"CDS;ID=637290879;locus_tag=SPO3414;product=lipoprotein, putative"	146	164	126	207	148	162
"CDS;ID=637290880;locus_tag=SPO3415;product=outer membrane lipoprotein carrier prot	92	71	64	76	57	78
CDS;ID=637290881;locus_tag=SPO3416;product=FtsK/SpoIIIE family protein	170	188	133	191	228	186
"CDS;ID=637290882;locus_tag=SPO3417;product=aminotransferase, classes I and II"	119	100	79	109	143	130
CDS;ID=637290883;locus_tag=SPO3418;product=amidase family protein	38	40	24	34	12	25
"CDS;ID=637290884;locus_tag=SPO3419;product=ubiquinone biosynthesis hydroxylase, Ub	27	24	16	28	25	33
CDS;ID=637290885;locus_tag=SPO3420;product=hypothetical protein	20	23	9	12	12	10
CDS;ID=637290886;locus_tag=SPO3421;product=hypothetical protein	9	6	2	4	11	12
CDS;ID=637290887;locus_tag=SPO3422;product=ATP-dependent protease La domain protein	10	9	14	18	22	20
CDS;ID=637290888;locus_tag=SPO3423;product=thioredoxin	54	53	28	43	57	68
CDS;ID=637290889;locus_tag=SPO3424;product=leucine rich repeat protein	29	32	20	24	37	33
"CDS;ID=637290890;locus_tag=SPO3425;product=exodeoxyribonuclease III, putative"	53	55	37	38	49	58
CDS;ID=637290891;locus_tag=SPO3426;product=DNA-binding response regulator	34	15	17	24	18	20
"CDS;ID=637290892;locus_tag=SPO3427;product=3,4-dihydroxy-2-butanone 4-phosphate sy	21	19	16	32	36	31
CDS;ID=637290893;locus_tag=SPO3428;product=hypothetical protein	8	7	7	5	9	8
CDS;ID=637290894;locus_tag=SPO3429;product=alanine racemase domain protein	36	39	39	44	30	43
CDS;ID=637290895;locus_tag=SPO3430;product=outer membrane porin	1370	1189	1337	1282	1385	1175
CDS;ID=637290896;locus_tag=SPO3431;product=hypothetical protein	77	64	61	82	63	78
CDS;ID=637290897;locus_tag=SPO3432;product=leucyl-tRNA synthetase	107	111	95	93	107	111
"CDS;ID=637290898;locus_tag=SPO3433;product=lipoprotein, putative"	11	24	17	15	20	11
CDS;ID=637290899;locus_tag=SPO3434;product=hypothetical protein	14	10	15	21	12	17
CDS;ID=637290900;locus_tag=SPO3435;product=glutathione S-transferase family protein	16	16	9	33	23	18

CDS;ID=637290901;locus_tag=SPO3436;product=hypothetical protein	13	5	7	10	9	14
CDS;ID=637290902;locus_tag=SPO3437;product=mechanosensitive ion channel family prot	13	7	5	13	15	6
CDS;ID=637290903;locus_tag=SPO3438;product=hypothetical protein	4	3	3	0	9	6
CDS;ID=637290904;locus_tag=SPO3439;product=enoyl-CoA hydratase/isomerase family pro	6	4	3	1	5	6
"CDS;ID=637290905;locus_tag=SPO3440;product=20-beta-hydroxysteroid dehydrogenase, p	16	12	17	16	18	21
CDS;ID=637290906;locus_tag=SPO3441;product=hypothetical protein	159	109	119	116	167	131
CDS;ID=637290907;locus_tag=SPO3442;product=PmbA	47	30	47	35	47	41
CDS;ID=637290908;locus_tag=SPO3443;product=inositol monophosphatase family protein	14	8	15	17	16	19
"CDS;ID=637290909;locus_tag=SPO3444;product=3-deoxy-D-manno-octulosonic-acid transf	13	17	9	14	17	16
CDS;ID=637290910;locus_tag=SPO3445;product=tetraacyldisaccharide 4'-kinase	46	27	35	27	20	27
"CDS;ID=637290911;locus_tag=SPO3446;product=thiol:disulfide interchange protein, Ds	175	129	138	175	138	203
CDS;ID=637290912;locus_tag=SPO3447;product=hypothetical protein	28	25	44	50	29	38
CDS;ID=637290913;locus_tag=SPO3448;product=A/G-specific adenine glycosylase	9	10	4	8	7	5
CDS;ID=637290914;locus_tag=SPO3449;product=fatty acid desaturase family protein	34	30	31	39	30	41
CDS;ID=637290915;locus_tag=SPO3450;product=adenylate/guanylate cyclase	25	32	20	22	21	33
CDS;ID=637290916;locus_tag=SPO3451;product=modification methylase	448	430	375	389	451	437
CDS;ID=637290917;locus_tag=SPO3452;product=ribonuclease HII	7	10	8	23	13	8
CDS;ID=637290918;locus_tag=SPO3453;product=hypothetical protein	22	20	18	26	40	26
CDS;ID=637290919;locus_tag=SPO3454;product=hypothetical protein	12	16	16	13	16	27
CDS;ID=637290920;locus_tag=SPO3455;product=adenylate/guanylate cyclase	71	70	38	53	57	68
"CDS;ID=637290921;locus_tag=SPO3456;product=flagellar protein, putative"	7	7	3	4	7	4
"CDS;ID=637290922;locus_tag=SPO3457;product=flagellin synthesis repressor protein F	0	0	0	2	1	1
"CDS;ID=637290923;locus_tag=SPO3458;product=flagellar protein FlaF, putative"	0	1	0	1	2	1
CDS;ID=637290924;locus_tag=SPO3459;product=flagellin protein	6	3	1	5	5	5
CDS;ID=637290925;locus_tag=SPO3460;product=hypothetical protein	1	0	1	1	0	1
"CDS;ID=637290926;locus_tag=SPO3461;product=flagellar protein FlgJ, putative"	0	0	0	0	0	1
CDS;ID=637290927;locus_tag=SPO3462;product=flagellar hook-length control protein	11	3	4	10	15	7
CDS;ID=637290928;locus_tag=SPO3463;product=basal-body rod modification protein FlgD	37	39	36	23	18	30
CDS;ID=637290929;locus_tag=SPO3464;product=hypothetical protein	11	7	10	3	4	12
CDS;ID=637290930;locus_tag=SPO3465;product=hypothetical protein	5	8	7	6	7	7
"CDS;ID=637290931;locus_tag=SPO3466;product=putrescine ABC transporter, permease pr	10	10	8	8	9	8
"CDS;ID=637290932;locus_tag=SPO3467;product=putrescine ABC transporter, permease pr	18	17	10	8	14	8
"CDS;ID=637290933;locus_tag=SPO3468;product=putrescine ABC transporter, ATP-binding	14	18	7	19	10	14
"CDS;ID=637290934;locus_tag=SPO3469;product=putrescine ABC transporter, periplasmic	68	74	59	46	52	50
"CDS;ID=637290935;locus_tag=SPO3470;product=transcriptional regulator, GntR family"	14	9	9	9	14	23
CDS;ID=637290936;locus_tag=SPO3471;product=hypothetical protein	36	32	42	38	36	17
"CDS;ID=637290937;locus_tag=SPO3472;product=polyamine ABC transporter, ATP-binding	214	224	138	171	197	186
"CDS;ID=637290938;locus_tag=SPO3473;product=polyamine ABC trasnporter, periplasmic	161	111	163	110	217	113
"CDS;ID=637290939;locus_tag=SPO3474;product=polyamine ABC transporter, permease pro	41	48	55	58	77	41
"CDS;ID=637290940;locus_tag=SPO3475;product=polyamine ABC transporter, permease pro	280	334	264	176	142	250
CDS;ID=637290941;locus_tag=SPO3476;product=hypothetical protein	1	2	2	1	2	1
CDS;ID=637290942;locus_tag=SPO3477;product=hypothetical protein	17	17	17	13	22	29
"CDS;ID=637290943;locus_tag=SPO3478;product=glycolate oxidase, GlcD subunit"	19	13	21	17	12	15

"CDS;ID=637290944;locus_tag=SPO3479;product=glycolate oxidase, GlcE subunit"	14	4	12	4	4	3
"CDS;ID=637290945;locus_tag=SPO3480;product=glycolate oxidase, iron-sulfur subunit'	12	13	15	5	8	8
CDS;ID=637290946;locus_tag=SPO3481;product=hypothetical protein	21	16	14	11	9	18
CDS;ID=637290947;locus_tag=SPO3482;product=trypsin domain protein	21	12	27	16	20	19
CDS;ID=637290948;locus_tag=SPO3483;product=hypothetical protein	0	2	2	1	1	1
"CDS;ID=637290949;locus_tag=SPO3484;product=heat shock protein, Hsp20 family"	31	23	39	35	59	47
CDS;ID=637290950;locus_tag=SPO3485;product=hypothetical protein	11	15	12	7	2	10
CDS;ID=637290951;locus_tag=SPO3486;product=hypothetical protein	6	12	5	6	6	5
CDS;ID=637290952;locus_tag=SPO3487;product=hypothetical protein	2	5	1	3	3	6
CDS;ID=637290953;locus_tag=SPO3488;product=peptidoglycan binding domain protein	86	69	48	58	64	57
CDS;ID=637290954;locus_tag=SPO3489;product=phenylalanyl-tRNA synthetase beta subun:	118	89	106	101	125	97
"CDS;ID=637290955;locus_tag=SPO3490;product=transcriptional regulator, LysR family'	1	6	3	7	6	3
"CDS;ID=637290956;locus_tag=SPO3491;product=methyltransferase, UbiE/COQ5 family"	3	5	2	3	3	0
CDS;ID=637290957;locus_tag=SPO3492;product=hypothetical protein	22	17	26	22	13	10
"CDS;ID=637290958;locus_tag=SPO3493;product=transporter, putative"	20	10	6	6	12	11
CDS;ID=637290959;locus_tag=SPO3494;product=glutathione S-transferase family proteir	36	55	29	19	34	35
CDS;ID=637290960;locus_tag=SPO3495;product=large conductance mechanosensitive chanr	10	8	10	8	17	6
CDS;ID=637290961;locus_tag=SPO3496;product=mechanosensitive ion channel family prot	10	13	8	4	17	12
tRNA;ID=640698558;locus_tag=SPO_tRNA-Met-4	352	166	97	111	25	87
CDS;ID=637290963;locus_tag=SPO3498;product=translation elongation factor Tu	968	984	924	818	1387	840
CDS;ID=637290964;locus_tag=SPO3499;product=translation elongation factor G	616	549	561	533	660	458
CDS;ID=637290965;locus_tag=SPO3500;product=30S ribosomal protein S7	207	222	216	230	263	148
CDS;ID=637290966;locus_tag=SPO3501;product=30S ribosomal protein S12	283	322	299	314	531	246
CDS;ID=637290967;locus_tag=SPO3502;product=hypothetical protein	86	74	61	99	72	87
CDS;ID=637290968;locus_tag=SPO3503;product=hypothetical protein	6	7	5	9	2	6
CDS;ID=637290969;locus_tag=SPO3504;product=hypothetical protein	27	26	16	21	14	32
CDS;ID=637290970;locus_tag=SPO3505;product=hypothetical protein	47	45	28	42	46	65
"CDS;ID=637290971;locus_tag=SPO3506;product=DNA-binding protein, putative"	3	3	1	5	3	1
CDS;ID=637290972;locus_tag=SPO3507;product=DNA-directed RNA polymerase beta' subun:	630	563	490	506	684	567
CDS;ID=637290973;locus_tag=SPO3508;product=DNA-directed RNA polymerase beta subunit	545	529	471	466	619	532
CDS;ID=637290974;locus_tag=SPO3509;product=ribosomal protein L7/L12	1612	1452	1523	1415	1528	1082
CDS;ID=637290975;locus_tag=SPO3510;product=ribosomal protein L10	1801	1623	1787	1965	2327	1961
CDS;ID=637290976;locus_tag=SPO3511;product=hypothetical protein	8	11	11	9	22	11
CDS;ID=637290977;locus_tag=SPO3512;product=hypothetical protein	34	17	17	17	18	28
CDS;ID=637290978;locus_tag=SPO3513;product=50S ribosomal protein L1	167	175	145	152	177	165
CDS;ID=637290979;locus_tag=SPO3514;product=ribosomal protein L11	184	180	179	234	369	238
CDS;ID=637290980;locus_tag=SPO3515;product=hypothetical protein	10	3	3	11	8	5
CDS;ID=637290981;locus_tag=SPO3516;product=transcription termination/antiterminatic	43	53	44	83	49	73
"CDS;ID=637290982;locus_tag=SPO3517;product=preprotein translocase, SecE subunit"	31	35	25	22	43	25
CDS;ID=637290983;locus_tag=SPO3518;product=hypothetical protein	23	10	16	10	19	25
"CDS;ID=637290984;locus_tag=SPO3519;product=cytochrome oxidase maturation protein,	14	11	7	5	3	8
CDS;ID=637290985;locus_tag=SPO3520;product=copper-translocating P-type ATPase	117	124	103	104	125	120
"CDS;ID=637290986;locus_tag=SPO3521;product=fixH protein, putative"	35	66	32	54	56	39

"CDS;ID=637290987;locus_tag=SPO3522;product=iron-sulfur-binding protein, RdxA/RdxB/	551	364	485	593	617	519
"CDS;ID=637290988;locus_tag=SPO3523;product=cytochrome c oxidase, cbb3-type, subuni	78	88	88	91	169	121
"CDS;ID=637290989;locus_tag=SPO3524;product=cytochrome c oxidase, cbb3-type, subuni	36	27	51	34	80	67
"CDS;ID=637290990;locus_tag=SPO3525;product=cytochrome c oxidase, cbb3-type, subuni	109	111	112	89	186	117
"CDS;ID=637290991;locus_tag=SPO3526;product=cytochrome c oxidase, cbb3-type, subuni	219	195	207	235	381	288
CDS;ID=637290992;locus_tag=SPO3527;product=universal stress protein family protein	185	181	205	239	244	217
CDS;ID=637290993;locus_tag=SPO3528;product=hypothetical protein	16	20	12	17	18	23
CDS;ID=637290994;locus_tag=SPO3529;product=serine hydroxymethyltransferase	4	11	2	7	10	8
"CDS;ID=637290995;locus_tag=SPO3530;product=transcriptional regulator, LysR family"	8	4	2	6	7	10
CDS;ID=637290996;locus_tag=SPO3531;product=transcriptional activator protein FnrL	129	133	97	131	144	125
CDS;ID=637290997;locus_tag=SPO3532;product=coproporphyrinogen III oxidase	174	114	143	179	189	161
"CDS;ID=637290998;locus_tag=SPO3533;product=translation elongation factor G, putati	47	44	33	31	51	28
"CDS;ID=637290999;locus_tag=SPO3534;product=oligopeptide/dipeptide uptake family AE	50	49	30	52	45	53
"CDS;ID=637291000;locus_tag=SPO3535;product=oligopeptide/dipeptide uptake family AE	16	22	19	18	18	15
"CDS;ID=637291001;locus_tag=SPO3536;product=oligopeptide/dipeptide uptake family AE	57	46	58	44	60	80
"CDS;ID=637291002;locus_tag=SPO3537;product=oligopeptide/dipeptide uptake family AE	88	108	67	91	90	120
CDS;ID=637291003;locus_tag=SPO3538;product=cytochrome c552	347	365	380	527	527	429
CDS;ID=637291004;locus_tag=SPO3539;product=prephenate dehydratase	19	14	18	14	23	22
CDS;ID=637291005;locus_tag=SPO3540;product=hypothetical protein	12	6	5	14	16	8
"CDS;ID=637291006;locus_tag=SPO3541;product=hydrolase, NUDIX family"	12	15	16	14	13	18
"CDS;ID=637291007;locus_tag=SPO3542;product=2`,3`-cyclic-nucleotide 2`-phosphodiest	9	13	13	20	13	13
RNA;ID=641222528;locus_tag=SPO_R0068;product=SRP_bact	51	20	16	17	9	28
CDS;ID=637291008;locus_tag=SPO3543;product=hypothetical protein	6	4	7	8	7	3
CDS;ID=637291009;locus_tag=SPO3544;product=hypothetical protein	5	8	2	2	8	7
"CDS;ID=637291010;locus_tag=SPO3545;product=transcriptional regulator, TetR family"	7	3	2	2	4	8
"CDS;ID=637291011;locus_tag=SPO3546;product=zinc metallopeptidase, putative"	52	25	15	33	35	38
RNA;ID=641222529;locus_tag=SPO_R0069;product=Cobalamin	60	60	70	68	62	106
CDS;ID=637291012;locus_tag=SPO3547;product=hypothetical protein	25	18	25	20	40	19
CDS;ID=637291013;locus_tag=SPO3548;product=hypothetical protein	13	27	22	19	29	28
CDS;ID=637291014;locus_tag=SPO3549;product=hypothetical protein	16	9	10	3	6	7
CDS;ID=637291015;locus_tag=SPO3550;product=DNA polymerase III subunits gamma and ta	74	86	61	97	82	77
"CDS;ID=637291016;locus_tag=SPO3551;product=acetyltransferase, GNAT family"	3	3	0	8	4	8
"CDS;ID=637291017;locus_tag=SPO3552;product=oxidoreductase, FAD-binding"	15	10	6	9	12	8
CDS;ID=637291018;locus_tag=SPO3553;product=hypothetical protein	18	17	18	19	11	12
CDS;ID=637291019;locus_tag=SPO3554;product=hypothetical protein	25	19	29	19	22	22
"CDS;ID=637291020;locus_tag=SPO3555;product=transglycosylase, Slt family"	157	168	135	122	106	130
CDS;ID=637291021;locus_tag=SPO3556;product=dihydrodipicolinate synthase	45	37	40	70	51	40
"CDS;ID=637291022;locus_tag=SPO3557;product=dimethyl sulfoxide reductase, C subunit	20	21	16	12	13	20
CDS;ID=637291023;locus_tag=SPO3558;product=iron-sulfur cluster-binding protein	15	16	9	8	8	14
"CDS;ID=637291024;locus_tag=SPO3559;product=oxidoreductase, molybdopterin-binding"	12	11	9	7	18	12
CDS;ID=637291025;locus_tag=SPO3560;product=phosphate acetyltransferase	8	3	0	1	2	7
CDS;ID=637291026;locus_tag=SPO3561;product=hypothetical protein	10	8	10	9	3	5
CDS;ID=637291027;locus_tag=SPO3562;product=transcriptional regulator	6	5	6	8	2	6

"CDS;ID=637291028;locus_tag=SPO3563;product=transcriptional regulator, ArsR family'	5	3	7	3	5	8
"CDS;ID=637291029;locus_tag=SPO3564;product=permease, putative"	4	1	0	3	3	4
CDS;ID=637291030;locus_tag=SPO3565;product=hypothetical protein	23	14	12	24	26	20
CDS;ID=637291031;locus_tag=SPO3566;product=hypothetical protein	132	127	126	153	193	157
CDS;ID=637291032;locus_tag=SPO3567;product=ribonuclease T2 family protein	6	9	11	4	4	6
CDS;ID=637291033;locus_tag=SPO3568;product=hypothetical protein	16	18	16	19	19	17
CDS;ID=637291034;locus_tag=SPO3569;product=recombination protein RecR	18	12	16	4	10	23
CDS;ID=637291035;locus_tag=SPO3570;product=conserved hypothetical protein TIGR0010:	227	168	171	228	208	245
"CDS;ID=637291036;locus_tag=SPO3571;product=transcriptional regulator, AraC family'	19	12	12	14	20	14
CDS;ID=637291037;locus_tag=SPO3572;product=isochorismatase family protein	0	2	1	0	3	1
CDS;ID=637291038;locus_tag=SPO3573;product=NADPH-dependent FMN reductase domain pro	16	10	14	14	19	14
CDS;ID=637291039;locus_tag=SPO3574;product=short chain dehydrogenase	2	4	0	3	6	3
"CDS;ID=637291040;locus_tag=SPO3575;product=transcriptional regulator, MerR family'	4	3	2	4	7	1
CDS;ID=637291041;locus_tag=SPO3576;product=OmpA domain protein	336	295	241	277	351	317
CDS;ID=637291042;locus_tag=SPO3577;product=hypothetical protein	3	4	6	0	1	2
CDS;ID=637291043;locus_tag=SPO3578;product=ADA regulatory protein	9	17	12	14	5	12
"CDS;ID=637291044;locus_tag=SPO3579;product=transcriptional regulator, MarR family'	8	1	0	4	5	4
CDS;ID=637291045;locus_tag=SPO3580;product=isochorismatase family protein	5	3	4	5	5	8
CDS;ID=637291046;locus_tag=SPO3581;product=endonuclease III	107	85	68	72	97	93
"CDS;ID=637291047;locus_tag=SPO3582;product=kinase, pfkB family"	83	78	47	59	60	75
CDS;ID=637291048;locus_tag=SPO3583;product=transcriptional regulator PecS	42	28	15	31	30	37
"CDS;ID=637291049;locus_tag=SPO3584;product=membrane protein, drug/metabolite trans	14	21	14	17	22	17
"CDS;ID=637291050;locus_tag=SPO3585;product=hydrolase, NUDIX family, NudH subfamily	25	28	21	31	32	41
"CDS;ID=637291051;locus_tag=SPO3586;product=2,4-dienoyl-CoA reductase"	125	119	115	111	164	168
CDS;ID=637291052;locus_tag=SPO3587;product=hypothetical protein	19	8	6	16	11	11
CDS;ID=637291053;locus_tag=SPO3588;product=hypothetical protein	1	2	3	1	2	1
CDS;ID=637291054;locus_tag=SPO3589;product=hypothetical protein	1	0	1	1	4	7
CDS;ID=637291055;locus_tag=SPO3590;product=hypothetical protein	9	7	1	10	0	2
CDS;ID=637291056;locus_tag=SPO3591;product=auxin efflux carrier family protein	8	3	3	1	2	3
"CDS;ID=637291057;locus_tag=SPO3592;product=transcriptional regulator, LysR family'	39	36	30	18	28	47
CDS;ID=637291058;locus_tag=SPO3593;product=sulfatase family protein	39	36	35	29	48	42
"CDS;ID=637291059;locus_tag=SPO3594;product=phenylalanyl-tRNA synthetase, alpha sub	130	135	175	167	148	170
"CDS;ID=637291060;locus_tag=SPO3595;product=transcriptional regulator, AraC family'	1	3	7	2	2	1
CDS;ID=637291061;locus_tag=SPO3596;product=pyridoxamine 5'-phosphate oxidase family	7	5	5	3	9	5
"CDS;ID=637291062;locus_tag=SPO3597;product=amine oxidase, flavin-containing"	13	15	7	13	8	9
CDS;ID=637291063;locus_tag=SPO3598;product=50S ribosomal protein L20	166	144	194	211	215	161
CDS;ID=637291064;locus_tag=SPO3599;product=50S ribosomal protein L35	341	230	276	415	510	352
CDS;ID=637291065;locus_tag=SPO3600;product=pyruvate kinase	146	175	96	201	160	163
CDS;ID=637291066;locus_tag=SPO3601;product=N-formylglutamate amidohydrolase family	19	16	28	23	23	17
CDS;ID=637291067;locus_tag=SPO3602;product=hypothetical protein	11	9	8	17	16	14
CDS;ID=637291068;locus_tag=SPO3603;product=thioesterase family protein	7	9	10	7	8	11
"CDS;ID=637291069;locus_tag=SPO3604;product=D-amino acid aminotransferase, putative	54	51	48	42	47	56
CDS;ID=637291070;locus_tag=SPO3605;product=hypothetical protein	23	27	22	39	39	22

CDS;ID=637291071;locus_tag=SPO3606;product=mandelate racemase/muconate lactonizing	31	37	21	34	60	47
CDS;ID=637291072;locus_tag=SPO3607;product=hypothetical protein	36	46	22	44	51	72
CDS;ID=637291073;locus_tag=SPO3608;product=malyl-CoA lyase	56	42	44	47	21	44
CDS;ID=637291074;locus_tag=SPO3609;product=short chain dehydrogenase	23	10	19	11	10	17
CDS;ID=637291075;locus_tag=SPO3610;product=hypothetical protein	5	2	3	3	3	3
CDS;ID=637291076;locus_tag=SPO3611;product=hypothetical protein	55	37	31	19	42	49
"CDS;ID=637291077;locus_tag=SPO3612;product=transcriptional regulator, AsnC family'	14	14	17	15	10	27
"CDS;ID=637291078;locus_tag=SPO3613;product=transporter, LysE family"	95	97	58	45	48	88
CDS;ID=637291079;locus_tag=SPO3614;product=hypothetical protein	8	8	1	1	1	6
"CDS;ID=637291080;locus_tag=SPO3615;product=transcriptional regulator, AraC family'	19	9	5	6	15	9
CDS;ID=637291081;locus_tag=SPO3616;product=acetyl-CoA carboxylase alpha subunit	21	33	19	26	26	43
"CDS;ID=637291082;locus_tag=SPO3617;product=peptidoglycan-binding protein, putative	153	164	153	107	117	166
"CDS;ID=637291083;locus_tag=SPO3618;product=sulfonate ABC transporter, permease pro	14	8	4	7	10	6
"CDS;ID=637291084;locus_tag=SPO3619;product=sulfonate ABC transporter, ATP-binding	32	32	15	26	21	21
"CDS;ID=637291085;locus_tag=SPO3620;product=sulfonate ABC transporter, periplamic s	46	42	32	34	29	32
"CDS;ID=637291086;locus_tag=SPO3621;product=transcriptional regulator, GntR family'	36	21	26	20	32	31
CDS;ID=637291087;locus_tag=SPO3622;product=hypothetical protein	38	25	28	37	17	23
CDS;ID=637291088;locus_tag=SPO3623;product=hypothetical protein	69	59	56	87	76	102
tRNA;ID=640698559;locus_tag=SPO_tRNA-Ala-3	948	495	230	192	38	211
CDS;ID=637291090;locus_tag=SPO3625;product=cold shock protein CspA	106	147	85	167	148	214
CDS;ID=637291091;locus_tag=SPO3626;product=glutamate--cysteine ligase	84	76	43	50	68	57
CDS;ID=637291092;locus_tag=SPO3627;product=hypothetical protein	15	14	5	12	18	20
"CDS;ID=637291093;locus_tag=SPO3628;product=acetyltransferase, GNAT family"	31	22	34	26	44	37
CDS;ID=637291094;locus_tag=SPO3629;product=hypothetical protein	49	31	41	38	67	40
CDS;ID=637291095;locus_tag=SPO3630;product=4-hydroxybenzoate polyprenyl transferase	27	27	23	25	39	24
CDS;ID=637291096;locus_tag=SPO3631;product=OmpA domain protein	401	328	259	290	335	406
CDS;ID=637291097;locus_tag=SPO3632;product=hypothetical protein	59	46	30	34	43	37
"CDS;ID=637291098;locus_tag=SPO3633;product=molybdopterin converting factor, subuni	52	65	37	50	43	54
"CDS;ID=637291099;locus_tag=SPO3634;product=molybdopterin converting factor, subuni	28	30	27	42	41	44
CDS;ID=637291100;locus_tag=SPO3635;product=CDP-diacylglycerol--glycerol-3-phosphate	53	52	45	50	72	85
"CDS;ID=637291101;locus_tag=SPO3636;product=beta-lactamase, putative"	13	7	12	14	20	8
CDS;ID=637291102;locus_tag=SPO3637;product=excinuclease ABC subunit C	40	40	30	40	50	40
CDS;ID=637291103;locus_tag=SPO3638;product=short chain dehydrogenase	24	16	13	15	16	22
CDS;ID=637291104;locus_tag=SPO3639;product=K+-dependent Na+/Ca+ exchanger related-p	42	41	37	38	30	55
"CDS;ID=637291105;locus_tag=SPO3640;product=peptidase, family S49"	119	66	88	92	125	116
"CDS;ID=637291106;locus_tag=SPO3641;product=ABC transporter, permease protein"	21	26	26	25	38	18
"CDS;ID=637291107;locus_tag=SPO3642;product=ABC transporter, ATP-binding protein"	41	22	33	33	43	43
"CDS;ID=637291108;locus_tag=SPO3901;product=carbon monoxide dehydrogenase G protein	1	2	3	1	3	1
CDS;ID=637291109;locus_tag=SPO3643;product=integral membrane protein	3	2	3	2	4	5
CDS;ID=637291110;locus_tag=SPO3644;product=hypothetical protein	4	5	4	3	0	9
"CDS;ID=637291111;locus_tag=SPO3645;product=transcriptional regulator, putative"	2	1	1	2	0	0
CDS;ID=637291112;locus_tag=SPO3646;product=enoyl-CoA hydratase	4	8	7	3	7	7
CDS;ID=637291113;locus_tag=SPO3647;product=CAIB/BAIF family protein	23	25	12	15	17	13

CDS;ID=637291114;locus_tag=SPO3648;product=uroporphyrinogen decarboxylase	35	42	40	44	67	68
CDS;ID=637291115;locus_tag=SPO3649;product=porphobilinogen deaminase	24	15	7	10	22	21
CDS;ID=637291116;locus_tag=SPO3650;product=adenylate/guanylate cyclase	8	2	3	7	11	4
CDS;ID=637291117;locus_tag=SPO3651;product=fatty acid desaturase family protein	27	27	23	24	22	17
CDS;ID=637291118;locus_tag=SPO3652;product=hypothetical protein	11	21	6	16	10	11
CDS;ID=637291119;locus_tag=SPO3653;product=coproporphyrinogen III oxidase	73	56	69	75	43	59
"CDS;ID=637291120;locus_tag=SPO3654;product=7-alpha-hydroxysteroid dehydrogenase, p	35	44	32	43	56	37
"CDS;ID=637291121;locus_tag=SPO3655;product=methyltransferase, putative"	21	21	29	31	30	24
"CDS;ID=637291122;locus_tag=SPO3656;product=ATP-dependent Clp protease, adaptor pro	62	68	58	45	44	61
"CDS;ID=637291123;locus_tag=SPO3657;product=hydrolase, haloacid delahogenase-like f	7	0	3	7	11	8
CDS;ID=637291124;locus_tag=SPO3658;product=D-alanyl-D-alanine carboxypeptidase fami	172	161	155	179	188	215
CDS;ID=637291125;locus_tag=SPO3659;product=hypothetical protein	10	12	11	8	8	5
"CDS;ID=637291126;locus_tag=SPO3660;product=urea amidolyase, homolog"	6	8	5	5	6	8
CDS;ID=637291127;locus_tag=SPO3661;product=allophanate hydrolase family protein	6	0	0	1	1	2
CDS;ID=637291128;locus_tag=SPO3662;product=hypothetical protein	5	3	0	2	2	2
"CDS;ID=637291129;locus_tag=SPO3663;product=TRAP dicarboxylate family transporter,	20	10	9	15	9	11
CDS;ID=637291130;locus_tag=SPO3664;product=hypothetical protein	14	11	6	6	3	12
"CDS;ID=637291131;locus_tag=SPO3665;product=bacterial extracellular solute-binding	48	33	20	17	34	24
"CDS;ID=637291132;locus_tag=SPO3666;product=oxidoreductase, FAD-binding"	67	43	34	39	80	61
CDS;ID=637291133;locus_tag=SPO3667;product=muconate cycloisomerase I	0	4	4	3	0	3
"CDS;ID=637291134;locus_tag=SPO3668;product=transcriptional regulator, LysR family"	11	9	6	8	9	6
tRNA;ID=640698560;locus_tag=SPO_tRNA-Ser-5	199	180	90	115	138	169
CDS;ID=637291136;locus_tag=SPO3902;product=transglycosylase domain protein	23	39	23	24	27	36
CDS;ID=637291137;locus_tag=SPO3670;product=hypothetical protein	2	9	2	3	2	5
CDS;ID=637291138;locus_tag=SPO3671;product=muticopper oxidase domain protein	63	75	51	69	48	64
CDS;ID=637291139;locus_tag=SPO3672;product=hypothetical protein	12	10	4	10	9	12
CDS;ID=637291140;locus_tag=SPO3673;product=type I secretion target repeat protein	2625	2703	2362	2653	3747	2730
"CDS;ID=637291141;locus_tag=SPO3674;product=ISSpo7, transposase"	2	0	1	5	2	5
CDS;ID=637291142;locus_tag=SPO3675;product=hypothetical protein	3	0	0	0	2	0
CDS;ID=637291143;locus_tag=SPO3676;product=acetylornithine deacetylase	7	14	6	8	21	8
"CDS;ID=637291144;locus_tag=SPO3677;product=transcriptional regulator, LysR family"	9	9	6	11	2	14
CDS;ID=637291145;locus_tag=SPO3678;product=Rieske 2Fe-2S domain protein	19	23	16	8	32	22
CDS;ID=637291146;locus_tag=SPO3679;product=hypothetical protein	7	5	3	13	8	12
CDS;ID=637291147;locus_tag=SPO3680;product=endoribonuclease L-PSP family protein	18	15	29	13	10	8
CDS;ID=637291148;locus_tag=SPO3681;product=Rieske 2Fe-2S domain protein	38	47	39	40	29	47
CDS;ID=637291149;locus_tag=SPO3682;product=hypothetical protein	19	16	14	28	18	10
tRNA;ID=640698561;locus_tag=SPO_tRNA-Arg-5	51	15	12	10	1	15
"CDS;ID=637291151;locus_tag=SPO3684;product=transcriptional regulator, LacI family"	58	63	38	52	61	73
CDS;ID=637291152;locus_tag=SPO3685;product=hypothetical protein	22	26	8	16	16	23
CDS;ID=637291153;locus_tag=SPO3686;product=HpcH/HpaI aldolase family protein	11	22	12	14	12	12
"CDS;ID=637291154;locus_tag=SPO3687;product=decarboxylase, pyridoxal-dependent"	21	16	3	16	15	12
CDS;ID=637291155;locus_tag=SPO3688;product=extradiol ring-cleavage dioxygenase fami	15	9	5	9	17	29
"CDS;ID=637291156;locus_tag=SPO3689;product=transcriptional regulator, MarR family"	91	73	90	79	65	103

"CDS;ID=637291157;locus_tag=SPO3690;product=gentisate 1,2-dioxygenase"	45	40	43	37	40	48
CDS;ID=637291158;locus_tag=SPO3691;product=fumarylacetoacetate hydrolase family pro	27	25	29	25	45	38
CDS;ID=637291159;locus_tag=SPO3692;product=salicylate hydroxylase	112	114	77	103	99	91
"CDS;ID=637291160;locus_tag=SPO3693;product=TRAP dicarboxylate transporter, DctP su	64	35	42	31	35	33
CDS;ID=637291161;locus_tag=SPO3694;product=hypothetical protein	11	5	5	11	5	11
"CDS;ID=637291162;locus_tag=SPO3695;product=TRAP dicarboxylate transporter, DctM su	4	6	5	3	10	6
CDS;ID=637291163;locus_tag=SPO3696;product=feruloyl-CoA synthase	11	7	9	10	8	8
CDS;ID=637291164;locus_tag=SPO3697;product=benzoate-coenzyme A ligase	15	18	10	23	18	16
"CDS;ID=637291165;locus_tag=SPO3698;product=hydrolase, putative"	10	8	5	2	2	6
CDS;ID=637291166;locus_tag=SPO3699;product=hypothetical protein	10	11	9	4	5	11
CDS;ID=637291167;locus_tag=SPO3700;product=enoyl-CoA hydratase	34	23	36	27	39	31
"CDS;ID=637291168;locus_tag=SPO3701;product=benzoyl-CoA oxygenase, B subunit"	18	17	15	15	13	28
CDS;ID=637291169;locus_tag=SPO3702;product=hypothetical protein	10	8	13	5	11	11
"CDS;ID=637291170;locus_tag=SPO3703;product=benzoyl-CoA oxygenase, A subunit"	21	17	15	17	34	16
CDS;ID=637291171;locus_tag=SPO3704;product=regulator of the anaerobic catobolism of	14	18	24	6	12	19
"CDS;ID=637291172;locus_tag=SPO3705;product=branched-chain amino acid ABC transport	164	65	86	50	85	94
"CDS;ID=637291173;locus_tag=SPO3706;product=branched-chain amino acid ABC transport	27	14	8	3	7	10
"CDS;ID=637291174;locus_tag=SPO3707;product=branched-chain amino acid ABC transport	28	5	11	5	16	9
"CDS;ID=637291175;locus_tag=SPO3708;product=branched-chain amino acid ABC transport	29	14	13	12	16	20
"CDS;ID=637291176;locus_tag=SPO3709;product=branched-chain amino acid ABC transport	38	14	37	15	20	18
CDS;ID=637291177;locus_tag=SPO3710;product=hypothetical protein	12	7	4	4	5	3
CDS;ID=637291178;locus_tag=SPO3711;product=hypothetical protein	19	18	12	11	10	21
CDS;ID=637291179;locus_tag=SPO3712;product=aspartate-semialdehyde dehydrogenase	36	31	26	43	46	49
CDS;ID=637291180;locus_tag=SPO3713;product=hypothetical protein	80	78	70	57	80	83
CDS;ID=637291181;locus_tag=SPO3714;product=hypothetical protein	5	2	4	5	8	4
"CDS;ID=637291182;locus_tag=SPO3715;product=carbonic anhydrase, putative"	80	52	65	101	94	91
CDS;ID=637291183;locus_tag=SPO3716;product=hypothetical protein	3	1	0	1	4	0
CDS;ID=637291184;locus_tag=SPO3717;product=cytosol aminopeptidase family protein	36	25	29	21	50	36
CDS;ID=637291185;locus_tag=SPO3718;product=NLP/P60 family protein	13	14	3	12	19	17
"CDS;ID=637291186;locus_tag=SPO3719;product=thiosulfate sulfurtransferase, putative	84	61	75	69	108	92
CDS;ID=637291187;locus_tag=SPO3720;product=aspartate aminotransferase	204	148	128	160	160	139
CDS;ID=637291188;locus_tag=SPO3721;product=hypothetical protein	18	8	6	17	19	14
CDS;ID=637291189;locus_tag=SPO3722;product=Rrf2 family protein	2	2	1	1	1	2
CDS;ID=637291190;locus_tag=SPO3723;product=ammonium transporter	5	2	1	2	4	9
CDS;ID=637291191;locus_tag=SPO3724;product=nitrogen regulatory protein P-II	1	1	2	0	3	1
"CDS;ID=637291192;locus_tag=SPO3725;product=penicillin-binding protein, 1A family"	85	90	56	83	80	76
CDS;ID=637291193;locus_tag=SPO3726;product=toluene tolerance family protein	43	38	30	43	44	32
"CDS;ID=637291194;locus_tag=SPO3727;product=VacJ lipoprotein, putative"	251	227	192	157	152	231
CDS;ID=637291195;locus_tag=SPO3728;product=type I secretion system ATPase	139	129	87	108	113	114
"CDS;ID=637291196;locus_tag=SPO3729;product=type I secretion membrane fusion protei	102	98	109	93	112	112
CDS;ID=637291197;locus_tag=SPO3730;product=endoribonuclease L-PSP family protein	94	92	63	68	94	98
"CDS;ID=637291198;locus_tag=SPO3731;product=glycerophosphoryl diester phosphodieste	30	25	32	20	24	27
CDS;ID=637291199;locus_tag=SPO3732;product=hypothetical protein	86	54	51	39	46	56

CDS;ID=637291200;locus_tag=SPO3733;product=4a-hydroxytetrahydrobiopterin dehydratas	17	7	11	7	5	9
"CDS;ID=637291201;locus_tag=SPO3734;product=transcriptional regulator, MerR family'	6	2	3	2	5	2
CDS;ID=637291202;locus_tag=SPO3735;product=major facilitator family protein	6	3	6	2	7	9
"CDS;ID=637291203;locus_tag=SPO3736;product=antioxidant, AhpC/Tsa family"	25	18	12	16	12	14
CDS;ID=637291204;locus_tag=SPO3737;product=pyridine nucleotide-disulphide oxidoredu	44	49	25	35	42	68
"CDS;ID=637291205;locus_tag=SPO3738;product=methyltransferase, putative"	12	10	2	13	20	9
CDS;ID=637291206;locus_tag=SPO3739;product=hydantoinase/oxoprolinase family protein	20	20	18	14	24	41
CDS;ID=637291207;locus_tag=SPO3740;product=methionine sulfoxide reductase A	30	46	37	31	33	43
CDS;ID=637291208;locus_tag=SPO3741;product=methionine sulfoxide reductase B	46	47	44	30	64	42
CDS;ID=637291209;locus_tag=SPO3742;product=glutathione peroxidase famly protein	9	4	6	2	6	9
CDS;ID=637291210;locus_tag=SPO3743;product=hypothetical protein	25	10	7	10	23	12
CDS;ID=637291211;locus_tag=SPO3744;product=DNA-binding protein HU	159	115	147	144	171	187
CDS;ID=637291212;locus_tag=SPO3745;product=AMP nucleosidase	77	76	72	64	100	80
CDS;ID=637291213;locus_tag=SPO3746;product=adenine deaminase	122	119	91	81	125	144
CDS;ID=637291214;locus_tag=SPO3747;product=hypothetical protein	3	4	5	8	15	9
"CDS;ID=637291215;locus_tag=SPO3748;product=transcriptional regulator, LysR family'	5	4	2	2	6	7
CDS;ID=637291216;locus_tag=SPO3749;product=hypothetical protein	8	7	12	12	16	13
CDS;ID=637291217;locus_tag=SPO3750;product=hypothetical protein	8	7	3	4	6	7
CDS;ID=637291218;locus_tag=SPO3751;product=ExsB	14	9	13	20	11	25
"CDS;ID=637291219;locus_tag=SPO3903;product=6-pyruvoyl tetrahydropterin synthase, p	1	6	5	4	0	10
CDS;ID=637291220;locus_tag=SPO3752;product=radical SAM domain protein	13	10	6	10	7	4
CDS;ID=637291221;locus_tag=SPO3753;product=GTP cyclohydrolase family protein	6	11	9	5	8	8
CDS;ID=637291222;locus_tag=SPO3754;product=hypothetical protein	9	5	14	3	5	8
CDS;ID=637291223;locus_tag=SPO3755;product=hypothetical protein	44	34	38	58	65	47
CDS;ID=637291224;locus_tag=SPO3756;product=OmpA domain protein	25	26	17	9	20	9
CDS;ID=637291225;locus_tag=SPO3757;product=alkylhydroperoxidase AhpD family core dc	42	36	31	33	39	40
"CDS;ID=637291226;locus_tag=SPO3758;product=acetyltransferase, GNAT family"	46	21	25	32	42	28
CDS;ID=637291227;locus_tag=SPO3759;product=molybdenum cofactor biosynthesis domain	94	63	73	62	101	67
CDS;ID=637291228;locus_tag=SPO3760;product=sugar fermentation stimulation protein A	14	17	14	16	18	18
"CDS;ID=637291229;locus_tag=SPO3761;product=methionine aminopeptidase, type I"	125	98	83	113	80	136
"CDS;ID=637291230;locus_tag=SPO3762;product=HAD-superfamily hydrolase, subfamily IA	44	56	36	57	31	43
CDS;ID=637291231;locus_tag=SPO3763;product=iron-sulfur cluster-binding protein	35	45	25	40	38	43
CDS;ID=637291232;locus_tag=SPO3764;product=glutathione S-transferase family protein	141	137	124	134	162	147
CDS;ID=637291233;locus_tag=SPO3765;product=hypothetical protein	4	4	0	2	1	4
CDS;ID=637291234;locus_tag=SPO3766;product=monofunctional biosynthetic peptidoglyca	49	40	47	51	57	38
CDS;ID=637291235;locus_tag=SPO3767;product=hypothetical protein	41	20	16	32	37	25
"CDS;ID=637291236;locus_tag=SPO3768;product=glutamate synthase, large subunit"	208	160	123	142	180	158
CDS;ID=637291237;locus_tag=SPO3769;product=hypothetical protein	20	14	14	12	15	17
"CDS;ID=637291238;locus_tag=SPO3770;product=glutamate synthase, small subunit"	85	55	66	64	101	100
"CDS;ID=637291239;locus_tag=SPO3771;product=undecaprenol kinase, putative"	35	32	23	26	27	32
"CDS;ID=637291240;locus_tag=SPO3772;product=NADH ubiquinone oxidoreductase, putativ	131	126	95	127	97	132
tRNA;ID=640698562;locus_tag=SPO_tRNA-Leu-6	1134	741	299	291	62	321
"CDS;ID=637291242;locus_tag=SPO3774;product=oligopeptide/dipeptide ABC transporter,	187	166	131	163	130	153

"CDS;ID=637291243;locus_tag=SPO3775;product=oligopeptide/dipeptide ABC transporter,	16	20	18	11	27	15
"CDS;ID=637291244;locus_tag=SPO3776;product=oligopeptide/dipeptide ABC transporter,	18	29	20	10	15	17
"CDS;ID=637291245;locus_tag=SPO3777;product=oligopeptide/dipeptide ABC transporter,	24	22	7	23	14	5
"CDS;ID=637291246;locus_tag=SPO3778;product=oligopeptide/dipeptide ABC transporter,	53	45	56	31	28	31
"CDS;ID=637291247;locus_tag=SPO3779;product=transcriptional regulator, LysR family"	25	13	10	12	13	14
CDS;ID=637291248;locus_tag=SPO3780;product=hypothetical protein	31	42	32	42	44	65
CDS;ID=637291249;locus_tag=SPO3781;product=hypothetical protein	6	5	14	2	4	4
CDS;ID=637291250;locus_tag=SPO3782;product=hypothetical protein	4	4	3	3	1	7
"CDS;ID=637291251;locus_tag=SPO3783;product=sugar ABC transporter, ATP-binding prot	1	7	5	0	5	5
"CDS;ID=637291252;locus_tag=SPO3784;product=sugar ABC transporter, ATP-binding prot	1	3	1	1	3	3
"CDS;ID=637291253;locus_tag=SPO3785;product=sugar ABC transporter, permease protein	2	4	2	1	6	3
"CDS;ID=637291254;locus_tag=SPO3786;product=sugar ABC transporter, permease protein	3	2	0	5	2	2
"CDS;ID=637291255;locus_tag=SPO3787;product=sugar ABC transporter, periplasmic suga	65	70	41	50	50	74
CDS;ID=637291256;locus_tag=SPO3788;product=acetoin catabolism regulatory protein	8	5	3	6	3	12
CDS;ID=637291257;locus_tag=SPO3789;product=4-carboxymuconolactone decarboxylase dom	2	0	1	2	1	3
CDS;ID=637291258;locus_tag=SPO3790;product=dihydrolipoamide acetyltransferase	2	3	0	2	0	2
"CDS;ID=637291259;locus_tag=SPO3791;product=acetoin dehydrogenase complex, E1 compo	6	2	6	6	6	4
"CDS;ID=637291260;locus_tag=SPO3792;product=acetoin dehydrogenase complex, E1 compo	14	2	5	4	5	2
CDS;ID=637291261;locus_tag=SPO3793;product=acetoin catabolism protein X	9	2	11	5	8	10
CDS;ID=637291262;locus_tag=SPO3794;product=hypothetical protein	0	3	1	1	0	0
CDS;ID=637291263;locus_tag=SPO3795;product=RNA polymerase sigma-54 factor	28	35	24	21	27	22
tRNA;ID=640698563;locus_tag=SPO_tRNA-Met-5	845	424	261	318	56	327
rRNA;ID=640698564;locus_tag=SPO_Sp5SF;product=5S	8541	6154	3230	3197	1810	3503
tRNA;ID=640698566;locus_tag=SPO_tRNA-Ala-4	2040	818	401	397	88	279
tRNA;ID=640698567;locus_tag=SPO_tRNA-Ile-3	1589	722	382	411	117	378
CDS;ID=637291270;locus_tag=SPO3802;product=hypothetical protein	2	1	6	0	3	3
"CDS;ID=637291271;locus_tag=SPO3803;product=transcriptional regulator, AraC family"	5	3	4	3	4	1
CDS;ID=637291272;locus_tag=SPO3804;product=acyl-CoA dehydrogenase family protein	20	29	27	27	22	18
CDS;ID=637291273;locus_tag=SPO3805;product=enoyl-CoA hydratase	23	16	13	6	23	10
CDS;ID=637291274;locus_tag=SPO3806;product=SsrA-binding protein	222	186	219	266	377	174
CDS;ID=637291275;locus_tag=SPO3807;product=hypothetical protein	15	26	6	16	25	27
CDS;ID=637291276;locus_tag=SPO3808;product=hypothetical protein	1	4	0	0	0	1
"CDS;ID=637291277;locus_tag=SPO3809;product=transcriptional regulator, ArsR family"	4	9	5	9	20	15
CDS;ID=637291278;locus_tag=SPO3810;product=phosphoglyceromutase	52	50	45	64	67	64
CDS;ID=637291279;locus_tag=SPO3811;product=hypothetical protein	90	89	70	77	106	129
CDS;ID=637291280;locus_tag=SPO3812;product=carboxyl-terminal protease family protei	157	112	110	111	161	124
CDS;ID=637291281;locus_tag=SPO3813;product=hypothetical protein	22	26	15	10	15	11
CDS;ID=637291282;locus_tag=SPO3814;product=hypothetical protein	63	50	42	50	35	67
"CDS;ID=637291283;locus_tag=SPO3815;product=ABC transporter, permease protein"	48	40	27	16	34	38
CDS;ID=637291284;locus_tag=SPO3816;product=CAAX amino terminal protease family prot	9	4	1	4	5	5
"CDS;ID=637291285;locus_tag=SPO3817;product=acetyl-CoA carboxylase, carboxyl transf	161	155	130	181	194	182
CDS;ID=637291286;locus_tag=SPO3818;product=folC bifunctional protein	60	24	48	52	47	42
"CDS;ID=637291287;locus_tag=SPO3819;product=ATPase, AFG1 family"	99	70	84	104	103	133

"CDS;ID=637291288;locus_tag=SPO3820;product=transmembrane transporter, major facil	33	29	25	16	29	42
CDS;ID=637291289;locus_tag=SPO3821;product=ornithine cyclodeaminase/mu-crystallin f	16	19	11	14	25	24
"CDS;ID=637291290;locus_tag=SPO3822;product=HAD-superfamily hydrolase, subfamily IA	41	46	44	41	42	44
CDS;ID=637291291;locus_tag=SPO3823;product=ribosomal protein L23	168	158	129	125	168	116
CDS;ID=637291292;locus_tag=SPO3824;product=50S ribosomal protein L4	379	377	311	341	527	358
CDS;ID=637291293;locus_tag=SPO3825;product=50S ribosomal protein L3	379	359	362	411	705	381
CDS;ID=637291294;locus_tag=SPO3826;product=30S ribosomal protein S10	687	602	641	858	1281	700
CDS;ID=637291295;locus_tag=SPO3827;product=YaiI/YqxD family protein	4	6	2	5	2	5
CDS;ID=637291296;locus_tag=SPO3828;product=soluble pyridine nucleotide transhydroge	55	48	37	48	66	74
"CDS;ID=637291297;locus_tag=SPO3829;product=S-formylglutathione hydrolase, putative	28	24	16	14	29	27
CDS;ID=637291298;locus_tag=SPO3830;product=peptidase T	51	64	38	39	54	40
"CDS;ID=637291299;locus_tag=SPO3831;product=hydrolase, alpha/beta fold family"	18	13	12	16	15	18
CDS;ID=637291300;locus_tag=SPO3832;product=ornithine cyclodeaminase/mu-crystallin f	13	5	9	9	5	12
"CDS;ID=637291301;locus_tag=SPO3833;product=ATP-dependent RNA helicase, DEAD/DEAH b	61	52	31	60	77	67
CDS;ID=637291302;locus_tag=SPO3834;product=dihydrodipicolinate reductase	49	33	34	54	58	63
CDS;ID=637291303;locus_tag=SPO3835;product=ribosome-binding factor A	45	63	37	67	72	60
CDS;ID=637291304;locus_tag=SPO3836;product=hypothetical protein	41	28	21	38	46	50
CDS;ID=637291305;locus_tag=SPO3837;product=tRNA pseudouridine synthase B	32	24	14	28	31	32
CDS;ID=637291306;locus_tag=SPO3838;product=hypothetical protein	13	13	18	15	14	17
CDS;ID=637291307;locus_tag=SPO3839;product=type I secretion target repeat protein	58	43	43	43	50	60
CDS;ID=637291308;locus_tag=SPO3840;product=ribosomal protein S15	287	224	269	274	350	260
"CDS;ID=637291309;locus_tag=SPO3841;product=transcriptional regulator, TetR family"	32	16	11	15	21	23
CDS;ID=637291310;locus_tag=SPO3842;product=hypothetical protein	16	18	13	10	15	24
"CDS;ID=637291311;locus_tag=SPO3843;product=malonate transporter, putative"	13	19	16	17	13	15
CDS;ID=637291312;locus_tag=SPO3844;product=DNA polymerase I	95	97	64	108	101	104
CDS;ID=637291313;locus_tag=SPO3845;product=hypothetical protein	2	5	9	3	4	2
CDS;ID=637291314;locus_tag=SPO3846;product=hypothetical protein	22	15	15	14	21	21
"CDS;ID=637291315;locus_tag=SPO3847;product=ABC transporter, ATP-binding protein"	49	47	49	37	60	45
CDS;ID=637291316;locus_tag=SPO3848;product=sterol desaturase family protein	36	24	28	19	15	27
CDS;ID=637291317;locus_tag=SPO3849;product=hypothetical protein	0	0	0	0	0	2
CDS;ID=637291318;locus_tag=SPO3850;product=glutathione-dependent formaldehyde dehyd	80	51	39	41	48	53
CDS;ID=637291319;locus_tag=SPO3851;product=HemY domain protein	73	88	74	70	83	69
CDS;ID=637291320;locus_tag=SPO3852;product=hypothetical protein	86	68	47	75	83	81
"CDS;ID=637291321;locus_tag=SPO3853;product=uroporphyrinogen-III synthase, putative	18	14	7	9	14	20
"CDS;ID=637291322;locus_tag=SPO3854;product=O-sialoglycoprotein endopeptidase, puta	33	31	28	51	39	42
CDS;ID=637291323;locus_tag=SPO3855;product=glycerol-3-phosphate dehydrogenase (NAD(	38	29	26	30	41	31
CDS;ID=637291324;locus_tag=SPO3856;product=YCII-related domain protein	26	12	18	19	19	25
CDS;ID=637291325;locus_tag=SPO3857;product=hypothetical protein	29	15	14	26	15	20
CDS;ID=637291326;locus_tag=SPO3858;product=hypothetical protein	20	25	9	18	16	22
CDS;ID=637291327;locus_tag=SPO3859;product=hypothetical protein	131	96	121	94	116	123
CDS;ID=637291328;locus_tag=SPO3860;product=hypothetical protein	31	36	31	51	42	39
CDS;ID=637291329;locus_tag=SPO3861;product=S-adenosyl-L-homocysteine hydrolase	231	183	192	268	302	251
"CDS;ID=637291330;locus_tag=SPO3862;product=lipoprotein, putative"	0	0	0	0	0	1

CDS;ID=637291331;locus_tag=SPO3863;product=HD domain protein	8	10	11	5	12	11
CDS;ID=637291332;locus_tag=SPO3864;product=hypothetical protein	45	47	31	40	49	54
CDS;ID=637291333;locus_tag=SPO3865;product=photosynthetic apparatus regulatory prot	51	69	39	48	51	55
CDS;ID=637291334;locus_tag=SPO3866;product=regulatory protein SenC	66	43	45	48	51	49
CDS;ID=637291335;locus_tag=SPO3867;product=sensor histidine kinase RegB	19	13	4	18	12	8
CDS;ID=637291336;locus_tag=SPO3868;product=hypothetical protein	27	28	21	28	22	30
CDS;ID=637291337;locus_tag=SPO3869;product=conserved hypothetical protein TIGR0015(	11	16	9	16	18	11
CDS;ID=637291338;locus_tag=SPO3870;product=hypothetical protein	17	11	8	11	14	12
CDS;ID=637291339;locus_tag=SPO3871;product=nucleotidyltransferase family protein	22	15	14	9	17	15
CDS;ID=637291340;locus_tag=SPO3872;product=hypothetical protein	48	32	26	30	33	29
"CDS;ID=637291341;locus_tag=SPO3873;product=ATP-dependent DNA helicase, UvrD/Rep fa	52	46	30	23	26	37
CDS;ID=637291342;locus_tag=SPO3874;product=thioredoxin	100	98	83	88	73	95
CDS;ID=637291343;locus_tag=SPO3875;product=hypothetical protein	16	14	19	33	20	29
CDS;ID=637291344;locus_tag=SPO3876;product=hypothetical protein	11	10	9	17	9	14
CDS;ID=637291345;locus_tag=SPO3877;product=transcriptional regulator/arsenate reduc	5	3	2	0	6	7
"CDS;ID=637291346;locus_tag=SPO3878;product=glyceraldehyde-3-phosphate dehydrogenas	4	0	9	5	1	3
CDS;ID=637291347;locus_tag=SPO3879;product=hypothetical protein	30	13	15	8	14	19
CDS;ID=637291348;locus_tag=SPO3880;product=ATP-dependent protease peptidase subunit	55	45	42	70	59	64
"CDS;ID=637291349;locus_tag=SPO3881;product=twin-arginine translocation pathway sig	5	8	9	11	12	3
CDS;ID=637291350;locus_tag=SPO3882;product=ATP-dependent protease ATP-binding subur	117	114	100	142	127	151
"CDS;ID=637291351;locus_tag=SPO3883;product=lipoprotein, putative"	11	9	13	19	19	11
CDS;ID=637291352;locus_tag=SPO3884;product=smr domain protein	5	9	5	13	7	10
CDS;ID=637291353;locus_tag=SPO3885;product=MltA/3D domain protein	18	13	14	22	11	23
"CDS;ID=637291354;locus_tag=SPO3886;product=transporter, Tim44 family"	109	85	94	136	113	111
CDS;ID=637291355;locus_tag=SPO3887;product=FxsA	37	19	24	38	27	30
CDS;ID=637291356;locus_tag=SPO3888;product=protein-export protein SecB	60	36	30	42	45	48
"CDS;ID=637291357;locus_tag=SPO3889;product=DNA polymerase III, epsilon subunit"	26	28	25	21	27	20
CDS;ID=637291358;locus_tag=SPO3890;product=dephospho-CoA kinase	16	16	16	19	15	15
CDS;ID=637291359;locus_tag=SPO3891;product=shikimate 5-dehydrogenase	41	44	36	29	42	43
CDS;ID=637291360;locus_tag=SPO3892;product=Maf	43	36	24	22	25	26
CDS;ID=637291361;locus_tag=SPO3893;product=hypothetical protein	27	16	19	13	22	15
CDS;ID=637291362;locus_tag=SPO3894;product=transcription termination factor Rho	136	113	133	168	162	152
CDS;ID=637291363;locus_tag=SPO3895;product=tRNA modification GTPase	49	60	49	60	71	54
CDS;ID=637613545;locus_tag=SPOA0449;product=phytanoyl-CoA dioxygenase family protei	7	3	0	1	6	6
CDS;ID=637613546;locus_tag=SPOA0001;product=hypothetical protein	26	41	31	30	32	33
CDS;ID=637613547;locus_tag=SPOA0002;product=tRNA-i(6)A37 thiotransferase enzyme Mia	58	46	58	62	76	80
CDS;ID=637613548;locus_tag=SPOA0003;product=hypothetical protein	19	31	11	23	29	36
CDS;ID=637613549;locus_tag=SPOA0004;product=OmpA family protein	33	16	21	29	27	18
"CDS;ID=637613550;locus_tag=SPOA0005;product=lipoprotein, putative"	88	64	32	69	44	90
CDS;ID=637613551;locus_tag=SPOA0006;product=PhoH family protein	252	267	181	127	246	241
CDS;ID=637613552;locus_tag=SPOA0007;product=hypothetical protein	35	39	30	41	39	44
CDS;ID=637613553;locus_tag=SPOA0008;product=conserved hypothetical protein TIGR0004	85	75	51	71	72	75
"CDS;ID=637613554;locus_tag=SPOA0009;product=hemolysin, putative"	165	108	126	143	147	131

CDS;ID=637613555;locus_tag=SPOA0010;product=apolipoprotein N-acyltransferase	47	60	44	51	40	67
CDS;ID=637613556;locus_tag=SPOA0011;product=S-adenosylmethionine synthetase	59	61	49	70	71	63
"CDS;ID=637613557;locus_tag=SPOA0012;product=DNA topology modulation kinase FlaR, p	5	3	2	5	6	9
CDS;ID=637613558;locus_tag=SPOA0013;product=hypothetical protein	20	26	10	16	7	8
CDS;ID=637613559;locus_tag=SPOA0014;product=tRNA (guanine-N(7)-)-methyltransferase	62	48	32	71	79	105
CDS;ID=637613560;locus_tag=SPOA0015;product=3-phosphoshikimate 1-carboxyvinyltransf	57	51	25	36	53	68
CDS;ID=637613561;locus_tag=SPOA0017;product=cytidylate kinase	45	27	23	25	34	32
CDS;ID=637613562;locus_tag=SPOA0018;product=YHS domain protein	5	2	5	6	4	2
CDS;ID=637613563;locus_tag=SPOA0019;product=hypothetical protein	6	10	3	3	2	7
CDS;ID=637613564;locus_tag=SPOA0020;product=alanine racemase domain protein	9	9	13	4	7	12
"CDS;ID=637613565;locus_tag=SPOA0021;product=oxidoreductase, FAD-binding"	8	6	8	12	13	13
CDS;ID=637613566;locus_tag=SPOA0022;product=cytochrome c5	2	2	1	1	7	3
"CDS;ID=637613567;locus_tag=SPOA0023;product=transcriptional regulator, TetR family	2	4	7	1	5	7
"CDS;ID=637613568;locus_tag=SPOA0024;product=2-oxo-hepta-3-ene-1,7-dioic acid hydra	4	6	3	10	13	12
CDS;ID=637613569;locus_tag=SPOA0025;product=fumarylacetoacetate hydrolase family pr	29	20	11	31	18	22
"CDS;ID=637613570;locus_tag=SPOA0026;product=3,4-dihydroxyphenylacetate 2,3-dioxyge	56	33	43	41	34	54
CDS;ID=637613571;locus_tag=SPOA0027;product=5-carboxy-2-hydroxymuconate semialdehyd	11	8	8	12	9	15
CDS;ID=637613572;locus_tag=SPOA0028;product=Na(+)-translocating NADH-quinone reduct	123	118	105	133	159	137
CDS;ID=637613573;locus_tag=SPOA0029;product=Na(+)-translocating NADH-quinone reduct	65	69	59	80	89	94
CDS;ID=637613574;locus_tag=SPOA0030;product=Na(+)-translocating NADH-quinone reduct	121	107	138	140	151	151
CDS;ID=637613575;locus_tag=SPOA0031;product=NADH-ubiquinone oxidoreductase	91	115	91	102	125	131
CDS;ID=637613576;locus_tag=SPOA0032;product=Na(+)-translocating NADH-quinone reduct	65	56	57	75	83	79
CDS;ID=637613577;locus_tag=SPOA0033;product=Na(+)-translocating NADH-quinone reduct	132	88	82	92	93	85
CDS;ID=637613578;locus_tag=SPOA0034;product=CBS domain protein	32	27	20	20	22	23
CDS;ID=637613579;locus_tag=SPOA0035;product=hypothetical protein	42	24	10	20	26	25
CDS;ID=637613580;locus_tag=SPOA0036;product=thiamin biosynthesis lipoprotein ApbE	77	82	93	129	95	117
CDS;ID=637613581;locus_tag=SPOA0037;product=hypothetical protein	17	8	11	11	8	9
"CDS;ID=637613582;locus_tag=SPOA0038;product=transcriptional regulator, LuxR family	56	60	38	28	42	59
CDS;ID=637613583;locus_tag=SPOA0039;product=hypothetical protein	126	96	114	98	133	116
CDS;ID=637613584;locus_tag=SPOA0040;product=crcB family protein	5	9	5	4	7	6
CDS;ID=637613585;locus_tag=SPOA0041;product=crcB family protein	6	4	4	11	12	3
CDS;ID=637613586;locus_tag=SPOA0042;product=hypothetical protein	15	13	6	6	14	14
"CDS;ID=637613587;locus_tag=SPOA0043;product=protocatechuate 3,4-dioxygenase alpha	7	9	4	7	6	5
"CDS;ID=637613588;locus_tag=SPOA0044;product=protocatechuate 3,4-dioxygenase beta s	13	10	7	7	9	7
CDS;ID=637613589;locus_tag=SPOA0045;product=4-carboxymuconolactone decarboxylase	25	16	11	9	7	22
CDS;ID=637613590;locus_tag=SPOA0046;product=4-hydroxybenzoate 3-monooxygenase	58	52	36	50	39	41
CDS;ID=637613591;locus_tag=SPOA0047;product=pca operon transcriptional activator Pc	11	6	7	2	8	9
CDS;ID=637613592;locus_tag=SPOA0048;product=hypothetical protein	0	1	1	2	0	0
CDS;ID=637613593;locus_tag=SPOA0049;product=nitrous-oxide reductase transcriptional	3	4	11	4	3	4
CDS;ID=637613594;locus_tag=SPOA0050;product=nitrous-oxide reductase precurser	11	8	8	22	5	15
CDS;ID=637613595;locus_tag=SPOA0051;product=nitrous oxide maturation protein NosD	0	0	0	1	3	5
"CDS;ID=637613596;locus_tag=SPOA0052;product=ABC transporter, ATP-binding protein N	0	1	0	0	0	0
CDS;ID=637613597;locus_tag=SPOA0053;product=nitrous oxide maturation protein NosY	0	0	2	0	0	1

CDS;ID=637613598;locus_tag=SPOA0054;product=nitrous oxide reductase accessory prote	2	0	2	0	1	0
CDS;ID=637613599;locus_tag=SPOA0055;product=cytochrome c family protein	5	7	7	6	13	11
"CDS;ID=637613600;locus_tag=SPOA0056;product=DNA-binding protein, putative"	30	49	23	19	29	29
CDS;ID=637613601;locus_tag=SPOA0057;product=glycine cleavage system T protein	44	54	31	36	40	43
CDS;ID=637613602;locus_tag=SPOA0058;product=glycine cleavage system H protein	58	35	33	38	37	48
CDS;ID=637613603;locus_tag=SPOA0059;product=glycine dehydrogenase	86	60	40	60	116	57
"CDS;ID=637613604;locus_tag=SPOA0060;product=transcriptional regulator, AraC family	4	7	4	4	2	9
CDS;ID=637613605;locus_tag=SPOA0061;product=catalase/peroxidase HPI	106	81	84	47	69	70
CDS;ID=637613606;locus_tag=SPOA0062;product=aspartate racemase	5	5	4	3	5	10
CDS;ID=637613607;locus_tag=SPOA0063;product=FAD dependent oxidoreductase/aminomethy	13	18	9	5	15	15
"CDS;ID=637613608;locus_tag=SPOA0064;product=NG,NG-dimethylarginine dimethylaminohy	7	0	5	3	2	3
CDS;ID=637613609;locus_tag=SPOA0065;product=hypothetical protein	9	5	9	1	2	1
"CDS;ID=637613610;locus_tag=SPOA0066;product=aspartate aminotransferase, putative"	4	7	12	4	5	8
"CDS;ID=637613611;locus_tag=SPOA0067;product=transcriptional regulator, TetR family	6	13	4	4	8	10
"CDS;ID=637613612;locus_tag=SPOA0068;product=polar amino acid uptake family ABC tra	21	29	10	10	21	22
"CDS;ID=637613613;locus_tag=SPOA0069;product=polar amino acid uptake family ABC tra	9	8	8	10	10	17
"CDS;ID=637613614;locus_tag=SPOA0070;product=polar amino acid uptake family ABC tra	5	5	3	6	1	3
"CDS;ID=637613615;locus_tag=SPOA0071;product=polar amino acid uptake family ABC tra	6	8	1	7	5	5
CDS;ID=637613616;locus_tag=SPOA0072;product=hypothetical protein	626	508	609	711	967	667
CDS;ID=637613617;locus_tag=SPOA0073;product=arsenate reductase	142	108	124	112	196	150
CDS;ID=637613618;locus_tag=SPOA0074;product=hypothetical protein	42	36	57	51	28	51
CDS;ID=637613619;locus_tag=SPOA0075;product=hypothetical protein	91	86	103	80	73	95
"CDS;ID=637613620;locus_tag=SPOA0076;product=hydrolase, carbon-nitrogen family"	114	104	74	58	86	100
CDS;ID=637613621;locus_tag=SPOA0077;product=translation initiation factor IF-1	162	130	117	147	126	155
CDS;ID=637613622;locus_tag=SPOA0078;product=maf protein	18	21	22	23	40	28
"CDS;ID=637613623;locus_tag=SPOA0079;product=ribonuclease, Rne/Rng family"	83	48	62	73	78	97
CDS;ID=637613624;locus_tag=SPOA0450;product=hypothetical protein	18	8	12	22	13	14
tRNA;ID=640706686;locus_tag=SPOA_tRNA-Phe-1	443	149	71	87	23	110
CDS;ID=637613626;locus_tag=SPOA0081;product=hypothetical protein	101	93	78	86	124	81
CDS;ID=637613627;locus_tag=SPOA0082;product=hypothetical protein	66	58	63	53	66	81
CDS;ID=637613628;locus_tag=SPOA0083;product=hypothetical protein	100	116	105	83	129	98
CDS;ID=637613629;locus_tag=SPOA0084;product=hypothetical protein	1	1	3	1	0	5
"CDS;ID=637613630;locus_tag=SPOA0085;product=DNA-binding protein, putative"	7	5	5	2	14	10
CDS;ID=637613631;locus_tag=SPOA0086;product=hypothetical protein	23	20	4	16	10	19
"CDS;ID=637613632;locus_tag=SPOA0087;product=site-specific recombinase, phage integ	13	6	7	11	7	6
CDS;ID=637613633;locus_tag=SPOA0088;product=hypothetical protein	1104	1120	1021	1052	1168	1253
CDS;ID=637613634;locus_tag=SPOA0089;product=hypothetical protein	4	1	2	1	2	3
"CDS;ID=637613635;locus_tag=SPOA0090;product=ISSpo7, transposase"	3	0	0	4	2	1
CDS;ID=637613636;locus_tag=SPOA0091;product=IS3 family transposase orfA	3	3	14	7	2	5
CDS;ID=640731104;locus_tag=SPOA0092	0	0	1	1	0	2
CDS;ID=637613637;locus_tag=SPOA0093;product=FAD binding domain protein	126	67	85	101	118	144
CDS;ID=637613638;locus_tag=SPOA0094;product=hypothetical protein	11	17	18	17	19	9
CDS;ID=637613639;locus_tag=SPOA0095;product=hypothetical protein	15	19	21	15	22	16

CDS;ID=637613640;locus_tag=SPOA0096;product=histone deacetylase/AcuC/AphA family pı	22	17	16	7	6	14
"CDS;ID=637613641;locus_tag=SPOA0097;product=branched-chain amino acid ABC transpoı	7	6	5	5	16	11
"CDS;ID=637613642;locus_tag=SPOA0098;product=branched-chain amino acid ABC transpoı	9	13	7	15	11	9
"CDS;ID=637613643;locus_tag=SPOA0099;product=branched-chain amino acid ABC transpoı	17	7	6	13	15	16
"CDS;ID=637613644;locus_tag=SPOA0100;product=branched-chain amino acid ABC transpoı	19	18	11	13	23	33
"CDS;ID=637613645;locus_tag=SPOA0101;product=branched-chain amino acid ABC transpoı	48	52	34	46	68	47
"CDS;ID=637613646;locus_tag=SPOA0102;product=transcriptional regulator, LuxR family	15	15	14	13	14	9
CDS;ID=637613647;locus_tag=SPOA0103;product=hypothetical protein	49	43	32	37	54	58
CDS;ID=637613648;locus_tag=SPOA0104;product=aldehyde dehydrogenase family protein	25	16	13	17	31	21
"CDS;ID=637613649;locus_tag=SPOA0105;product=transcriptional regulator, putative"	3	7	5	3	5	8
CDS;ID=637613650;locus_tag=SPOA0106;product=hypothetical protein	6	10	8	16	6	13
"CDS;ID=637613651;locus_tag=SPOA0107;product=transcriptional regulator, LysR family	27	27	19	30	25	43
CDS;ID=637613652;locus_tag=SPOA0108;product=hypothetical protein	1	9	3	15	10	6
"CDS;ID=637613653;locus_tag=SPOA0451;product=transcriptional regulator, MarR family	104	80	73	80	127	82
"CDS;ID=637613654;locus_tag=SPOA0452;product=transcriptional regulator, MarR family	76	67	46	61	82	72
CDS;ID=637613655;locus_tag=SPOA0109;product=hypothetical protein	55	38	40	50	38	51
CDS;ID=637613656;locus_tag=SPOA0110;product=indolepyruvate ferredoxin oxidoreductas	18	14	11	20	18	13
"CDS;ID=637613657;locus_tag=SPOA0111;product=indolepyruvate oxidoreductase, IorA su	16	8	11	28	11	11
CDS;ID=637613658;locus_tag=SPOA0112;product=phenylacetaldehyde dehydrogenase	4	4	4	2	8	5
CDS;ID=637613659;locus_tag=SPOA0113;product=hypothetical protein	5	9	9	3	8	13
CDS;ID=637613660;locus_tag=SPOA0114;product=nitrilase family protein	2	0	3	0	5	4
"CDS;ID=637613661;locus_tag=SPOA0115;product=gentisate 1,2-dioxygenase"	2	3	1	2	8	10
CDS;ID=637613662;locus_tag=SPOA0116;product=fumarylacetoacetate hydrolase family pı	4	0	3	1	3	3
CDS;ID=637613663;locus_tag=SPOA0117;product=Asp/Glu/hydantoin racemase family prote	5	4	5	2	2	1
CDS;ID=637613664;locus_tag=SPOA0118;product=hypothetical protein	3	4	4	2	6	6
CDS;ID=637613665;locus_tag=SPOA0119;product=hypothetical protein	8	9	7	1	10	16
CDS;ID=637613666;locus_tag=SPOA0120;product=hypothetical protein	40	33	33	34	37	33
CDS;ID=637613667;locus_tag=SPOA0121;product=sulfatase family protein	15	24	18	20	25	27
"CDS;ID=637613668;locus_tag=SPOA0122;product=ISSpo9, transposase"	9	8	2	7	7	5
CDS;ID=637613669;locus_tag=SPOA0123;product=hypothetical protein	91	101	52	63	113	105
CDS;ID=637613670;locus_tag=SPOA0124;product=hypothetical protein	132	110	84	79	119	119
CDS;ID=637613671;locus_tag=SPOA0125;product=hypothetical protein	69	46	45	45	64	71
"CDS;ID=637613672;locus_tag=SPOA0126;product=oxidoreductase, short chain dehydroger	5	2	4	0	3	7
CDS;ID=637613673;locus_tag=SPOA0127;product=cyclase family protein	5	6	6	16	10	12
"CDS;ID=637613674;locus_tag=SPOA0128;product=oxidoreductase, short chain dehydroger	7	8	5	2	10	9
CDS;ID=637613675;locus_tag=SPOA0129;product=hypothetical protein	9	2	5	5	9	3
CDS;ID=637613676;locus_tag=SPOA0130;product=AMP-binding enzyme family protein	13	7	11	10	11	8
"CDS;ID=637613677;locus_tag=SPOA0131;product=aromatic 1,2-dioxygenase, beta subunit	2	8	11	4	3	4
"CDS;ID=637613678;locus_tag=SPOA0132;product=aromatic 1,2-dioxygenase, alpha subuni	10	9	17	17	21	13
CDS;ID=637613679;locus_tag=SPOA0133;product=oxidoreductase FAD-binding domain/oxido	5	6	5	4	9	12
"CDS;ID=637613680;locus_tag=SPOA0134;product=ISSpo7, transposase"	1	6	3	4	4	5
tRNA;ID=640706687;locus_tag=SPOA_tRNA-Thr-1	1149	548	313	236	42	302
CDS;ID=637613682;locus_tag=SPOA0136;product=hypothetical protein	113	92	86	113	124	68

CDS;ID=637613683;locus_tag=SPOA0137;product=UDP-N-acetylglucosamine 1-carboxyvinylt	68	44	50	65	75	62
CDS;ID=637613684;locus_tag=SPOA0138;product=hypothetical protein	11	10	6	9	7	14
"CDS;ID=637613685;locus_tag=SPOA0139;product=acetyltransferase, GNAT family"	17	12	11	9	13	14
CDS;ID=637613686;locus_tag=SPOA0140;product=glucosamine--fructose-6-phosphate aminc	30	29	32	23	24	37
CDS;ID=637613687;locus_tag=SPOA0141;product=hypothetical protein	32	30	21	20	15	27
CDS;ID=637613688;locus_tag=SPOA0142;product=hypothetical protein	17	16	11	12	13	21
"CDS;ID=637613689;locus_tag=SPOA0143;product=transcriptional regulator, IclR family	18	8	11	8	20	19
"CDS;ID=637613690;locus_tag=SPOA0144;product=serine--glyoxylate transaminase, putat	4	5	4	1	5	4
CDS;ID=637613691;locus_tag=SPOA0145;product=pyridoxal-phosphate dependent enzyme	13	6	18	7	12	11
"CDS;ID=637613692;locus_tag=SPOA0146;product=threonine aldolase, low-specificity"	11	22	12	17	13	10
CDS;ID=637613693;locus_tag=SPOA0147;product=ornithine cyclodeaminase/mu-crystallin	10	8	7	5	1	1
"CDS;ID=637613694;locus_tag=SPOA0148;product=membrane protein, major facilitator tı	0	0	2	1	0	0
"CDS;ID=637613695;locus_tag=SPOA0149;product=transcriptional regulator, GntR family	2	4	2	1	0	1
"CDS;ID=637613696;locus_tag=SPOA0150;product=transcriptional regulator, AraC family	5	7	6	2	8	4
CDS;ID=637613697;locus_tag=SPOA0151;product=cytochrome c family protein	55	46	37	39	53	55
CDS;ID=637613698;locus_tag=SPOA0152;product=di-haem cytochrome c peroxidase family	53	52	52	39	71	49
CDS;ID=637613699;locus_tag=SPOA0153;product=hypothetical protein	67	57	42	64	101	60
CDS;ID=637613700;locus_tag=SPOA0154;product=hypothetical protein	84	90	92	100	156	75
CDS;ID=637613701;locus_tag=SPOA0155;product=type I secretion target repeat protein	112	88	72	85	161	137
CDS;ID=637613702;locus_tag=SPOA0156;product=hypothetical protein	80	43	75	76	108	91
CDS;ID=637613703;locus_tag=SPOA0157;product=hypothetical protein	6	2	7	7	4	5
CDS;ID=637613704;locus_tag=SPOA0158;product=D-cysteine desulfhydrase	3	6	8	7	8	2
"CDS;ID=637613705;locus_tag=SPOA0159;product=transcriptional regulator, LysR family	11	1	5	6	3	4
"CDS;ID=637613706;locus_tag=SPOA0160;product=TRAP dicarboxylate transporter, DctM s	31	16	26	27	26	21
"CDS;ID=637613707;locus_tag=SPOA0161;product=TRAP dicarboxylate transporter, DctQ s	1	0	3	5	4	1
"CDS;ID=637613708;locus_tag=SPOA0162;product=TRAP dicarboxylate family transporter,	9	6	2	3	0	5
"CDS;ID=637613709;locus_tag=SPOA0163;product=hydroxymethylglutaryl-CoA reductase, d	7	8	9	3	6	7
"CDS;ID=637613710;locus_tag=SPOA0164;product=transcriptional regulator, GntR family	8	4	12	2	9	11
"CDS;ID=637613711;locus_tag=SPOA0165;product=transcriptional regulator, LysR family	11	13	4	12	7	12
"CDS;ID=637613712;locus_tag=SPOA0166;product=TRAP transporter solute receptor, TAXI	4	0	5	4	6	3
"CDS;ID=637613713;locus_tag=SPOA0167;product=TRAP transporter, 4TM/12TM fusion prot	29	15	16	17	31	28
"CDS;ID=637613714;locus_tag=SPOA0168;product=oxidoreductase, FAD-binding"	9	7	11	5	7	9
CDS;ID=637613715;locus_tag=SPOA0169;product=MOSC domain protein	17	11	9	13	13	19
"CDS;ID=637613716;locus_tag=SPOA0170;product=transcriptional regulator, MarR family	21	26	9	6	18	28
CDS;ID=637613717;locus_tag=SPOA0171;product=hypothetical protein	16	13	15	30	52	25
CDS;ID=637613718;locus_tag=SPOA0172;product=hypothetical protein	7	1	5	8	13	6
CDS;ID=637613719;locus_tag=SPOA0173;product=hypothetical protein	9	3	1	5	4	2
CDS;ID=637613720;locus_tag=SPOA0174;product=MOSC domain protein	1	5	7	5	2	1
CDS;ID=637613721;locus_tag=SPOA0175;product=oxidoreductase FAD-binding domain/oxidc	12	12	5	5	8	12
CDS;ID=637613722;locus_tag=SPOA0176;product=hypothetical protein	4	2	3	5	4	2
"CDS;ID=637613723;locus_tag=SPOA0177;product=hydrogen peroxide-inducible genes acti	3	3	1	7	2	4
CDS;ID=640731105;locus_tag=SPOA0179	5	5	4	6	5	7
CDS;ID=637613724;locus_tag=SPOA0180;product=cytochrome c family protein	10	2	5	6	11	3

"CDS;ID=637613725;locus_tag=SPOA0181;product=transporter, FNT family"	6	13	9	5	7	9
CDS;ID=637613726;locus_tag=SPOA0182;product=hypothetical protein	3	1	1	0	2	4
CDS;ID=637613727;locus_tag=SPOA0183;product=SCO1/SenC family protein	0	1	0	0	0	0
CDS;ID=637613728;locus_tag=SPOA0184;product=heme-copper respiratory oxidase family	10	11	19	11	8	8
"CDS;ID=637613729;locus_tag=SPOA0185;product=cytochrome c oxidase,ba3-type, subunit	2	1	2	3	3	1
CDS;ID=637613730;locus_tag=SPOA0186;product=Rrf2 family protein	17	22	12	13	17	11
"CDS;ID=637613731;locus_tag=SPOA0187;product=cytochrome c oxidase, cbb3-type, subur	12	10	14	6	7	13
"CDS;ID=637613732;locus_tag=SPOA0188;product=cytochrome c oxidase, cbb3-type, subur	1	1	0	2	1	1
"CDS;ID=637613733;locus_tag=SPOA0189;product=cytochrome c oxidase, cbb3-type, subur	10	9	5	10	13	8
"CDS;ID=637613734;locus_tag=SPOA0190;product=cytochrome c oxidase, cbb3-type, subur	11	16	8	10	16	10
CDS;ID=637613735;locus_tag=SPOA0191;product=hypothetical protein	3	6	4	1	5	4
CDS;ID=637613736;locus_tag=SPOA0192;product=hypothetical protein	4	9	10	5	12	1
CDS;ID=637613737;locus_tag=SPOA0193;product=cytochrome c-type biogenesis protein Cy	37	34	28	43	33	36
CDS;ID=637613738;locus_tag=SPOA0194;product=5-aminolevulinate synthase	2	4	3	2	4	5
CDS;ID=637613739;locus_tag=SPOA0195;product=hypothetical protein	2	1	0	2	1	3
CDS;ID=637613740;locus_tag=SPOA0196;product=cytochrome c-type biogenesis protein Cy	0	0	0	2	0	1
CDS;ID=637613741;locus_tag=SPOA0197;product=thiol:disulfide interchange protein Dsl	2	2	1	0	0	1
CDS;ID=637613742;locus_tag=SPOA0198;product=cytochrome c-type biogenesis protein Cy	3	4	4	1	3	5
"CDS;ID=637613743;locus_tag=SPOA0199;product=transcriptional regulator, GntR family	3	7	13	3	7	14
CDS;ID=637613744;locus_tag=SPOA0200;product=hypothetical protein	11	5	12	5	6	11
CDS;ID=637613745;locus_tag=SPOA0201;product=hydroxylamine oxidoreductase	16	6	10	7	1	15
CDS;ID=637613746;locus_tag=SPOA0202;product=histidinol dehydrogenase	27	21	16	18	25	22
CDS;ID=637613747;locus_tag=SPOA0203;product=hypothetical protein	42	42	27	28	56	40
CDS;ID=637613748;locus_tag=SPOA0204;product=hypothetical protein	22	33	24	29	36	42
"CDS;ID=637613749;locus_tag=SPOA0205;product=serine protease, subtilase family"	76	33	39	30	42	54
"CDS;ID=637613750;locus_tag=SPOA0206;product=transcriptional regulator, Crp/Fnr far	17	12	15	14	18	28
CDS;ID=637613751;locus_tag=SPOA0207;product=adenylate/guanylate cyclase	139	118	91	98	109	136
"CDS;ID=637613752;locus_tag=SPOA0208;product=oxidoreductase, FAD-binding"	385	351	304	331	419	418
"CDS;ID=637613753;locus_tag=SPOA0209;product=oxidoreductase, FAD-binding"	14	23	16	2	4	7
CDS;ID=637613754;locus_tag=SPOA0210;product=integral membrane protein	26	23	11	16	16	20
CDS;ID=637613755;locus_tag=SPOA0211;product=Na/Pi-cotransporter family protein	75	115	69	71	81	92
"CDS;ID=637613756;locus_tag=SPOA0212;product=nitric oxide reductase F protein, puta	0	0	0	0	1	2
"CDS;ID=637613757;locus_tag=SPOA0213;product=nitric oxide reductase E protein, puta	0	0	1	1	0	2
CDS;ID=637613758;locus_tag=SPOA0214;product=nitric oxide reductase D protein	4	6	0	0	2	4
CDS;ID=637613759;locus_tag=SPOA0215;product=nitric oxide reductase Q protein	6	2	0	3	4	9
"CDS;ID=637613760;locus_tag=SPOA0216;product=nitric oxide reductase, large subunit'	3	14	6	5	14	11
"CDS;ID=637613761;locus_tag=SPOA0217;product=nitric oxide reductase, small subunit'	11	0	6	3	7	7
"CDS;ID=637613762;locus_tag=SPOA0218;product=magnesium transporter, CorA family"	4	4	6	0	3	1
"CDS;ID=637613763;locus_tag=SPOA0219;product=NnrS, putative"	6	3	10	2	4	5
CDS;ID=637613764;locus_tag=SPOA0220;product=cytochrome cd1 nitrite reductase	11	2	5	10	10	7
CDS;ID=637613765;locus_tag=SPOA0221;product=nitrite reductase heme biosynthesis E p	3	1	1	0	2	0
CDS;ID=637613766;locus_tag=SPOA0222;product=cytochrome c55X	0	0	0	0	2	0
CDS;ID=637613767;locus_tag=SPOA0223;product=cytochrome cd1 nitrite reductase	7	3	1	1	6	3

"CDS;ID=637613768;locus_tag=SPOA0224;product=nitrite reductase heme biosynthesis D/	0	2	3	0	0	0
CDS;ID=637613769;locus_tag=SPOA0225;product=nitrite reductase heme biosynthesis G p	0	0	0	0	1	0
CDS;ID=637613770;locus_tag=SPOA0226;product=nitrite reductase heme biosynthesis H p	0	1	0	0	1	0
CDS;ID=637613771;locus_tag=SPOA0227;product=nitrite reductase heme biosynthesis J p	4	1	0	1	1	2
CDS;ID=637613772;locus_tag=SPOA0228;product=nitrite reductase protein N	3	3	3	2	2	6
CDS;ID=637613773;locus_tag=SPOA0229;product=hypothetical protein	0	0	0	0	1	0
"CDS;ID=637613774;locus_tag=SPOA0230;product=transcriptional regulator, LysR family	21	28	23	19	23	20
"CDS;ID=637613775;locus_tag=SPOA0231;product=glycine betaine/proline ABC transporte	2	2	1	2	3	1
"CDS;ID=637613776;locus_tag=SPOA0232;product=glycine betaine/proline ABC transporte	3	2	1	2	2	8
"CDS;ID=637613777;locus_tag=SPOA0233;product=glycine betaine/proline ABC transporte	9	2	1	3	4	8
"CDS;ID=637613778;locus_tag=SPOA0234;product=agmatinase, putative"	2	2	0	1	9	2
CDS;ID=637613779;locus_tag=SPOA0235;product=agmatinase	4	2	0	1	3	1
CDS;ID=637613780;locus_tag=SPOA0236;product=C4-dicarboxylate transport sensor prote	15	21	20	18	24	23
CDS;ID=637613781;locus_tag=SPOA0237;product=C4-dicarboxylate transport transcriptio	10	6	8	7	11	20
"CDS;ID=637613782;locus_tag=SPOA0238;product=TRAP dicarboxylate transporter, DctP s	44	33	24	25	32	45
"CDS;ID=637613783;locus_tag=SPOA0239;product=TRAP dicarboxylate transporter, DctQ s	9	2	2	5	3	6
"CDS;ID=637613784;locus_tag=SPOA0240;product=TRAP transporter, DctM subunit"	10	11	8	4	7	18
"CDS;ID=637613785;locus_tag=SPOA0241;product=gluconolactonase, putative"	1	7	7	6	7	3
"CDS;ID=637613786;locus_tag=SPOA0242;product=transcriptional regulator, LacI family	33	39	37	44	36	53
CDS;ID=637613787;locus_tag=SPOA0243;product=aldehyde dehydrogenase family protein	36	37	22	40	31	45
CDS;ID=637613788;locus_tag=SPOA0244;product=dihydrodipicolinate synthase family pro	1	5	3	7	7	7
"CDS;ID=637613789;locus_tag=SPOA0245;product=galactonate dehydratase, putative"	15	7	13	15	13	19
CDS;ID=637613790;locus_tag=SPOA0246;product=4-hydroxythreonine-4-phosphate dehydrog	5	12	11	9	9	15
CDS;ID=637613791;locus_tag=SPOA0247;product=hypothetical protein	4	2	3	4	0	2
CDS;ID=637613792;locus_tag=SPOA0248;product=hypothetical protein	1	0	0	1	0	1
"CDS;ID=637613793;locus_tag=SPOA0249;product=TRAP dicarboxylate transporter, DctP s	6	7	6	2	8	3
"CDS;ID=637613794;locus_tag=SPOA0250;product=TRAP dicarboxylate transporter, DctM s	6	6	4	12	7	7
"CDS;ID=637613795;locus_tag=SPOA0251;product=TRAP dicarboxylate transporter, DctQ s	10	8	7	4	10	5
"CDS;ID=637613796;locus_tag=SPOA0252;product=transcriptional regulator, LacI family	32	11	13	8	18	24
"CDS;ID=637613797;locus_tag=SPOA0253;product=ribose ABC transporter, periplasmic ri	6	5	3	6	6	9
"CDS;ID=637613798;locus_tag=SPOA0254;product=ribose ABC transporter, permease prote	3	10	9	7	6	10
"CDS;ID=637613799;locus_tag=SPOA0255;product=sugar ABC transporter, ATP binding pro	0	0	0	0	0	2
"CDS;ID=637613800;locus_tag=SPOA0256;product=ribose ABC transporter, periplasmic ri	4	3	3	4	3	8
"CDS;ID=637613801;locus_tag=SPOA0257;product=ribose ABC transporter, permease prote	11	3	0	10	5	10
"CDS;ID=637613802;locus_tag=SPOA0258;product=ribose ABC transporter, ATP-binding pr	8	1	4	1	1	9
"CDS;ID=637613803;locus_tag=SPOA0259;product=transcriptional regulator, LacI family	29	37	23	28	31	38
CDS;ID=637613804;locus_tag=SPOA0260;product=aldehyde dehydrogenase family protein	1	3	10	7	4	6
"CDS;ID=637613805;locus_tag=SPOA0261;product=transcriptional regulator, GntR family	31	15	35	24	36	34
"CDS;ID=637613806;locus_tag=SPOA0262;product=oxidoreductase, FAD-binding"	1	2	2	1	3	1
"CDS;ID=637613807;locus_tag=SPOA0263;product=TRAP transporter, DctM subunit"	7	4	6	3	5	8
"CDS;ID=637613808;locus_tag=SPOA0264;product=TRAP transporter, DctQ family"	2	1	2	4	0	3
"CDS;ID=637613809;locus_tag=SPOA0265;product=TRAP transporter, DctP family, putativ	2	9	5	5	4	4
"CDS;ID=637613810;locus_tag=SPOA0266;product=proline racemase, putative"	6	1	3	2	1	5

CDS;ID=637613811;locus_tag=SPOA0267;product=dihydroxydipicolinate synthase family p	1	6	2	3	10	7
"CDS;ID=637613812;locus_tag=SPOA0268;product=transcriptional regulator, IclR family	23	14	16	14	24	12
CDS;ID=637613813;locus_tag=SPOA0269;product=hypothetical protein	11	5	8	8	14	12
CDS;ID=637613814;locus_tag=SPOA0270;product=hypothetical protein	4	2	2	2	1	2
"CDS;ID=637613815;locus_tag=SPOA0271;product=methylamine utilization protein MauG,	3	2	1	2	3	4
CDS;ID=637613816;locus_tag=SPOA0272;product=glutathione-dependent formaldehyde dehy	15	21	17	12	12	12
"CDS;ID=637613817;locus_tag=SPOA0273;product=DNA-binding protein, putative"	14	17	9	15	20	12
CDS;ID=637613818;locus_tag=SPOA0274;product=4-aminobutyrate aminotransferase	7	6	7	8	14	3
CDS;ID=637613819;locus_tag=SPOA0275;product=succinate-semialdehyde dehydrogenase	4	2	5	1	4	3
CDS;ID=637613820;locus_tag=SPOA0276;product=fructokinase	2	2	3	3	1	5
"CDS;ID=637613821;locus_tag=SPOA0277;product=hydrolase, alpha/beta fold family"	3	4	0	2	2	2
"CDS;ID=637613822;locus_tag=SPOA0278;product=TRAP dicarboxylate transporter, DctM s	10	5	4	9	9	10
"CDS;ID=637613823;locus_tag=SPOA0279;product=TRAP dicarboxylate transporter, DctQ s	3	1	1	2	1	2
"CDS;ID=637613824;locus_tag=SPOA0280;product=TRAP dicarboxylate transporter, DctP s	7	8	2	4	4	6
"CDS;ID=637613825;locus_tag=SPOA0281;product=transcriptional regulator, LysR family	3	7	4	7	1	5
CDS;ID=637613826;locus_tag=SPOA0282;product=malonyl-CoA synthase	77	66	58	49	109	92
"CDS;ID=637613827;locus_tag=SPOA0283;product=oxidoreductase, GMC family"	0	0	0	2	2	1
"CDS;ID=637613828;locus_tag=SPOA0284;product=transcriptional regulator, LysR family	1	6	0	2	1	2
CDS;ID=637613829;locus_tag=SPOA0285;product=carnitinyl-CoA dehydratase	7	6	4	8	3	9
CDS;ID=637613830;locus_tag=SPOA0286;product=ThiJ/PfpI family protein	21	17	12	12	16	17
"CDS;ID=637613831;locus_tag=SPOA0287;product=acyl-CoA synthetase, putative"	6	4	9	2	10	5
CDS;ID=637613832;locus_tag=SPOA0288;product=acyl-CoA dehydrogenase	1	5	0	4	0	3
"CDS;ID=637613833;locus_tag=SPOA0289;product=transcriptional regulator, AraC family	4	4	2	3	3	5
CDS;ID=637613834;locus_tag=SPOA0290;product=hypothetical protein	10	15	10	8	10	7
"CDS;ID=637613835;locus_tag=SPOA0291;product=branched-chain amino acid aminotransfe	77	88	85	65	95	114
CDS;ID=637613836;locus_tag=SPOA0292;product=phosphatidylserine decarboxylase	43	35	33	43	56	38
CDS;ID=637613837;locus_tag=SPOA0293;product=CDP-diacylglycerol--serine O-phosphatic	56	57	68	56	72	93
CDS;ID=637613838;locus_tag=SPOA0294;product=phosphatidylethanolamine N-methyltransf	71	66	52	54	67	64
CDS;ID=637613839;locus_tag=SPOA0295;product=hydantoin racemase	7	1	2	4	2	5
"CDS;ID=637613840;locus_tag=SPOA0296;product=branched-chain amino acid ABC transpor	2	4	3	3	5	5
"CDS;ID=637613841;locus_tag=SPOA0297;product=branched-chain amino acid ABC transpor	0	2	2	3	1	5
"CDS;ID=637613842;locus_tag=SPOA0298;product=branched-chain amino acid ABC transpor	1	6	4	6	5	5
"CDS;ID=637613843;locus_tag=SPOA0299;product=branched-chain amino acid ABC transpor	8	5	3	4	5	2
"CDS;ID=637613844;locus_tag=SPOA0300;product=branched-chain amino acid ABC transpor	10	14	10	14	8	13
"CDS;ID=637613845;locus_tag=SPOA0301;product=transcriptional regulator, GntR family	43	51	67	32	58	66
"CDS;ID=637613846;locus_tag=SPOA0302;product=site-specific recombinase, phage integ	3	0	4	0	2	0
"CDS;ID=637613847;locus_tag=SPOA0303;product=replication protein, putative"	6	8	11	1	12	5
CDS;ID=637613848;locus_tag=SPOA0304;product=replication protein	8	9	6	8	9	7
CDS;ID=637613849;locus_tag=SPOA0305;product=hypothetical protein	8	18	9	7	20	12
CDS;ID=637613850;locus_tag=SPOA0306;product=S1 RNA binding domain protein	80	61	51	83	114	101
CDS;ID=640731106;locus_tag=SPOA0308	11	5	15	7	8	11
"CDS;ID=637613851;locus_tag=SPOA0309;product=sulphoacetaldehyde acetyltransferase,	15	6	12	4	10	13
CDS;ID=637613852;locus_tag=SPOA0310;product=aminomethyl transferase family protein	8	16	5	7	3	21

CDS;ID=637613853;locus_tag=SPOA0311;product=FAD dependent oxidoreductase/aminomethy	5	10	7	12	10	15
"CDS;ID=637613854;locus_tag=SPOA0312;product=glutamate-1-semialdehyde 2,1-aminomuta	1	2	3	4	0	5
CDS;ID=637613855;locus_tag=SPOA0313;product=trimethylamine methyltransferase family	11	1	5	9	7	9
"CDS;ID=637613856;locus_tag=SPOA0314;product=transcriptional regulator, LysR family	16	20	11	9	14	24
"CDS;ID=637613857;locus_tag=SPOA0315;product=isocitrate dehydrogenase, NADP-depende	60	66	70	89	136	133
CDS;ID=637613858;locus_tag=SPOA0316;product=amidohydrolase domain protein	71	57	57	39	54	59
"CDS;ID=637613859;locus_tag=SPOA0317;product=transcriptional regulator, AsnC family	5	4	1	2	6	5
CDS;ID=637613860;locus_tag=SPOA0318;product=methionine gamma-lyase	41	48	45	30	41	42
"CDS;ID=637613861;locus_tag=SPOA0319;product=transketolase, putative"	74	59	57	54	45	51
CDS;ID=637613862;locus_tag=SPOA0320;product=5-carboxymethyl-2-hydroxymuconate delta	0	3	0	0	1	2
CDS;ID=637613863;locus_tag=SPOA0321;product=glyoxalase family protein	4	4	2	1	4	10
CDS;ID=637613864;locus_tag=SPOA0322;product=hypothetical protein	28	28	26	35	37	28
CDS;ID=637613865;locus_tag=SPOA0323;product=cyclic nucleotide-binding protein	50	32	25	26	31	58
CDS;ID=637613866;locus_tag=SPOA0324;product=hypothetical protein	22	9	10	20	13	21
"CDS;ID=637613867;locus_tag=SPOA0325;product=oxidoreductase, 2-nitropropane dioxyge	6	3	7	11	6	8
"CDS;ID=637613868;locus_tag=SPOA0326;product=transcriptional regulator, LysR family	4	4	1	7	5	6
"CDS;ID=637613869;locus_tag=SPOA0327;product=rzcC protein, putative"	21	26	18	12	21	27
CDS;ID=637613870;locus_tag=SPOA0328;product=hypothetical protein	6	9	8	3	6	7
CDS;ID=637613871;locus_tag=SPOA0329;product=methylglyoxal synthase	1	3	2	4	2	3
CDS;ID=637613872;locus_tag=SPOA0330;product=2-dehydro-3-deoxyphosphogluconate aldol	2	9	6	3	8	7
"CDS;ID=637613873;locus_tag=SPOA0331;product=2-dehydro-3-deoxygluconokinase, putati	16	7	11	14	11	7
CDS;ID=637613874;locus_tag=SPOA0332;product=dihydroxy-acid dehydratase	13	14	6	8	14	19
"CDS;ID=637613875;locus_tag=SPOA0333;product=TRAP dicarboxylate transporter, DctM s	2	8	7	3	5	7
"CDS;ID=637613876;locus_tag=SPOA0334;product=TRAP dicarboxylate family transporter,	3	2	1	3	4	1
"CDS;ID=637613877;locus_tag=SPOA0335;product=TRAP dicarboxylate family transporter,	11	5	4	4	8	15
"CDS;ID=637613878;locus_tag=SPOA0336;product=transcriptional regulator, LacI family	74	52	61	78	88	78
CDS;ID=637613879;locus_tag=SPOA0337;product=hypothetical protein	68	49	51	30	43	43
"CDS;ID=637613880;locus_tag=SPOA0338;product=transcriptional regulator, LysR family	75	93	70	15	9	12
"CDS;ID=637613881;locus_tag=SPOA0339;product=HAD-superfamily hydrolase, subfamily I	725	758	535	23	24	9
"CDS;ID=637613882;locus_tag=SPOA0340;product=tautomerase, putative"	206	208	189	8	5	3
CDS;ID=637613883;locus_tag=SPOA0341;product=hypothetical protein	234	247	242	13	11	11
CDS;ID=637613884;locus_tag=SPOA0342;product=hypothetical protein	891	866	871	35	34	20
CDS;ID=637613885;locus_tag=SPOA0343;product=hypothetical protein	805	784	790	21	21	8
"CDS;ID=637613886;locus_tag=SPOA0344;product=transcriptional regulator, LysR family	35	24	36	11	20	18
"CDS;ID=637613887;locus_tag=SPOA0345;product=oxidoreductase, aldo/keto reductase fa	2660	2311	2238	57	46	12
CDS;ID=637613888;locus_tag=SPOA0346;product=carboxymuconolactone decarboxylase fami	321	330	323	12	9	0
CDS;ID=637613889;locus_tag=SPOA0347;product=hypothetical protein	430	427	378	19	10	13
CDS;ID=637613890;locus_tag=SPOA0348;product=hypothetical protein	77	66	48	13	11	31
CDS;ID=637613891;locus_tag=SPOA0349;product=hypothetical protein	15	12	3	3	5	5
CDS;ID=637613892;locus_tag=SPOA0350;product=hypothetical protein	8	2	6	3	10	6
CDS;ID=637613893;locus_tag=SPOA0351;product=hypothetical protein	16	2	11	5	15	9
CDS;ID=637613894;locus_tag=SPOA0352;product=hypothetical protein	35	17	27	13	31	29
CDS;ID=637613895;locus_tag=SPOA0353;product=aldehyde dehydrogenase family protein	7	12	4	9	9	15

"CDS;ID=637613896;locus_tag=SPOA0354;product=aminotransferase, class III"	9	9	3	10	3	13
CDS;ID=637613897;locus_tag=SPOA0355;product=hypothetical protein	13	14	18	12	19	33
CDS;ID=637613898;locus_tag=SPOA0356;product=hypothetical protein	7	6	4	4	6	4
CDS;ID=637613899;locus_tag=SPOA0357;product=DNA-binding response regulator	24	14	17	15	14	24
CDS;ID=637613900;locus_tag=SPOA0358;product=sensor histidine kinase	24	14	13	13	8	10
CDS;ID=637613901;locus_tag=SPOA0359;product=cytochrome c family protein	0	1	5	3	4	0
"CDS;ID=637613902;locus_tag=SPOA0360;product=multicopper oxidase, putative"	11	7	0	2	8	8
CDS;ID=637613903;locus_tag=SPOA0361;product=cytochrome c family protein	3	3	0	3	2	2
CDS;ID=637613904;locus_tag=SPOA0362;product=27kDa outer membrane protein	0	2	2	0	0	0
CDS;ID=637613905;locus_tag=SPOA0363;product=hypothetical protein	1	0	0	0	2	2
CDS;ID=637613906;locus_tag=SPOA0364;product=hypothetical protein	2	3	4	1	5	2
CDS;ID=637613907;locus_tag=SPOA0365;product=copper-translocating P-type ATPase	4	2	2	4	4	3
CDS;ID=637613908;locus_tag=SPOA0366;product=amino acid permease	13	7	7	4	6	11
"CDS;ID=637613909;locus_tag=SPOA0367;product=ABC transporter, permease protein"	2	5	4	4	4	1
CDS;ID=637613910;locus_tag=SPOA0368;product=hypothetical protein	4	5	4	6	5	10
"CDS;ID=637613911;locus_tag=SPOA0369;product=copper resistance protein B, putative'	6	4	3	5	5	6
CDS;ID=637613912;locus_tag=SPOA0370;product=copper resistance protein A	11	9	8	11	10	14
CDS;ID=637613913;locus_tag=SPOA0371;product=hypothetical protein	15	4	5	3	7	9
"CDS;ID=637613914;locus_tag=SPOA0372;product=TRAP dicarboxylate transporter, DctQ s	9	11	7	6	1	17
"CDS;ID=637613915;locus_tag=SPOA0373;product=TRAP dicarboxylate transporter, DctM s	3	1	1	5	2	2
"CDS;ID=637613916;locus_tag=SPOA0374;product=TRAP dicarboxylate transporter, DctP s	1	4	2	0	4	4
"CDS;ID=637613917;locus_tag=SPOA0375;product=transcriptional regulator, gntR family	28	34	17	21	21	19
CDS;ID=637613918;locus_tag=SPOA0376;product=epoxide hydrolase domain protein	5	2	4	1	4	5
CDS;ID=637613919;locus_tag=SPOA0377;product=aldehyde dehydrogenase family protein	7	5	1	4	2	8
CDS;ID=637613920;locus_tag=SPOA0378;product=mandelate racemase/muconate lactonizing	5	9	3	9	9	14
"CDS;ID=637613921;locus_tag=SPOA0379;product=transcriptional regulator, GntR family	3	0	4	7	6	4
CDS;ID=637613922;locus_tag=SPOA0380;product=hypothetical protein	10	10	6	10	8	12
"CDS;ID=637613923;locus_tag=SPOA0381;product=spermidine/putrescine ABC transporter,	6	5	0	4	6	14
"CDS;ID=637613924;locus_tag=SPOA0382;product=spermidine/putrescine ABC transporter,	5	3	3	0	8	0
"CDS;ID=637613925;locus_tag=SPOA0383;product=spermidine/putrescine ABC transporter,	3	4	3	3	2	3
"CDS;ID=637613926;locus_tag=SPOA0384;product=spermidine/putrescine ABC transporter,	4	5	5	4	3	6
CDS;ID=637613927;locus_tag=SPOA0385;product=hypothetical protein	7	8	8	3	6	11
"CDS;ID=637613928;locus_tag=SPOA0386;product=oxidoreductase, GMC family"	7	5	1	2	7	2
"CDS;ID=637613929;locus_tag=SPOA0387;product=transcriptional regulator, LysR family	10	4	7	7	2	8
"CDS;ID=637613930;locus_tag=SPOA0388;product=acetyl-CoA synthetase, putative"	4	1	1	2	2	1
"CDS;ID=637613931;locus_tag=SPOA0389;product=transcriptional regulator, MerR family	9	2	6	6	2	6
"CDS;ID=637613932;locus_tag=SPOA0390;product=phosphonate monoester hydrolase, putat	68	40	33	29	34	54
CDS;ID=637613933;locus_tag=SPOA0391;product=cyclic nucleotide-binding protein	2	3	2	0	2	3
"CDS;ID=637613934;locus_tag=SPOA0392;product=RNA polymerase sigma-70 factor, putat:	0	0	0	1	0	1
CDS;ID=637613935;locus_tag=SPOA0393;product=R body protein RebB homolog	0	0	0	0	0	1
CDS;ID=637613936;locus_tag=SPOA0394;product=hypothetical protein	1	0	0	1	1	1
CDS;ID=637613937;locus_tag=SPOA0395;product=hypothetical protein	5	7	5	6	7	6
CDS;ID=637613938;locus_tag=SPOA0396;product=hypothetical protein	0	0	2	0	2	0

CDS;ID=637613939;locus_tag=SPOA0397;product=hypothetical protein	0	1	0	0	1	0
CDS;ID=637613940;locus_tag=SPOA0398;product=R body protein RebB homolog	2	1	0	1	3	0
CDS;ID=637613941;locus_tag=SPOA0399;product=R body protein RebB homolog	0	1	0	1	1	2
CDS;ID=637613942;locus_tag=SPOA0400;product=endoribonuclease L-PSP family protein	10	5	7	10	9	10
CDS;ID=637613943;locus_tag=SPOA0401;product=benzoate-coenzyme A ligase	15	12	8	15	15	15
CDS;ID=637613944;locus_tag=SPOA0402;product=polyketide synthesis domain protein	1	3	0	3	7	5
CDS;ID=637613945;locus_tag=SPOA0403;product=acyl-CoA dehydrogenase family protein	1	2	5	8	9	8
CDS;ID=637613946;locus_tag=SPOA0404;product=enoyl-CoA hydratase	8	8	5	3	9	5
"CDS;ID=637613947;locus_tag=SPOA0405;product=transcriptional regulator, MarR family	3	0	0	1	2	1
"CDS;ID=637613948;locus_tag=SPOA0406;product=D-beta-hydroxybutyrate dehydrogenase,	12	4	8	4	3	10
CDS;ID=637613949;locus_tag=SPOA0407;product=hypothetical protein	33	42	22	27	43	48
CDS;ID=637613950;locus_tag=SPOA0408;product=hypothetical protein	7	12	4	5	7	7
"CDS;ID=637613951;locus_tag=SPOA0409;product=tryptophan 2,3-dioxygenase, putative"	3	2	6	5	4	5
CDS;ID=637613952;locus_tag=SPOA0410;product=hypothetical protein	412	299	258	331	328	344
CDS;ID=637613953;locus_tag=SPOA0411;product=hypothetical protein	260	179	165	206	188	200
"CDS;ID=637613954;locus_tag=SPOA0412;product=xanthine dehydrogenase family protein,	74	77	49	52	70	52
"CDS;ID=637613955;locus_tag=SPOA0413;product=xanthine dehydrogenase family protein,	36	20	16	24	39	23
"CDS;ID=637613956;locus_tag=SPOA0414;product=xanthine dehydrogenase family protein,	76	62	51	74	89	109
CDS;ID=637613957;locus_tag=SPOA0415;product=hypothetical protein	9	10	7	14	17	9
CDS;ID=637613958;locus_tag=SPOA0416;product=hypothetical protein	11	8	12	14	6	11
"CDS;ID=637613959;locus_tag=SPOA0417;product=transcriptional regulator, GntR family	9	12	4	8	9	6
"CDS;ID=637613960;locus_tag=SPOA0418;product=acetolactate synthase, large subunit,	10	9	5	6	7	15
"CDS;ID=637613961;locus_tag=SPOA0419;product=phenazine biosynthesis protein, PhzF f	6	0	4	3	3	1
CDS;ID=637613962;locus_tag=SPOA0420;product=hypothetical protein	2	1	2	0	2	2
CDS;ID=637613963;locus_tag=SPOA0421;product=pyridoxamine 5'-phosphate oxidase fami	0	2	3	1	0	4
CDS;ID=637613964;locus_tag=SPOA0422;product=hypothetical protein	5	2	1	0	1	2
"CDS;ID=637613965;locus_tag=SPOA0423;product=transcriptional regulator, TetR family	55	39	52	65	54	69
"CDS;ID=637613966;locus_tag=SPOA0424;product=fatty oxidation complex, alpha subunit	27	7	23	13	16	20
CDS;ID=637613967;locus_tag=SPOA0425;product=acetyl-CoA acetyltransferase	1	4	2	4	6	6
"CDS;ID=637613968;locus_tag=SPOA0426;product=transcriptional regulator, AraC family	13	14	6	5	6	10
"CDS;ID=637613969;locus_tag=SPOA0427;product=transcriptional regulator, ArsR family	31	36	22	24	19	39
CDS;ID=637613970;locus_tag=SPOA0428;product=mandelate racemase/muconate lactonizing	31	40	16	23	27	46
CDS;ID=637613971;locus_tag=SPOA0429;product=hypothetical protein	43	41	52	32	38	51
"CDS;ID=637613972;locus_tag=SPOA0430;product=oxidoreductase, zinc-binding dehydroge	13	13	22	17	16	25
CDS;ID=637613973;locus_tag=SPOA0431;product=tetracycline resistance protein	40	31	17	13	21	41
"CDS;ID=637613974;locus_tag=SPOA0432;product=3-carboxy-cis,cis-muconate cycloisomer	33	31	15	35	32	28
"CDS;ID=637613975;locus_tag=SPOA0433;product=esterase EstC, putative"	33	30	27	25	34	17
CDS;ID=637613976;locus_tag=SPOA0434;product=3-oxoadipate enol-lactone hydrolase	61	64	53	65	61	90
CDS;ID=637613977;locus_tag=SPOA0435;product=pyridoxal-phosphate dependent enzyme	18	18	9	21	19	14
"CDS;ID=637613978;locus_tag=SPOA0436;product=hydrolase, alpha/beta fold family"	24	23	16	21	28	13
"CDS;ID=637613979;locus_tag=SPOA0437;product=haloacid dehalogenase, type II"	7	6	1	2	4	9
"CDS;ID=637613980;locus_tag=SPOA0438;product=peptidyl-prolyl cis-trans isomerase, F	13	16	7	8	19	14
CDS;ID=637613981;locus_tag=SPOA0439;product=enoyl-(acyl-carrier-protein) reductase	164	103	122	161	103	160

CDS;ID=637613982;locus_tag=SPOA0440;product=3-oxoacyl-(acyl-carrier-protein) syntha	205	187	148	193	253	247
CDS;ID=637613983;locus_tag=SPOA0441;product=hypothetical protein	13	7	11	10	3	21
CDS;ID=637613984;locus_tag=SPOA0442;product=hypothetical protein	23	20	25	31	13	27
CDS;ID=637613985;locus_tag=SPOA0443;product=hypothetical protein	9	10	4	11	9	4
CDS;ID=637613986;locus_tag=SPOA0444;product=3-hydroxydecanoyl-ACP dehydratase	139	116	100	118	127	139
"CDS;ID=637613987;locus_tag=SPOA0445;product=transcriptional regulator, Fur family"	11	6	14	7	6	18
"CDS;ID=637613988;locus_tag=SPOA0446;product=transcriptional regulator, LysR family	14	10	8	6	8	3
