# Supplementary material for: A Model Roseobacter, Ruegeria pomeroyi DSS-3, Employs a Diffusible Killing Mechanism To Eliminate Competitors
Source: mSystems. 2020 Aug 11;5(4):e00443-20. doi: 10.1128/mSystems.00443-20 (PMC7426152; doi:10.1128/mSystems.00443-20)
Supplement: TEXT S1 [file mSystems.00443-20-s0001.docx]

**Supplemental Materials and Methods**

**Strains and Isolation Conditions**

All strains, including those provided by other labs, are listed in Supplemental Table S3. *Microbacterium sp*. RAM275 was isolated on Zobell marine agar from a research cruise in the North Atlantic (EN556). *Sulfitobacter sp*. RAM1190 and Idiomarina sp. RAM1191 were isolated on 1/10^th^ YTSS, also from a research cruise in the North Atlantic (EN584). *Phaeobacter* *daeponensis* and *Phaeobacter sp*. ANS2052 were both isolated on Zobell marine agar from the shells of oysters from the coast of North Carolina. *Micrococcus sp.* RAM1600 was isolated on LBS media from Bogue Sound, North Carolina, and *Alteromonas sp.* RAM1611 and *Saccharospirillum sp.* RAM1647 were isolated on 1/10^th^ YTSS from the same location. All strains were purified by restreaking three times from single colonies. In all of our competitions, these environmental strains were grown on ½ YTSS at 29˚C.

**Tagged Strains and plasmid constructs.** Tagged strains were constructed in order to coculture DSS-3 and competing strains and then separate and enumerate them. DSS-3 Tn5-Km (DSS-3 Kn) was created by conjugation with *E. coli* RHO3+ pUT mini Tn5-Km, a diaminopimelic acid (DAP) auxotroph containing a kanamycin-resistant transposon delivery vector. For conjugation, DSS-3 WT and RHO3+ pUT mini Tn5-Km were grown in liquid media overnight in ½ YTSS at 29˚C or in LB media containing 100 µM DAP and 40 µg mL^-1^ kanamycin at 37˚C, respectively. 40 µL of DSS-3 culture was mixed with 160 µL of RHO3+ pUT mini Tn5-Km culture and centrifuged at 15,000 rpm for 3 minutes. The supernatant was removed and the pellet was then resuspended in fresh ½ YTSS liquid media and centrifuged at 15,000 rpm for 3 minutes. Most of the supernatant was then removed, and the pellet was resuspended in the remaining media and spotted onto ½ YTSS agar containing 100 µM DAP. The conjugation spot was incubated for 16 hours at 29˚C and was then resuspended in 500 µL ½ YTSS and centrifuged at 10000 rpm for 5 minutes. The DAP-containing supernatant was removed, and the sample was resuspended in 100 µL of fresh ½ YTSS media and plated onto ½ YTSS agar plates supplemented with 100 µg mL^-1^ kanamycin. One DSS-3 mutant colony (DSS-3 Kn) was selected from the plate and grown on DAP-free 100 µg mL^-1^ Kan ½ YTSS plates. The site of the transposon insertion for this mutant was located by inverse PCR (described below) and was found to have a non-disruptive insertion between genes *lepA* (SPO0983) and *znuB* (SPO0984) that did not cause a statistically different growth rate compared to DSS-3 WT (Fig. S1). DSS-3 Kn was therefore selected for use as the tagged wild-type strain.

Red-fluorescent tagged target strains were created using the pBBR1MCS-mCherry plasmid, which conveys chloramphenicol resistance. A gene encoding the red fluorescent protein, mCherry, was amplified from pTM214 plasmid (Miyashiro, 2011) with primers AS1152 (ATGGATCCGACATCATAACGGTTCTGGC) and AS1153 (ATGGATCCGTCTCTTGTACACATCTTGC) and was digested with BamHI enzyme (New England Biolabs, Ipswich, MA) overnight at 37˚C. The pBBR1MCS plasmid was extracted from *E. coli* DH5α pBBR1MCS using the ZR Plasmid Miniprep kit (Zymo Research, Irvine, CA) and was also digested overnight with BamHI enzyme at 37˚C. The cut plasmid and PCR product were ligated for 15 minutes at room temperature using T4 DNA ligase (New England Biolabs, Ipswich, MA). The ligation reaction was transformed into chemically competent *E. coli* DH5α cells and plated onto LB agar plates supplemented with 20 µg mL^-1^ chloramphenicol. The pBBR1MCS-mCherry plasmid (pGS001) was then transformed into *E. coli* RHO3+ cells, and the RHO3+ cells were cultured overnight in liquid and combined at a 1:1 volume ratio with DSS-3 and the roseobacters *Sagittula stellata* E-37 (Gonzalez, 1997), and *Roseovarius sp.* TM1035 (Miller, 2004), spotted on 100 µM DAP ½ YTSS plates, and incubated for 24 hours at 29°C before plating on DAP-free ½ YTSS plates supplemented with 2 µg mL^-1^ chloramphenicol. The resulting roseobacter strains containing pGS001 showed poor growth and irregular colony morphology on any concentration of chloramphenicol, so we decided to only use pGS001 to select for *E. coli* DH5α in the competition experiments.

To fluorescently tag the other target strains, the gene encoding mCherry was subcloned from pGS001 into the pBBR1MCS-5 plasmid, which is a gentamicin resistant variant of pBBR1MCS. Both plasmids were extracted from DH5α pGS001 and DH5α pBBR1MCS-5 and digested for one hour with BamHI enzyme at 37˚C. To dephosphorylate the ends of cut pBBR1MCS-5 vector, rSAP (New England Biolabs, Ipswich, MA) was added to the pBBR1MCS-5 BamHI digest, followed by an additional 30 minute incubation to prevent self-ligation in downstream steps. Both digests were cleaned and concentrated using the DNA Clean and Concentrator-5 Kit (Zymo Research, Irvine, CA) and quantified, and then 150 ng of each plasmid digest was ligated using T4 DNA ligase (New England Biolabs, Ipswich, MA). The resulting ligation was transformed into chemically competent *E*. coli DH5α cells and plated onto LB agar plates supplemented with 40 µg mL^-1^ gentamicin. A red-fluorescent gentamicin-resistant *E. coli* DH5α colony was selected from the plate, grown up in 40 µg mL^-1^ gentamicin LB media at 37°C, and stocked down. The pBBR1MCS-5 mCherry plasmid (pGS002) was then transformed into *E. coli* RHO3+ cells in order to conjugate the plasmid into the target strains. Overnight RHO3+ pGS002 liquid culture was mixed with liquid cultures of each target strain at a 1:1 volume ratio (100 µL RHO3+ pBBR1MCS-5 mCherry: 100 µL target strain). Preparation of this mixture for spotting was performed as described above for generation of DSS-3 Tn5-Km, except with a 24 hour incubation at 29˚C. Two strains, *Sagittula stellata* E-37 pGS002 and *Roseovarius* sp. TM1035 pGS002, required plating on 100 µg mL^-1^ gentamicin ½ YTSS plates. The other strains with pGS002, *Phaeobacter sp.* ANS2052, *Sulfitobacter sp*. RAM1190, *Phaeobacter daeponensis*, *Ruegeria sp.* RAM1602, *Idiomarina* sp. RAM1191, *Alteromonas sp.* RAM1611, *Sacchospirillium sp.* RAM1647, *Micrococcus sp.* RAM275, and *Microbacterium sp.* RAM1600, all required plating on 70 µg mL^-1^ gentamicin ½ YTSS plates. A previously constructed tagged *Vibrio fischeri* ES114 strain carrying the plasmid pVSV208, was used and required growth on LBS agar plates supplemented with 2 µg mL^-1^ chloramphenicol.

**Coincubation assays.** For coincubations on agar plates, tagged strains were cultured overnight in the appropriate media containing the proper concentration of antibiotic (kanamycin for DSS-3 Kn, gentamicin or chloramphenicol for target strains) to select for only the tagged strain. The starter cultures were then separated from the antibiotic-containing media by centrifuging at 10 000 rpm for 5 minutes and discarding the supernatant, and the cell pellet was then resuspended in fresh antibiotic-free ½ YTSS media. The two cultures were both normalized to an OD_600_ of 1.0, and then mixed at a DSS-3 to target OD_600_ ratio of either 1:1 or 9:1 depending on the experiment. The competition assays were then started by spotting 5 µl of the coculture mixture into one well of a 24 well plate containing 1 mL of ½ YTSS agar and incubated (24 hrs at 29˚C). The initial cell concentrations of each strain within the mixture were enumerated by serially diluting the coculture mixture, incubating, and then counting colony forming units (CFUs). Final strain cell concentrations were determined by resuspending the culture spot in 1 mL of ½ YTSS, performing a serial dilution, and then spotting 5 µl of each dilution onto the appropriate selective media for each strain.

Filter separation experiments were carried out by spotting a 20 µL DSS-3 Kn culture (OD_600 =_ 1) onto ½ YTSS plates, toping it with a 0.2 µm nitrocellulose filter (Millipore Sigma, Burlington, MA), and then placing 5 µL TM1035 pGS002 (OD_600 =_ 1) culture on top. No strain on bottom and a 20 µL spot of 100 µg/mL kanamycin served as negative and positive killing controls for diffusible antimicrobials, respectively. To control for the possibility of growth-inhibition of the target strain above the filter by overgrowth of the strain below the filter, a differentially tagged TM1035 strain (TM1035 Kn) was placed on the bottom, and there was no statistical difference in growth of the above TM1035 when grown above another TM1035 strain or above no other strain. Initial strain concentrations were enumerated by plating on selective media and counting CFUs. After incubating for 24 hrs at 29 ˚C, the filters were removed from the plate, and placed in 1.5 ml microcentrifuge tubes containing 1 mL ½ YTSS liquid media and vortexed for 10 minutes to resuspend the adhered bacteria, and the CFU counts of the TM1035 pGS002 target strain were enumerated by plating serial dilutions on selective media and counting CFUs.

**Competition assays in liquid media**. Overnight 15 mL starter cultures of each strain were incubated in ½ YTSS liquid media containing the appropriate antibiotic concentrations at 29˚C at 200 rpm. The cultures were then centrifuged (4000 rpm, 10 minutes), the supernatant was removed, and the pellet was resuspended in 3 mL of ½ YTSS without antibiotic. The resuspended culture optical densities were measured and the two competing strains were mixed in a 15 mL ½ YTSS coculture such that the DSS-3 Kn killer strain had a final OD_600_ of 0.2 and the target TM1035 pBBR1MCS-5 strain had a final OD_600_ of 0.02. Dilution co-cultures were also created (2X, 4X, 6X, and 8X) using 5 mL of the undiluted coculture to determine whether killing was dependent on the concentration of the killer strain. All cocultures were then incubated (29˚C, shaken at 200 rpm). Population densities at 0, 2, 4, 6, and 8 hours were determined by removing an aliquot of the coculture, performing a serial dilution, and plating it on selective media and counting CFUs.

**Transposon mutant library generation and screening.** A DAP auxotrophic *E. coli* strain (RHO3+ pUT mini Tn5-Km) containing the transposon delivery vector was grown in LB media containing 100 µM DAP and 40 µg/mL kanamycin for 16 hours. Wild-type DSS-3 was grown in ½ YTSS for 16 hours. The *E. coli* and DSS-3 cultures were mixed at a 4:1 volumetric ratio (160 µL *E. coli* :40 µL DSS-3) and centrifuged (15 000 rpm, 3 min). The supernatant was removed, and the pellet was washed with 1 mL ½ YTSS and centrifuged again (15 000 rpm, 3 min). All but 10 µL of media was removed, and this remaining supernatant was used to resuspend the pellet. This conjugation mixture was spotted into the wells of a 24 well plate containing 1 mL of ½ YTSS agar with 100 µM DAP. The conjugation spots were incubated for 16 hr at 29˚C and then resuspended in 500 µL ½ YTSS by pipetting within the well. The resuspended sample was transferred to a microcentrifuge tube and centrifuged (10 000 rpm, 5 minutes), and the DAP containing supernatant was removed. The conjugation mixture was then resuspended in 100 µL of fresh ½ YTSS media and plated onto ½ YTSS agar plates with 100 µg/mL kanamycin to select for DSS-3 Tn5-Km mutants. Each conjugation spot yielded 250-500 mutants.

The mutant library was screened for the non-killing phenotype by patching each mutant onto agar overlay plates containing TM1035 pGS002, which is killed by wild-type DSS-3. The TM1035 agar overlay plates were made by diluting an overnight TM1035 pGS002 culture in ½ YTSS media with 1% agarose to an OD_600_ of 0.005, and then pouring the culture agarose mix over pre-prepared 1.5% agar ½ YTSS plates and allowing it to solidify overnight at room temperature. DSS-3 transposon mutants were then patched on top of the overlay as well as onto ½ YTSS plates with 100 µg/mL kanamycin, and after incubating for 24 hours, the patches were examined using a fluorescence dissecting microscope to identify zones of killing in the fluorescent TM1035 overlay. DSS-3 transposon mutants that retained the ability to kill created a zone of clearing around the patched colony and were discarded, while mutants that no longer could create a zone of clearing were taken from the corresponding antibiotic plate, restreaked, placed into 100 µg/mL kanamycin ½ YTSS liquid media and grown overnight at 29˚C at 200 rpm, and stocked down at -80˚C for further study and sequencing. Ten thousand mutants were generated and screened. We considered the mutant library to be saturated when three different mutants were identified with transposon insertions in the same gene.

**Mapping transposon insertion sites via inverse PCR.** Mutants identified as losing the killing phenotype were cultured overnight in ½ YTSS media containing 100 µg/mL kanamycin, and their DNA was extracted using the ZR Fungal/Bacterial DNA Miniprep Kit (Zymo Research, Irvine, CA). The transposon insertion sites were then identified by inverse PCR as follows (Ochman 1990). Two micrograms of mutant genomic DNA was digested overnight using BssHII enzyme (New England Biolabs, Ipswich, MA) in a 50 µL reaction and then concentrated to 20 µL using the ZR DNA Clean and Concentrator-5 kit. The resulting linear genomic DNA fragments were circularized using T4 DNA ligase. DNA flanking the transposon insertion was amplified by PCR using EconoTaq DNA polymerase, one of three primers that binds to the 5’-end of the transposon (5’-endseq, AS1193, or GS010), and one of three primers that binds to the 3’-end of the transposon (3’-endseq, AS1196, or GS009). Approximately 100 ng of BssHII digested and ligated mutant DSS-3 DNA was used for each iPCR amplification, and the PCR protocol is as follows: 95˚C for 3 min followed by 5 cycles of (95˚C 30 s- 60˚C 1 min- 72˚C 4 min), then 30 cycles of (95˚C 30 s- 55˚C 30 s- 72˚C 2 min), and a final extension step at 72˚C for 1min. The resulting PCR products were visualized via gel electrophoresis, cleaned and concentrated using the ZR DNA Clean and Concentrator-5 kit, quantified using a spectrophotometer, and then Sanger sequenced by Eton Bio. The identities of each gene were found using NCBI-BLASTn. Mutation sites were confirmed by amplifying the transposon insertion site of uncut genomic DNA using a transposon-specific primer and a primer that is specific to the flanking DNA sequence. PCR parameters: 94˚C for 2min followed by 30 cycles of (94˚C 30s- 55˚C 30s- 72˚C 45s) and a final extension step at 72˚C for 2 min). Primer sets used to confirm each mutation insertion site were as follows: DSS-3 Kn (GS007-AS1193), GCS64 (GS001-AS1196), GCS121 (GS008-AS1196), GCS122 (GS008-GS009), GCS124 (GS001-GS009), GCS134 (GS013-GS010), GCS140 (GS015-AS1196), and GCS141 (GS014-GS009). Gene cluster function was determined through the use of the anti-SMASH program (Weber 2015). All primer sequences can be found in Supplemental Figure S1.

**Sequencing and Analysis of Competition Transcriptomes.** Liquid suspension competitions were set up as described above by mixing either DSS-3 Kn or the DSS-3 SPOA0342 mutant (GCS64) with TM1035 pBBR1MCS-5 at a 1:1 OD_600_ ratio in triplicate 10 mL co-cultures (each strain at a final OD_600_ of 0.2) . The competition assay was subsampled regularly for up to 24 hours to determine strain population densities via serial dilutions on selective media plates. At 1.5 hours after starting the experiment, 5 mL of co-culture was filtered through a 0.22 µm polyethersulfone filter, and the filters were flash frozen in liquid nitrogen and stored at -80˚C to preserve the RNA until extraction. RNA was extracted from the filters using the Mirvana RNA Extraction kit (Invitrogen, Carlsbad, CA). Residual DNA was removed using the TURBO DNA-free Kit (Invitrogen, Carlsbad, CA). cDNA libraries were prepared using the ScriptSEQ v2 kit and barcodes (Epicentre, Madison, WI) and sequenced with the HiSEQ4000 platform (PE 50x50).

The following workflow was used for processing the transcriptome data. Each raw Illumina file was checked by FastQC to calculate initial quantity and quality of the forward and reverse reads. FastQ Groomr was used to convert the Illumina file to fastqsanger format. For trimming, the initial ILLUMINACLIP step was used (TruSeq3 adapters, max mismatch= 2, match accuracy for PE palindrome read alignment= 30, adapter sequence accuracy= 10) to remove adapter sequence. Low quality sequences were removed using trimmomatic (sliding window trimming with average quality lower than 20 averaged across 4 bases was removed). Quality of the trimmed reads was assessed using FastQC. Reads were mapped to the DSS-3 genome using a file containing a concatenated composite genome of both DSS-3 and TM1035 and Bowtie2 (local alignment, sensitive, disallow gaps within 4-positions of read). Total number of transcripts for each DSS-3 gene was quantified using the count intervals tool in Galaxy.

Genes with statistically different relative abundances were identified with DESeq using the DE App deSEQ2 (Li 2017). A table of read counts for each sample, with each row corresponding to a DSS-3 gene and each column corresponding to a transcriptome sample, and a metadata file labeling which transcriptome samples belong to the wild-type and mutant treatment groups were used as the data inputs for the DEApp Single-factor Experiment DESeq2 analysis (see supplemental table S2). Low expression mapped genetic features were removed if they were only expressed at a 1 count per million (CPM) value in at least 2 samples. For the DE analysis, group 1 was the mutant treatment (DSS-3 *afsA* mutant GCS64 vs TM1035), and group 2 was the wild-type treatment (DSS-3 Kn vs TM1035), with the filtering criteria being a p-value or FDR adjusted p-value lower than or equal to 0.05 and a fold change of at least 1.5.

**References**:

Miyashiro T, Klein W, Oehlert D, Cao X, Schwartzman J, Ruby EG. The N‐acetyl‐d‐glucosamine repressor NagC of *Vibrio fischeri* facilitates colonization of *Euprymna scolopes*. Molecular microbiology. 2011 Nov;82(4):894-903.

Gonzalez JM, Mayer F, Moran MA, Hodson RE, Whitman WB. *Sagittula stellata* gen. nov., sp. nov., a lignin-transforming bacterium from a coastal environment. International Journal of Systematic and Evolutionary Microbiology. 1997 Jul 1;47(3):773-80.

Miller TR, Belas R. Dimethylsulfoniopropionate metabolism by *Pfiesteria*-associated *Roseobacter* spp. Appl. Environ. Microbiol.. 2004 Jun 1;70(6):3383-91.

Ochman H, Medhora MM, Garza D, Hartl DL. Amplification of flanking sequences by inverse PCR. PCR protocols: A guide to methods and applications. 1990:219-27.

Weber T, Blin K, Duddela S, Krug D, Kim HU, Bruccoleri R, et al. antiSMASH 3.0-a comprehensive resource for the genome mining of biosynthetic gene clusters. Nucleic Acids Res. 2015;43(W1):W237-W43.

Li Y, Andrade J. DEApp: an interactive web interface for differential expression analysis of next generation sequence data. Source Code Biol Med. 2017;12:2.
