# Supplementary material for: A Model Roseobacter, Ruegeria pomeroyi DSS-3, Employs a Diffusible Killing Mechanism To Eliminate Competitors
Source: mSystems. 2020 Aug 11;5(4):e00443-20. doi: 10.1128/mSystems.00443-20 (PMC7426152; doi:10.1128/mSystems.00443-20)
Supplement: TABLE S3 [file mSystems.00443-20-st003.docx]

**Supplemental Table S3.** Strains, Plasmids, Oligo table

| Strains or Plasmids | Relevant characteristics | Source or Ref. |
| --- | --- | --- |
| ***E. coli*** |  |  |
| DH5α | F’/*endA1 hsdR17 glnV44 thi-1 recA1 gyrA relA1* Δ(*lacIZYA-argF*)*U169deoR*(f80*dlacI*Δ(*lacZ*)*M15*) | Hanahan 1983 |
| DH5αλ pir | λ*pir* derivative of DH5α | Dunn et al. 2005 |
| RHO3_+_ | SM10 (λ*pir*)^1^ Δ*asd*::*FRT* Δ*aphA*::*FRT* | Lopez et al. 2009 |
| ***R. pomeroyi*** |  |  |
| GCS002 | Wild-type | Gonzalez et al. 2003 |
| GCS005 | DSS-3 Kn; DSS-3 with intergenic disruption (Kan^R^) between *lepA* and *zncB* | This study |
| GCS64 | DSS-3 with SPOA0342 disruption (Kan^R^) | This study |
| GCS121 | DSS-3 with SPOA0341 disruption (Kan^R^) | This study |
| GCS122 | DSS-3 with SPOA0341 disruption (Kan^R^) | This study |
| GCS124 | DSS-3 with SPOA0342 disruption (Kan^R^) | This study |
| GCS134 | DSS-3 with SPO0900 disruption (Kan^R^) | This study |
| GCS140 | DSS-3 with SPOA0344 disruption (Kan^R^) | This study |
| GCS141 | DSS-3 with SPOA0342 disruption (Kan^R^) | This study |
| **Plasmids** |  |  |
| pUT mini Tn5-km | Tn5 transposon delivery vector, Km^R^ | Lorenzo et al. 1990 |
| pBBR1MCS | *lacZ, mob,* Cm^R^ | Kovach et al. 1994 |
| pBBR1MCS-5 | *lacZ, mob,* Gm^R^ | Kovach et al, 1995 |
| pGS001 | *mcherry+, lacZ, mob,* Cm^R^ | This study |
| pGS002 | *mcherry+, LacZ, mob,* Gm^R^ | This study |
| pVSV208 | *dsRed+, oriV*_R6Kγ_, *oriV*_pES213_, *oriT*, Cm^R^ | Dunn et al. 2006 |
| **Competitors^a^** |  |  |
| GCS006 | *Roseovarius* sp. TM1035 Tn5-Km | This study |
| GCS47 | *Roseovarius* sp. TM1035 pBBR1MCS-5 | This study |
| GCS51 | *Sagittula stellata* E-37 pGS002 | This study |
| GCS52 | *Roseovarius* sp. TM1035 pGS002 | This study |
| GCS53 | *Phaeobacter* sp. ANS2052 pGS002 | This study |
| GCS56 | *Phaeobacter daepensis* pGS002 | This study |
| GCS57 | *Sulfitobacter* sp. RAM1190 pGS002 | This study |
| GCS118 | *Ruegeria* sp. RAM1602 pGS002 | This study |
| GCS138 | *Idiomarina* sp. RAM1191 pGS002 | This study |
| GCS119 | *Alteromonas* sp. RAM1611 pGS002 | This study |
| GCS120 | *Saccharospirillium* sp. RAM1647 pGS002 | This study |
| GCS48 | *Vibrio fischeri* ES114 pVSV208 | Speare et al. 2018 |
| GCS16 | *Escherichia coli* DH5α pGS001 | This study |
| GCS137 | *Microbacterium* sp. RAM275 pGS002 | This study |
| GCS139 | *Micrococcus* sp. RAM1600 pGS002 | This study |
|  |  |  |
| **Oligonucleotides** |  |  |
| AS1152 | ATGGATCCGACATCATAACGGTTCTGGC | This study |
| AS1153 | ATGGATCCGTCTCTTGTACACATCTTGC | This study |
| 5’-endseq | TATTAACTCCTACTACACATTAAACTG | This study |
| 3’-endseq | ATGATTCAATATATTGTTAATAAACC | This study |
| AS1193 | GGATTTATCTCGCCCACGAGAGC | This study |
| AS1196 | CATTAAACGCGTATTCAGGCTGACC | This study |
| GS001 | CCATGAAGTTCGACACGTTG | This study |
| GS007 | GATTGCCACCTGAACCCTGA | This study |
| GS008 | GATTGCCACCTGAACCCTGA | This study |
| GS009 | GAGAGCCAGATTCCCGAGAAGA | This study |
| GS010 | GCGCAGGGCTTTATTGATTCC | This study |
| GS013 | GCTGGGCAAGGGTAAATCCT | This study |
| GS014 | CCTATCACTCGGTCGTCAGC | This study |
| GS015 | AGATCCTGCCCAAGTTTACGTC | This study |
|  |  |  |
|  |  |  |

^a^ For more details about competitor strain isolation, see Supplemental Information.

**References**:

Hanahan D. Studies on transformation of *Escherichia coli* with plasmids. Journal of molecular biology. 1983 Jun 5;166(4):557-80.

Dunn AK, Martin MO, Stabb EV. Characterization of pES213, a small mobilizable plasmid from *Vibrio fischeri*. Plasmid. 2005 Sep 1;54(2):114-34.

López CM, Rholl DA, Trunck LA, Schweizer HP. Versatile dual-technology system for markerless allele replacement in *Burkholderia* *pseudomallei*. Appl. Environ. Microbiol.. 2009 Oct 15;75(20):6496-503.

Gonzalez JM, Covert JS, Whitman WB, Henriksen JR, Mayer F, Scharf B, Schmitt R, Buchan A, Fuhrman JA, Kiene RP, Moran MA. *Silicibacter* *pomeroyi* sp. nov. and *Roseovarius nubinhibens* sp. nov., dimethylsulfoniopropionate-demethylating bacteria from marine environments. International journal of systematic and evolutionary microbiology. 2003 Sep 1;53(5):1261-9.

De Lorenzo V, Herrero M, Jakubzik U, Timmis KN. Mini-Tn5 transposon derivatives for insertion mutagenesis, promoter probing, and chromosomal insertion of cloned DNA in gram-negative eubacteria. Journal of bacteriology. 1990 Nov 1;172(11):6568-72.

Kovach ME, Phillips RW, Elzer PH, Peterson KM. pBBR1MCS: a broad-host-range cloning vector. BioTechniques. 1994 May;16(5):800-2.

Kovach ME, Elzer PH, Hill DS, Robertson GT, Farris MA, Roop II RM, Peterson KM. Four new derivatives of the broad-host-range cloning vector pBBR1MCS, carrying different antibiotic-resistance cassettes. Gene. 1995 Dec 1;166(1):175-6.

Dunn, A.K., Millikan, D.S., Adin, D.M., Bose, J.L. and Stabb, E.V., 2006. New rfp-and pES213-derived tools for analyzing symbiotic *Vibrio fischeri* reveal patterns of infection and lux expression in situ. *Appl. Environ. Microbiol.*, *72*(1), pp.802-810.

Liss, L., 1987. New M13 host: DH5αF′ competent cells. *Focus*, *9*(3), p.13.

Gonzalez JM, Mayer F, Moran MA, Hodson RE, Whitman WB. *Sagittula stellata* gen. nov., sp. nov., a lignin-transforming bacterium from a coastal environment. International Journal of Systematic and Evolutionary Microbiology. 1997 Jul 1;47(3):773-80.

Miller TR, Belas R. Dimethylsulfoniopropionate metabolism by *Pfiesteria*-associated Roseobacter spp. Appl. Environ. Microbiol.. 2004 Jun 1;70(6):3383-91.
